# Hierarchical genomic analysis of carried and invasive serogroup A *Neisseria meningitidis* during the 2011 epidemic in Chad

**DOI:** 10.1186/s12864-017-3789-0

**Published:** 2017-05-22

**Authors:** Kanny Diallo, Kadija Gamougam, Doumagoum M. Daugla, Odile B. Harrison, James E. Bray, Dominique A. Caugant, Jay Lucidarme, Caroline L. Trotter, Musa Hassan-King, James M. Stuart, Olivier Manigart, Brian M. Greenwood, Martin C. J. Maiden

**Affiliations:** 1 Centre pour les Vaccins en Développement, Bamako, Mali; 2 Centre de Support en Santé International, N’Djamena, Chad; 3 Department of Zoology, University of Oxford, Peter Medawar Building for Pathogen Research, South Parks Road, OX1 3SY Oxford, UK; 4 Norwegian Institute of Public Health, Oslo, Norway; 5 Vaccine Evaluation Unit, Public Health England, Manchester, UK; 6 Department of Veterinary Medicine, University of Cambridge, Cambridge, UK; 7 London School of Hygiene & Tropical Medicine, London, UK

**Keywords:** Whole genome sequencing, Meningitis epidemic, African meningitis belt, Pharyngeal carriage, Serogroup A *Neisseria meningitidis*

## Abstract

**Background:**

Serogroup A *Neisseria meningitidis* (*Nm*A) was the cause of the 2011 meningitis epidemics in Chad. This bacterium, often carried asymptomatically, is considered to be an “accidental pathogen”; however, the transition from carriage to disease phenotype remains poorly understood. This study examined the role genetic diversity might play in this transition by comparing genomes from geographically and temporally matched invasive and carried *Nm*A isolates.

**Results:**

All 23 *Nm*A isolates belonged to the ST-5 clonal complex (cc5). Ribosomal MLST comparison with other publically available *Nm*A:cc5 showed that isolates were closely related, although those from Chad formed two distinct branches and did not cluster with other *Nm*A, based on their MLST profile, geographical and temporal location. Whole genome MLST (wgMLST) comparison identified 242 variable genes among all Chadian isolates and clustered them into three distinct phylogenetic groups (Clusters 1, 2, and 3): no systematic clustering by disease or carriage source was observed. There was a significant difference (*p* = *0.0070*) between the mean age of the individuals from which isolates from Cluster 1 and Cluster 2 were obtained, irrespective of whether the person was a case or a carrier.

**Conclusions:**

Whole genome sequencing provided high-resolution characterization of the genetic diversity of these closely related *Nm*A isolates. The invasive meningococcal isolates obtained during the epidemic were not homogeneous; rather, a variety of closely related but distinct clones were circulating in the human population with some clones preferentially colonizing specific age groups, reflecting a potential age-related niche adaptation. Systematic genetic differences were not identified between carriage and disease isolates consistent with invasive meningococcal disease being a multi-factorial event resulting from changes in host-pathogen interactions along with the bacterium.

**Electronic supplementary material:**

The online version of this article (doi:10.1186/s12864-017-3789-0) contains supplementary material, which is available to authorized users.

## Background

*Neisseria meningitidis* (*Nm*) is a Gram-negative bacterium, which is frequently carried asymptomatically in the human nasopharynx. It is considered to be an “accidental pathogen”: a normally commensal organism that occasionally invades the bloodstream causing septicemia and/or meningitis. The factors that determine whether a person infected with *Nm* becomes a carrier or a case remain poorly understood. Meningococcal genetic and antigenic diversity are likely to be important and whole genome comparative analysis of carriage and disease isolates provides one means of investigating this. Different approaches have been used to compare carried and invasive isolates. Early studies compared the proportions of clonal complexes (cc), defined by Multi Locus Sequence Typing (MLST), among serogroups and identified hyper-virulent lineages that were overrepresented in invasive isolates and less common in carriage samples [1]. More recent studies have used whole genome technology to compare a broad range of disease and carriage isolates. These studies have, for example, identified a prophage present in some disease-associated isolates but not limited to them [2–4]. Another recent study compared carried and invasive serogroup Y *Nm* (*Nm*Y) from the UK and identified a disease-associated clone [5]; however, there is still much uncertainty over what determines the carried or invasive state, especially during epidemics.

For over 100 years, the Sahelian and sub-Sahelian regions of Africa, the African meningitis belt, have experienced large epidemics of meningococcal disease [6–13]. Serogroup A *N. meningitidis* (*Nm*A) was the most common cause in this region before the introduction of the TT-PsA conjugate vaccine (MenAfriVac®), which started in 2010 and will have been deployed in all 26 countries of the meningitis belt by the end of 2016, with 235 million doses administered at the time of writing. Since vaccine implementation, other previously less common groups including W (*Nm*W), C (*Nm*C), X (*Nm*X), and Y (*Nm*Y) have been more frequently associated with meningococcal disease in this region [14].

The southern part of the Republic of Chad lies in the African meningitis belt and has been subject to recurrent meningitis outbreaks since the early 1900s [15]. Since 2005, *Nm*W and *Nm*A have alternated as the major epidemic strains in small-scale outbreaks [16]. A large epidemic was recorded in Chad in 2011: seventeen districts reached the epidemic threshold of 10 per 100,000 per week and a total of 5960 suspect cases and 270 deaths were reported. Cerebrospinal fluid samples (CSFs) were obtained from only 3.8% of the cases for laboratory confirmation, but *Nm*A was the pathogen identified most commonly by culture and sero-agglutination methods [14]. Vaccination with MenAfriVac® of all subjects aged 1–29 years old was undertaken in the capital N’Djamena and in the surrounding area in 2012 with a dramatic impact on the epidemic, which continued in the rest of the country [15]. Vaccination of all previously unvaccinated areas the following year ended the epidemic and few cases of meningitis have been recorded since [17]. The MenAfriCar consortium, established in 2009 to study the carriage of *Nm* before and after the introduction of MenAfriVac® in the African meningitis belt, undertook three carriage surveys in the Mandelia district of Chad, two before and one after the vaccination campaign [15, 18]. Carriage of *Nm*A was low (<1%) prior to vaccination but fell to almost zero following vaccine implementation [15].

Isolates from serogroup A carriers and from patients with serogroup A invasive disease were retained, providing an opportunity to compare the genomic characteristics of carried and invasive isolates obtained during the same *Nm*A African epidemic. Here we describe the high-resolution provided by whole genome sequence (WGS) analysis and demonstrate how this can identify differences among closely related isolates. No systematic clustering by carried or invasive phenotype was observed, but three distinct clusters were identified circulating during the 2011 Chadian epidemic, two of them preferentially isolated from different age groups.

## Results

### Genome assembly statistics

High-quality draft genomes [19] were obtained for each isolate (Additional file 1: Table S1). In summary: the average number of contigs was 154 (range 113 to 243), the average N50 was 43378 (range 28,533 to 52,853); and the average length of the assembled genome was 2164341 (range 2,155,465 to 2,174,025). The average number of genes with allele designations, based on the 1605 *Neisseria* core genes list [19], was 1568 (97.7%) ranging from 1539 (95.9%) to 1577 (98.3%) loci.

### MLST analysis

The 23 isolates all had *Nm*A-associated capsule synthesis genes in region A of the capsule polysaccharide region of the genome [20], as expected, and belonged to cc5. Three different sequence types (ST) were found (Table 1), with their Multi-Locus Sequence Typing (MLST) profiles varying at up to two loci from the central Sequence Type, ST-5. The majority of isolates (21/23, 91%) were ST-7 and one isolate was ST-9021. Both STs had a single allelic difference at the *pgm* locus from ST-5. One invasive isolate lacked the *gdh* gene and therefore could not be assigned an ST. Comparison of the contig lacking *gdh* from this isolate with another isolate where *gdh* was present identified the deletion of six contiguous genes all involved in glucose metabolism, *NEIS1325*, *NEIS1326*, *NEIS1328* to *NEIS1331* (Additional file 1: Figure S1).

**Table 1:** Summary of MLST profiles

Number of isolates	*abcZ*	*adk*	*aroE*	*fumC*	*gdh*	*pdhC*	*pgm*	ST (MLST)	Clonal complex (MLST)
21	1	1	2	1	3	2	19	7	ST-5 complex/subgroup III
1	1	1	2	1	3	2	599	9021	ST-5 complex/subgroup III
1	1	1	2	1	-	2	19	ND	ST-5 complex/subgroup III

ND: Not Determined

### rMLST analysis

**Fig. 1:**
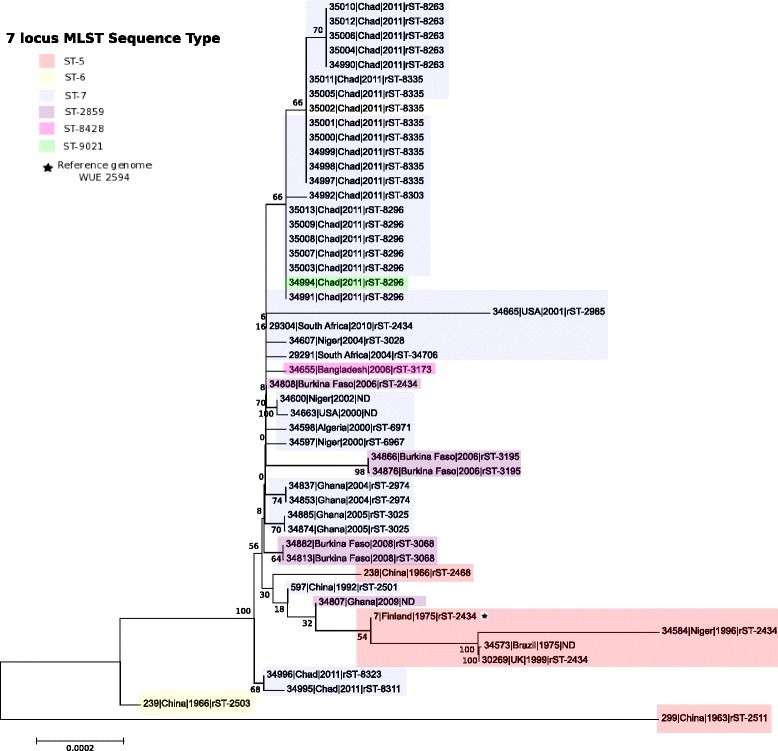
rMLST Neighbor-Joining Tree of cc5 *Nm*A. The relationship from the concatenated nucleotide sequences of the ribosomal genes between the *Nm*A isolates from Chad (*n* = 23) and other publically available cc5 *Nm*A genomes from the PubMLST database is represented in this tree. The label on each node indicates: the PubMLST ID number, the country, the date and the rST for each isolate represented. A total of 141 other cc5 *Nm*A isolates were found in PubMLST but only one representative of each unique strain (defined as isolates sharing the same alleles at all 53 ribosomal locus) was included in the tree alongside all the Chadian *Nm*A from the 2011 meningitis epidemic; the full list of publically available cc5 *NmA* isolates is shown in Additional file 1: Table S2. The seven-locus MLST profiles of the isolates are indicated by different colored boxes. The position of the reference genome used in this study (WUE 2594) is represented by a black star

Higher resolution relationships were obtained through the comparisons of the 53 ribosomal MLST (rMLST) loci, which identified six new rSTs: rST-8263; rST-8296; rST-8303; rST-8311; rST-8323; and rST-8335. The *Nm*A genomes from Chad exhibited allelic variation in 0 to 5 rMLST loci in pairwise comparisons, with an average of 1.4 loci different. Phylogenetic comparison with other *Nm*A:cc5 isolates, available through PubMLST.org/neisseria [21] (Additional file 1: Table S2), was performed including isolates from: a global collection of *Nm* isolates (*n* = 12) [1]; the African meningitis belt (*n* = 99) [22]; and other publically available isolates (*n* = 30) [23, 24]. The Neighbor-Joining Tree generated showed that most of the Chadian 2011 *Nm*A (21/23, 91%) formed a distinct branch (bootstrap value of 66); however, two isolates were found to cluster separately with a bootstrap value of a 100 (Fig. 1). Isolates most closely related to the 21 Chadian *Nm*A isolates were from South Africa, Niger, Bangladesh, USA and Burkina Faso, between 2001 and 2010 and exhibited several rSTs (rST-7, rST-8428 and rST-2859).

### Whole genome MLST (wgMLST) clusters isolates into 3 groups

**Fig. 2:**
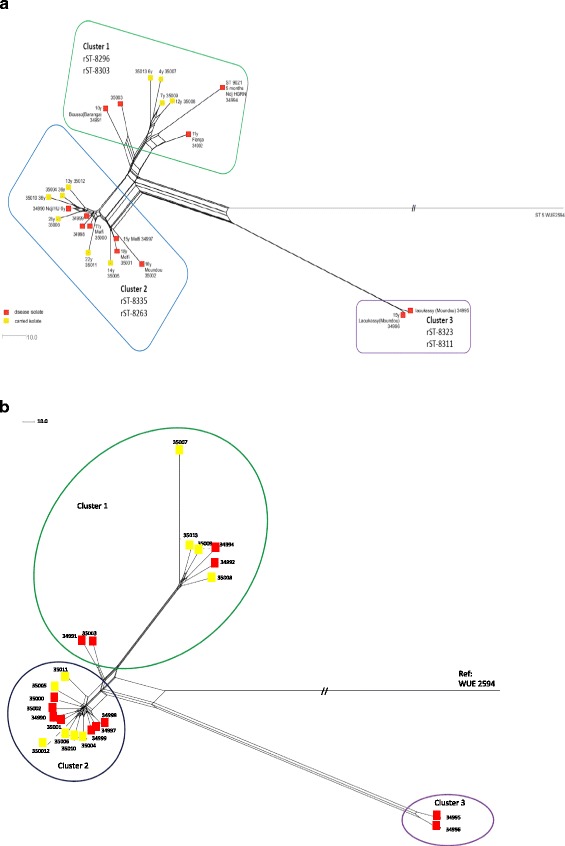
wgMLST and wgSNP Neighbor Net Tree. The genomic relationship based on wgMLST (2**a**) and wgSNP (2**b**) between the Chad *Nm*A isolates is depicted in relation to the reference genome WUE2594. Three clusters are observed and labeled on both trees. The invasive isolates are depicted in *red* and the carried ones in *yellow*. The rSTs contained in each cluster are also indicated as well as age and region of the patient/healthy volunteer are indicated when available, ND corresponds to the absence of any epidemiological information for the specific isolate (2**a**). The tree were produced based on a comparison in terms of *n* = 2070 loci defined in the reference genome (2**a**) and the 1942 SNPs identified (2**b**)

Allelic comparison with the 2070 genes annotated in the reference genome (WUE2594) identified 1542 (74.5%) identical genes among all isolates, 1347 (65.1%) of which possessed the same allele as WUE2594. A total of 196 (9.5%) genes were identical in the Chad isolates but different from the reference. Sixty-six (3.2%) genes present in WUE2594 were absent in the Chadian isolates, while 242 (11.7%) were present in all but variable among the 23 *Nm*A isolates from Chad. A total of 221 (10.7%) of these loci had incomplete sequences, as a consequence of incomplete assembly and were not included in pairwise comparisons. Neighbor-Net analysis using a distance matrix based on 1849 (89.3%) genes (excluding the 221 incomplete loci) resolved three distinct clusters: Cluster 1, comprising 8 isolates; Cluster 2, 13 isolates; and, Cluster 3, 2 isolates (Table 2 and Fig. 2a).

**Table 2:** Epidemiological data associated with the host of the isolates and bacterial clusters

ID	Epidemiological data of the host			Bacterial characteristic^a^		
PubMLST	Phenotype	Sex	Age (year/month)	ST	rST	Genomic clusters
34990	Disease	F	6	ST-7	rST-8263	2
34991	Disease	M	10	ST-7	rST-8296	1
34992	Disease	M	11	ST-7	rST-8303	1
34994	Disease	F	0/5	ST-9021	rST-8296	1
34995	Disease	M	-	ST-7	rST-8311	3
34996	Disease	M	15	ST-7	rST-8323	3
34997	Disease	M	15	ST-7	rST-8335	2
34998	Disease	-	-	ST-7	rST-8335	2
34999	Disease	-	-	ST-7	rST-8335	2
35000	Disease	M	11	ST-7	rST-8335	2
35001	Disease	M	18	ST-7	rST-8335	2
35002	Disease	M	16	ND	rST-8335	2
35003	Disease	-	-	ST-7	rST-8296	1
35004	Carriage	M	36	ST-7	rST-8263	2
35005	Carriage	M	14	ST-7	rST-8335	2
35006	Carriage	F	28	ST-7	rST-8263	2
35007	Carriage	F	4	ST-7	rST-8296	1
35008	Carriage	M	12	ST-7	rST-8296	1
35009	Carriage	M	7	ST-7	rST-8296	1
35010	Carriage	M	36	ST-7	rST-8263	2
35011	Carriage	F	22	ST-7	rST-8335	2
35012	Carriage	F	13	ST-7	rST-8263	2
35013	Carriage	F	6	ST-7	rST-8296	1

^a^All the isolates had the following strain designation: A: P1. 20,9: F3-1: ST: cc5; the variable STs are indicated in the table

### Differences among clusters

As cluster 3 comprised only two invasive isolates from the same geographical location it was not included in further statistical analysis. A significant difference in the mean age of the individuals from whom the *Nm*A were isolated was observed between Clusters 1 and 2, with Cluster 1 exhibiting a mean age of 7.2 years and Cluster 2 a mean age of 19.5 years, *p* = *0.007*. No significant difference between the clusters was found for the gender or residence of individuals from whom the meningococci were isolated. Cluster-specific allelic differences were identified in nine loci for Cluster 1 and 10 loci for Cluster 2. These allelic differences included non-synonymous mutations (NSMs) in seven genes. In Cluster 1, four of the NSMs led to changes in the chemical properties of the encoded amino acids (e.g. polar to non-polar; acidic to basic) and similarly, in Cluster 2 with six NSMs. In both clusters most of these genes were annotated as encoding components of metabolic pathways, however, this included enzymes, genes associated with antibiotic resistance, toxicity, and genetic information processing (Tables 3 and 4).

**Table 3:** Genes with alleles specific to Cluster 1 isolates

Locus	Functional category	Number of mutation	Number of non-synonymous mutation
NEIS2150 (*alx/TerC*)	Integral membrane protein (tellurite resistance protein)	1 (bp 676)	1 (A → S)^a^
NMAA_0255	Insertion element IS1016 transposase	1 (bp 28)	1 (T → A)^a^
NEIS0947 (*trpG*)	Amino acid metabolism (phenylalanine, tyrosine and tryptophan biosynthesis)	1 (bp 414)	0
NMAA_0798	Hypothetical protein	1 (bp 400)	1 (E → K)^a^
NEIS1071	Hypothetical protein	1 (bp 461)	1 (Y → C)
NEIS1383 (*aroD*)	Amino acid metabolism (phenylalanine, tyrosine and tryptophan biosynthesis)	1 (bp 418)	1 (V → M)
NEIS1605	Enzyme (TatD DNase family)	1 (bp 186)	0
NEIS1742 (*murG*)	Glycan metabolism/Vancomycin resistance	1 (bp 632)	1 (A → V)
NEIS2135	Nicotinate and nicotinamide metabolism	1 insertion (bp 155)	15 stop codon^a^

^a^Change in the chemistry properties of the AA side chains

**Table 4:** Genes with alleles specific to cluster 2 isolates

Locus	Functional category	Number of mutation	Number of non-synonymous mutation
NEIS0370 (*carA*)	Metabolism (pyrimidine and alanine, aspartate and glutamate)	1 (bp 1057)	1 (T → A)^a^
NEIS0422 (*metK*)	Metabolism (cysteine and methionine metabolism)	1 (bp 726)	0
NEIS0529	Metal ions transport (zinc/manganese)	1 (bp 363)	0
NEIS1091 (*cysl*)	Metabolism (sulfite reductase)	1 (bp 1021)	1 (P → T)^a^
NEIS1237 (*cmk*)	Metabolism (pyrimidine)	1 (bp 611)	1 (I → T)^a^
NEIS1379 (*dnaZX*)	Metabolism/DNA processing	1 (bp 1774)	1 (G → S)^a^
NEIS1460	Putative metabolism (Glucose-6-Phosphate 1-Dehydrogenase_bact motif)	1 (bp 402)	0
NEIS1741 (*murC*)	Metabolism (D-glutamine/D-Glutamate, peptidoglycan biosynthesis)	1 (bp 397)	1 (A → S)^a^
NEIS1804	putative role in toxicity (associated with Maf Genomic islands MGI-1 scheme pubmlst)	1 (bp 868)	1 (F → V)
NEIS0132 (*rplC*)	Genetic information processing (ribosomal protein)	1 (bp 22)	1 (C → R)^a^

^a^Change in the chemistry properties of the AA side chains

Within cluster comparisons identified four genes that included alleles distinct to the 4 carried isolates from Cluster 1 (Table 5); however, one of these genes, NMAA_0123, was found to be identical to that found in the two invasive isolates in Cluster 3. All of the other alleles were specific to the carried sub-cluster.

**Table 5:** Genes with alleles specific to the carried isolates of Cluster 1

Locus	Functional category	Number of mutation	Number of non-synonymous mutation
NMAA_0123	Hypothetical protein	Difference in G polymeric track length	Preliminary stop Codon^a^
NEIS1527	Two component response regulator	Insertion of “GCGTT”	Frameshift leading to a longer coding sequence^a^
NEIS2894	Hypothetical protein	1 (bp 65)	1 (T → I)^a^
NEIS0291 (lot)	LOS O-acetyltransferase	1 (bp 465)	0

^a^Change in the chemistry properties of the AA side chains

### Whole genome Single Nucleotide Polymorphism (wgSNP) analysis of non-coding regions

WgSNP comparison of the 23 *Nm*A genomes from Chad and the WUE2594 reference genome identified 1942 SNPs using assembled fasta sequences as input files, with 1924 SNPs identified when raw fastq files were used. Neighbor Net trees (Fig. 2b and Additional file 1: Figure S4) generated from the SNP matrix had a similar topology to those generated by wgMLST analysis (Fig. 2a).

Using the annotations from the finished genome, WUE2594, enabled coding-regions, which had been included in wgMLST analysis, to be distinguished from non-coding regions, as well as other regions not found in the reference genome. There were 182 SNPs (assembled fasta files) and 181 SNPs (original fastq files) identified in non-coding regions: the majority of them 109 (fasta) and 112 (fastq) discriminated between the reference genome and the 23 Chadian *Nm*A; 26 SNPs were specific to Cluster 3; 6 SNPs were specific to Cluster 2 and 16 were specific to Cluster 1 by both methods. Within those 16 SNPs, only 3 were specific to the whole Cluster 1; a total of nine SNPs grouped six isolates (PubMLST ID: 34992, 34994, 35007, 35008, 35009 and 35013), three SNPs were specific to two isolates (PubMLST ID: 34991 and 35003) and one SNP was specific to the four carried isolates (PubMLST ID: 35007, 35008, 35009 and 35013). The other SNPs were unique, with 13 specific to PubMLST ID: 35007 by both methods (Additional file 2: Table S4). The SNP specific to the four carried isolates sub-cluster was mapped to a non-coding region between NEIS1544 and NEIS1545; an alignment of that region from all isolates of Cluster 1 allowed the identification of a nucleotide change from a G to an A, 132 base pairs upstream of the start codon of NEIS1545.

## Discussion

In the absence of comprehensive vaccines, meningococcal meningitis epidemics remain a serious threat to public health in the African meningitis belt and elsewhere. Understanding the transition from the carried to the invasive phenotype will contribute to improved preventive measures in both epidemic and non-epidemic periods. The simultaneous collection of carried and invasive isolates during the 2011 epidemic in Chad enabled the comparison of closely related isolates obtained in the same temporal and geographic sampling frame. Such genomic comparisons rely on the availability of comprehensive collections of isolates, which are difficult to obtain and access from countries of the African meningitis belt. For example, isolate storage requires a −80 °C freezer with a reliable electricity supply. While all of the Chadian isolates were appropriately stored at −80 °C at the National Reference Laboratory of N’Djamena, the recovery rate was very low, perhaps a consequence of delays in the samples reaching the laboratory and/or difficulties in maintaining storage temperatures. The invasive isolates were mainly sourced from different areas around the capital N’Djamena, whilst the carried isolates were all obtained in the district of Mandelia, situated about 65 km away from N’Djamena (Additional file 1: Figure S2). The carriage study involved an age-stratified randomly selected proportion of the population of the study area and so were likely to be representative of those circulating in the community [18], whilst only a small proportion of disease cases were investigated microbiologically [14]; nevertheless, these samples offer comparisons of meningococci from disease and carriage in the same region at the same time.

All of the *Nm*A isolates belonged to the hyper-invasive, pandemic, ST-5 clonal complex (cc5), with rMLST analysis placing the Chadian epidemic *Nm*A in the context of 141 other publically available *Nm*A:cc5 isolates (Additional file 1: Table S2), dating from 1963 to 2011 and from five continents allowing a global representation of *Nm*A:cc5. This confirmed the close genetic relatedness of members of this complex [25], but the clustering within cc5 was not entirely congruent with time and place. For example, the Chadian isolates occupied two different branches: one including the majority of isolates (*n* = 21), comprised of 4 different rSTs; the other comprising the two remaining isolates with two different rSTs (Fig. 1). These isolates did not cluster with other meningococci from the African meningitis belt; however, a global distribution of cc5 sub-variants spanning several years was apparent with isolates from Bangladesh, USA, and South Africa clustering with Chadian meningococci and isolates from Niger or Burkina.

Chadian isolates were present on branches of the phylogeny that were distinct from those which included the ST-7 and ST-2859 *Nm*A isolates from Ghana and Burkina Faso, which have also been analyzed at the whole genome level [22], highlighting the need for the additional resolution obtained by rMLST in epidemiological studies [24]. The two isolates from Cluster 3 were more distantly related, as were the two Chinese isolates, indicating that this relatively small sample contained much of the diversity seen in publically available isolates from different locations. The previous study [22] noted potentially significant changes between ST-7 and ST-2859 *Nm*A isolates at: (i) the *pgl* locus, involved in glycosylation mechanisms; (ii) pilus regulation associated genes; and (iii) the *maf3* locus. In the Chad isolates, which were mostly ST-7, Cluster 1 and Cluster 2 shared the same alleles as the Ghanaian ST-7 isolates at the *pglD*, *pglC* and *pglB* locus: Cluster 3 had a different allele which may represent the acquisition of Deoxyribonucleic acid (DNA) from another source by homologous genetic transfer. The *pglH* locus was located at the beginning or the end of a contig in the majority of the draft genomes obtained and consequently its diversity could not be assessed in this analysis. The pilus genes were also variable in the Chad isolates but their variation did not correlate with the Clusters identified. On the other hand, all the Chadian isolates shared the same *maf3* alleles as the Ghanaian ST-7 isolates. Whole genome comparison of the *Nm*A:cc5 isolates clearly showed that the Chadian isolates were distinct at multiple loci from the Burkina Faso and Ghanaian isolates (Additional file 1: Figure S3); additional whole genome comparisons are required, however, to elucidate further differences between these two isolate collections.

This study is the first description of a meningococcus lacking the *gdh* gene, encoding glucose-6-phosphate 1-dehydrogenase, making it impossible to define its sequence type by seven-locus MLST and reiterating the usefulness of whole genome analysis. The deleted region also included loci encoding a 6-phosphogluconolactonase, a glucokinase, and *pgi1* (a glycose-6-phophate isomerase) which are involved in glucose metabolism. Glucose being an essential source of energy for *Nm* in blood and CSF [26], such an invasive isolate would be at a disadvantage during an infection. This deletion probably occurred during sub-cultivation and is unlikely to be of biological relevance.

Whole genome gene-by-gene analysis identified three different *Nm*A clusters circulating during the epidemic in Chad. Isolates were very similar, with 74.5% of the genes identical in all genomes and 242 (11.6%) genes confirmed as variable. No clustering by disease phenotype was evident, with Clusters 1 and 2 containing both disease and carried isolates in similar proportions, indicating that the invasive and carried isolates circulating during the epidemic were part of the same bacterial population. This was consistent with previous genomic comparisons among more diverse carried and disease isolates, which found no distinct monophyletic groups by gene content [4] or SNP analysis [22, 27].

Previously proposed “virulence-associated” genes were not systematically clustered on the basis of disease phenotype [28, 29] among these isolates. Within-cluster analysis identified two genes that had alterations specific to the four carried isolates of Cluster 1: NEIS1527 encodes a two-component response regulator member of the ActR/RegA family that is involved in signal transduction mechanism and transcription [30] and NEIS2894 encodes a hypothetical protein of unknown function. The impact of these genetic mutations in the biological function of these bacteria would need to be assessed further.

The use of wgSNP analysis to detect nucleotide changes in non-coding regions that discriminated carried and invasive isolates identified 9.4% of SNPs in non-coding regions (Additional file 2: Table S4). Only one SNP was found to discriminate carried from invasive isolates of Cluster 1, this SNP was found within the proximal promoter region of the gene NEIS1545, which is known to include transcription regulatory elements, but is located away from the −10 and −35 region which are part of the core promoter required for initiation of transcription [31]; additional analyses is necessary to determine the impact of this particular SNP on the transcription of NEIS1545. The wgSNP Neighbor Net trees (Fig. 2b and Additional file 1: Figure S4) showed identical phylogenetic clustering to that found with the wgMLST tree (Fig. 2a), with the same nodes; however, Cluster 1 appeared to be more diverse than observed with wgMLST analysis. SNPs found in the non-coding regions specific to sub-clusters and single isolates within Cluster 1 led to the observed diversity (28 SNPs in total, Additional file 2: Table S4). The wgSNP analysis based on the assembled fasta file and the original fastq files gave similar results.

Gene presence does not always correlate with expression and it is possible that despite both disease and carriage isolates possessing the same complement of genes, their expression levels might vary [32]. A study comparing two serogroup B *Nm* (*Nm*B) from different clonal complexes, a carried ST-41/44 isolate and an invasive ST-32 isolate, identified eight putative virulence-associated genes missing or non-functional from the carried isolate and considerable differences in their expression patterns [33]. *Nm* are highly variable bacteria and it is difficult to determine whether changes are due to the different phenotype or to inherent differences between the two clonal complexes. Analyses with additional genomes from the same clonal complexes are therefore essential for such comparisons. The results of the wgSNP analysis did not identify any changes at the nucleotide sequence level that could predict a change in gene expression between the carried and invasive isolates of this study except for one SNP found in the proximal promoter upstream of NEIS1545 that differentiated carried and invasive isolates of Cluster 1. A study applying similar methods to those presented here compared 172 carried and invasive *Nm*Y collected in the UK between 1997 and 2011 with an overlapping collection of both phenotypes obtained only in 2010. As found in this study, clusters of isolates contained both carried and invasive isolates; however, these investigators were able to identify a disease-associated clone within their clonal complex 23 *Nm*Y, which included 90% of invasive isolates [5].

The genetic differentiation between Clusters 1 and 2, and their strong association with host age, may represent bacterial adaptation to a particular niche and the change in the ecology of the nasopharynx from children to young adults in Chad. This is supported by the fact that most of the allelic variations found between the clusters were identified in metabolic genes (Tables 3 and 4). A previous study of the pharyngeal carriage of members of the *Neisseria* genus found that there was an inverse relationship between carriage of *Nm* and other non-pathogenic *Neisseria* species by age group which indicate a potential role that other microbes may play in modulating *Nm* carriage and could explain the age difference seen in this study [34]. Further studies, similar to those undertaken on the gut microbiota [35, 36], might address this issue. DNA microarray studies have identified a bacteriophage that was mostly found in genomes from the hyper-invasive clonal complexes [2]. This “meningococcal disease associated island” (MDA island) phage is associated with disease in young adults [3]. The MDA island genes were present in both Clusters 1 and 2 with the same alleles and thus could not explain the differentiation seen in this collection of isolates. Host genetic polymorphisms affecting the susceptibility of an individual to invasive meningococcal disease have been described, such as those in complement components such as factor H [37] or C6 deficiencies known to vary depending on the racial group and described as more common in African-American in the USA [38], interleukin-1 gene cluster [39] or the plasminogen activator inhibitor 1 [40]. Differences in host genetics could also explain our clusters; however, such studies have yet to be undertaken in African subjects.

## Conclusion

During the epidemic in and around N’Djamena, carriers and cases were infected by meningococci that were indistinguishable at the whole genome level, an observation which is consistent with host factors playing a major role in determining whether an infected person remains a carrier or becomes a case. Potential factors include the ability of the host immune response to contain the meningococcus in the pharynx or to eliminate it rapidly if invasion does occur, an ability which is at least partly genetically determined [37]. Gene expression among the isolates has not been directly compared in this work, and this may also play a role in the differentiation between carried and invasive isolates; one interesting SNP was identified in this study, upstream of a gene encoding a hypothetical protein, both the SNP and function of the gene should be investigated further to determine their role, if any, in meningococcal colonization and disease. In addition, it is possible that other bacteria within the pharyngeal microbiome influence the ability of a meningococcus to invade. Further bacterial genetic and protein expression studies, microbiome and human genetic studies comparing carriers and cases from similar backgrounds would help elucidate the role played by each factor in isolation and understand their interactions and the mechanisms driving *Nm* from a carried commensal to an invasive pathogen.

## Methods

### Isolate collection

A total of 33 *Nm*A carried isolates were collected during the second MenAfriCar carriage survey in Chad in 2011 and stored in BHI and 20% glycerol in a −80 °C freezer. These isolates were revived by inoculation on blood agar plates (BAP) and incubated at 37 °C with 5% CO_2_ for between 16 and 48 h. Ten viable isolates were recovered and were included in this dataset.

CSF samples were collected from patients with meningitis for laboratory confirmation as part of the national meningitis surveillance program. The majority of meningococci recovered from these specimens by culture methods were stored at the meningitis reference laboratory in N’Djamena, Chad and a small number of isolates were also stored at the WHO collaborating center for reference and research on Meningococci, based at the Norwegian Institute of public Health. A total of 13 invasive *NmA* were recovered and included in this analysis out of the 98 reported in the 2011 surveillance records [14].

Ethical approval for the MenAfriCar studies were obtained from the ethics committee of the London School of Hygiene & Tropical Medicine and ethical committees of each of the African partner institutions, with the exception of Chad, which did not have a formal ethical committee at the time of this study. Here, approval was granted by a committee that was established to oversee the MenAfriCar studies by the Chad Ministry of Health. Written informed consent or assent was obtained from subjects who participated in the pharyngeal carriage study. Invasive isolates from Chad were obtained from CSF samples collected from patients during the course of routine clinical care following a national protocol. The MenAfriCar studies were registered with ClinicalTrials.gov (NCT01119482).

### DNA extraction and sequencing

DNA was extracted in two different laboratories: the first batch included 19 isolates and DNA was extracted using the DNeasy® Blood and Tissue kit (Qiagen) as previously described [24]. Briefly, a sterile 1 μl loop was used to transfer bacterial growth into a tube containing 180 μl of buffer ATL and 20 μl of Proteinase K. The tube was then incubated at 56 °C for at least 2 h with intermittent mixing to lyse the cells. Following treatment with RNase A and subsequent addition of buffer AL/ethanol, the lysate was loaded on to the spinning column provided and the DNA was extracted and purified through a series of spinning and washing steps in accordance with the manufacturer’s instructions employing two elution steps each using 75 μL of buffer AE. The second batch included the remaining 4 isolates and DNA was extracted using an Eppendorf robot and the NucleoSpin 96 tissue kit (Macherey-Nagel) according to manufacturer’s instructions.

DNA samples were sequenced at the Oxford Genomics Centre. The library preparation was done using the Ultra DNA Sample Prep Kit (NEBNext) according to the manufacturer instructions and automated using a Biomek FX (Beckman Coulter). The genomic DNA (gDNA) was fragmented using the Episonic 2000 sonication system (Epigentek), then end-repaired, A-tailed and adapter-ligated using adapter designed “in house” [41], before the size selection, amplification and paired end sequencing performed on the Illumina HiSeq 2000 using a 100 base pair paired-end protocol as previously described [19].

### Genome annotation

Short-read sequences were assembled using the Velvet genome assembly program (v1.2.08) [42], after a performance comparison with the program Spades (v3.9.1) (Additional file 1: Table S3). All odd-numbered kmer lengths between 21 and 99 were sampled using the VelvetOptimiser software (v2.2.4) [43] to automatically calculate the optimal assembly parameters for Velvet (default optimization functions used). Assembled contigs were deposited into the *Neisseria* PubMLST database [21] which uses the Bacterial Isolate Genome Sequence Database (BIGSdb) software [44]. Draft genomes were first automatically curated at all loci defined in the database using the BLAST algorithm [45] and a sequence similarity threshold of >98%, allowing rapid annotation of known alleles and sequences which were very similar to defined loci [19]. Manual verification was then performed for variable loci, such as those containing internal stop codons, frame shifts or those with sequence similarities lower than 98%. Incomplete gene sequences at the beginning or end of a contig were identified as such and were excluded from further analyses.

### Hierarchical genomic analysis

Hierarchical gene-by-gene analysis was performed: conventional seven locus MLST [1], rMLST [46] and wgMLST [19] using the BIGSdb Genome Comparator tool [44] as previously described; briefly, the loci of interest: (i) 7 housekeeping genes of MLST [1]; (ii) 53 ribosomal genes of rMLST [46]; and (iii) 2070 genes annotated in the WUE2594 reference genome for wgMLST [47] were extracted from the 24 assembled genomes and a pairwise allelic comparison was performed using Genome Comparator with the following parameters: Min % identity of 70, Min % alignment of 50 and BLASTN word size of 15. A total of 141 publically available *Nm*A from the ST-5 clonal complex (*Nm*A:cc5), were obtained from PubMLST.org/neisseria [21] and included in the rMLST analysis to provide a global perspective to the epidemic using Neighbor-Joining trees with 50 bootstrap replications and the kimura 2-parameter model, generated with MEGA6 [48]. Only the unique strains were represented and they were defined as groups of isolates sharing the same alleles at all 53 ribosomal loci. The finished genome WUE2594 (GenBank accession number FR774048), an *Nm*A*:*cc5 invasive isolate [47], was used as a reference genome for the wgMLST analysis. Neighbor-Net trees were computed with SplitsTree (version 4.13.1) [49] using distance matrices generated by Genome Comparator after pairwise allelic comparisons of all 23 genomes at the loci of interest described above. Isolates with allelic variations specific to each of the clusters were identified from the variable gene lists generated by Genome Comparator. The nucleotide sequences of these genes were then extracted from the database for each genome and aligned in MEGA6 [48]. All nucleotide sequences and amino acid changes specific to a cluster were noted. The Artemis Comparison Tool [50] and MAUVE [51] were used to visualize, align and compare the organization of the loci on the contiguous sequence assemblies (contigs) obtained using default settings.

WgSNP analysis was performed using the program kSNP3 [52] using first the velvet assembled fasta files, then the raw fastq files of all 23 isolates alongside the finished reference genome WUE 2594. In order to use the fastq files, first these were downloaded from the European Nucleotide Archive (ENA) [53] using the ENA accession numbers available in the PubMLST isolate records and then processed with the tools from the FASTX-toolkit [54], using the command-line : the low quality reads were trimmed for each isolate data using “fastq_quality_trimmer” with a quality threshold of 28, then “fastq_to_fasta” was used on the trimmed data to obtain fasta files for each isolates (the –n option was used to keep all the sequences’ information available in the trimmed files). Once the fasta files were obtained, the built in option “MakeKSNP3infile” of kSNP3 was used to make the required input file, “MakeFasta” was used to transform that “infile” into a fasta format that was used to run “Kchooser” in order to determine the optimal kmer size (k) or the length of the sequence that kSNP3 will find in each isolate sequence data; the optimal k was 19 for the velvet assembled fasta files and 21 for the original fastq files. The values of k obtained were then used to run kSNP3 with the annotations from the reference genome WUE2594 manually downloaded from genbank and including the gi numbers (-genbank argument). Finally the output files were filtered out to remove all the SNPs found in a coding region. The Neighbor Net trees were computed using SplitsTree (version 4.13.1) [49]. Further SNPs characterization was done when necessary by visualizing the genome sequences in Artemis and aligning the sequences of interest in MEGA6 [48], using MUSCLE [55].

### Statistical analysis

The age, sex and geographical location of the meningococcal cases and carriers were recovered. A *t*-test was used to measure the statistical significance of the changes in age distribution observed between Cluster 1 and 2 identified, by comparing the mean age of the individuals from which the isolates from each cluster were collected. A *t*-test was also used to measure significant differences in the proportions of gender and provenances between the clusters by comparing the proportion of the gender group and the proportions of different provenance of the individuals from which the isolates came from between both clusters. T-tests were performed in Excel (version 2013).

## Additional files

Additional file 1: Supplemental Figures and Tables. Description of Data: Supplemental Figures and Tables referred to in the main manuscript and the data and discussion generated from the comparison between the velvet/VelvetOptimiser and Spades assembly methods. **Figure S1.** Deletion of six genes in isolate 120–2011. **Figure S2.** Geographical location of the *Nm*A isolates. **Figure S3.** wgMLST relationship of the *Nm*A:cc5 isolates. **Figure S4.** wgSNP relationship of the 23 Chadian *Nm*A from their original fastq files. **Table S1.** Velvet assembly statistics. **Table S2.** ID and characteristics of cc5 *Nm*A isolated in previous studies. **Table S3.** Velvet/VelvetOptimiser and Spades assembly statistics. (DOCX 699 kb)

Additional file 2: SNP analysis supplemental data. Description of Data: List of Cluster’s specifics SNPs and detailed list of all SNPs identified. (DOCX 390 kb)

## Abbreviations

Cc: Clonal complex
CSF: Cerebrospinal fluid
DNA: Deoxyribonucleic acid
ENA: European Nucleotide Archive
MDA island: Meningococcal disease associated island
MLST: Multi-Locus Sequence Typing
*Nm*: *Neisseria meningitidis*
NmA: Serogroup A *Neisseria meningitidis*
NmB: Serogroup B *Neisseria meningitidis*
NmC: Serogroup C *Neisseria meningitidis*
NmW: Serogroup W *Neisseria meningitidis*
NmX: Serogroup X *Neisseria meningitidis*
NmY: Serogroup Y *Neisseria meningitidis*
NSM: Non- synonymous mutations
rMLST: Ribosomal Multi-Locus Sequence Typing
ST: Sequence Type
wgMLST: Whole genome Multi-Locus Sequence Typing
wgNSP: Whole genome Single Nucleotide Polymorphism
WGS: Whole Genome Sequences
