# Supplementary material for: Hierarchical genomic analysis of carried and invasive serogroup A Neisseria meningitidis during the 2011 epidemic in Chad
Source: BMC Genomics. 2017 May 22;18:398. doi: 10.1186/s12864-017-3789-0 (PMC5441073; doi:10.1186/s12864-017-3789-0)
Supplement: Supplementary file 1 — Supplemental Figures and Tables. Description of Data: Supplemental Figures and Tables referred to in the main manuscript and the data and discussion generated from the comparison between the velvet/VelvetOptimiser and Spades assembly methods. Figure S1. Deletion of six genes in isolate 120–2011. Figure S2. Geographical location of the NmA isolates. Figure S3. wgMLST relationship of the NmA:cc5 isolates. Figure S4. wgSNP relationship of the 23 Chadian NmA from their original fastq files. Table S1. Velvet assembly statistics. Table S2. ID and characteristics of cc5 NmA isolated in previous studies. Table S3. Velvet/VelvetOptimiser and Spades assembly statistics. (DOCX 699 kb) [file 12864_2017_3789_MOESM1_ESM.docx]

**Additional file 1**


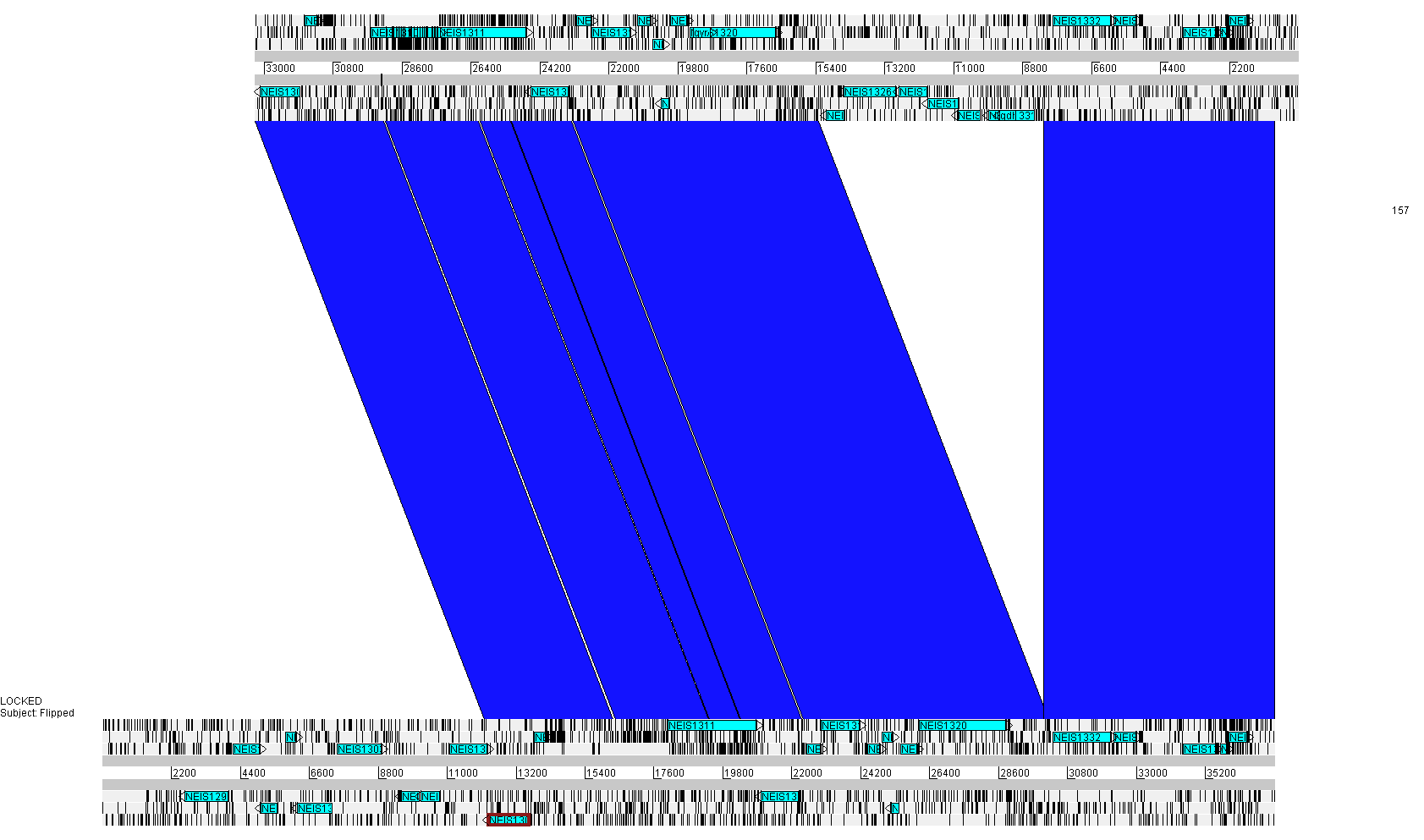


#### Figure S1. Deletion of six genes in isolate 120-2011

ACT comparison of isolates 10-2011 (on the top) and 120-2011 (at the bottom). The blue blocks represent identical sequences in reversed orientation and the red box highlights the deleted genetic sequence.

**Figure S2. Geographical location of the *Nm*A isolates**

A map of the African meningitis belt showing Chad in relation its neighboring countries; countries of the MenAfriCar project are shown in orange and the name of the countries sharing borders with Chad. The map was adapted from the MenAfriCar website, <http://www.menafricar.org> (A). The provenance location of the 2011 *Nm*A isolates included in this study on a map of Chad adapted from Daugla, MD et al. 2014 [1] . The black stars represent the disease isolates and the red ones the carried isolates (B).


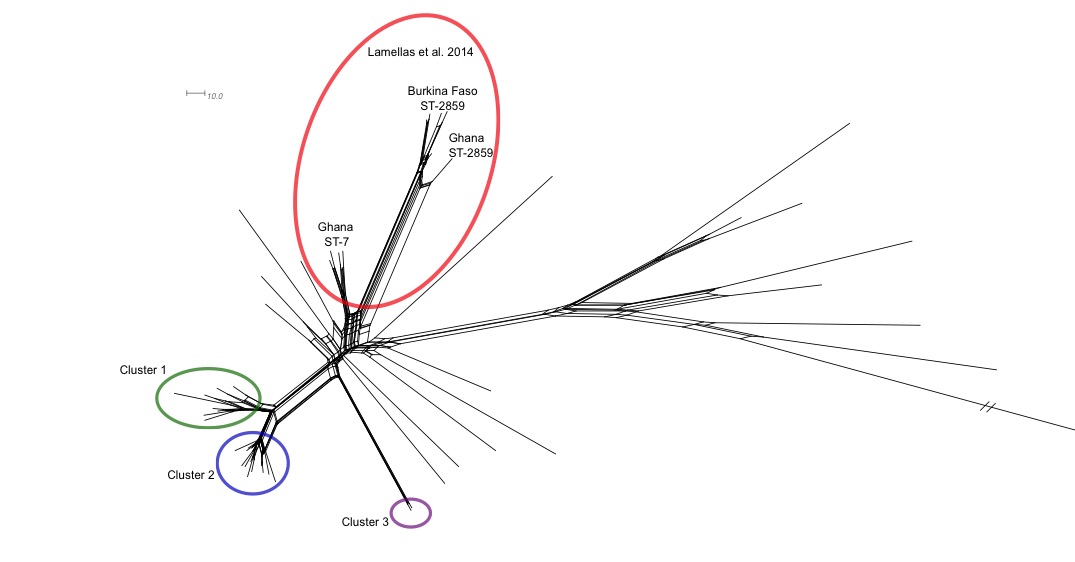


**Figure S3. wgMLST relationship of the *Nm*A:cc5 isolates**

Whole genome analysis of the 50 isolates included in the rMLST analysis (all the Chadian isolates and a single representative of unique strain of the other *Nm*A:cc5 publically available isolate) is presented in this neighbor Net tree. The three clusters identified in this study are labeled as well as the isolates from the most recent publication on genomic of *Nm*A:cc5 in the African meningitis belt in Africa

**Figure S4. wgSNP relationship of the 23 Chadian *Nm*A from their original fastq files**

Whole genome SNP analysis based on the 1924 SNPs identified in the kSNP3 analysis based on the processed fastq files is presented in this neighbor Net tree. The three clusters identified in this study are labeled. The same reference genome WUE 2504 was used in this analysis

#### Table S1. Velvet assembly statistics

| PubMLST ID | Isolate ID | Number of contigs | N50 | Allele designated (%)* | Total length bp |
| --- | --- | --- | --- | --- | --- |
| 34990 | Tchad54/11 | 125 | 51921 | 1574 (98.1) | 2160120 |
| 34991 | Tchad78/11 | 171 | 40301 | 1575 (98.1) | 2163900 |
| 34992 | Tchad95/11 | 175 | 37578 | 1574 (98.1) | 2159198 |
| 34994 | Tchad106/11 | 185 | 35661 | 1561 (97.3) | 2174025 |
| 34995 | 8-2011 | 130 | 43934 | 1573 (98) | 2161554 |
| 34996 | 10-2011 | 113 | 51921 | 1577 (98.3) | 2159817 |
| 34997 | 39-2011 | 162 | 41190 | 1560 (97.2) | 2166409 |
| 34998 | 59-2011 | 126 | 51915 | 1573 (98) | 2165858 |
| 34999 | 63-2011 | 125 | 52444 | 1573 (98) | 2165886 |
| 35000 | 75-2011 | 134 | 40895 | 1571 (97.9) | 2162502 |
| 35001 | 76-2011 | 148 | 48158 | 1575 (98.1) | 2163115 |
| 35002 | 120-2011 | 156 | 38996 | 1558 (97.1) | 2155465 |
| 35003 | 403-2011 | 243 | 28533 | 1539 (95.9) | 2173410 |
| 35004 | 12-15398-XS2-1 | 135 | 52853 | 1567 (97.6) | 2165054 |
| 35005 | 12-13952-XS2-1 | 193 | 29845 | 1564 (97.4) | 2163657 |
| 35006 | 12-15317-XS2-1 | 173 | 39334 | 1562 (97.3) | 2166457 |
| 35007 | 12-15047-XS2-1 | 167 | 41374 | 1572 (97.9) | 2166917 |
| 35008 | 12-15661-XS2-1 | 134 | 44112 | 1574 (98.1) | 2163800 |
| 35009 | 12-15657-XS2-1 | 164 | 41379 | 1567 (97.6) | 2165755 |
| 35010 | 12-17186-XS2-1 | 145 | 45919 | 1568 (97.7) | 2161231 |
| 35011 | 12-17194-XS2-1 | 132 | 48170 | 1570 (97.8) | 2164552 |
| 35012 | 12-17009-XS2-1 | 149 | 50196 | 1562 (97.3) | 2168433 |
| 35013 | 12-14973-XS2-1 | 160 | 41070 | 1571 (97.9) | 2162718 |

* Number of alleles designated from the 1605 core genome MLST (cgMLST) v1.0 loci [2]

**Table S2. ID and characteristics of cc5 *Nm*A isolated in previous studies.**

| pubMLST ID | Country | Year | Disease | rST | Strain designation | Reference |
| --- | --- | --- | --- | --- | --- | --- |
| 34598 | Algeria | 2000 | Invasive (unspecified/other) | 6971 | A: P1.20,9: F3-1: ST-7 (cc5) |  |
| 34654 | Bangladesh | 2003 | Invasive (unspecified/other) | 2434 | A: P1.20,9: F3-1: ST-4789 (cc5) |  |
| 34655 | Bangladesh | 2006 | Invasive (unspecified/other) | 3173 | A: P1.20,9: F-ND: ST-8428 (cc5) |  |
| 34572 | Brazil | 1975 | Invasive (unspecified/other) | 2434 | A: P1.20,9: F2-1: ST-5 (cc5) |  |
| 34573 | Brazil | 1975 | Invasive (unspecified/other) | ND | A: P1.20,9: F3-1: ST-5 (cc5) |  |
| 84 | Brazil | 1976 |  | 2434 | A: P1.20,9: F2-1: ST-5 (cc5) | Maiden, MC et al. 1998 |
| 34599 | Burkina Faso | 2001 | Invasive (unspecified/other) | 2434 | A: P1.20,9: F3-1: ST-7 (cc5) |  |
| 34808 | Burkina Faso | 2006 | Invasive (unspecified/other) | 2434 | A: P1.20,9: F3-1: ST-2859 (cc5) | Lamelas, A et al. 2014 |
| 34809 | Burkina Faso | 2006 | Carrier | 2434 | A: P1.20,9: F3-1: ST-2859 (cc5) | Lamelas, A et al. 2014 |
| 34810 | Burkina Faso | 2006 | Carrier | 2434 | A: P1.20,9: F3-1: ST-2859 (cc5) | Lamelas, A et al. 2014 |
| 34812 | Burkina Faso | 2006 | Carrier | 2434 | A: P1.20,9: F3-1: ST-2859 (cc5) | Lamelas, A et al. 2014 |
| 34817 | Burkina Faso | 2006 | Carrier | 2434 | A: P1.20,9: F3-1: ST-2859 (cc5) | Lamelas, A et al. 2014 |
| 34818 | Burkina Faso | 2006 | Invasive (unspecified/other) | 2434 | A: P1.20,9: F3-1: ST-2859 (cc5) | Lamelas, A et al. 2014 |
| 34820 | Burkina Faso | 2006 | Carrier | 2434 | A: P1.20,9: F3-1: ST-2859 (cc5) | Lamelas, A et al. 2014 |
| 34822 | Burkina Faso | 2006 | Invasive (unspecified/other) | 2434 | A: P1.20,9: F3-1: ST-2859 (cc5) | Lamelas, A et al. 2014 |
| 34824 | Burkina Faso | 2006 | Carrier | 2434 | A: P1.20,9: F3-1: ST-2859 (cc5) | Lamelas, A et al. 2014 |
| 34834 | Burkina Faso | 2006 | Carrier | 2434 | A: P1.20,9: F3-1: ST-2859 (cc5) | Lamelas, A et al. 2014 |
| 34838 | Burkina Faso | 2006 | Carrier | 2434 | A: P1.20,9: F3-1: ST-2859 (cc5) | Lamelas, A et al. 2014 |
| 34841 | Burkina Faso | 2006 | Carrier | 2434 | A: P1.20,9: F3-1: ST-2859 (cc5) | Lamelas, A et al. 2014 |
| 34842 | Burkina Faso | 2006 | Carrier | 2434 | A: P1.20,9: F3-1: ST-2859 (cc5) | Lamelas, A et al. 2014 |
| 34843 | Burkina Faso | 2006 | Invasive (unspecified/other) | 2434 | A: P1.20,9: F3-1: ST-2859 (cc5) | Lamelas, A et al. 2014 |
| 34847 | Burkina Faso | 2006 | Invasive (unspecified/other) | 2434 | A: P1.20,9: F3-1: ST-2859 (cc5) | Lamelas, A et al. 2014 |
| 34856 | Burkina Faso | 2006 | Carrier | 2434 | A: P1.20,9: F3-1: ST-2859 (cc5) | Lamelas, A et al. 2014 |
| 34863 | Burkina Faso | 2006 | Carrier | 2434 | A: P1.20,9: F3-1: ST-2859 (cc5) | Lamelas, A et al. 2014 |
| 34864 | Burkina Faso | 2006 | Carrier | 2434 | A: P1.20,9: F3-1: ST-2859 (cc5) | Lamelas, A et al. 2014 |
| 34866 | Burkina Faso | 2006 | Carrier | 3195 | A: P1.20,9: F3-1: ST-2859 (cc5) | Lamelas, A et al. 2014 |
| 34872 | Burkina Faso | 2006 | Carrier | 2434 | A: P1.20,9: F3-1: ST-2859 (cc5) | Lamelas, A et al. 2014 |
| 34876 | Burkina Faso | 2006 | Carrier | 3195 | A: P1.20,9: F3-1: ST-2859 (cc5) | Lamelas, A et al. 2014 |
| 34877 | Burkina Faso | 2006 | Invasive (unspecified/other) | 2434 | A: P1.20,9: F3-1: ST-2859 (cc5) | Lamelas, A et al. 2014 |
| 34883 | Burkina Faso | 2006 | Carrier | 2434 | A: P1.20,9: F3-1: ST-2859 (cc5) | Lamelas, A et al. 2014 |
| 34888 | Burkina Faso | 2006 | Invasive (unspecified/other) | 2434 | A: P1.20,9: F3-1: ST-2859 (cc5) | Lamelas, A et al. 2014 |
| 34890 | Burkina Faso | 2006 | Invasive (unspecified/other) | 2434 | A: P1.20,9: F3-1: ST-2859 (cc5) | Lamelas, A et al. 2014 |
| 34892 | Burkina Faso | 2006 | Invasive (unspecified/other) | 2434 | A: P1.20,9: F3-1: ST-2859 (cc5) | Lamelas, A et al. 2014 |
| 34893 | Burkina Faso | 2006 | Carrier | 2434 | A: P1.20,9: F3-1: ST-2859 (cc5) | Lamelas, A et al. 2014 |
| 34894 | Burkina Faso | 2006 | Invasive (unspecified/other) | 2434 | A: P1.20,9: F3-1: ST-2859 (cc5) | Lamelas, A et al. 2014 |
| 34610 | Burkina Faso | 2007 | Invasive (unspecified/other) | 2434 | A: P1.20,9: F3-1: ST-2859 (cc5) |  |
| 34811 | Burkina Faso | 2007 | Invasive (unspecified/other) | 2434 | A: P1.20,9: F3-1: ST-2859 (cc5) | Lamelas, A et al. 2014 |
| 34819 | Burkina Faso | 2007 | Carrier | 2434 | A: P1.20,9: F3-1: ST-2859 (cc5) | Lamelas, A et al. 2014 |
| 34835 | Burkina Faso | 2007 | Carrier | 2434 | A: P1.20,9: F3-1: ST-2859 (cc5) | Lamelas, A et al. 2014 |
| 34854 | Burkina Faso | 2007 | Invasive (unspecified/other) | 2434 | A: P1.20,9: F3-1: ST-2859 (cc5) | Lamelas, A et al. 2014 |
| 34813 | Burkina Faso | 2008 | Carrier | 3068 | A: P1.20,9: F3-1: ST-2859 (cc5) | Lamelas, A et al. 2014 |
| 34882 | Burkina Faso | 2008 | Carrier | 3068 | A: P1.20,9: F3-1: ST-2859 (cc5) | Lamelas, A et al. 2014 |
| 128 | Chad | 1988 | Invasive (unspecified/other) | 2434 | A: P1.20,9: F3-1: ST-5 (cc5) | Maiden et al. 1998 |
| 34578 | Chad | 1988 | Invasive (unspecified/other) | 2434 | A: P1.20,9: F3-1: ST-5 (cc5) |  |
| 299 | China | 1963 | Carrier | 2511 | A: P1.5-2,10: F1-5: ST-5 (cc5) | Maiden et al. 1998 |
| 238 | China | 1966 | Invasive (unspecified/other) | 2468 | A: P1.20,9: F3-1: ST-5 (cc5) | Maiden et al. 1998 |
| 239 | China | 1966 | Invasive (unspecified/other) | 2503 | A: P1.20,9: F3-1: ST-6 (cc5) | Maiden et al. 1998 |
| 82 | China | 1984 | Invasive (unspecified/other) | 2434 | A: P1.20,9: F3-8: ST-5 (cc5) | Maiden et al. 1998 |
| 597 | China | 1992 | Invasive (unspecified/other) | 2501 | A: P1.20,9: F3-1: ST-7 (cc5) | Maiden et al. 1998 |
| 30466 | China | 2006 | Meningitis | 2434 | A: P1.20,ND: F3-1: ST-7 (cc5) | Zhang, Y et al. 2014 |
| 24 | Denmark | 1974 | Invasive (unspecified/other) | 2434 | A: P1.5-1,9: F3-1: ST-5 (cc5) | Maiden et al. 1998 |
| 7 | Finland | 1975 | Invasive (unspecified/other) | 2434 | A: P1.20,9: F3-1: ST-5 (cc5) | Maiden et al. 1998 |
| 31215 | France | 2006 | Meningitis and septicemia | 2434 | A: P1.20,9: F3-1: ST-4789 (cc5) |  |
| 19260 | Germany | 1991 | Meningitis | 2434 | A: P1.20,9: F1-21: ST-5 (cc5) | Schoen, C et al. 2011 |
| 34836 | Ghana | 2001 | Invasive (unspecified/other) | 2434 | A: P1.20,9: F3-1: ST-7 (cc5) | Lamelas, A et al. 2014 |
| 34829 | Ghana | 2002 | Carrier | 2434 | A: P1.20,9: F3-1: ST-7 (cc5) | Lamelas, A et al. 2014 |
| 34840 | Ghana | 2002 | Invasive (unspecified/other) | 2434 | A: P1.20,9: F3-1: ST-7 (cc5) | Lamelas, A et al. 2014 |
| 34844 | Ghana | 2002 | Invasive (unspecified/other) | 2434 | A: P1.20,9: F3-1: ST-7 (cc5) | Lamelas, A et al. 2014 |
| 34859 | Ghana | 2002 | Invasive (unspecified/other) | 2434 | A: P1.20,9: F3-1: ST-7 (cc5) | Lamelas, A et al. 2014 |
| 34861 | Ghana | 2002 | Carrier | 2434 | A: P1.20,9: F3-1: ST-7 (cc5) | Lamelas, A et al. 2014 |
| 34869 | Ghana | 2002 | Invasive (unspecified/other) | 2434 | A: P1.20,9: F3-1: ST-7 (cc5) | Lamelas, A et al. 2014 |
| 34875 | Ghana | 2002 | Invasive (unspecified/other) | 2434 | A: P1.20,9: F3-1: ST-7 (cc5) | Lamelas, A et al. 2014 |
| 34880 | Ghana | 2002 | Invasive (unspecified/other) | 2434 | A: P1.20,9: F3-1: ST-7 (cc5) | Lamelas, A et al. 2014 |
| 34900 | Ghana | 2002 | Invasive (unspecified/other) | 2434 | A: P1.20,9: F3-1: ST-7 (cc5) | Lamelas, A et al. 2014 |
| 34902 | Ghana | 2002 | Invasive (unspecified/other) | 2434 | A: P1.20,9: F3-1: ST-7 (cc5) | Lamelas, A et al. 2014 |
| 34827 | Ghana | 2003 | Carrier | 2434 | A: P1.20,9: F3-1: ST-7 (cc5) | Lamelas, A et al. 2014 |
| 34850 | Ghana | 2003 | Invasive (unspecified/other) | 2434 | A: P1.20,9: F3-1: ST-7 (cc5) | Lamelas, A et al. 2014 |
| 34852 | Ghana | 2003 | Invasive (unspecified/other) | 2434 | A: P1.20,9: F3-1: ST-7 (cc5) | Lamelas, A et al. 2014 |
| 34855 | Ghana | 2003 | Invasive (unspecified/other) | 2434 | A: P1.20,9: F3-1: ST-7 (cc5) | Lamelas, A et al. 2014 |
| 34858 | Ghana | 2003 | Carrier | 2434 | A: P1.20,9: F3-1: ST-7 (cc5) | Lamelas, A et al. 2014 |
| 34860 | Ghana | 2003 | Carrier | 2434 | A: P1.20,9: F3-1: ST-7 (cc5) | Lamelas, A et al. 2014 |
| 34871 | Ghana | 2003 | Invasive (unspecified/other) | 2434 | A: P1.20,9: F3-1: ST-7 (cc5) | Lamelas, A et al. 2014 |
| 34878 | Ghana | 2003 | Carrier | 2434 | A: P1.20,9: F3-1: ST-7 (cc5) | Lamelas, A et al. 2014 |
| 34881 | Ghana | 2003 | Carrier | 2434 | A: P1.20,9: F3-1: ST-7 (cc5) | Lamelas, A et al. 2014 |
| 34884 | Ghana | 2003 | Carrier | 2434 | A: P1.20,9: F3-1: ST-7 (cc5) | Lamelas, A et al. 2014 |
| 34887 | Ghana | 2003 | Invasive (unspecified/other) | 2434 | A: P1.20,9: F3-1: ST-7 (cc5) | Lamelas, A et al. 2014 |
| 34825 | Ghana | 2004 | Invasive (unspecified/other) | 2434 | A: P1.20,9: F3-1: ST-7 (cc5) | Lamelas, A et al. 2014 |
| 34826 | Ghana | 2004 | Carrier | 2434 | A: P1.20,9: F3-1: ST-7 (cc5) | Lamelas, A et al. 2014 |
| 34828 | Ghana | 2004 | Carrier | 2434 | A: P1.20,9: F3-1: ST-7 (cc5) | Lamelas, A et al. 2014 |
| 34837 | Ghana | 2004 | Invasive (unspecified/other) | 2974 | A: P1.20,9: F3-1: ST-7 (cc5) | Lamelas, A et al. 2014 |
| 34845 | Ghana | 2004 | Invasive (unspecified/other) | 2434 | A: P1.20,9: F3-1: ST-7 (cc5) | Lamelas, A et al. 2014 |
| 34848 | Ghana | 2004 | Invasive (unspecified/other) | 2434 | A: P1.20,9: F3-1: ST-7 (cc5) | Lamelas, A et al. 2014 |
| 34849 | Ghana | 2004 | Carrier | 2974 | A: P1.20,9: F3-1: ST-7 (cc5) | Lamelas, A et al. 2014 |
| 34853 | Ghana | 2004 | Invasive (unspecified/other) | 2974 | A: P1.20,9: F3-1: ST-7 (cc5) | Lamelas, A et al. 2014 |
| 34862 | Ghana | 2004 | Carrier | 2974 | A: P1.20,9: F3-1: ST-7 (cc5) | Lamelas, A et al. 2014 |
| 34865 | Ghana | 2004 | Invasive (unspecified/other) | 2434 | A: P1.20,9: F3-1: ST-7 (cc5) | Lamelas, A et al. 2014 |
| 34867 | Ghana | 2004 | Carrier | 2974 | A: P1.20,9: F3-1: ST-7 (cc5) | Lamelas, A et al. 2014 |
| 34868 | Ghana | 2004 | Invasive (unspecified/other) | 2974 | A: P1.20,9: F3-1: ST-7 (cc5) | Lamelas, A et al. 2014 |
| 34873 | Ghana | 2004 | Carrier | 2434 | A: P1.20,9: F3-1: ST-7 (cc5) | Lamelas, A et al. 2014 |
| 34879 | Ghana | 2004 | Carrier | 2434 | A: P1.20,9: F3-1: ST-7 (cc5) | Lamelas, A et al. 2014 |
| 34886 | Ghana | 2004 | Invasive (unspecified/other) | 2434 | A: P1.20,9: F3-1: ST-7 (cc5) | Lamelas, A et al. 2014 |
| 34891 | Ghana | 2004 | Carrier | 2434 | A: P1.20,9: F3-1: ST-7 (cc5) | Lamelas, A et al. 2014 |
| 34895 | Ghana | 2004 | Invasive (unspecified/other) | 2434 | A: P1.20,9: F3-1: ST-7 (cc5) | Lamelas, A et al. 2014 |
| 34896 | Ghana | 2004 | Carrier | 2434 | A: P1.20,9: F3-1: ST-7 (cc5) | Lamelas, A et al. 2014 |
| 34898 | Ghana | 2004 | Carrier | 2434 | A: P1.20,9: F3-1: ST-7 (cc5) | Lamelas, A et al. 2014 |
| 34899 | Ghana | 2004 | Invasive (unspecified/other) | 2434 | A: P1.20,9: F3-1: ST-7 (cc5) | Lamelas, A et al. 2014 |
| 34901 | Ghana | 2004 | Carrier | 2974 | A: P1.20,9: F3-1: ST-7 (cc5) | Lamelas, A et al. 2014 |
| 35095 | Ghana | 2004 | Carrier | 2434 | A: P1.20,9: F3-1: ST-7 (cc5) | Lamelas, A et al. 2014 |
| 34870 | Ghana | 2005 | Carrier | 2434 | A: P1.20,9: F3-1: ST-7 (cc5) | Lamelas, A et al. 2014 |
| 34874 | Ghana | 2005 | Invasive (unspecified/other) | 3025 | A: P1.20,9: F3-1: ST-7 (cc5) | Lamelas, A et al. 2014 |
| 34885 | Ghana | 2005 | Carrier | 3025 | A: P1.20,9: F3-1: ST-7 (cc5) | Lamelas, A et al. 2014 |
| 34889 | Ghana | 2005 | Invasive (unspecified/other) | 2434 | A: P1.20,9: F3-1: ST-7 (cc5) | Lamelas, A et al. 2014 |
| 34897 | Ghana | 2005 | Carrier | 2434 | A: P1.20,9: F3-1: ST-7 (cc5) | Lamelas, A et al. 2014 |
| 34830 | Ghana | 2007 | Carrier | 2434 | A: P1.20,9: F3-1: ST-2859 (cc5) | Lamelas, A et al. 2014 |
| 34805 | Ghana | 2008 | Carrier | 2434 | A: P1.20,9: F3-1: ST-2859 (cc5) | Lamelas, A et al. 2014 |
| 34814 | Ghana | 2008 | Carrier | 2434 | A: P1.20,9: F3-1: ST-2859 (cc5) | Lamelas, A et al. 2014 |
| 34815 | Ghana | 2008 | Carrier | 2434 | A: P1.20,9: F3-1: ST-2859 (cc5) | Lamelas, A et al. 2014 |
| 34816 | Ghana | 2008 | Carrier | 2434 | A: P1.20,9: F3-1: ST-2859 (cc5) | Lamelas, A et al. 2014 |
| 34821 | Ghana | 2008 | Carrier | 2434 | A: P1.20,9: F3-1: ST-2859 (cc5) | Lamelas, A et al. 2014 |
| 34823 | Ghana | 2008 | Carrier | 2434 | A: P1.20,9: F3-1: ST-2859 (cc5) | Lamelas, A et al. 2014 |
| 34831 | Ghana | 2008 | Carrier | 2434 | A: P1.20,9: F3-1: ST-2859 (cc5) | Lamelas, A et al. 2014 |
| 34832 | Ghana | 2008 | Carrier | 2434 | A: P1.20,9: F3-1: ST-2859 (cc5) | Lamelas, A et al. 2014 |
| 34833 | Ghana | 2008 | Carrier | 2434 | A: P1.20,9: F3-1: ST-2859 (cc5) | Lamelas, A et al. 2014 |
| 34839 | Ghana | 2008 | Carrier | 2434 | A: P1.20,9: F3-1: ST-2859 (cc5) | Lamelas, A et al. 2014 |
| 34846 | Ghana | 2008 | Carrier | 2434 | A: P1.20,9: F3-1: ST-2859 (cc5) | Lamelas, A et al. 2014 |
| 34851 | Ghana | 2008 | Carrier | 2434 | A: P1.20,9: F3-1: ST-2859 (cc5) | Lamelas, A et al. 2014 |
| 35094 | Ghana | 2008 | Carrier | 2434 | A: P1.20,9: F3-1: ST-2859 (cc5) | Lamelas, A et al. 2014 |
| 34806 | Ghana | 2009 | Carrier | 2434 | A: P1.20,9: F3-1: ST-2859 (cc5) | Lamelas, A et al. 2014 |
| 34807 | Ghana | 2009 | Carrier | ND | A: P1.20,9: F3-1: ST-2859 (cc5) | Lamelas, A et al. 2014 |
| 34589 | Mali | 1997 | Invasive (unspecified/other) | 2434 | A: P1.20,9: F3-1: ST-580 (cc5) |  |
| 34590 | Mali | 1997 | Invasive (unspecified/other) | 2434 | A: P1.20,9: F3-1: ST-580 (cc5) |  |
| 34583 | Niger | 1996 | Invasive (unspecified/other) | 2434 | A: P1.20,9: F3-1: ST-5 (cc5) |  |
| 34584 | Niger | 1996 | Invasive (unspecified/other) | 3016 | A: P1.20,9: F3-1: ST-5 (cc5) |  |
| 34587 | Niger | 1997 | Invasive (unspecified/other) | 2434 | A: P1.20,9: F3-1: ST-5 (cc5) |  |
| 34594 | Niger | 1998 | Invasive (unspecified/other) | 2434 | A: P1.20,9: F3-1: ST-5 (cc5) |  |
| 34597 | Niger | 2000 | Invasive (unspecified/other) | 6967 | A: P1.20,9: F3-1: ST-7 (cc5) |  |
| 34600 | Niger | 2002 | Invasive (unspecified/other) | ND | A: P1.20,9: F3-1: ST-7 (cc5) |  |
| 34604 | Niger | 2003 | Invasive (unspecified/other) | 2434 | A: P1.20,9: F3-1: ST-7 (cc5) |  |
| 34606 | Niger | 2004 | Invasive (unspecified/other) | 2434 | A: P1.20,9: F3-1: ST-7 (cc5) |  |
| 34607 | Niger | 2004 | Invasive (unspecified/other) | 3028 | A: P1.20,9: F3-1: ST-7 (cc5) |  |
| 451 | Russia | 1970 | Invasive (unspecified/other) | 2434 | A: P1.20,9: F3-1: ST-5 (cc5) | Maiden et al. 1998 |
| 34857 | Russia |  |  | 2434 | A: P1.20,9: F3-1: ST-7 (cc5) |  |
| 120 | Saudi Arabia | 1987 | Carrier | 2434 | A: P1.20,9: F3-1: ST-5 (cc5) | Maiden et al. 1998 |
| 29291 | South Africa | 2004 | Meningitis | 34706 | A: P1.20,9: F5-70: ST-7 (cc5) |  |
| 29434 | South Africa | 2005 | Meningitis | 2434 | A: P1.20,9: F3-1: ST-7 (cc5) |  |
| 29304 | South Africa | 2010 | Meningitis | 2434 | A: P1.20,9: F3-1: ST-7 (cc5) |  |
| 210 | UK | 1987 | Invasive (unspecified/other) | 2434 | A: P1.20,9: F3-1: ST-5 (cc5) | Maiden et al. 1998 |
| 30269 | UK | 1999 | Invasive (unspecified/other) | 2434 | A: P1.20,9: F3-1: ST-5 (cc5) |  |
| 20338 | UK | 2011 | Invasive (unspecified/other) | 2434 | A: P1.20,9: F3-1: ST-4789 (cc5) | Hill, DM et al. 2015 |
| 34663 | USA | 2000 | Invasive (unspecified/other) | ND | A: P1.20-1,9: F3-1: ST-7 (cc5) |  |
| 34665 | USA | 2001 | Invasive (unspecified/other) | 2985 | A: P1.20,9: F3-1: ST-7 (cc5) |  |

**Comparison between Velvet/VelvetOptimiser and Spades assemblies**

#### Table S3. Velvet/VelvetOptimiser and Spades assembly statistics

|  | **Velvet** | **Spades** | **Velvet** | **Spades** | **Velvet** | **Spades** | **Velvet** | **Spades** |
| --- | --- | --- | --- | --- | --- | --- | --- | --- |
| **Isolate ID** | **Number of Contigs** | | **Genome length** | | **N50** | | **Number of alleles designated (cgMLST v1.0 1605 loci) (%)** | |
| Tchad54/11 | 125 | 222 | 2160120 | 2152402 | 51921 | 36616 | 1574 (98.1) | 1590 (99.1) |
| Tchad78/11 | 171 | 275 | 2163900 | 2174251 | 40301 | 35212 | 1575 (98.1) | 1584 (98.7) |
| Tchad95/11 | 175 | 417 | 2159198 | 2233641 | 37578 | 33960 | 1574 (98.1) | 1588 (98.9) |
| Tchad106/11* | 185 | 3054 | 2174025 | 3362286 | 35661 | 1708 | 1561 (97.3) | 948 (59.1) |
| 8-2011 | 130 | 202 | 2161554 | 2155485 | 43934 | 32587 | 1573 (98) | 1588 (98.9) |
| 10-2011 | 113 | 216 | 2159817 | 2154902 | 51921 | 34753 | 1577 (98.3) | 1586 (98.8) |
| 39-2011 | 162 | 187 | 2166409 | 2146453 | 41190 | 33684 | 1560 (97.2) | 1589 (99) |
| 59-2011 | 126 | 212 | 2165858 | 2154741 | 51915 | 36616 | 1573 (98) | 1587 (98.9) |
| 63-2011 | 125 | 192 | 2165886 | 2149039 | 52444 | 34041 | 1573 (98) | 1588 (98.9) |
| 75-2011 | 134 | 263 | 2162502 | 2170834 | 40895 | 37875 | 1571 (97.9) | 1589 (99) |
| 76-2011 | 148 | 198 | 2163115 | 2149691 | 48158 | 33410 | 1575 (98.1) | 1587 (98.9) |
| 120-2011 | 156 | 225 | 2155465 | 2153417 | 38996 | 35995 | 1558 (97.1) | 1584 (98.7) |
| 403-2011 | 243 | 222 | 2173410 | 2160712 | 28533 | 40345 | 1539 (95.9) | 1587 (98.9) |
| 12-15398-XS2-1 | 135 | 195 | 2165054 | 2149651 | 52853 | 34752 | 1567 (97.6) | 1589 (99) |
| 12-13952-XS2-1 | 193 | 224 | 2163657 | 2152579 | 29845 | 35305 | 1564 (97.4) | 1585 (98.8) |
| 12-15317-XS2-1 | 173 | 262 | 2166457 | 2168661 | 39334 | 36616 | 1562 (97.3) | 1588 (98.9) |
| 12-15047-XS2-1 | 167 | 211 | 2166917 | 2153162 | 41374 | 34753 | 1572 (97.9) | 1589 (99) |
| 12-15661-XS2-1 | 134 | 261 | 2163800 | 2164291 | 44112 | 32587 | 1574 (98.1) | 1588 (98.9) |
| 12-15657-XS2-1 | 164 | 196 | 2165755 | 2152400 | 41379 | 32587 | 1567 (97.6) | 1586 (98.8) |
| 12-17186-XS2-1 | 145 | 197 | 2161231 | 2142409 | 45919 | 33410 | 1568 (97.7) | 1587 (98.9) |
| 12-17194-XS2-1 | 132 | 199 | 2164552 | 2152840 | 48170 | 34753 | 1570 (97.8) | 1587 (98.9) |
| 12-17009-XS2-1 | 149 | 227 | 2168433 | 2160729 | 50196 | 35212 | 1562 (97.3) | 1591 (99.1) |
| 12-14973-XS2-1 | 160 | 229 | 2162718 | 2160948 | 41070 | 40209 | 1571 (97.9) | 1584 (98.7) |
| **Average (excl. Tchad106/11)** | **152.7** | **228.7** | **2163900** | **2159693** | **43729** | **35240** | **1568.1 (97.7)** | **1587.3 (98.9)** |

***Method, Results and Discussion***

For comparison, the 23 isolates were assembled using Spades (v3.9.1) using the read error correction option (‘--careful’) and k-mers sampled from 21bp to 99bp.

Table S3 shows the assembly statistics for the Velvet/VelvetOptimiser and Spades assembly methods for all 23 genomes. Both methods were set for a minimum contig size of 200bp. One genome (Tchad106/11) did not assemble well by Spades, resulting in more than 3,000 contigs and was excluded from the calculation of averages. In general, Velvet assembled genomes with a smaller number of contigs (average of 152.7 for Velvet compared with 228.7 for Spades), and a larger N50 contig size (average of 43,729 for Velvet compared with 35,240 for Spades). To measure the number of complete loci that were recovered by each assembly, the number of allele designations was calculated for the core genome MLST *N. meningitidis* scheme (v1.0) containing 1605 loci generally found in at least 95% of meningococci [2]. Both Velvet/VelvetOptimiser and Spades methods consistently assembled more than 97% of the cgMLST loci to give known allelic variants, with Velvet averaging 97.7% and Spades averaging 98.9%. These values indicate that both methods are producing high quality genome assemblies.

Closer inspection of the allele designations for the 7 locus MLST genes (*abcZ, adk, aroE, fumC, gdh, pdhC, pgm)*, identified a different *fumC* allele in nine of the 23 Spades assemblies compared with the corresponding Velvet assembly. For example, isolate 8-2011 had *fumC* allele 1 in the Velvet assembly (resulting in ST-7) and *fumC* allele 843 in the Spades assembly. There were two bases different between these two alleles: T262 and C273 in allele 1 are G262 and T273 in allele 843. For both positions Spades was calling the minority base and was therefore less accurate in this case. In order to enable comparisons with previously published studies stored in the PubMLST Neisseria database, we chose to continue our analysis using Velvet/VelvetOptimiser assemblies.

***Reference***

1. Daugla DM, Gami JP, Gamougam K, Naibei N, Mbainadji L, Narbe M, Toralta J, Kodbesse B, Ngadoua C, Coldiron ME, et al: **Effect of a serogroup A meningococcal conjugate vaccine (PsA-TT) on serogroup A meningococcal meningitis and carriage in Chad: a community study [corrected].** *Lancet* 2014, **383:**40-47.

2. Bratcher HB, Corton C, Jolley KA, Parkhill J, Maiden MC: **A gene-by-gene population genomics platform: de novo assembly, annotation and genealogical analysis of 108 representative Neisseria meningitidis genomes.** *BMC Genomics* 2014, **15:**1138.
