# Supplementary material for: Hierarchical genomic analysis of carried and invasive serogroup A Neisseria meningitidis during the 2011 epidemic in Chad
Source: BMC Genomics. 2017 May 22;18:398. doi: 10.1186/s12864-017-3789-0 (PMC5441073; doi:10.1186/s12864-017-3789-0)
Supplement: Supplementary file 2 — SNP analysis supplemental data. Description of Data: List of Cluster’s specifics SNPs and detailed list of all SNPs identified. (DOCX 390 kb) [file 12864_2017_3789_MOESM2_ESM.docx]

|  | Cluster 1 | | | | | Cluster 2 | Cluster 3 |
| --- | --- | --- | --- | --- | --- | --- | --- |
|  | All Cluster 1 | Carried isolates of Cluster 1 | Carried isolates + 34992 and 34994 | 34991 and 35003 | 35007 |  |  |
| SNP LocusNum in Table S4 | 1500; 1667; 1895 | 1052 | 58; 178; 265; 463; 686; 1468; 1705; 1809;  1889 | 0;  719;  1382 | 26; 551;  695;  793;  828;  986;  1056;  1142;  1491;  1521;  1680;  1841;  1844 | 156;  572;  644; 914;  1790; 1870 | 47; 118; 174; 182; 291; 315; 320; 333; 404; 438; 522; 687; 769; 813; 833; 876; 878; 1161; 1239; 1385; 1430; 1431; 1504; 1556; 1765; 1793 |

**Table S4. SNPs in the non coding region or not found in the reference**

**genome (WUE 2594)**

| **LocusNum** | **Context** | **Allele** | **Position Strand** | **ID** | **AnnotationType** |
| --- | --- | --- | --- | --- | --- |
| 0 | AAAAAAAAAA.AAACTTACCT | T | 105910 R | 19260_WUE_2594 | UnannotatedRegion |
| 0 | AAAAAAAAAA.AAACTTACCT | T | x | 34990_Tchad54_11 | UnannotatedRegion |
| 0 | AAAAAAAAAA.AAACTTACCT | A | x | 34991_Tchad78_11 | UnannotatedRegion |
| 0 | AAAAAAAAAA.AAACTTACCT | T | x | 34992_Tchad95_11 | UnannotatedRegion |
| 0 | AAAAAAAAAA.AAACTTACCT | T | x | 34994_Tchad106_11 | UnannotatedRegion |
| 0 | AAAAAAAAAA.AAACTTACCT | T | x | 34995_8_2011 | UnannotatedRegion |
| 0 | AAAAAAAAAA.AAACTTACCT | T | x | 34996_10_2011 | UnannotatedRegion |
| 0 | AAAAAAAAAA.AAACTTACCT | T | x | 34997_39_2011 | UnannotatedRegion |
| 0 | AAAAAAAAAA.AAACTTACCT | T | x | 34998_59_2011 | UnannotatedRegion |
| 0 | AAAAAAAAAA.AAACTTACCT | T | x | 34999_63_2011 | UnannotatedRegion |
| 0 | AAAAAAAAAA.AAACTTACCT | T | x | 35000_75_2011 | UnannotatedRegion |
| 0 | AAAAAAAAAA.AAACTTACCT | T | x | 35001_76_2011 | UnannotatedRegion |
| 0 | AAAAAAAAAA.AAACTTACCT | T | x | 35002_120_2011 | UnannotatedRegion |
| 0 | AAAAAAAAAA.AAACTTACCT | A | x | 35003_403_2011 | UnannotatedRegion |
| 0 | AAAAAAAAAA.AAACTTACCT | T | x | 35004_12_15398_XS2_1 | UnannotatedRegion |
| 0 | AAAAAAAAAA.AAACTTACCT | T | x | 35005_12_13952_XS2_1 | UnannotatedRegion |
| 0 | AAAAAAAAAA.AAACTTACCT | T | x | 35006_12_15317_XS2_1 | UnannotatedRegion |
| 0 | AAAAAAAAAA.AAACTTACCT | T | x | 35007_12_15047_XS2_1 | UnannotatedRegion |
| 0 | AAAAAAAAAA.AAACTTACCT | T | x | 35008_12_15661_XS2_1 | UnannotatedRegion |
| 0 | AAAAAAAAAA.AAACTTACCT | T | x | 35009_12_15657_XS2_1 | UnannotatedRegion |
| 0 | AAAAAAAAAA.AAACTTACCT | T | x | 35010_12_17186_XS2_1 | UnannotatedRegion |
| 0 | AAAAAAAAAA.AAACTTACCT | T | x | 35011_12_17194_XS2_1 | UnannotatedRegion |
| 0 | AAAAAAAAAA.AAACTTACCT | T | x | 35012_12_17009_XS2_1 | UnannotatedRegion |
| 0 | AAAAAAAAAA.AAACTTACCT | T | x | 35013_12_14973_XS2_1 | UnannotatedRegion |
|  |  |  |  |  |  |
| 11 | AAAAAGAAAG.GCAAACCATG | A | 1277332 F | 19260_WUE_2594 | UnannotatedRegion |
| 11 | AAAAAGAAAG.GCAAACCATG | G | x | 34990_Tchad54_11 | UnannotatedRegion |
| 11 | AAAAAGAAAG.GCAAACCATG | G | x | 34991_Tchad78_11 | UnannotatedRegion |
| 11 | AAAAAGAAAG.GCAAACCATG | G | x | 34992_Tchad95_11 | UnannotatedRegion |
| 11 | AAAAAGAAAG.GCAAACCATG | G | x | 34994_Tchad106_11 | UnannotatedRegion |
| 11 | AAAAAGAAAG.GCAAACCATG | G | x | 34995_8_2011 | UnannotatedRegion |
| 11 | AAAAAGAAAG.GCAAACCATG | G | x | 34996_10_2011 | UnannotatedRegion |
| 11 | AAAAAGAAAG.GCAAACCATG | G | x | 34997_39_2011 | UnannotatedRegion |
| 11 | AAAAAGAAAG.GCAAACCATG | G | x | 34998_59_2011 | UnannotatedRegion |
| 11 | AAAAAGAAAG.GCAAACCATG | G | x | 34999_63_2011 | UnannotatedRegion |
| 11 | AAAAAGAAAG.GCAAACCATG | G | x | 35000_75_2011 | UnannotatedRegion |
| 11 | AAAAAGAAAG.GCAAACCATG | G | x | 35001_76_2011 | UnannotatedRegion |
| 11 | AAAAAGAAAG.GCAAACCATG | G | x | 35002_120_2011 | UnannotatedRegion |
| 11 | AAAAAGAAAG.GCAAACCATG | G | x | 35003_403_2011 | UnannotatedRegion |
| 11 | AAAAAGAAAG.GCAAACCATG | G | x | 35004_12_15398_XS2_1 | UnannotatedRegion |
| 11 | AAAAAGAAAG.GCAAACCATG | G | x | 35005_12_13952_XS2_1 | UnannotatedRegion |
| 11 | AAAAAGAAAG.GCAAACCATG | G | x | 35006_12_15317_XS2_1 | UnannotatedRegion |
| 11 | AAAAAGAAAG.GCAAACCATG | G | x | 35007_12_15047_XS2_1 | UnannotatedRegion |
| 11 | AAAAAGAAAG.GCAAACCATG | G | x | 35008_12_15661_XS2_1 | UnannotatedRegion |
| 11 | AAAAAGAAAG.GCAAACCATG | G | x | 35009_12_15657_XS2_1 | UnannotatedRegion |
| 11 | AAAAAGAAAG.GCAAACCATG | G | x | 35010_12_17186_XS2_1 | UnannotatedRegion |
| 11 | AAAAAGAAAG.GCAAACCATG | G | x | 35011_12_17194_XS2_1 | UnannotatedRegion |
| 11 | AAAAAGAAAG.GCAAACCATG | G | x | 35012_12_17009_XS2_1 | UnannotatedRegion |
| 11 | AAAAAGAAAG.GCAAACCATG | G | x | 35013_12_14973_XS2_1 | UnannotatedRegion |
|  |  |  |  |  |  |
| 26 | AAAACGCCAA.CGCGCCTATT | A | x | 34990_Tchad54_11 | NotInAnnotatedGenome |
| 26 | AAAACGCCAA.CGCGCCTATT | A | x | 34991_Tchad78_11 | NotInAnnotatedGenome |
| 26 | AAAACGCCAA.CGCGCCTATT | A | x | 34992_Tchad95_11 | NotInAnnotatedGenome |
| 26 | AAAACGCCAA.CGCGCCTATT | A | x | 34994_Tchad106_11 | NotInAnnotatedGenome |
| 26 | AAAACGCCAA.CGCGCCTATT | A | x | 34995_8_2011 | NotInAnnotatedGenome |
| 26 | AAAACGCCAA.CGCGCCTATT | A | x | 34996_10_2011 | NotInAnnotatedGenome |
| 26 | AAAACGCCAA.CGCGCCTATT | A | x | 34997_39_2011 | NotInAnnotatedGenome |
| 26 | AAAACGCCAA.CGCGCCTATT | A | x | 34998_59_2011 | NotInAnnotatedGenome |
| 26 | AAAACGCCAA.CGCGCCTATT | A | x | 34999_63_2011 | NotInAnnotatedGenome |
| 26 | AAAACGCCAA.CGCGCCTATT | A | x | 35000_75_2011 | NotInAnnotatedGenome |
| 26 | AAAACGCCAA.CGCGCCTATT | A | x | 35001_76_2011 | NotInAnnotatedGenome |
| 26 | AAAACGCCAA.CGCGCCTATT | A | x | 35002_120_2011 | NotInAnnotatedGenome |
| 26 | AAAACGCCAA.CGCGCCTATT | A | x | 35003_403_2011 | NotInAnnotatedGenome |
| 26 | AAAACGCCAA.CGCGCCTATT | A | x | 35004_12_15398_XS2_1 | NotInAnnotatedGenome |
| 26 | AAAACGCCAA.CGCGCCTATT | A | x | 35005_12_13952_XS2_1 | NotInAnnotatedGenome |
| 26 | AAAACGCCAA.CGCGCCTATT | A | x | 35006_12_15317_XS2_1 | NotInAnnotatedGenome |
| 26 | AAAACGCCAA.CGCGCCTATT | C | x | 35007_12_15047_XS2_1 | NotInAnnotatedGenome |
| 26 | AAAACGCCAA.CGCGCCTATT | A | x | 35008_12_15661_XS2_1 | NotInAnnotatedGenome |
| 26 | AAAACGCCAA.CGCGCCTATT | A | x | 35009_12_15657_XS2_1 | NotInAnnotatedGenome |
| 26 | AAAACGCCAA.CGCGCCTATT | A | x | 35010_12_17186_XS2_1 | NotInAnnotatedGenome |
| 26 | AAAACGCCAA.CGCGCCTATT | A | x | 35011_12_17194_XS2_1 | NotInAnnotatedGenome |
| 26 | AAAACGCCAA.CGCGCCTATT | A | x | 35012_12_17009_XS2_1 | NotInAnnotatedGenome |
| 26 | AAAACGCCAA.CGCGCCTATT | A | x | 35013_12_14973_XS2_1 | NotInAnnotatedGenome |
|  |  |  |  |  |  |
| 28 | AAAACGGCCG.TATGTTTCTA | A | 510399 F | 19260_WUE_2594 | UnannotatedRegion |
| 28 | AAAACGGCCG.TATGTTTCTA | G | x | 34990_Tchad54_11 | UnannotatedRegion |
| 28 | AAAACGGCCG.TATGTTTCTA | G | x | 34991_Tchad78_11 | UnannotatedRegion |
| 28 | AAAACGGCCG.TATGTTTCTA | G | x | 34992_Tchad95_11 | UnannotatedRegion |
| 28 | AAAACGGCCG.TATGTTTCTA | G | x | 34994_Tchad106_11 | UnannotatedRegion |
| 28 | AAAACGGCCG.TATGTTTCTA | G | x | 34995_8_2011 | UnannotatedRegion |
| 28 | AAAACGGCCG.TATGTTTCTA | G | x | 34996_10_2011 | UnannotatedRegion |
| 28 | AAAACGGCCG.TATGTTTCTA | G | x | 34997_39_2011 | UnannotatedRegion |
| 28 | AAAACGGCCG.TATGTTTCTA | G | x | 34998_59_2011 | UnannotatedRegion |
| 28 | AAAACGGCCG.TATGTTTCTA | G | x | 34999_63_2011 | UnannotatedRegion |
| 28 | AAAACGGCCG.TATGTTTCTA | G | x | 35000_75_2011 | UnannotatedRegion |
| 28 | AAAACGGCCG.TATGTTTCTA | G | x | 35001_76_2011 | UnannotatedRegion |
| 28 | AAAACGGCCG.TATGTTTCTA | G | x | 35002_120_2011 | UnannotatedRegion |
| 28 | AAAACGGCCG.TATGTTTCTA | G | x | 35003_403_2011 | UnannotatedRegion |
| 28 | AAAACGGCCG.TATGTTTCTA | G | x | 35004_12_15398_XS2_1 | UnannotatedRegion |
| 28 | AAAACGGCCG.TATGTTTCTA | G | x | 35005_12_13952_XS2_1 | UnannotatedRegion |
| 28 | AAAACGGCCG.TATGTTTCTA | G | x | 35006_12_15317_XS2_1 | UnannotatedRegion |
| 28 | AAAACGGCCG.TATGTTTCTA | G | x | 35007_12_15047_XS2_1 | UnannotatedRegion |
| 28 | AAAACGGCCG.TATGTTTCTA | G | x | 35008_12_15661_XS2_1 | UnannotatedRegion |
| 28 | AAAACGGCCG.TATGTTTCTA | G | x | 35009_12_15657_XS2_1 | UnannotatedRegion |
| 28 | AAAACGGCCG.TATGTTTCTA | G | x | 35010_12_17186_XS2_1 | UnannotatedRegion |
| 28 | AAAACGGCCG.TATGTTTCTA | G | x | 35011_12_17194_XS2_1 | UnannotatedRegion |
| 28 | AAAACGGCCG.TATGTTTCTA | G | x | 35012_12_17009_XS2_1 | UnannotatedRegion |
| 28 | AAAACGGCCG.TATGTTTCTA | G | x | 35013_12_14973_XS2_1 | UnannotatedRegion |
|  |  |  |  |  |  |
| 47 | AAAATTACCT.ACTAAATTCA | T | 2195329 F | 19260_WUE_2594 | UnannotatedRegion |
| 47 | AAAATTACCT.ACTAAATTCA | T | x | 34990_Tchad54_11 | UnannotatedRegion |
| 47 | AAAATTACCT.ACTAAATTCA | T | x | 34991_Tchad78_11 | UnannotatedRegion |
| 47 | AAAATTACCT.ACTAAATTCA | T | x | 34992_Tchad95_11 | UnannotatedRegion |
| 47 | AAAATTACCT.ACTAAATTCA | T | x | 34994_Tchad106_11 | UnannotatedRegion |
| 47 | AAAATTACCT.ACTAAATTCA | G | x | 34995_8_2011 | UnannotatedRegion |
| 47 | AAAATTACCT.ACTAAATTCA | G | x | 34996_10_2011 | UnannotatedRegion |
| 47 | AAAATTACCT.ACTAAATTCA | T | x | 34997_39_2011 | UnannotatedRegion |
| 47 | AAAATTACCT.ACTAAATTCA | T | x | 34998_59_2011 | UnannotatedRegion |
| 47 | AAAATTACCT.ACTAAATTCA | T | x | 34999_63_2011 | UnannotatedRegion |
| 47 | AAAATTACCT.ACTAAATTCA | T | x | 35000_75_2011 | UnannotatedRegion |
| 47 | AAAATTACCT.ACTAAATTCA | T | x | 35001_76_2011 | UnannotatedRegion |
| 47 | AAAATTACCT.ACTAAATTCA | T | x | 35002_120_2011 | UnannotatedRegion |
| 47 | AAAATTACCT.ACTAAATTCA | T | x | 35003_403_2011 | UnannotatedRegion |
| 47 | AAAATTACCT.ACTAAATTCA | T | x | 35004_12_15398_XS2_1 | UnannotatedRegion |
| 47 | AAAATTACCT.ACTAAATTCA | T | x | 35005_12_13952_XS2_1 | UnannotatedRegion |
| 47 | AAAATTACCT.ACTAAATTCA | T | x | 35006_12_15317_XS2_1 | UnannotatedRegion |
| 47 | AAAATTACCT.ACTAAATTCA | T | x | 35007_12_15047_XS2_1 | UnannotatedRegion |
| 47 | AAAATTACCT.ACTAAATTCA | T | x | 35008_12_15661_XS2_1 | UnannotatedRegion |
| 47 | AAAATTACCT.ACTAAATTCA | T | x | 35009_12_15657_XS2_1 | UnannotatedRegion |
| 47 | AAAATTACCT.ACTAAATTCA | T | x | 35010_12_17186_XS2_1 | UnannotatedRegion |
| 47 | AAAATTACCT.ACTAAATTCA | T | x | 35011_12_17194_XS2_1 | UnannotatedRegion |
| 47 | AAAATTACCT.ACTAAATTCA | T | x | 35012_12_17009_XS2_1 | UnannotatedRegion |
| 47 | AAAATTACCT.ACTAAATTCA | T | x | 35013_12_14973_XS2_1 | UnannotatedRegion |
|  |  |  |  |  |  |
| 48 | AAAATTAGAC.TATTGTCCAT | G | 1121345 R | 19260_WUE_2594 | UnannotatedRegion |
| 48 | AAAATTAGAC.TATTGTCCAT | A | x | 34990_Tchad54_11 | UnannotatedRegion |
| 48 | AAAATTAGAC.TATTGTCCAT | A | x | 34991_Tchad78_11 | UnannotatedRegion |
| 48 | AAAATTAGAC.TATTGTCCAT | A | x | 34992_Tchad95_11 | UnannotatedRegion |
| 48 | AAAATTAGAC.TATTGTCCAT | A | x | 34995_8_2011 | UnannotatedRegion |
| 48 | AAAATTAGAC.TATTGTCCAT | A | x | 34996_10_2011 | UnannotatedRegion |
| 48 | AAAATTAGAC.TATTGTCCAT | A | x | 34997_39_2011 | UnannotatedRegion |
| 48 | AAAATTAGAC.TATTGTCCAT | A | x | 34998_59_2011 | UnannotatedRegion |
| 48 | AAAATTAGAC.TATTGTCCAT | A | x | 34999_63_2011 | UnannotatedRegion |
| 48 | AAAATTAGAC.TATTGTCCAT | A | x | 35000_75_2011 | UnannotatedRegion |
| 48 | AAAATTAGAC.TATTGTCCAT | A | x | 35001_76_2011 | UnannotatedRegion |
| 48 | AAAATTAGAC.TATTGTCCAT | A | x | 35002_120_2011 | UnannotatedRegion |
| 48 | AAAATTAGAC.TATTGTCCAT | A | x | 35003_403_2011 | UnannotatedRegion |
| 48 | AAAATTAGAC.TATTGTCCAT | A | x | 35004_12_15398_XS2_1 | UnannotatedRegion |
| 48 | AAAATTAGAC.TATTGTCCAT | A | x | 35005_12_13952_XS2_1 | UnannotatedRegion |
| 48 | AAAATTAGAC.TATTGTCCAT | A | x | 35006_12_15317_XS2_1 | UnannotatedRegion |
| 48 | AAAATTAGAC.TATTGTCCAT | A | x | 35007_12_15047_XS2_1 | UnannotatedRegion |
| 48 | AAAATTAGAC.TATTGTCCAT | A | x | 35008_12_15661_XS2_1 | UnannotatedRegion |
| 48 | AAAATTAGAC.TATTGTCCAT | A | x | 35009_12_15657_XS2_1 | UnannotatedRegion |
| 48 | AAAATTAGAC.TATTGTCCAT | A | x | 35010_12_17186_XS2_1 | UnannotatedRegion |
| 48 | AAAATTAGAC.TATTGTCCAT | A | x | 35011_12_17194_XS2_1 | UnannotatedRegion |
| 48 | AAAATTAGAC.TATTGTCCAT | A | x | 35012_12_17009_XS2_1 | UnannotatedRegion |
| 48 | AAAATTAGAC.TATTGTCCAT | A | x | 35013_12_14973_XS2_1 | UnannotatedRegion |
|  |  |  |  |  |  |
| 55 | AAACAGGCAA.AAAACCAATC | C | 1106942 R | 19260_WUE_2594 | UnannotatedRegion |
| 55 | AAACAGGCAA.AAAACCAATC | C | x | 34990_Tchad54_11 | UnannotatedRegion |
| 55 | AAACAGGCAA.AAAACCAATC | T | x | 34991_Tchad78_11 | UnannotatedRegion |
| 55 | AAACAGGCAA.AAAACCAATC | C | x | 34992_Tchad95_11 | UnannotatedRegion |
| 55 | AAACAGGCAA.AAAACCAATC | C | x | 34994_Tchad106_11 | UnannotatedRegion |
| 55 | AAACAGGCAA.AAAACCAATC | C | x | 34995_8_2011 | UnannotatedRegion |
| 55 | AAACAGGCAA.AAAACCAATC | C | x | 34996_10_2011 | UnannotatedRegion |
| 55 | AAACAGGCAA.AAAACCAATC | C | x | 34997_39_2011 | UnannotatedRegion |
| 55 | AAACAGGCAA.AAAACCAATC | C | x | 34998_59_2011 | UnannotatedRegion |
| 55 | AAACAGGCAA.AAAACCAATC | C | x | 34999_63_2011 | UnannotatedRegion |
| 55 | AAACAGGCAA.AAAACCAATC | C | x | 35000_75_2011 | UnannotatedRegion |
| 55 | AAACAGGCAA.AAAACCAATC | C | x | 35001_76_2011 | UnannotatedRegion |
| 55 | AAACAGGCAA.AAAACCAATC | C | x | 35002_120_2011 | UnannotatedRegion |
| 55 | AAACAGGCAA.AAAACCAATC | C | x | 35003_403_2011 | UnannotatedRegion |
| 55 | AAACAGGCAA.AAAACCAATC | C | x | 35004_12_15398_XS2_1 | UnannotatedRegion |
| 55 | AAACAGGCAA.AAAACCAATC | C | x | 35005_12_13952_XS2_1 | UnannotatedRegion |
| 55 | AAACAGGCAA.AAAACCAATC | C | x | 35006_12_15317_XS2_1 | UnannotatedRegion |
| 55 | AAACAGGCAA.AAAACCAATC | C | x | 35007_12_15047_XS2_1 | UnannotatedRegion |
| 55 | AAACAGGCAA.AAAACCAATC | C | x | 35008_12_15661_XS2_1 | UnannotatedRegion |
| 55 | AAACAGGCAA.AAAACCAATC | C | x | 35009_12_15657_XS2_1 | UnannotatedRegion |
| 55 | AAACAGGCAA.AAAACCAATC | C | x | 35010_12_17186_XS2_1 | UnannotatedRegion |
| 55 | AAACAGGCAA.AAAACCAATC | C | x | 35011_12_17194_XS2_1 | UnannotatedRegion |
| 55 | AAACAGGCAA.AAAACCAATC | C | x | 35012_12_17009_XS2_1 | UnannotatedRegion |
| 55 | AAACAGGCAA.AAAACCAATC | C | x | 35013_12_14973_XS2_1 | UnannotatedRegion |
|  |  |  |  |  |  |
| 58 | AAACATTTCA.ACGGCCTGAA | G | 2161039 R | 19260_WUE_2594 | UnannotatedRegion |
| 58 | AAACATTTCA.ACGGCCTGAA | G | x | 34990_Tchad54_11 | UnannotatedRegion |
| 58 | AAACATTTCA.ACGGCCTGAA | G | x | 34991_Tchad78_11 | UnannotatedRegion |
| 58 | AAACATTTCA.ACGGCCTGAA | A | x | 34992_Tchad95_11 | UnannotatedRegion |
| 58 | AAACATTTCA.ACGGCCTGAA | A | x | 34994_Tchad106_11 | UnannotatedRegion |
| 58 | AAACATTTCA.ACGGCCTGAA | G | x | 34995_8_2011 | UnannotatedRegion |
| 58 | AAACATTTCA.ACGGCCTGAA | G | x | 34996_10_2011 | UnannotatedRegion |
| 58 | AAACATTTCA.ACGGCCTGAA | G | x | 34997_39_2011 | UnannotatedRegion |
| 58 | AAACATTTCA.ACGGCCTGAA | G | x | 34998_59_2011 | UnannotatedRegion |
| 58 | AAACATTTCA.ACGGCCTGAA | G | x | 34999_63_2011 | UnannotatedRegion |
| 58 | AAACATTTCA.ACGGCCTGAA | G | x | 35000_75_2011 | UnannotatedRegion |
| 58 | AAACATTTCA.ACGGCCTGAA | G | x | 35001_76_2011 | UnannotatedRegion |
| 58 | AAACATTTCA.ACGGCCTGAA | G | x | 35002_120_2011 | UnannotatedRegion |
| 58 | AAACATTTCA.ACGGCCTGAA | G | x | 35003_403_2011 | UnannotatedRegion |
| 58 | AAACATTTCA.ACGGCCTGAA | G | x | 35004_12_15398_XS2_1 | UnannotatedRegion |
| 58 | AAACATTTCA.ACGGCCTGAA | G | x | 35005_12_13952_XS2_1 | UnannotatedRegion |
| 58 | AAACATTTCA.ACGGCCTGAA | G | x | 35006_12_15317_XS2_1 | UnannotatedRegion |
| 58 | AAACATTTCA.ACGGCCTGAA | A | x | 35007_12_15047_XS2_1 | UnannotatedRegion |
| 58 | AAACATTTCA.ACGGCCTGAA | A | x | 35008_12_15661_XS2_1 | UnannotatedRegion |
| 58 | AAACATTTCA.ACGGCCTGAA | A | x | 35009_12_15657_XS2_1 | UnannotatedRegion |
| 58 | AAACATTTCA.ACGGCCTGAA | G | x | 35010_12_17186_XS2_1 | UnannotatedRegion |
| 58 | AAACATTTCA.ACGGCCTGAA | G | x | 35011_12_17194_XS2_1 | UnannotatedRegion |
| 58 | AAACATTTCA.ACGGCCTGAA | G | x | 35012_12_17009_XS2_1 | UnannotatedRegion |
| 58 | AAACATTTCA.ACGGCCTGAA | A | x | 35013_12_14973_XS2_1 | UnannotatedRegion |
|  |  |  |  |  |  |
| 66 | AAACCTATCT.GCCGCATTCC | C | 1436075 F | 19260_WUE_2594 | UnannotatedRegion |
| 66 | AAACCTATCT.GCCGCATTCC | T | x | 34990_Tchad54_11 | UnannotatedRegion |
| 66 | AAACCTATCT.GCCGCATTCC | T | x | 34991_Tchad78_11 | UnannotatedRegion |
| 66 | AAACCTATCT.GCCGCATTCC | T | x | 34992_Tchad95_11 | UnannotatedRegion |
| 66 | AAACCTATCT.GCCGCATTCC | T | x | 34994_Tchad106_11 | UnannotatedRegion |
| 66 | AAACCTATCT.GCCGCATTCC | T | x | 34995_8_2011 | UnannotatedRegion |
| 66 | AAACCTATCT.GCCGCATTCC | T | x | 34996_10_2011 | UnannotatedRegion |
| 66 | AAACCTATCT.GCCGCATTCC | T | x | 34997_39_2011 | UnannotatedRegion |
| 66 | AAACCTATCT.GCCGCATTCC | T | x | 34998_59_2011 | UnannotatedRegion |
| 66 | AAACCTATCT.GCCGCATTCC | T | x | 34999_63_2011 | UnannotatedRegion |
| 66 | AAACCTATCT.GCCGCATTCC | T | x | 35000_75_2011 | UnannotatedRegion |
| 66 | AAACCTATCT.GCCGCATTCC | T | x | 35001_76_2011 | UnannotatedRegion |
| 66 | AAACCTATCT.GCCGCATTCC | T | x | 35002_120_2011 | UnannotatedRegion |
| 66 | AAACCTATCT.GCCGCATTCC | T | x | 35003_403_2011 | UnannotatedRegion |
| 66 | AAACCTATCT.GCCGCATTCC | T | x | 35004_12_15398_XS2_1 | UnannotatedRegion |
| 66 | AAACCTATCT.GCCGCATTCC | T | x | 35005_12_13952_XS2_1 | UnannotatedRegion |
| 66 | AAACCTATCT.GCCGCATTCC | T | x | 35006_12_15317_XS2_1 | UnannotatedRegion |
| 66 | AAACCTATCT.GCCGCATTCC | T | x | 35007_12_15047_XS2_1 | UnannotatedRegion |
| 66 | AAACCTATCT.GCCGCATTCC | T | x | 35008_12_15661_XS2_1 | UnannotatedRegion |
| 66 | AAACCTATCT.GCCGCATTCC | T | x | 35009_12_15657_XS2_1 | UnannotatedRegion |
| 66 | AAACCTATCT.GCCGCATTCC | T | x | 35010_12_17186_XS2_1 | UnannotatedRegion |
| 66 | AAACCTATCT.GCCGCATTCC | T | x | 35011_12_17194_XS2_1 | UnannotatedRegion |
| 66 | AAACCTATCT.GCCGCATTCC | T | x | 35012_12_17009_XS2_1 | UnannotatedRegion |
| 66 | AAACCTATCT.GCCGCATTCC | T | x | 35013_12_14973_XS2_1 | UnannotatedRegion |
|  |  |  |  |  |  |
| 77 | AAAGCAAAAC.CCGCCCCATC | A | 858300 F | 19260_WUE_2594 | UnannotatedRegion |
| 77 | AAAGCAAAAC.CCGCCCCATC | G | x | 34990_Tchad54_11 | UnannotatedRegion |
| 77 | AAAGCAAAAC.CCGCCCCATC | G | x | 34991_Tchad78_11 | UnannotatedRegion |
| 77 | AAAGCAAAAC.CCGCCCCATC | G | x | 34992_Tchad95_11 | UnannotatedRegion |
| 77 | AAAGCAAAAC.CCGCCCCATC | G | x | 34994_Tchad106_11 | UnannotatedRegion |
| 77 | AAAGCAAAAC.CCGCCCCATC | G | x | 34995_8_2011 | UnannotatedRegion |
| 77 | AAAGCAAAAC.CCGCCCCATC | G | x | 34996_10_2011 | UnannotatedRegion |
| 77 | AAAGCAAAAC.CCGCCCCATC | G | x | 34997_39_2011 | UnannotatedRegion |
| 77 | AAAGCAAAAC.CCGCCCCATC | G | x | 34998_59_2011 | UnannotatedRegion |
| 77 | AAAGCAAAAC.CCGCCCCATC | G | x | 34999_63_2011 | UnannotatedRegion |
| 77 | AAAGCAAAAC.CCGCCCCATC | G | x | 35000_75_2011 | UnannotatedRegion |
| 77 | AAAGCAAAAC.CCGCCCCATC | G | x | 35001_76_2011 | UnannotatedRegion |
| 77 | AAAGCAAAAC.CCGCCCCATC | G | x | 35002_120_2011 | UnannotatedRegion |
| 77 | AAAGCAAAAC.CCGCCCCATC | G | x | 35003_403_2011 | UnannotatedRegion |
| 77 | AAAGCAAAAC.CCGCCCCATC | G | x | 35004_12_15398_XS2_1 | UnannotatedRegion |
| 77 | AAAGCAAAAC.CCGCCCCATC | G | x | 35005_12_13952_XS2_1 | UnannotatedRegion |
| 77 | AAAGCAAAAC.CCGCCCCATC | G | x | 35006_12_15317_XS2_1 | UnannotatedRegion |
| 77 | AAAGCAAAAC.CCGCCCCATC | G | x | 35007_12_15047_XS2_1 | UnannotatedRegion |
| 77 | AAAGCAAAAC.CCGCCCCATC | G | x | 35008_12_15661_XS2_1 | UnannotatedRegion |
| 77 | AAAGCAAAAC.CCGCCCCATC | G | x | 35009_12_15657_XS2_1 | UnannotatedRegion |
| 77 | AAAGCAAAAC.CCGCCCCATC | G | x | 35010_12_17186_XS2_1 | UnannotatedRegion |
| 77 | AAAGCAAAAC.CCGCCCCATC | G | x | 35011_12_17194_XS2_1 | UnannotatedRegion |
| 77 | AAAGCAAAAC.CCGCCCCATC | G | x | 35012_12_17009_XS2_1 | UnannotatedRegion |
| 77 | AAAGCAAAAC.CCGCCCCATC | G | x | 35013_12_14973_XS2_1 | UnannotatedRegion |
|  |  |  |  |  |  |
| 81 | AAAGCGAAGA.TAGGGAAACA | G | 1731001 R | 19260_WUE_2594 | UnannotatedRegion |
| 81 | AAAGCGAAGA.TAGGGAAACA | T | x | 34990_Tchad54_11 | UnannotatedRegion |
| 81 | AAAGCGAAGA.TAGGGAAACA | T | x | 34991_Tchad78_11 | UnannotatedRegion |
| 81 | AAAGCGAAGA.TAGGGAAACA | T | x | 34992_Tchad95_11 | UnannotatedRegion |
| 81 | AAAGCGAAGA.TAGGGAAACA | T | x | 34994_Tchad106_11 | UnannotatedRegion |
| 81 | AAAGCGAAGA.TAGGGAAACA | T | x | 34995_8_2011 | UnannotatedRegion |
| 81 | AAAGCGAAGA.TAGGGAAACA | T | x | 34996_10_2011 | UnannotatedRegion |
| 81 | AAAGCGAAGA.TAGGGAAACA | T | x | 34997_39_2011 | UnannotatedRegion |
| 81 | AAAGCGAAGA.TAGGGAAACA | T | x | 34998_59_2011 | UnannotatedRegion |
| 81 | AAAGCGAAGA.TAGGGAAACA | T | x | 34999_63_2011 | UnannotatedRegion |
| 81 | AAAGCGAAGA.TAGGGAAACA | T | x | 35000_75_2011 | UnannotatedRegion |
| 81 | AAAGCGAAGA.TAGGGAAACA | T | x | 35001_76_2011 | UnannotatedRegion |
| 81 | AAAGCGAAGA.TAGGGAAACA | T | x | 35002_120_2011 | UnannotatedRegion |
| 81 | AAAGCGAAGA.TAGGGAAACA | T | x | 35003_403_2011 | UnannotatedRegion |
| 81 | AAAGCGAAGA.TAGGGAAACA | T | x | 35004_12_15398_XS2_1 | UnannotatedRegion |
| 81 | AAAGCGAAGA.TAGGGAAACA | T | x | 35005_12_13952_XS2_1 | UnannotatedRegion |
| 81 | AAAGCGAAGA.TAGGGAAACA | T | x | 35006_12_15317_XS2_1 | UnannotatedRegion |
| 81 | AAAGCGAAGA.TAGGGAAACA | G | x | 35007_12_15047_XS2_1 | UnannotatedRegion |
| 81 | AAAGCGAAGA.TAGGGAAACA | T | x | 35008_12_15661_XS2_1 | UnannotatedRegion |
| 81 | AAAGCGAAGA.TAGGGAAACA | T | x | 35009_12_15657_XS2_1 | UnannotatedRegion |
| 81 | AAAGCGAAGA.TAGGGAAACA | T | x | 35010_12_17186_XS2_1 | UnannotatedRegion |
| 81 | AAAGCGAAGA.TAGGGAAACA | T | x | 35011_12_17194_XS2_1 | UnannotatedRegion |
| 81 | AAAGCGAAGA.TAGGGAAACA | T | x | 35012_12_17009_XS2_1 | UnannotatedRegion |
| 81 | AAAGCGAAGA.TAGGGAAACA | T | x | 35013_12_14973_XS2_1 | UnannotatedRegion |
|  |  |  |  |  |  |
| 86 | AAAGGTTACA.TGAAAATTGT | G | 1741250 R | 19260_WUE_2594 | UnannotatedRegion |
| 86 | AAAGGTTACA.TGAAAATTGT | A | x | 34990_Tchad54_11 | UnannotatedRegion |
| 86 | AAAGGTTACA.TGAAAATTGT | A | x | 34991_Tchad78_11 | UnannotatedRegion |
| 86 | AAAGGTTACA.TGAAAATTGT | A | x | 34992_Tchad95_11 | UnannotatedRegion |
| 86 | AAAGGTTACA.TGAAAATTGT | A | x | 34994_Tchad106_11 | UnannotatedRegion |
| 86 | AAAGGTTACA.TGAAAATTGT | A | x | 34995_8_2011 | UnannotatedRegion |
| 86 | AAAGGTTACA.TGAAAATTGT | A | x | 34996_10_2011 | UnannotatedRegion |
| 86 | AAAGGTTACA.TGAAAATTGT | A | x | 34997_39_2011 | UnannotatedRegion |
| 86 | AAAGGTTACA.TGAAAATTGT | A | x | 34998_59_2011 | UnannotatedRegion |
| 86 | AAAGGTTACA.TGAAAATTGT | A | x | 34999_63_2011 | UnannotatedRegion |
| 86 | AAAGGTTACA.TGAAAATTGT | A | x | 35000_75_2011 | UnannotatedRegion |
| 86 | AAAGGTTACA.TGAAAATTGT | A | x | 35001_76_2011 | UnannotatedRegion |
| 86 | AAAGGTTACA.TGAAAATTGT | A | x | 35002_120_2011 | UnannotatedRegion |
| 86 | AAAGGTTACA.TGAAAATTGT | A | x | 35003_403_2011 | UnannotatedRegion |
| 86 | AAAGGTTACA.TGAAAATTGT | A | x | 35004_12_15398_XS2_1 | UnannotatedRegion |
| 86 | AAAGGTTACA.TGAAAATTGT | A | x | 35005_12_13952_XS2_1 | UnannotatedRegion |
| 86 | AAAGGTTACA.TGAAAATTGT | A | x | 35006_12_15317_XS2_1 | UnannotatedRegion |
| 86 | AAAGGTTACA.TGAAAATTGT | A | x | 35007_12_15047_XS2_1 | UnannotatedRegion |
| 86 | AAAGGTTACA.TGAAAATTGT | A | x | 35008_12_15661_XS2_1 | UnannotatedRegion |
| 86 | AAAGGTTACA.TGAAAATTGT | A | x | 35009_12_15657_XS2_1 | UnannotatedRegion |
| 86 | AAAGGTTACA.TGAAAATTGT | A | x | 35010_12_17186_XS2_1 | UnannotatedRegion |
| 86 | AAAGGTTACA.TGAAAATTGT | A | x | 35011_12_17194_XS2_1 | UnannotatedRegion |
| 86 | AAAGGTTACA.TGAAAATTGT | A | x | 35012_12_17009_XS2_1 | UnannotatedRegion |
| 86 | AAAGGTTACA.TGAAAATTGT | A | x | 35013_12_14973_XS2_1 | UnannotatedRegion |
|  |  |  |  |  |  |
| 87 | AAAGGTTGGG.GAGGGTTGGC | G | 1962065 R | 19260_WUE_2594 | UnannotatedRegion |
| 87 | AAAGGTTGGG.GAGGGTTGGC | A | x | 34990_Tchad54_11 | UnannotatedRegion |
| 87 | AAAGGTTGGG.GAGGGTTGGC | A | x | 34991_Tchad78_11 | UnannotatedRegion |
| 87 | AAAGGTTGGG.GAGGGTTGGC | A | x | 34992_Tchad95_11 | UnannotatedRegion |
| 87 | AAAGGTTGGG.GAGGGTTGGC | A | x | 34994_Tchad106_11 | UnannotatedRegion |
| 87 | AAAGGTTGGG.GAGGGTTGGC | A | x | 34995_8_2011 | UnannotatedRegion |
| 87 | AAAGGTTGGG.GAGGGTTGGC | A | x | 34996_10_2011 | UnannotatedRegion |
| 87 | AAAGGTTGGG.GAGGGTTGGC | A | x | 34997_39_2011 | UnannotatedRegion |
| 87 | AAAGGTTGGG.GAGGGTTGGC | A | x | 34998_59_2011 | UnannotatedRegion |
| 87 | AAAGGTTGGG.GAGGGTTGGC | A | x | 34999_63_2011 | UnannotatedRegion |
| 87 | AAAGGTTGGG.GAGGGTTGGC | A | x | 35000_75_2011 | UnannotatedRegion |
| 87 | AAAGGTTGGG.GAGGGTTGGC | A | x | 35001_76_2011 | UnannotatedRegion |
| 87 | AAAGGTTGGG.GAGGGTTGGC | A | x | 35002_120_2011 | UnannotatedRegion |
| 87 | AAAGGTTGGG.GAGGGTTGGC | A | x | 35003_403_2011 | UnannotatedRegion |
| 87 | AAAGGTTGGG.GAGGGTTGGC | A | x | 35004_12_15398_XS2_1 | UnannotatedRegion |
| 87 | AAAGGTTGGG.GAGGGTTGGC | A | x | 35005_12_13952_XS2_1 | UnannotatedRegion |
| 87 | AAAGGTTGGG.GAGGGTTGGC | A | x | 35006_12_15317_XS2_1 | UnannotatedRegion |
| 87 | AAAGGTTGGG.GAGGGTTGGC | A | x | 35007_12_15047_XS2_1 | UnannotatedRegion |
| 87 | AAAGGTTGGG.GAGGGTTGGC | A | x | 35008_12_15661_XS2_1 | UnannotatedRegion |
| 87 | AAAGGTTGGG.GAGGGTTGGC | A | x | 35009_12_15657_XS2_1 | UnannotatedRegion |
| 87 | AAAGGTTGGG.GAGGGTTGGC | A | x | 35010_12_17186_XS2_1 | UnannotatedRegion |
| 87 | AAAGGTTGGG.GAGGGTTGGC | A | x | 35011_12_17194_XS2_1 | UnannotatedRegion |
| 87 | AAAGGTTGGG.GAGGGTTGGC | A | x | 35012_12_17009_XS2_1 | UnannotatedRegion |
| 87 | AAAGGTTGGG.GAGGGTTGGC | A | x | 35013_12_14973_XS2_1 | UnannotatedRegion |
|  |  |  |  |  |  |
| 103 | AAATCGGGAA.ATGCCGTCTG | C | 740510 F | 19260_WUE_2594 | UnannotatedRegion |
| 103 | AAATCGGGAA.ATGCCGTCTG | A | x | 34990_Tchad54_11 | UnannotatedRegion |
| 103 | AAATCGGGAA.ATGCCGTCTG | A | x | 34991_Tchad78_11 | UnannotatedRegion |
| 103 | AAATCGGGAA.ATGCCGTCTG | A | x | 34992_Tchad95_11 | UnannotatedRegion |
| 103 | AAATCGGGAA.ATGCCGTCTG | A | x | 34994_Tchad106_11 | UnannotatedRegion |
| 103 | AAATCGGGAA.ATGCCGTCTG | A | x | 34995_8_2011 | UnannotatedRegion |
| 103 | AAATCGGGAA.ATGCCGTCTG | A | x | 34996_10_2011 | UnannotatedRegion |
| 103 | AAATCGGGAA.ATGCCGTCTG | A | x | 34997_39_2011 | UnannotatedRegion |
| 103 | AAATCGGGAA.ATGCCGTCTG | A | x | 34998_59_2011 | UnannotatedRegion |
| 103 | AAATCGGGAA.ATGCCGTCTG | A | x | 34999_63_2011 | UnannotatedRegion |
| 103 | AAATCGGGAA.ATGCCGTCTG | A | x | 35000_75_2011 | UnannotatedRegion |
| 103 | AAATCGGGAA.ATGCCGTCTG | A | x | 35001_76_2011 | UnannotatedRegion |
| 103 | AAATCGGGAA.ATGCCGTCTG | A | x | 35002_120_2011 | UnannotatedRegion |
| 103 | AAATCGGGAA.ATGCCGTCTG | A | x | 35003_403_2011 | UnannotatedRegion |
| 103 | AAATCGGGAA.ATGCCGTCTG | A | x | 35004_12_15398_XS2_1 | UnannotatedRegion |
| 103 | AAATCGGGAA.ATGCCGTCTG | A | x | 35005_12_13952_XS2_1 | UnannotatedRegion |
| 103 | AAATCGGGAA.ATGCCGTCTG | A | x | 35006_12_15317_XS2_1 | UnannotatedRegion |
| 103 | AAATCGGGAA.ATGCCGTCTG | A | x | 35007_12_15047_XS2_1 | UnannotatedRegion |
| 103 | AAATCGGGAA.ATGCCGTCTG | A | x | 35008_12_15661_XS2_1 | UnannotatedRegion |
| 103 | AAATCGGGAA.ATGCCGTCTG | A | x | 35009_12_15657_XS2_1 | UnannotatedRegion |
| 103 | AAATCGGGAA.ATGCCGTCTG | A | x | 35010_12_17186_XS2_1 | UnannotatedRegion |
| 103 | AAATCGGGAA.ATGCCGTCTG | A | x | 35011_12_17194_XS2_1 | UnannotatedRegion |
| 103 | AAATCGGGAA.ATGCCGTCTG | A | x | 35012_12_17009_XS2_1 | UnannotatedRegion |
| 103 | AAATCGGGAA.ATGCCGTCTG | A | x | 35013_12_14973_XS2_1 | UnannotatedRegion |
|  |  |  |  |  |  |
| 118 | AAATTCAAGC.GTTCAATGTG | A | 866155 R | 19260_WUE_2594 | UnannotatedRegion |
| 118 | AAATTCAAGC.GTTCAATGTG | A | x | 34990_Tchad54_11 | UnannotatedRegion |
| 118 | AAATTCAAGC.GTTCAATGTG | A | x | 34991_Tchad78_11 | UnannotatedRegion |
| 118 | AAATTCAAGC.GTTCAATGTG | A | x | 34992_Tchad95_11 | UnannotatedRegion |
| 118 | AAATTCAAGC.GTTCAATGTG | A | x | 34994_Tchad106_11 | UnannotatedRegion |
| 118 | AAATTCAAGC.GTTCAATGTG | G | x | 34995_8_2011 | UnannotatedRegion |
| 118 | AAATTCAAGC.GTTCAATGTG | G | x | 34996_10_2011 | UnannotatedRegion |
| 118 | AAATTCAAGC.GTTCAATGTG | A | x | 34997_39_2011 | UnannotatedRegion |
| 118 | AAATTCAAGC.GTTCAATGTG | A | x | 34998_59_2011 | UnannotatedRegion |
| 118 | AAATTCAAGC.GTTCAATGTG | A | x | 34999_63_2011 | UnannotatedRegion |
| 118 | AAATTCAAGC.GTTCAATGTG | A | x | 35000_75_2011 | UnannotatedRegion |
| 118 | AAATTCAAGC.GTTCAATGTG | A | x | 35001_76_2011 | UnannotatedRegion |
| 118 | AAATTCAAGC.GTTCAATGTG | A | x | 35002_120_2011 | UnannotatedRegion |
| 118 | AAATTCAAGC.GTTCAATGTG | A | x | 35003_403_2011 | UnannotatedRegion |
| 118 | AAATTCAAGC.GTTCAATGTG | A | x | 35004_12_15398_XS2_1 | UnannotatedRegion |
| 118 | AAATTCAAGC.GTTCAATGTG | A | x | 35005_12_13952_XS2_1 | UnannotatedRegion |
| 118 | AAATTCAAGC.GTTCAATGTG | A | x | 35006_12_15317_XS2_1 | UnannotatedRegion |
| 118 | AAATTCAAGC.GTTCAATGTG | A | x | 35007_12_15047_XS2_1 | UnannotatedRegion |
| 118 | AAATTCAAGC.GTTCAATGTG | A | x | 35008_12_15661_XS2_1 | UnannotatedRegion |
| 118 | AAATTCAAGC.GTTCAATGTG | A | x | 35009_12_15657_XS2_1 | UnannotatedRegion |
| 118 | AAATTCAAGC.GTTCAATGTG | A | x | 35010_12_17186_XS2_1 | UnannotatedRegion |
| 118 | AAATTCAAGC.GTTCAATGTG | A | x | 35011_12_17194_XS2_1 | UnannotatedRegion |
| 118 | AAATTCAAGC.GTTCAATGTG | A | x | 35012_12_17009_XS2_1 | UnannotatedRegion |
| 118 | AAATTCAAGC.GTTCAATGTG | A | x | 35013_12_14973_XS2_1 | UnannotatedRegion |
|  |  |  |  |  |  |
| 120 | AAATTTCCCG.CAAAATTGAC | T | 1619707 F | 19260_WUE_2594 | UnannotatedRegion |
| 120 | AAATTTCCCG.CAAAATTGAC | G | x | 34990_Tchad54_11 | UnannotatedRegion |
| 120 | AAATTTCCCG.CAAAATTGAC | G | x | 34991_Tchad78_11 | UnannotatedRegion |
| 120 | AAATTTCCCG.CAAAATTGAC | G | x | 34992_Tchad95_11 | UnannotatedRegion |
| 120 | AAATTTCCCG.CAAAATTGAC | G | x | 34994_Tchad106_11 | UnannotatedRegion |
| 120 | AAATTTCCCG.CAAAATTGAC | G | x | 34995_8_2011 | UnannotatedRegion |
| 120 | AAATTTCCCG.CAAAATTGAC | G | x | 34996_10_2011 | UnannotatedRegion |
| 120 | AAATTTCCCG.CAAAATTGAC | G | x | 34997_39_2011 | UnannotatedRegion |
| 120 | AAATTTCCCG.CAAAATTGAC | G | x | 34998_59_2011 | UnannotatedRegion |
| 120 | AAATTTCCCG.CAAAATTGAC | G | x | 34999_63_2011 | UnannotatedRegion |
| 120 | AAATTTCCCG.CAAAATTGAC | G | x | 35000_75_2011 | UnannotatedRegion |
| 120 | AAATTTCCCG.CAAAATTGAC | G | x | 35001_76_2011 | UnannotatedRegion |
| 120 | AAATTTCCCG.CAAAATTGAC | G | x | 35002_120_2011 | UnannotatedRegion |
| 120 | AAATTTCCCG.CAAAATTGAC | G | x | 35003_403_2011 | UnannotatedRegion |
| 120 | AAATTTCCCG.CAAAATTGAC | G | x | 35004_12_15398_XS2_1 | UnannotatedRegion |
| 120 | AAATTTCCCG.CAAAATTGAC | G | x | 35005_12_13952_XS2_1 | UnannotatedRegion |
| 120 | AAATTTCCCG.CAAAATTGAC | G | x | 35006_12_15317_XS2_1 | UnannotatedRegion |
| 120 | AAATTTCCCG.CAAAATTGAC | G | x | 35007_12_15047_XS2_1 | UnannotatedRegion |
| 120 | AAATTTCCCG.CAAAATTGAC | G | x | 35008_12_15661_XS2_1 | UnannotatedRegion |
| 120 | AAATTTCCCG.CAAAATTGAC | G | x | 35009_12_15657_XS2_1 | UnannotatedRegion |
| 120 | AAATTTCCCG.CAAAATTGAC | G | x | 35010_12_17186_XS2_1 | UnannotatedRegion |
| 120 | AAATTTCCCG.CAAAATTGAC | G | x | 35011_12_17194_XS2_1 | UnannotatedRegion |
| 120 | AAATTTCCCG.CAAAATTGAC | G | x | 35012_12_17009_XS2_1 | UnannotatedRegion |
| 120 | AAATTTCCCG.CAAAATTGAC | G | x | 35013_12_14973_XS2_1 | UnannotatedRegion |
|  |  |  |  |  |  |
| 122 | AAATTTGCGG.ATCCGCCGCA | A | 2037522 R | 19260_WUE_2594 | UnannotatedRegion |
| 122 | AAATTTGCGG.ATCCGCCGCA | C | x | 34990_Tchad54_11 | UnannotatedRegion |
| 122 | AAATTTGCGG.ATCCGCCGCA | C | x | 34991_Tchad78_11 | UnannotatedRegion |
| 122 | AAATTTGCGG.ATCCGCCGCA | C | x | 34992_Tchad95_11 | UnannotatedRegion |
| 122 | AAATTTGCGG.ATCCGCCGCA | C | x | 34994_Tchad106_11 | UnannotatedRegion |
| 122 | AAATTTGCGG.ATCCGCCGCA | C | x | 34995_8_2011 | UnannotatedRegion |
| 122 | AAATTTGCGG.ATCCGCCGCA | C | x | 34996_10_2011 | UnannotatedRegion |
| 122 | AAATTTGCGG.ATCCGCCGCA | C | x | 34997_39_2011 | UnannotatedRegion |
| 122 | AAATTTGCGG.ATCCGCCGCA | C | x | 34998_59_2011 | UnannotatedRegion |
| 122 | AAATTTGCGG.ATCCGCCGCA | C | x | 34999_63_2011 | UnannotatedRegion |
| 122 | AAATTTGCGG.ATCCGCCGCA | C | x | 35000_75_2011 | UnannotatedRegion |
| 122 | AAATTTGCGG.ATCCGCCGCA | C | x | 35001_76_2011 | UnannotatedRegion |
| 122 | AAATTTGCGG.ATCCGCCGCA | C | x | 35002_120_2011 | UnannotatedRegion |
| 122 | AAATTTGCGG.ATCCGCCGCA | C | x | 35003_403_2011 | UnannotatedRegion |
| 122 | AAATTTGCGG.ATCCGCCGCA | C | x | 35004_12_15398_XS2_1 | UnannotatedRegion |
| 122 | AAATTTGCGG.ATCCGCCGCA | C | x | 35005_12_13952_XS2_1 | UnannotatedRegion |
| 122 | AAATTTGCGG.ATCCGCCGCA | C | x | 35006_12_15317_XS2_1 | UnannotatedRegion |
| 122 | AAATTTGCGG.ATCCGCCGCA | C | x | 35007_12_15047_XS2_1 | UnannotatedRegion |
| 122 | AAATTTGCGG.ATCCGCCGCA | C | x | 35008_12_15661_XS2_1 | UnannotatedRegion |
| 122 | AAATTTGCGG.ATCCGCCGCA | C | x | 35009_12_15657_XS2_1 | UnannotatedRegion |
| 122 | AAATTTGCGG.ATCCGCCGCA | C | x | 35010_12_17186_XS2_1 | UnannotatedRegion |
| 122 | AAATTTGCGG.ATCCGCCGCA | C | x | 35011_12_17194_XS2_1 | UnannotatedRegion |
| 122 | AAATTTGCGG.ATCCGCCGCA | C | x | 35012_12_17009_XS2_1 | UnannotatedRegion |
| 122 | AAATTTGCGG.ATCCGCCGCA | C | x | 35013_12_14973_XS2_1 | UnannotatedRegion |
|  |  |  |  |  |  |
| 128 | AACACATTCC.GATTCGTATT | A | 856497 F | 19260_WUE_2594 | UnannotatedRegion |
| 128 | AACACATTCC.GATTCGTATT | G | x | 34990_Tchad54_11 | UnannotatedRegion |
| 128 | AACACATTCC.GATTCGTATT | G | x | 34991_Tchad78_11 | UnannotatedRegion |
| 128 | AACACATTCC.GATTCGTATT | G | x | 34992_Tchad95_11 | UnannotatedRegion |
| 128 | AACACATTCC.GATTCGTATT | G | x | 34994_Tchad106_11 | UnannotatedRegion |
| 128 | AACACATTCC.GATTCGTATT | G | x | 34995_8_2011 | UnannotatedRegion |
| 128 | AACACATTCC.GATTCGTATT | G | x | 34996_10_2011 | UnannotatedRegion |
| 128 | AACACATTCC.GATTCGTATT | G | x | 34997_39_2011 | UnannotatedRegion |
| 128 | AACACATTCC.GATTCGTATT | G | x | 34998_59_2011 | UnannotatedRegion |
| 128 | AACACATTCC.GATTCGTATT | G | x | 34999_63_2011 | UnannotatedRegion |
| 128 | AACACATTCC.GATTCGTATT | G | x | 35000_75_2011 | UnannotatedRegion |
| 128 | AACACATTCC.GATTCGTATT | G | x | 35001_76_2011 | UnannotatedRegion |
| 128 | AACACATTCC.GATTCGTATT | G | x | 35002_120_2011 | UnannotatedRegion |
| 128 | AACACATTCC.GATTCGTATT | G | x | 35003_403_2011 | UnannotatedRegion |
| 128 | AACACATTCC.GATTCGTATT | G | x | 35004_12_15398_XS2_1 | UnannotatedRegion |
| 128 | AACACATTCC.GATTCGTATT | G | x | 35005_12_13952_XS2_1 | UnannotatedRegion |
| 128 | AACACATTCC.GATTCGTATT | G | x | 35006_12_15317_XS2_1 | UnannotatedRegion |
| 128 | AACACATTCC.GATTCGTATT | G | x | 35007_12_15047_XS2_1 | UnannotatedRegion |
| 128 | AACACATTCC.GATTCGTATT | G | x | 35008_12_15661_XS2_1 | UnannotatedRegion |
| 128 | AACACATTCC.GATTCGTATT | G | x | 35009_12_15657_XS2_1 | UnannotatedRegion |
| 128 | AACACATTCC.GATTCGTATT | G | x | 35010_12_17186_XS2_1 | UnannotatedRegion |
| 128 | AACACATTCC.GATTCGTATT | G | x | 35011_12_17194_XS2_1 | UnannotatedRegion |
| 128 | AACACATTCC.GATTCGTATT | G | x | 35012_12_17009_XS2_1 | UnannotatedRegion |
| 128 | AACACATTCC.GATTCGTATT | G | x | 35013_12_14973_XS2_1 | UnannotatedRegion |
|  |  |  |  |  |  |
| 139 | AACATTTGTT.CAGACGGCAT | T | 1119784 R | 19260_WUE_2594 | UnannotatedRegion |
| 139 | AACATTTGTT.CAGACGGCAT | C | x | 34990_Tchad54_11 | UnannotatedRegion |
| 139 | AACATTTGTT.CAGACGGCAT | C | x | 34991_Tchad78_11 | UnannotatedRegion |
| 139 | AACATTTGTT.CAGACGGCAT | C | x | 34992_Tchad95_11 | UnannotatedRegion |
| 139 | AACATTTGTT.CAGACGGCAT | C | x | 34994_Tchad106_11 | UnannotatedRegion |
| 139 | AACATTTGTT.CAGACGGCAT | C | x | 34995_8_2011 | UnannotatedRegion |
| 139 | AACATTTGTT.CAGACGGCAT | C | x | 34996_10_2011 | UnannotatedRegion |
| 139 | AACATTTGTT.CAGACGGCAT | C | x | 34997_39_2011 | UnannotatedRegion |
| 139 | AACATTTGTT.CAGACGGCAT | C | x | 34998_59_2011 | UnannotatedRegion |
| 139 | AACATTTGTT.CAGACGGCAT | C | x | 34999_63_2011 | UnannotatedRegion |
| 139 | AACATTTGTT.CAGACGGCAT | C | x | 35000_75_2011 | UnannotatedRegion |
| 139 | AACATTTGTT.CAGACGGCAT | C | x | 35001_76_2011 | UnannotatedRegion |
| 139 | AACATTTGTT.CAGACGGCAT | C | x | 35002_120_2011 | UnannotatedRegion |
| 139 | AACATTTGTT.CAGACGGCAT | C | x | 35003_403_2011 | UnannotatedRegion |
| 139 | AACATTTGTT.CAGACGGCAT | C | x | 35004_12_15398_XS2_1 | UnannotatedRegion |
| 139 | AACATTTGTT.CAGACGGCAT | C | x | 35005_12_13952_XS2_1 | UnannotatedRegion |
| 139 | AACATTTGTT.CAGACGGCAT | C | x | 35006_12_15317_XS2_1 | UnannotatedRegion |
| 139 | AACATTTGTT.CAGACGGCAT | C | x | 35007_12_15047_XS2_1 | UnannotatedRegion |
| 139 | AACATTTGTT.CAGACGGCAT | C | x | 35008_12_15661_XS2_1 | UnannotatedRegion |
| 139 | AACATTTGTT.CAGACGGCAT | C | x | 35009_12_15657_XS2_1 | UnannotatedRegion |
| 139 | AACATTTGTT.CAGACGGCAT | C | x | 35010_12_17186_XS2_1 | UnannotatedRegion |
| 139 | AACATTTGTT.CAGACGGCAT | C | x | 35011_12_17194_XS2_1 | UnannotatedRegion |
| 139 | AACATTTGTT.CAGACGGCAT | C | x | 35012_12_17009_XS2_1 | UnannotatedRegion |
| 139 | AACATTTGTT.CAGACGGCAT | C | x | 35013_12_14973_XS2_1 | UnannotatedRegion |
|  |  |  |  |  |  |
| 151 | AACCGATTTT.TTGCCTTAAA | A | 739707 R | 19260_WUE_2594 | UnannotatedRegion |
| 151 | AACCGATTTT.TTGCCTTAAA | G | x | 34990_Tchad54_11 | UnannotatedRegion |
| 151 | AACCGATTTT.TTGCCTTAAA | G | x | 34991_Tchad78_11 | UnannotatedRegion |
| 151 | AACCGATTTT.TTGCCTTAAA | G | x | 34992_Tchad95_11 | UnannotatedRegion |
| 151 | AACCGATTTT.TTGCCTTAAA | G | x | 34994_Tchad106_11 | UnannotatedRegion |
| 151 | AACCGATTTT.TTGCCTTAAA | G | x | 34995_8_2011 | UnannotatedRegion |
| 151 | AACCGATTTT.TTGCCTTAAA | G | x | 34996_10_2011 | UnannotatedRegion |
| 151 | AACCGATTTT.TTGCCTTAAA | G | x | 34997_39_2011 | UnannotatedRegion |
| 151 | AACCGATTTT.TTGCCTTAAA | G | x | 34998_59_2011 | UnannotatedRegion |
| 151 | AACCGATTTT.TTGCCTTAAA | G | x | 34999_63_2011 | UnannotatedRegion |
| 151 | AACCGATTTT.TTGCCTTAAA | G | x | 35000_75_2011 | UnannotatedRegion |
| 151 | AACCGATTTT.TTGCCTTAAA | G | x | 35001_76_2011 | UnannotatedRegion |
| 151 | AACCGATTTT.TTGCCTTAAA | G | x | 35002_120_2011 | UnannotatedRegion |
| 151 | AACCGATTTT.TTGCCTTAAA | G | x | 35003_403_2011 | UnannotatedRegion |
| 151 | AACCGATTTT.TTGCCTTAAA | G | x | 35004_12_15398_XS2_1 | UnannotatedRegion |
| 151 | AACCGATTTT.TTGCCTTAAA | G | x | 35005_12_13952_XS2_1 | UnannotatedRegion |
| 151 | AACCGATTTT.TTGCCTTAAA | G | x | 35006_12_15317_XS2_1 | UnannotatedRegion |
| 151 | AACCGATTTT.TTGCCTTAAA | G | x | 35007_12_15047_XS2_1 | UnannotatedRegion |
| 151 | AACCGATTTT.TTGCCTTAAA | G | x | 35008_12_15661_XS2_1 | UnannotatedRegion |
| 151 | AACCGATTTT.TTGCCTTAAA | G | x | 35009_12_15657_XS2_1 | UnannotatedRegion |
| 151 | AACCGATTTT.TTGCCTTAAA | G | x | 35010_12_17186_XS2_1 | UnannotatedRegion |
| 151 | AACCGATTTT.TTGCCTTAAA | G | x | 35011_12_17194_XS2_1 | UnannotatedRegion |
| 151 | AACCGATTTT.TTGCCTTAAA | G | x | 35012_12_17009_XS2_1 | UnannotatedRegion |
| 151 | AACCGATTTT.TTGCCTTAAA | G | x | 35013_12_14973_XS2_1 | UnannotatedRegion |
|  |  |  |  |  |  |
| 156 | AACCTGAGCC.TTTGTAACAC | C | 2160902 R | 19260_WUE_2594 | UnannotatedRegion |
| 156 | AACCTGAGCC.TTTGTAACAC | T | x | 34990_Tchad54_11 | UnannotatedRegion |
| 156 | AACCTGAGCC.TTTGTAACAC | T | x | 34991_Tchad78_11 | UnannotatedRegion |
| 156 | AACCTGAGCC.TTTGTAACAC | C | x | 34992_Tchad95_11 | UnannotatedRegion |
| 156 | AACCTGAGCC.TTTGTAACAC | C | x | 34994_Tchad106_11 | UnannotatedRegion |
| 156 | AACCTGAGCC.TTTGTAACAC | C | x | 34995_8_2011 | UnannotatedRegion |
| 156 | AACCTGAGCC.TTTGTAACAC | C | x | 34996_10_2011 | UnannotatedRegion |
| 156 | AACCTGAGCC.TTTGTAACAC | T | x | 34997_39_2011 | UnannotatedRegion |
| 156 | AACCTGAGCC.TTTGTAACAC | T | x | 34998_59_2011 | UnannotatedRegion |
| 156 | AACCTGAGCC.TTTGTAACAC | T | x | 34999_63_2011 | UnannotatedRegion |
| 156 | AACCTGAGCC.TTTGTAACAC | T | x | 35000_75_2011 | UnannotatedRegion |
| 156 | AACCTGAGCC.TTTGTAACAC | T | x | 35001_76_2011 | UnannotatedRegion |
| 156 | AACCTGAGCC.TTTGTAACAC | T | x | 35002_120_2011 | UnannotatedRegion |
| 156 | AACCTGAGCC.TTTGTAACAC | T | x | 35003_403_2011 | UnannotatedRegion |
| 156 | AACCTGAGCC.TTTGTAACAC | T | x | 35004_12_15398_XS2_1 | UnannotatedRegion |
| 156 | AACCTGAGCC.TTTGTAACAC | T | x | 35005_12_13952_XS2_1 | UnannotatedRegion |
| 156 | AACCTGAGCC.TTTGTAACAC | T | x | 35006_12_15317_XS2_1 | UnannotatedRegion |
| 156 | AACCTGAGCC.TTTGTAACAC | C | x | 35007_12_15047_XS2_1 | UnannotatedRegion |
| 156 | AACCTGAGCC.TTTGTAACAC | C | x | 35008_12_15661_XS2_1 | UnannotatedRegion |
| 156 | AACCTGAGCC.TTTGTAACAC | C | x | 35009_12_15657_XS2_1 | UnannotatedRegion |
| 156 | AACCTGAGCC.TTTGTAACAC | T | x | 35010_12_17186_XS2_1 | UnannotatedRegion |
| 156 | AACCTGAGCC.TTTGTAACAC | T | x | 35011_12_17194_XS2_1 | UnannotatedRegion |
| 156 | AACCTGAGCC.TTTGTAACAC | T | x | 35012_12_17009_XS2_1 | UnannotatedRegion |
| 156 | AACCTGAGCC.TTTGTAACAC | C | x | 35013_12_14973_XS2_1 | UnannotatedRegion |
|  |  |  |  |  |  |
| 157 | AACCTGCACG.TTCGGCGTTC | A | 2073237 F | 19260_WUE_2594 | UnannotatedRegion |
| 157 | AACCTGCACG.TTCGGCGTTC | G | x | 34990_Tchad54_11 | UnannotatedRegion |
| 157 | AACCTGCACG.TTCGGCGTTC | G | x | 34991_Tchad78_11 | UnannotatedRegion |
| 157 | AACCTGCACG.TTCGGCGTTC | G | x | 34992_Tchad95_11 | UnannotatedRegion |
| 157 | AACCTGCACG.TTCGGCGTTC | G | x | 34994_Tchad106_11 | UnannotatedRegion |
| 157 | AACCTGCACG.TTCGGCGTTC | G | x | 34995_8_2011 | UnannotatedRegion |
| 157 | AACCTGCACG.TTCGGCGTTC | G | x | 34996_10_2011 | UnannotatedRegion |
| 157 | AACCTGCACG.TTCGGCGTTC | G | x | 34997_39_2011 | UnannotatedRegion |
| 157 | AACCTGCACG.TTCGGCGTTC | G | x | 34998_59_2011 | UnannotatedRegion |
| 157 | AACCTGCACG.TTCGGCGTTC | G | x | 34999_63_2011 | UnannotatedRegion |
| 157 | AACCTGCACG.TTCGGCGTTC | G | x | 35000_75_2011 | UnannotatedRegion |
| 157 | AACCTGCACG.TTCGGCGTTC | G | x | 35001_76_2011 | UnannotatedRegion |
| 157 | AACCTGCACG.TTCGGCGTTC | G | x | 35002_120_2011 | UnannotatedRegion |
| 157 | AACCTGCACG.TTCGGCGTTC | G | x | 35003_403_2011 | UnannotatedRegion |
| 157 | AACCTGCACG.TTCGGCGTTC | G | x | 35004_12_15398_XS2_1 | UnannotatedRegion |
| 157 | AACCTGCACG.TTCGGCGTTC | G | x | 35005_12_13952_XS2_1 | UnannotatedRegion |
| 157 | AACCTGCACG.TTCGGCGTTC | G | x | 35006_12_15317_XS2_1 | UnannotatedRegion |
| 157 | AACCTGCACG.TTCGGCGTTC | G | x | 35007_12_15047_XS2_1 | UnannotatedRegion |
| 157 | AACCTGCACG.TTCGGCGTTC | G | x | 35008_12_15661_XS2_1 | UnannotatedRegion |
| 157 | AACCTGCACG.TTCGGCGTTC | G | x | 35009_12_15657_XS2_1 | UnannotatedRegion |
| 157 | AACCTGCACG.TTCGGCGTTC | G | x | 35010_12_17186_XS2_1 | UnannotatedRegion |
| 157 | AACCTGCACG.TTCGGCGTTC | G | x | 35011_12_17194_XS2_1 | UnannotatedRegion |
| 157 | AACCTGCACG.TTCGGCGTTC | G | x | 35012_12_17009_XS2_1 | UnannotatedRegion |
| 157 | AACCTGCACG.TTCGGCGTTC | G | x | 35013_12_14973_XS2_1 | UnannotatedRegion |
|  |  |  |  |  |  |
| 159 | AACCTGTGTT.GGGTTTCGGA | T | 460329 R | 19260_WUE_2594 | UnannotatedRegion |
| 159 | AACCTGTGTT.GGGTTTCGGA | G | x | 34990_Tchad54_11 | UnannotatedRegion |
| 159 | AACCTGTGTT.GGGTTTCGGA | G | x | 34991_Tchad78_11 | UnannotatedRegion |
| 159 | AACCTGTGTT.GGGTTTCGGA | G | x | 34992_Tchad95_11 | UnannotatedRegion |
| 159 | AACCTGTGTT.GGGTTTCGGA | G | x | 34994_Tchad106_11 | UnannotatedRegion |
| 159 | AACCTGTGTT.GGGTTTCGGA | G | x | 34995_8_2011 | UnannotatedRegion |
| 159 | AACCTGTGTT.GGGTTTCGGA | G | x | 34996_10_2011 | UnannotatedRegion |
| 159 | AACCTGTGTT.GGGTTTCGGA | G | x | 34997_39_2011 | UnannotatedRegion |
| 159 | AACCTGTGTT.GGGTTTCGGA | G | x | 34998_59_2011 | UnannotatedRegion |
| 159 | AACCTGTGTT.GGGTTTCGGA | G | x | 34999_63_2011 | UnannotatedRegion |
| 159 | AACCTGTGTT.GGGTTTCGGA | G | x | 35000_75_2011 | UnannotatedRegion |
| 159 | AACCTGTGTT.GGGTTTCGGA | G | x | 35001_76_2011 | UnannotatedRegion |
| 159 | AACCTGTGTT.GGGTTTCGGA | G | x | 35002_120_2011 | UnannotatedRegion |
| 159 | AACCTGTGTT.GGGTTTCGGA | G | x | 35003_403_2011 | UnannotatedRegion |
| 159 | AACCTGTGTT.GGGTTTCGGA | G | x | 35004_12_15398_XS2_1 | UnannotatedRegion |
| 159 | AACCTGTGTT.GGGTTTCGGA | G | x | 35005_12_13952_XS2_1 | UnannotatedRegion |
| 159 | AACCTGTGTT.GGGTTTCGGA | G | x | 35006_12_15317_XS2_1 | UnannotatedRegion |
| 159 | AACCTGTGTT.GGGTTTCGGA | G | x | 35007_12_15047_XS2_1 | UnannotatedRegion |
| 159 | AACCTGTGTT.GGGTTTCGGA | G | x | 35008_12_15661_XS2_1 | UnannotatedRegion |
| 159 | AACCTGTGTT.GGGTTTCGGA | G | x | 35009_12_15657_XS2_1 | UnannotatedRegion |
| 159 | AACCTGTGTT.GGGTTTCGGA | G | x | 35010_12_17186_XS2_1 | UnannotatedRegion |
| 159 | AACCTGTGTT.GGGTTTCGGA | G | x | 35011_12_17194_XS2_1 | UnannotatedRegion |
| 159 | AACCTGTGTT.GGGTTTCGGA | G | x | 35012_12_17009_XS2_1 | UnannotatedRegion |
| 159 | AACCTGTGTT.GGGTTTCGGA | G | x | 35013_12_14973_XS2_1 | UnannotatedRegion |
|  |  |  |  |  |  |
| 174 | AACGGTCAAT.CTTTTCTGAA | A | 154821 R | 19260_WUE_2594 | UnannotatedRegion |
| 174 | AACGGTCAAT.CTTTTCTGAA | G | x | 34990_Tchad54_11 | UnannotatedRegion |
| 174 | AACGGTCAAT.CTTTTCTGAA | G | x | 34991_Tchad78_11 | UnannotatedRegion |
| 174 | AACGGTCAAT.CTTTTCTGAA | G | x | 34992_Tchad95_11 | UnannotatedRegion |
| 174 | AACGGTCAAT.CTTTTCTGAA | G | x | 34994_Tchad106_11 | UnannotatedRegion |
| 174 | AACGGTCAAT.CTTTTCTGAA | A | x | 34995_8_2011 | UnannotatedRegion |
| 174 | AACGGTCAAT.CTTTTCTGAA | A | x | 34996_10_2011 | UnannotatedRegion |
| 174 | AACGGTCAAT.CTTTTCTGAA | G | x | 34997_39_2011 | UnannotatedRegion |
| 174 | AACGGTCAAT.CTTTTCTGAA | G | x | 34998_59_2011 | UnannotatedRegion |
| 174 | AACGGTCAAT.CTTTTCTGAA | G | x | 34999_63_2011 | UnannotatedRegion |
| 174 | AACGGTCAAT.CTTTTCTGAA | G | x | 35000_75_2011 | UnannotatedRegion |
| 174 | AACGGTCAAT.CTTTTCTGAA | G | x | 35001_76_2011 | UnannotatedRegion |
| 174 | AACGGTCAAT.CTTTTCTGAA | G | x | 35002_120_2011 | UnannotatedRegion |
| 174 | AACGGTCAAT.CTTTTCTGAA | G | x | 35003_403_2011 | UnannotatedRegion |
| 174 | AACGGTCAAT.CTTTTCTGAA | G | x | 35004_12_15398_XS2_1 | UnannotatedRegion |
| 174 | AACGGTCAAT.CTTTTCTGAA | G | x | 35005_12_13952_XS2_1 | UnannotatedRegion |
| 174 | AACGGTCAAT.CTTTTCTGAA | G | x | 35006_12_15317_XS2_1 | UnannotatedRegion |
| 174 | AACGGTCAAT.CTTTTCTGAA | G | x | 35007_12_15047_XS2_1 | UnannotatedRegion |
| 174 | AACGGTCAAT.CTTTTCTGAA | G | x | 35008_12_15661_XS2_1 | UnannotatedRegion |
| 174 | AACGGTCAAT.CTTTTCTGAA | G | x | 35009_12_15657_XS2_1 | UnannotatedRegion |
| 174 | AACGGTCAAT.CTTTTCTGAA | G | x | 35010_12_17186_XS2_1 | UnannotatedRegion |
| 174 | AACGGTCAAT.CTTTTCTGAA | G | x | 35011_12_17194_XS2_1 | UnannotatedRegion |
| 174 | AACGGTCAAT.CTTTTCTGAA | G | x | 35012_12_17009_XS2_1 | UnannotatedRegion |
| 174 | AACGGTCAAT.CTTTTCTGAA | G | x | 35013_12_14973_XS2_1 | UnannotatedRegion |
|  |  |  |  |  |  |
| 178 | AACTATTACA.AAAAAACAAA | T | 2162363 R | 19260_WUE_2594 | UnannotatedRegion |
| 178 | AACTATTACA.AAAAAACAAA | T | x | 34990_Tchad54_11 | UnannotatedRegion |
| 178 | AACTATTACA.AAAAAACAAA | T | x | 34991_Tchad78_11 | UnannotatedRegion |
| 178 | AACTATTACA.AAAAAACAAA | C | x | 34992_Tchad95_11 | UnannotatedRegion |
| 178 | AACTATTACA.AAAAAACAAA | C | x | 34994_Tchad106_11 | UnannotatedRegion |
| 178 | AACTATTACA.AAAAAACAAA | T | x | 34995_8_2011 | UnannotatedRegion |
| 178 | AACTATTACA.AAAAAACAAA | T | x | 34996_10_2011 | UnannotatedRegion |
| 178 | AACTATTACA.AAAAAACAAA | T | x | 34997_39_2011 | UnannotatedRegion |
| 178 | AACTATTACA.AAAAAACAAA | T | x | 34998_59_2011 | UnannotatedRegion |
| 178 | AACTATTACA.AAAAAACAAA | T | x | 34999_63_2011 | UnannotatedRegion |
| 178 | AACTATTACA.AAAAAACAAA | T | x | 35000_75_2011 | UnannotatedRegion |
| 178 | AACTATTACA.AAAAAACAAA | T | x | 35001_76_2011 | UnannotatedRegion |
| 178 | AACTATTACA.AAAAAACAAA | T | x | 35002_120_2011 | UnannotatedRegion |
| 178 | AACTATTACA.AAAAAACAAA | T | x | 35003_403_2011 | UnannotatedRegion |
| 178 | AACTATTACA.AAAAAACAAA | T | x | 35004_12_15398_XS2_1 | UnannotatedRegion |
| 178 | AACTATTACA.AAAAAACAAA | T | x | 35005_12_13952_XS2_1 | UnannotatedRegion |
| 178 | AACTATTACA.AAAAAACAAA | T | x | 35006_12_15317_XS2_1 | UnannotatedRegion |
| 178 | AACTATTACA.AAAAAACAAA | C | x | 35007_12_15047_XS2_1 | UnannotatedRegion |
| 178 | AACTATTACA.AAAAAACAAA | C | x | 35008_12_15661_XS2_1 | UnannotatedRegion |
| 178 | AACTATTACA.AAAAAACAAA | C | x | 35009_12_15657_XS2_1 | UnannotatedRegion |
| 178 | AACTATTACA.AAAAAACAAA | T | x | 35010_12_17186_XS2_1 | UnannotatedRegion |
| 178 | AACTATTACA.AAAAAACAAA | T | x | 35011_12_17194_XS2_1 | UnannotatedRegion |
| 178 | AACTATTACA.AAAAAACAAA | T | x | 35012_12_17009_XS2_1 | UnannotatedRegion |
| 178 | AACTATTACA.AAAAAACAAA | C | x | 35013_12_14973_XS2_1 | UnannotatedRegion |
|  |  |  |  |  |  |
| 182 | AACTCCTTCG.ATGTTGCCGC | A | 2079946 R | 19260_WUE_2594 | UnannotatedRegion |
| 182 | AACTCCTTCG.ATGTTGCCGC | A | x | 34990_Tchad54_11 | UnannotatedRegion |
| 182 | AACTCCTTCG.ATGTTGCCGC | A | x | 34991_Tchad78_11 | UnannotatedRegion |
| 182 | AACTCCTTCG.ATGTTGCCGC | A | x | 34992_Tchad95_11 | UnannotatedRegion |
| 182 | AACTCCTTCG.ATGTTGCCGC | A | x | 34994_Tchad106_11 | UnannotatedRegion |
| 182 | AACTCCTTCG.ATGTTGCCGC | G | x | 34995_8_2011 | UnannotatedRegion |
| 182 | AACTCCTTCG.ATGTTGCCGC | G | x | 34996_10_2011 | UnannotatedRegion |
| 182 | AACTCCTTCG.ATGTTGCCGC | A | x | 34997_39_2011 | UnannotatedRegion |
| 182 | AACTCCTTCG.ATGTTGCCGC | A | x | 34998_59_2011 | UnannotatedRegion |
| 182 | AACTCCTTCG.ATGTTGCCGC | A | x | 34999_63_2011 | UnannotatedRegion |
| 182 | AACTCCTTCG.ATGTTGCCGC | A | x | 35000_75_2011 | UnannotatedRegion |
| 182 | AACTCCTTCG.ATGTTGCCGC | A | x | 35001_76_2011 | UnannotatedRegion |
| 182 | AACTCCTTCG.ATGTTGCCGC | A | x | 35002_120_2011 | UnannotatedRegion |
| 182 | AACTCCTTCG.ATGTTGCCGC | A | x | 35003_403_2011 | UnannotatedRegion |
| 182 | AACTCCTTCG.ATGTTGCCGC | A | x | 35004_12_15398_XS2_1 | UnannotatedRegion |
| 182 | AACTCCTTCG.ATGTTGCCGC | A | x | 35005_12_13952_XS2_1 | UnannotatedRegion |
| 182 | AACTCCTTCG.ATGTTGCCGC | A | x | 35006_12_15317_XS2_1 | UnannotatedRegion |
| 182 | AACTCCTTCG.ATGTTGCCGC | A | x | 35007_12_15047_XS2_1 | UnannotatedRegion |
| 182 | AACTCCTTCG.ATGTTGCCGC | A | x | 35008_12_15661_XS2_1 | UnannotatedRegion |
| 182 | AACTCCTTCG.ATGTTGCCGC | A | x | 35009_12_15657_XS2_1 | UnannotatedRegion |
| 182 | AACTCCTTCG.ATGTTGCCGC | A | x | 35010_12_17186_XS2_1 | UnannotatedRegion |
| 182 | AACTCCTTCG.ATGTTGCCGC | A | x | 35011_12_17194_XS2_1 | UnannotatedRegion |
| 182 | AACTCCTTCG.ATGTTGCCGC | A | x | 35012_12_17009_XS2_1 | UnannotatedRegion |
| 182 | AACTCCTTCG.ATGTTGCCGC | A | x | 35013_12_14973_XS2_1 | UnannotatedRegion |
|  |  |  |  |  |  |
| 215 | AAGCAGAAAT.TCAAAACAAT | G | 19607 R | 19260_WUE_2594 | UnannotatedRegion |
| 215 | AAGCAGAAAT.TCAAAACAAT | A | x | 34990_Tchad54_11 | UnannotatedRegion |
| 215 | AAGCAGAAAT.TCAAAACAAT | A | x | 34991_Tchad78_11 | UnannotatedRegion |
| 215 | AAGCAGAAAT.TCAAAACAAT | A | x | 34992_Tchad95_11 | UnannotatedRegion |
| 215 | AAGCAGAAAT.TCAAAACAAT | A | x | 34994_Tchad106_11 | UnannotatedRegion |
| 215 | AAGCAGAAAT.TCAAAACAAT | A | x | 34995_8_2011 | UnannotatedRegion |
| 215 | AAGCAGAAAT.TCAAAACAAT | A | x | 34996_10_2011 | UnannotatedRegion |
| 215 | AAGCAGAAAT.TCAAAACAAT | A | x | 34997_39_2011 | UnannotatedRegion |
| 215 | AAGCAGAAAT.TCAAAACAAT | A | x | 34998_59_2011 | UnannotatedRegion |
| 215 | AAGCAGAAAT.TCAAAACAAT | A | x | 34999_63_2011 | UnannotatedRegion |
| 215 | AAGCAGAAAT.TCAAAACAAT | A | x | 35000_75_2011 | UnannotatedRegion |
| 215 | AAGCAGAAAT.TCAAAACAAT | A | x | 35001_76_2011 | UnannotatedRegion |
| 215 | AAGCAGAAAT.TCAAAACAAT | A | x | 35002_120_2011 | UnannotatedRegion |
| 215 | AAGCAGAAAT.TCAAAACAAT | A | x | 35003_403_2011 | UnannotatedRegion |
| 215 | AAGCAGAAAT.TCAAAACAAT | A | x | 35004_12_15398_XS2_1 | UnannotatedRegion |
| 215 | AAGCAGAAAT.TCAAAACAAT | A | x | 35005_12_13952_XS2_1 | UnannotatedRegion |
| 215 | AAGCAGAAAT.TCAAAACAAT | A | x | 35006_12_15317_XS2_1 | UnannotatedRegion |
| 215 | AAGCAGAAAT.TCAAAACAAT | A | x | 35007_12_15047_XS2_1 | UnannotatedRegion |
| 215 | AAGCAGAAAT.TCAAAACAAT | A | x | 35008_12_15661_XS2_1 | UnannotatedRegion |
| 215 | AAGCAGAAAT.TCAAAACAAT | A | x | 35009_12_15657_XS2_1 | UnannotatedRegion |
| 215 | AAGCAGAAAT.TCAAAACAAT | A | x | 35010_12_17186_XS2_1 | UnannotatedRegion |
| 215 | AAGCAGAAAT.TCAAAACAAT | A | x | 35011_12_17194_XS2_1 | UnannotatedRegion |
| 215 | AAGCAGAAAT.TCAAAACAAT | A | x | 35012_12_17009_XS2_1 | UnannotatedRegion |
| 215 | AAGCAGAAAT.TCAAAACAAT | A | x | 35013_12_14973_XS2_1 | UnannotatedRegion |
|  |  |  |  |  |  |
| 222 | AAGCCCCTCC.GCCCTTCAGA | T | 1147159 F | 19260_WUE_2594 | UnannotatedRegion |
| 222 | AAGCCCCTCC.GCCCTTCAGA | C | x | 34990_Tchad54_11 | UnannotatedRegion |
| 222 | AAGCCCCTCC.GCCCTTCAGA | C | x | 34991_Tchad78_11 | UnannotatedRegion |
| 222 | AAGCCCCTCC.GCCCTTCAGA | C | x | 34992_Tchad95_11 | UnannotatedRegion |
| 222 | AAGCCCCTCC.GCCCTTCAGA | C | x | 34994_Tchad106_11 | UnannotatedRegion |
| 222 | AAGCCCCTCC.GCCCTTCAGA | C | x | 34995_8_2011 | UnannotatedRegion |
| 222 | AAGCCCCTCC.GCCCTTCAGA | C | x | 34996_10_2011 | UnannotatedRegion |
| 222 | AAGCCCCTCC.GCCCTTCAGA | C | x | 34997_39_2011 | UnannotatedRegion |
| 222 | AAGCCCCTCC.GCCCTTCAGA | C | x | 34998_59_2011 | UnannotatedRegion |
| 222 | AAGCCCCTCC.GCCCTTCAGA | C | x | 34999_63_2011 | UnannotatedRegion |
| 222 | AAGCCCCTCC.GCCCTTCAGA | C | x | 35000_75_2011 | UnannotatedRegion |
| 222 | AAGCCCCTCC.GCCCTTCAGA | C | x | 35001_76_2011 | UnannotatedRegion |
| 222 | AAGCCCCTCC.GCCCTTCAGA | C | x | 35002_120_2011 | UnannotatedRegion |
| 222 | AAGCCCCTCC.GCCCTTCAGA | C | x | 35003_403_2011 | UnannotatedRegion |
| 222 | AAGCCCCTCC.GCCCTTCAGA | C | x | 35004_12_15398_XS2_1 | UnannotatedRegion |
| 222 | AAGCCCCTCC.GCCCTTCAGA | C | x | 35005_12_13952_XS2_1 | UnannotatedRegion |
| 222 | AAGCCCCTCC.GCCCTTCAGA | C | x | 35006_12_15317_XS2_1 | UnannotatedRegion |
| 222 | AAGCCCCTCC.GCCCTTCAGA | C | x | 35007_12_15047_XS2_1 | UnannotatedRegion |
| 222 | AAGCCCCTCC.GCCCTTCAGA | C | x | 35008_12_15661_XS2_1 | UnannotatedRegion |
| 222 | AAGCCCCTCC.GCCCTTCAGA | C | x | 35009_12_15657_XS2_1 | UnannotatedRegion |
| 222 | AAGCCCCTCC.GCCCTTCAGA | C | x | 35010_12_17186_XS2_1 | UnannotatedRegion |
| 222 | AAGCCCCTCC.GCCCTTCAGA | C | x | 35011_12_17194_XS2_1 | UnannotatedRegion |
| 222 | AAGCCCCTCC.GCCCTTCAGA | C | x | 35012_12_17009_XS2_1 | UnannotatedRegion |
| 222 | AAGCCCCTCC.GCCCTTCAGA | C | x | 35013_12_14973_XS2_1 | UnannotatedRegion |
|  |  |  |  |  |  |
| 232 | AAGCGGCGTG.CAAAACGGGG | C | 2037439 R | 19260_WUE_2594 | UnannotatedRegion |
| 232 | AAGCGGCGTG.CAAAACGGGG | C | x | 34990_Tchad54_11 | UnannotatedRegion |
| 232 | AAGCGGCGTG.CAAAACGGGG | C | x | 34991_Tchad78_11 | UnannotatedRegion |
| 232 | AAGCGGCGTG.CAAAACGGGG | C | x | 34992_Tchad95_11 | UnannotatedRegion |
| 232 | AAGCGGCGTG.CAAAACGGGG | C | x | 34995_8_2011 | UnannotatedRegion |
| 232 | AAGCGGCGTG.CAAAACGGGG | C | x | 34996_10_2011 | UnannotatedRegion |
| 232 | AAGCGGCGTG.CAAAACGGGG | C | x | 34997_39_2011 | UnannotatedRegion |
| 232 | AAGCGGCGTG.CAAAACGGGG | C | x | 34998_59_2011 | UnannotatedRegion |
| 232 | AAGCGGCGTG.CAAAACGGGG | C | x | 34999_63_2011 | UnannotatedRegion |
| 232 | AAGCGGCGTG.CAAAACGGGG | C | x | 35000_75_2011 | UnannotatedRegion |
| 232 | AAGCGGCGTG.CAAAACGGGG | C | x | 35001_76_2011 | UnannotatedRegion |
| 232 | AAGCGGCGTG.CAAAACGGGG | C | x | 35002_120_2011 | UnannotatedRegion |
| 232 | AAGCGGCGTG.CAAAACGGGG | C | x | 35003_403_2011 | UnannotatedRegion |
| 232 | AAGCGGCGTG.CAAAACGGGG | C | x | 35004_12_15398_XS2_1 | UnannotatedRegion |
| 232 | AAGCGGCGTG.CAAAACGGGG | C | x | 35005_12_13952_XS2_1 | UnannotatedRegion |
| 232 | AAGCGGCGTG.CAAAACGGGG | C | x | 35006_12_15317_XS2_1 | UnannotatedRegion |
| 232 | AAGCGGCGTG.CAAAACGGGG | C | x | 35007_12_15047_XS2_1 | UnannotatedRegion |
| 232 | AAGCGGCGTG.CAAAACGGGG | C | x | 35009_12_15657_XS2_1 | UnannotatedRegion |
| 232 | AAGCGGCGTG.CAAAACGGGG | C | x | 35010_12_17186_XS2_1 | UnannotatedRegion |
| 232 | AAGCGGCGTG.CAAAACGGGG | T | x | 35011_12_17194_XS2_1 | UnannotatedRegion |
| 232 | AAGCGGCGTG.CAAAACGGGG | C | x | 35012_12_17009_XS2_1 | UnannotatedRegion |
| 232 | AAGCGGCGTG.CAAAACGGGG | C | x | 35013_12_14973_XS2_1 | UnannotatedRegion |
|  |  |  |  |  |  |
| 246 | AAGGGCAGGG.GTCAGCGTTG | G | 856769 F | 19260_WUE_2594 | UnannotatedRegion |
| 246 | AAGGGCAGGG.GTCAGCGTTG | A | x | 34990_Tchad54_11 | UnannotatedRegion |
| 246 | AAGGGCAGGG.GTCAGCGTTG | A | x | 34991_Tchad78_11 | UnannotatedRegion |
| 246 | AAGGGCAGGG.GTCAGCGTTG | A | x | 34992_Tchad95_11 | UnannotatedRegion |
| 246 | AAGGGCAGGG.GTCAGCGTTG | A | x | 34995_8_2011 | UnannotatedRegion |
| 246 | AAGGGCAGGG.GTCAGCGTTG | A | x | 34996_10_2011 | UnannotatedRegion |
| 246 | AAGGGCAGGG.GTCAGCGTTG | A | x | 34997_39_2011 | UnannotatedRegion |
| 246 | AAGGGCAGGG.GTCAGCGTTG | A | x | 34998_59_2011 | UnannotatedRegion |
| 246 | AAGGGCAGGG.GTCAGCGTTG | A | x | 34999_63_2011 | UnannotatedRegion |
| 246 | AAGGGCAGGG.GTCAGCGTTG | A | x | 35000_75_2011 | UnannotatedRegion |
| 246 | AAGGGCAGGG.GTCAGCGTTG | A | x | 35001_76_2011 | UnannotatedRegion |
| 246 | AAGGGCAGGG.GTCAGCGTTG | A | x | 35002_120_2011 | UnannotatedRegion |
| 246 | AAGGGCAGGG.GTCAGCGTTG | A | x | 35003_403_2011 | UnannotatedRegion |
| 246 | AAGGGCAGGG.GTCAGCGTTG | A | x | 35004_12_15398_XS2_1 | UnannotatedRegion |
| 246 | AAGGGCAGGG.GTCAGCGTTG | A | x | 35005_12_13952_XS2_1 | UnannotatedRegion |
| 246 | AAGGGCAGGG.GTCAGCGTTG | A | x | 35006_12_15317_XS2_1 | UnannotatedRegion |
| 246 | AAGGGCAGGG.GTCAGCGTTG | A | x | 35007_12_15047_XS2_1 | UnannotatedRegion |
| 246 | AAGGGCAGGG.GTCAGCGTTG | A | x | 35008_12_15661_XS2_1 | UnannotatedRegion |
| 246 | AAGGGCAGGG.GTCAGCGTTG | A | x | 35009_12_15657_XS2_1 | UnannotatedRegion |
| 246 | AAGGGCAGGG.GTCAGCGTTG | A | x | 35010_12_17186_XS2_1 | UnannotatedRegion |
| 246 | AAGGGCAGGG.GTCAGCGTTG | A | x | 35011_12_17194_XS2_1 | UnannotatedRegion |
| 246 | AAGGGCAGGG.GTCAGCGTTG | A | x | 35012_12_17009_XS2_1 | UnannotatedRegion |
| 246 | AAGGGCAGGG.GTCAGCGTTG | A | x | 35013_12_14973_XS2_1 | UnannotatedRegion |
|  |  |  |  |  |  |
| 261 | AATAACATTA.CCGACCGCAA | T | 1303309 R | 19260_WUE_2594 | UnannotatedRegion |
| 261 | AATAACATTA.CCGACCGCAA | C | x | 34990_Tchad54_11 | UnannotatedRegion |
| 261 | AATAACATTA.CCGACCGCAA | C | x | 34991_Tchad78_11 | UnannotatedRegion |
| 261 | AATAACATTA.CCGACCGCAA | C | x | 34992_Tchad95_11 | UnannotatedRegion |
| 261 | AATAACATTA.CCGACCGCAA | C | x | 34994_Tchad106_11 | UnannotatedRegion |
| 261 | AATAACATTA.CCGACCGCAA | C | x | 34995_8_2011 | UnannotatedRegion |
| 261 | AATAACATTA.CCGACCGCAA | C | x | 34996_10_2011 | UnannotatedRegion |
| 261 | AATAACATTA.CCGACCGCAA | C | x | 34997_39_2011 | UnannotatedRegion |
| 261 | AATAACATTA.CCGACCGCAA | C | x | 34998_59_2011 | UnannotatedRegion |
| 261 | AATAACATTA.CCGACCGCAA | C | x | 34999_63_2011 | UnannotatedRegion |
| 261 | AATAACATTA.CCGACCGCAA | C | x | 35000_75_2011 | UnannotatedRegion |
| 261 | AATAACATTA.CCGACCGCAA | C | x | 35001_76_2011 | UnannotatedRegion |
| 261 | AATAACATTA.CCGACCGCAA | C | x | 35002_120_2011 | UnannotatedRegion |
| 261 | AATAACATTA.CCGACCGCAA | C | x | 35003_403_2011 | UnannotatedRegion |
| 261 | AATAACATTA.CCGACCGCAA | C | x | 35004_12_15398_XS2_1 | UnannotatedRegion |
| 261 | AATAACATTA.CCGACCGCAA | C | x | 35005_12_13952_XS2_1 | UnannotatedRegion |
| 261 | AATAACATTA.CCGACCGCAA | C | x | 35006_12_15317_XS2_1 | UnannotatedRegion |
| 261 | AATAACATTA.CCGACCGCAA | C | x | 35007_12_15047_XS2_1 | UnannotatedRegion |
| 261 | AATAACATTA.CCGACCGCAA | C | x | 35008_12_15661_XS2_1 | UnannotatedRegion |
| 261 | AATAACATTA.CCGACCGCAA | C | x | 35009_12_15657_XS2_1 | UnannotatedRegion |
| 261 | AATAACATTA.CCGACCGCAA | C | x | 35010_12_17186_XS2_1 | UnannotatedRegion |
| 261 | AATAACATTA.CCGACCGCAA | C | x | 35011_12_17194_XS2_1 | UnannotatedRegion |
| 261 | AATAACATTA.CCGACCGCAA | C | x | 35012_12_17009_XS2_1 | UnannotatedRegion |
| 261 | AATAACATTA.CCGACCGCAA | C | x | 35013_12_14973_XS2_1 | UnannotatedRegion |
|  |  |  |  |  |  |
| 265 | AATACGGCAA.TTTTTTATTG | G | x | 34990_Tchad54_11 | NotInAnnotatedGenome |
| 265 | AATACGGCAA.TTTTTTATTG | G | x | 34991_Tchad78_11 | NotInAnnotatedGenome |
| 265 | AATACGGCAA.TTTTTTATTG | A | x | 34992_Tchad95_11 | NotInAnnotatedGenome |
| 265 | AATACGGCAA.TTTTTTATTG | G | x | 34995_8_2011 | NotInAnnotatedGenome |
| 265 | AATACGGCAA.TTTTTTATTG | G | x | 34996_10_2011 | NotInAnnotatedGenome |
| 265 | AATACGGCAA.TTTTTTATTG | G | x | 34997_39_2011 | NotInAnnotatedGenome |
| 265 | AATACGGCAA.TTTTTTATTG | G | x | 34998_59_2011 | NotInAnnotatedGenome |
| 265 | AATACGGCAA.TTTTTTATTG | G | x | 34999_63_2011 | NotInAnnotatedGenome |
| 265 | AATACGGCAA.TTTTTTATTG | G | x | 35000_75_2011 | NotInAnnotatedGenome |
| 265 | AATACGGCAA.TTTTTTATTG | G | x | 35001_76_2011 | NotInAnnotatedGenome |
| 265 | AATACGGCAA.TTTTTTATTG | G | x | 35002_120_2011 | NotInAnnotatedGenome |
| 265 | AATACGGCAA.TTTTTTATTG | G | x | 35003_403_2011 | NotInAnnotatedGenome |
| 265 | AATACGGCAA.TTTTTTATTG | G | x | 35004_12_15398_XS2_1 | NotInAnnotatedGenome |
| 265 | AATACGGCAA.TTTTTTATTG | G | x | 35005_12_13952_XS2_1 | NotInAnnotatedGenome |
| 265 | AATACGGCAA.TTTTTTATTG | G | x | 35006_12_15317_XS2_1 | NotInAnnotatedGenome |
| 265 | AATACGGCAA.TTTTTTATTG | A | x | 35007_12_15047_XS2_1 | NotInAnnotatedGenome |
| 265 | AATACGGCAA.TTTTTTATTG | A | x | 35008_12_15661_XS2_1 | NotInAnnotatedGenome |
| 265 | AATACGGCAA.TTTTTTATTG | A | x | 35009_12_15657_XS2_1 | NotInAnnotatedGenome |
| 265 | AATACGGCAA.TTTTTTATTG | G | x | 35010_12_17186_XS2_1 | NotInAnnotatedGenome |
| 265 | AATACGGCAA.TTTTTTATTG | G | x | 35011_12_17194_XS2_1 | NotInAnnotatedGenome |
| 265 | AATACGGCAA.TTTTTTATTG | G | x | 35012_12_17009_XS2_1 | NotInAnnotatedGenome |
| 265 | AATACGGCAA.TTTTTTATTG | A | x | 35013_12_14973_XS2_1 | NotInAnnotatedGenome |
|  |  |  |  |  |  |
| 271 | AATAGAGTGG.AAATATGCAT | A | 160916 F | 19260_WUE_2594 | UnannotatedRegion |
| 271 | AATAGAGTGG.AAATATGCAT | G | x | 34990_Tchad54_11 | UnannotatedRegion |
| 271 | AATAGAGTGG.AAATATGCAT | G | x | 34991_Tchad78_11 | UnannotatedRegion |
| 271 | AATAGAGTGG.AAATATGCAT | G | x | 34992_Tchad95_11 | UnannotatedRegion |
| 271 | AATAGAGTGG.AAATATGCAT | G | x | 34994_Tchad106_11 | UnannotatedRegion |
| 271 | AATAGAGTGG.AAATATGCAT | G | x | 34995_8_2011 | UnannotatedRegion |
| 271 | AATAGAGTGG.AAATATGCAT | G | x | 34996_10_2011 | UnannotatedRegion |
| 271 | AATAGAGTGG.AAATATGCAT | G | x | 34997_39_2011 | UnannotatedRegion |
| 271 | AATAGAGTGG.AAATATGCAT | G | x | 34998_59_2011 | UnannotatedRegion |
| 271 | AATAGAGTGG.AAATATGCAT | G | x | 34999_63_2011 | UnannotatedRegion |
| 271 | AATAGAGTGG.AAATATGCAT | G | x | 35000_75_2011 | UnannotatedRegion |
| 271 | AATAGAGTGG.AAATATGCAT | G | x | 35001_76_2011 | UnannotatedRegion |
| 271 | AATAGAGTGG.AAATATGCAT | G | x | 35002_120_2011 | UnannotatedRegion |
| 271 | AATAGAGTGG.AAATATGCAT | G | x | 35003_403_2011 | UnannotatedRegion |
| 271 | AATAGAGTGG.AAATATGCAT | G | x | 35004_12_15398_XS2_1 | UnannotatedRegion |
| 271 | AATAGAGTGG.AAATATGCAT | G | x | 35005_12_13952_XS2_1 | UnannotatedRegion |
| 271 | AATAGAGTGG.AAATATGCAT | G | x | 35006_12_15317_XS2_1 | UnannotatedRegion |
| 271 | AATAGAGTGG.AAATATGCAT | G | x | 35007_12_15047_XS2_1 | UnannotatedRegion |
| 271 | AATAGAGTGG.AAATATGCAT | G | x | 35008_12_15661_XS2_1 | UnannotatedRegion |
| 271 | AATAGAGTGG.AAATATGCAT | G | x | 35009_12_15657_XS2_1 | UnannotatedRegion |
| 271 | AATAGAGTGG.AAATATGCAT | G | x | 35010_12_17186_XS2_1 | UnannotatedRegion |
| 271 | AATAGAGTGG.AAATATGCAT | G | x | 35011_12_17194_XS2_1 | UnannotatedRegion |
| 271 | AATAGAGTGG.AAATATGCAT | G | x | 35012_12_17009_XS2_1 | UnannotatedRegion |
| 271 | AATAGAGTGG.AAATATGCAT | G | x | 35013_12_14973_XS2_1 | UnannotatedRegion |
|  |  |  |  |  |  |
| 274 | AATATCAACT.TCTGTTTTAA | G | 1139535 F | 19260_WUE_2594 | UnannotatedRegion |
| 274 | AATATCAACT.TCTGTTTTAA | A | x | 34990_Tchad54_11 | UnannotatedRegion |
| 274 | AATATCAACT.TCTGTTTTAA | A | x | 34991_Tchad78_11 | UnannotatedRegion |
| 274 | AATATCAACT.TCTGTTTTAA | A | x | 34992_Tchad95_11 | UnannotatedRegion |
| 274 | AATATCAACT.TCTGTTTTAA | A | x | 34994_Tchad106_11 | UnannotatedRegion |
| 274 | AATATCAACT.TCTGTTTTAA | A | x | 34995_8_2011 | UnannotatedRegion |
| 274 | AATATCAACT.TCTGTTTTAA | A | x | 34996_10_2011 | UnannotatedRegion |
| 274 | AATATCAACT.TCTGTTTTAA | A | x | 34997_39_2011 | UnannotatedRegion |
| 274 | AATATCAACT.TCTGTTTTAA | A | x | 34998_59_2011 | UnannotatedRegion |
| 274 | AATATCAACT.TCTGTTTTAA | A | x | 34999_63_2011 | UnannotatedRegion |
| 274 | AATATCAACT.TCTGTTTTAA | A | x | 35000_75_2011 | UnannotatedRegion |
| 274 | AATATCAACT.TCTGTTTTAA | A | x | 35001_76_2011 | UnannotatedRegion |
| 274 | AATATCAACT.TCTGTTTTAA | A | x | 35002_120_2011 | UnannotatedRegion |
| 274 | AATATCAACT.TCTGTTTTAA | A | x | 35003_403_2011 | UnannotatedRegion |
| 274 | AATATCAACT.TCTGTTTTAA | A | x | 35004_12_15398_XS2_1 | UnannotatedRegion |
| 274 | AATATCAACT.TCTGTTTTAA | A | x | 35005_12_13952_XS2_1 | UnannotatedRegion |
| 274 | AATATCAACT.TCTGTTTTAA | A | x | 35006_12_15317_XS2_1 | UnannotatedRegion |
| 274 | AATATCAACT.TCTGTTTTAA | A | x | 35007_12_15047_XS2_1 | UnannotatedRegion |
| 274 | AATATCAACT.TCTGTTTTAA | A | x | 35008_12_15661_XS2_1 | UnannotatedRegion |
| 274 | AATATCAACT.TCTGTTTTAA | A | x | 35009_12_15657_XS2_1 | UnannotatedRegion |
| 274 | AATATCAACT.TCTGTTTTAA | A | x | 35010_12_17186_XS2_1 | UnannotatedRegion |
| 274 | AATATCAACT.TCTGTTTTAA | A | x | 35011_12_17194_XS2_1 | UnannotatedRegion |
| 274 | AATATCAACT.TCTGTTTTAA | A | x | 35012_12_17009_XS2_1 | UnannotatedRegion |
| 274 | AATATCAACT.TCTGTTTTAA | A | x | 35013_12_14973_XS2_1 | UnannotatedRegion |
|  |  |  |  |  |  |
| 275 | AATATCCGGA.GCACAAAACA | C | 1333345 R | 19260_WUE_2594 | UnannotatedRegion |
| 275 | AATATCCGGA.GCACAAAACA | T | x | 34990_Tchad54_11 | UnannotatedRegion |
| 275 | AATATCCGGA.GCACAAAACA | T | x | 34991_Tchad78_11 | UnannotatedRegion |
| 275 | AATATCCGGA.GCACAAAACA | T | x | 34992_Tchad95_11 | UnannotatedRegion |
| 275 | AATATCCGGA.GCACAAAACA | T | x | 34994_Tchad106_11 | UnannotatedRegion |
| 275 | AATATCCGGA.GCACAAAACA | T | x | 34995_8_2011 | UnannotatedRegion |
| 275 | AATATCCGGA.GCACAAAACA | T | x | 34996_10_2011 | UnannotatedRegion |
| 275 | AATATCCGGA.GCACAAAACA | T | x | 34997_39_2011 | UnannotatedRegion |
| 275 | AATATCCGGA.GCACAAAACA | T | x | 34998_59_2011 | UnannotatedRegion |
| 275 | AATATCCGGA.GCACAAAACA | T | x | 34999_63_2011 | UnannotatedRegion |
| 275 | AATATCCGGA.GCACAAAACA | T | x | 35000_75_2011 | UnannotatedRegion |
| 275 | AATATCCGGA.GCACAAAACA | T | x | 35001_76_2011 | UnannotatedRegion |
| 275 | AATATCCGGA.GCACAAAACA | T | x | 35002_120_2011 | UnannotatedRegion |
| 275 | AATATCCGGA.GCACAAAACA | T | x | 35003_403_2011 | UnannotatedRegion |
| 275 | AATATCCGGA.GCACAAAACA | T | x | 35004_12_15398_XS2_1 | UnannotatedRegion |
| 275 | AATATCCGGA.GCACAAAACA | T | x | 35005_12_13952_XS2_1 | UnannotatedRegion |
| 275 | AATATCCGGA.GCACAAAACA | T | x | 35006_12_15317_XS2_1 | UnannotatedRegion |
| 275 | AATATCCGGA.GCACAAAACA | T | x | 35007_12_15047_XS2_1 | UnannotatedRegion |
| 275 | AATATCCGGA.GCACAAAACA | T | x | 35008_12_15661_XS2_1 | UnannotatedRegion |
| 275 | AATATCCGGA.GCACAAAACA | T | x | 35009_12_15657_XS2_1 | UnannotatedRegion |
| 275 | AATATCCGGA.GCACAAAACA | T | x | 35010_12_17186_XS2_1 | UnannotatedRegion |
| 275 | AATATCCGGA.GCACAAAACA | T | x | 35011_12_17194_XS2_1 | UnannotatedRegion |
| 275 | AATATCCGGA.GCACAAAACA | T | x | 35012_12_17009_XS2_1 | UnannotatedRegion |
| 275 | AATATCCGGA.GCACAAAACA | T | x | 35013_12_14973_XS2_1 | UnannotatedRegion |
|  |  |  |  |  |  |
| 276 | AATATGTCGG.ATTTCCCGTA | C | 1147044 F | 19260_WUE_2594 | UnannotatedRegion |
| 276 | AATATGTCGG.ATTTCCCGTA | T | x | 34990_Tchad54_11 | UnannotatedRegion |
| 276 | AATATGTCGG.ATTTCCCGTA | T | x | 34991_Tchad78_11 | UnannotatedRegion |
| 276 | AATATGTCGG.ATTTCCCGTA | T | x | 34992_Tchad95_11 | UnannotatedRegion |
| 276 | AATATGTCGG.ATTTCCCGTA | T | x | 34994_Tchad106_11 | UnannotatedRegion |
| 276 | AATATGTCGG.ATTTCCCGTA | T | x | 34995_8_2011 | UnannotatedRegion |
| 276 | AATATGTCGG.ATTTCCCGTA | T | x | 34996_10_2011 | UnannotatedRegion |
| 276 | AATATGTCGG.ATTTCCCGTA | T | x | 34997_39_2011 | UnannotatedRegion |
| 276 | AATATGTCGG.ATTTCCCGTA | T | x | 34998_59_2011 | UnannotatedRegion |
| 276 | AATATGTCGG.ATTTCCCGTA | T | x | 34999_63_2011 | UnannotatedRegion |
| 276 | AATATGTCGG.ATTTCCCGTA | T | x | 35000_75_2011 | UnannotatedRegion |
| 276 | AATATGTCGG.ATTTCCCGTA | T | x | 35001_76_2011 | UnannotatedRegion |
| 276 | AATATGTCGG.ATTTCCCGTA | T | x | 35002_120_2011 | UnannotatedRegion |
| 276 | AATATGTCGG.ATTTCCCGTA | T | x | 35003_403_2011 | UnannotatedRegion |
| 276 | AATATGTCGG.ATTTCCCGTA | T | x | 35004_12_15398_XS2_1 | UnannotatedRegion |
| 276 | AATATGTCGG.ATTTCCCGTA | T | x | 35005_12_13952_XS2_1 | UnannotatedRegion |
| 276 | AATATGTCGG.ATTTCCCGTA | T | x | 35006_12_15317_XS2_1 | UnannotatedRegion |
| 276 | AATATGTCGG.ATTTCCCGTA | T | x | 35007_12_15047_XS2_1 | UnannotatedRegion |
| 276 | AATATGTCGG.ATTTCCCGTA | T | x | 35008_12_15661_XS2_1 | UnannotatedRegion |
| 276 | AATATGTCGG.ATTTCCCGTA | T | x | 35009_12_15657_XS2_1 | UnannotatedRegion |
| 276 | AATATGTCGG.ATTTCCCGTA | T | x | 35010_12_17186_XS2_1 | UnannotatedRegion |
| 276 | AATATGTCGG.ATTTCCCGTA | T | x | 35011_12_17194_XS2_1 | UnannotatedRegion |
| 276 | AATATGTCGG.ATTTCCCGTA | T | x | 35012_12_17009_XS2_1 | UnannotatedRegion |
| 276 | AATATGTCGG.ATTTCCCGTA | T | x | 35013_12_14973_XS2_1 | UnannotatedRegion |
|  |  |  |  |  |  |
| 280 | AATCCCGCAA.ATTTTTAGCT | A | 1303843 F | 19260_WUE_2594 | UnannotatedRegion |
| 280 | AATCCCGCAA.ATTTTTAGCT | G | x | 34990_Tchad54_11 | UnannotatedRegion |
| 280 | AATCCCGCAA.ATTTTTAGCT | G | x | 34991_Tchad78_11 | UnannotatedRegion |
| 280 | AATCCCGCAA.ATTTTTAGCT | G | x | 34992_Tchad95_11 | UnannotatedRegion |
| 280 | AATCCCGCAA.ATTTTTAGCT | G | x | 34994_Tchad106_11 | UnannotatedRegion |
| 280 | AATCCCGCAA.ATTTTTAGCT | G | x | 34995_8_2011 | UnannotatedRegion |
| 280 | AATCCCGCAA.ATTTTTAGCT | G | x | 34996_10_2011 | UnannotatedRegion |
| 280 | AATCCCGCAA.ATTTTTAGCT | G | x | 34997_39_2011 | UnannotatedRegion |
| 280 | AATCCCGCAA.ATTTTTAGCT | G | x | 34998_59_2011 | UnannotatedRegion |
| 280 | AATCCCGCAA.ATTTTTAGCT | G | x | 34999_63_2011 | UnannotatedRegion |
| 280 | AATCCCGCAA.ATTTTTAGCT | G | x | 35000_75_2011 | UnannotatedRegion |
| 280 | AATCCCGCAA.ATTTTTAGCT | G | x | 35001_76_2011 | UnannotatedRegion |
| 280 | AATCCCGCAA.ATTTTTAGCT | G | x | 35002_120_2011 | UnannotatedRegion |
| 280 | AATCCCGCAA.ATTTTTAGCT | G | x | 35003_403_2011 | UnannotatedRegion |
| 280 | AATCCCGCAA.ATTTTTAGCT | G | x | 35004_12_15398_XS2_1 | UnannotatedRegion |
| 280 | AATCCCGCAA.ATTTTTAGCT | G | x | 35005_12_13952_XS2_1 | UnannotatedRegion |
| 280 | AATCCCGCAA.ATTTTTAGCT | G | x | 35006_12_15317_XS2_1 | UnannotatedRegion |
| 280 | AATCCCGCAA.ATTTTTAGCT | G | x | 35007_12_15047_XS2_1 | UnannotatedRegion |
| 280 | AATCCCGCAA.ATTTTTAGCT | G | x | 35008_12_15661_XS2_1 | UnannotatedRegion |
| 280 | AATCCCGCAA.ATTTTTAGCT | G | x | 35009_12_15657_XS2_1 | UnannotatedRegion |
| 280 | AATCCCGCAA.ATTTTTAGCT | G | x | 35010_12_17186_XS2_1 | UnannotatedRegion |
| 280 | AATCCCGCAA.ATTTTTAGCT | G | x | 35011_12_17194_XS2_1 | UnannotatedRegion |
| 280 | AATCCCGCAA.ATTTTTAGCT | G | x | 35012_12_17009_XS2_1 | UnannotatedRegion |
| 280 | AATCCCGCAA.ATTTTTAGCT | G | x | 35013_12_14973_XS2_1 | UnannotatedRegion |
|  |  |  |  |  |  |
| 291 | AATCGGGCGT.CGCCGCAGGT | G | 390673 F | 19260_WUE_2594 | UnannotatedRegion |
| 291 | AATCGGGCGT.CGCCGCAGGT | G | x | 34990_Tchad54_11 | UnannotatedRegion |
| 291 | AATCGGGCGT.CGCCGCAGGT | G | x | 34991_Tchad78_11 | UnannotatedRegion |
| 291 | AATCGGGCGT.CGCCGCAGGT | G | x | 34992_Tchad95_11 | UnannotatedRegion |
| 291 | AATCGGGCGT.CGCCGCAGGT | G | x | 34994_Tchad106_11 | UnannotatedRegion |
| 291 | AATCGGGCGT.CGCCGCAGGT | A | x | 34995_8_2011 | UnannotatedRegion |
| 291 | AATCGGGCGT.CGCCGCAGGT | A | x | 34996_10_2011 | UnannotatedRegion |
| 291 | AATCGGGCGT.CGCCGCAGGT | G | x | 34997_39_2011 | UnannotatedRegion |
| 291 | AATCGGGCGT.CGCCGCAGGT | G | x | 34998_59_2011 | UnannotatedRegion |
| 291 | AATCGGGCGT.CGCCGCAGGT | G | x | 34999_63_2011 | UnannotatedRegion |
| 291 | AATCGGGCGT.CGCCGCAGGT | G | x | 35000_75_2011 | UnannotatedRegion |
| 291 | AATCGGGCGT.CGCCGCAGGT | G | x | 35001_76_2011 | UnannotatedRegion |
| 291 | AATCGGGCGT.CGCCGCAGGT | G | x | 35002_120_2011 | UnannotatedRegion |
| 291 | AATCGGGCGT.CGCCGCAGGT | G | x | 35003_403_2011 | UnannotatedRegion |
| 291 | AATCGGGCGT.CGCCGCAGGT | G | x | 35004_12_15398_XS2_1 | UnannotatedRegion |
| 291 | AATCGGGCGT.CGCCGCAGGT | G | x | 35005_12_13952_XS2_1 | UnannotatedRegion |
| 291 | AATCGGGCGT.CGCCGCAGGT | G | x | 35006_12_15317_XS2_1 | UnannotatedRegion |
| 291 | AATCGGGCGT.CGCCGCAGGT | G | x | 35007_12_15047_XS2_1 | UnannotatedRegion |
| 291 | AATCGGGCGT.CGCCGCAGGT | G | x | 35008_12_15661_XS2_1 | UnannotatedRegion |
| 291 | AATCGGGCGT.CGCCGCAGGT | G | x | 35009_12_15657_XS2_1 | UnannotatedRegion |
| 291 | AATCGGGCGT.CGCCGCAGGT | G | x | 35010_12_17186_XS2_1 | UnannotatedRegion |
| 291 | AATCGGGCGT.CGCCGCAGGT | G | x | 35011_12_17194_XS2_1 | UnannotatedRegion |
| 291 | AATCGGGCGT.CGCCGCAGGT | G | x | 35012_12_17009_XS2_1 | UnannotatedRegion |
| 291 | AATCGGGCGT.CGCCGCAGGT | G | x | 35013_12_14973_XS2_1 | UnannotatedRegion |
|  |  |  |  |  |  |
| 300 | AATGACGACT.CTTCACCAGC | T | 1487308 R | 19260_WUE_2594 | UnannotatedRegion |
| 300 | AATGACGACT.CTTCACCAGC | C | x | 34990_Tchad54_11 | UnannotatedRegion |
| 300 | AATGACGACT.CTTCACCAGC | C | x | 34991_Tchad78_11 | UnannotatedRegion |
| 300 | AATGACGACT.CTTCACCAGC | C | x | 34992_Tchad95_11 | UnannotatedRegion |
| 300 | AATGACGACT.CTTCACCAGC | C | x | 34995_8_2011 | UnannotatedRegion |
| 300 | AATGACGACT.CTTCACCAGC | C | x | 34996_10_2011 | UnannotatedRegion |
| 300 | AATGACGACT.CTTCACCAGC | C | x | 34997_39_2011 | UnannotatedRegion |
| 300 | AATGACGACT.CTTCACCAGC | C | x | 34998_59_2011 | UnannotatedRegion |
| 300 | AATGACGACT.CTTCACCAGC | C | x | 34999_63_2011 | UnannotatedRegion |
| 300 | AATGACGACT.CTTCACCAGC | C | x | 35000_75_2011 | UnannotatedRegion |
| 300 | AATGACGACT.CTTCACCAGC | C | x | 35001_76_2011 | UnannotatedRegion |
| 300 | AATGACGACT.CTTCACCAGC | C | x | 35002_120_2011 | UnannotatedRegion |
| 300 | AATGACGACT.CTTCACCAGC | C | x | 35003_403_2011 | UnannotatedRegion |
| 300 | AATGACGACT.CTTCACCAGC | C | x | 35004_12_15398_XS2_1 | UnannotatedRegion |
| 300 | AATGACGACT.CTTCACCAGC | C | x | 35005_12_13952_XS2_1 | UnannotatedRegion |
| 300 | AATGACGACT.CTTCACCAGC | C | x | 35006_12_15317_XS2_1 | UnannotatedRegion |
| 300 | AATGACGACT.CTTCACCAGC | C | x | 35007_12_15047_XS2_1 | UnannotatedRegion |
| 300 | AATGACGACT.CTTCACCAGC | C | x | 35008_12_15661_XS2_1 | UnannotatedRegion |
| 300 | AATGACGACT.CTTCACCAGC | C | x | 35009_12_15657_XS2_1 | UnannotatedRegion |
| 300 | AATGACGACT.CTTCACCAGC | C | x | 35010_12_17186_XS2_1 | UnannotatedRegion |
| 300 | AATGACGACT.CTTCACCAGC | C | x | 35011_12_17194_XS2_1 | UnannotatedRegion |
| 300 | AATGACGACT.CTTCACCAGC | C | x | 35012_12_17009_XS2_1 | UnannotatedRegion |
| 300 | AATGACGACT.CTTCACCAGC | C | x | 35013_12_14973_XS2_1 | UnannotatedRegion |
|  |  |  |  |  |  |
| 315 | AATTATATTA.CCGCGCCTCA | G | 258281 F | 19260_WUE_2594 | UnannotatedRegion |
| 315 | AATTATATTA.CCGCGCCTCA | G | x | 34990_Tchad54_11 | UnannotatedRegion |
| 315 | AATTATATTA.CCGCGCCTCA | G | x | 34991_Tchad78_11 | UnannotatedRegion |
| 315 | AATTATATTA.CCGCGCCTCA | G | x | 34992_Tchad95_11 | UnannotatedRegion |
| 315 | AATTATATTA.CCGCGCCTCA | G | x | 34994_Tchad106_11 | UnannotatedRegion |
| 315 | AATTATATTA.CCGCGCCTCA | A | x | 34995_8_2011 | UnannotatedRegion |
| 315 | AATTATATTA.CCGCGCCTCA | A | x | 34996_10_2011 | UnannotatedRegion |
| 315 | AATTATATTA.CCGCGCCTCA | G | x | 34997_39_2011 | UnannotatedRegion |
| 315 | AATTATATTA.CCGCGCCTCA | G | x | 34998_59_2011 | UnannotatedRegion |
| 315 | AATTATATTA.CCGCGCCTCA | G | x | 34999_63_2011 | UnannotatedRegion |
| 315 | AATTATATTA.CCGCGCCTCA | G | x | 35000_75_2011 | UnannotatedRegion |
| 315 | AATTATATTA.CCGCGCCTCA | G | x | 35001_76_2011 | UnannotatedRegion |
| 315 | AATTATATTA.CCGCGCCTCA | G | x | 35002_120_2011 | UnannotatedRegion |
| 315 | AATTATATTA.CCGCGCCTCA | G | x | 35003_403_2011 | UnannotatedRegion |
| 315 | AATTATATTA.CCGCGCCTCA | G | x | 35004_12_15398_XS2_1 | UnannotatedRegion |
| 315 | AATTATATTA.CCGCGCCTCA | G | x | 35005_12_13952_XS2_1 | UnannotatedRegion |
| 315 | AATTATATTA.CCGCGCCTCA | G | x | 35006_12_15317_XS2_1 | UnannotatedRegion |
| 315 | AATTATATTA.CCGCGCCTCA | G | x | 35007_12_15047_XS2_1 | UnannotatedRegion |
| 315 | AATTATATTA.CCGCGCCTCA | G | x | 35008_12_15661_XS2_1 | UnannotatedRegion |
| 315 | AATTATATTA.CCGCGCCTCA | G | x | 35009_12_15657_XS2_1 | UnannotatedRegion |
| 315 | AATTATATTA.CCGCGCCTCA | G | x | 35010_12_17186_XS2_1 | UnannotatedRegion |
| 315 | AATTATATTA.CCGCGCCTCA | G | x | 35011_12_17194_XS2_1 | UnannotatedRegion |
| 315 | AATTATATTA.CCGCGCCTCA | G | x | 35012_12_17009_XS2_1 | UnannotatedRegion |
| 315 | AATTATATTA.CCGCGCCTCA | G | x | 35013_12_14973_XS2_1 | UnannotatedRegion |
|  |  |  |  |  |  |
| 318 | AATTCTCTGC.TTTGCTTGAA | T | 508264 F | 19260_WUE_2594 | UnannotatedRegion |
| 318 | AATTCTCTGC.TTTGCTTGAA | C | x | 34990_Tchad54_11 | UnannotatedRegion |
| 318 | AATTCTCTGC.TTTGCTTGAA | C | x | 34991_Tchad78_11 | UnannotatedRegion |
| 318 | AATTCTCTGC.TTTGCTTGAA | C | x | 34992_Tchad95_11 | UnannotatedRegion |
| 318 | AATTCTCTGC.TTTGCTTGAA | C | x | 34994_Tchad106_11 | UnannotatedRegion |
| 318 | AATTCTCTGC.TTTGCTTGAA | C | x | 34995_8_2011 | UnannotatedRegion |
| 318 | AATTCTCTGC.TTTGCTTGAA | C | x | 34996_10_2011 | UnannotatedRegion |
| 318 | AATTCTCTGC.TTTGCTTGAA | C | x | 34997_39_2011 | UnannotatedRegion |
| 318 | AATTCTCTGC.TTTGCTTGAA | C | x | 34998_59_2011 | UnannotatedRegion |
| 318 | AATTCTCTGC.TTTGCTTGAA | C | x | 34999_63_2011 | UnannotatedRegion |
| 318 | AATTCTCTGC.TTTGCTTGAA | C | x | 35000_75_2011 | UnannotatedRegion |
| 318 | AATTCTCTGC.TTTGCTTGAA | C | x | 35001_76_2011 | UnannotatedRegion |
| 318 | AATTCTCTGC.TTTGCTTGAA | C | x | 35002_120_2011 | UnannotatedRegion |
| 318 | AATTCTCTGC.TTTGCTTGAA | C | x | 35003_403_2011 | UnannotatedRegion |
| 318 | AATTCTCTGC.TTTGCTTGAA | C | x | 35004_12_15398_XS2_1 | UnannotatedRegion |
| 318 | AATTCTCTGC.TTTGCTTGAA | C | x | 35005_12_13952_XS2_1 | UnannotatedRegion |
| 318 | AATTCTCTGC.TTTGCTTGAA | C | x | 35006_12_15317_XS2_1 | UnannotatedRegion |
| 318 | AATTCTCTGC.TTTGCTTGAA | C | x | 35007_12_15047_XS2_1 | UnannotatedRegion |
| 318 | AATTCTCTGC.TTTGCTTGAA | C | x | 35008_12_15661_XS2_1 | UnannotatedRegion |
| 318 | AATTCTCTGC.TTTGCTTGAA | C | x | 35009_12_15657_XS2_1 | UnannotatedRegion |
| 318 | AATTCTCTGC.TTTGCTTGAA | C | x | 35010_12_17186_XS2_1 | UnannotatedRegion |
| 318 | AATTCTCTGC.TTTGCTTGAA | C | x | 35011_12_17194_XS2_1 | UnannotatedRegion |
| 318 | AATTCTCTGC.TTTGCTTGAA | C | x | 35012_12_17009_XS2_1 | UnannotatedRegion |
| 318 | AATTCTCTGC.TTTGCTTGAA | C | x | 35013_12_14973_XS2_1 | UnannotatedRegion |
|  |  |  |  |  |  |
| 320 | AATTGAATGC.GAGTGTCATG | C | x | 34990_Tchad54_11 | NotInAnnotatedGenome |
| 320 | AATTGAATGC.GAGTGTCATG | C | x | 34991_Tchad78_11 | NotInAnnotatedGenome |
| 320 | AATTGAATGC.GAGTGTCATG | C | x | 34992_Tchad95_11 | NotInAnnotatedGenome |
| 320 | AATTGAATGC.GAGTGTCATG | C | x | 34994_Tchad106_11 | NotInAnnotatedGenome |
| 320 | AATTGAATGC.GAGTGTCATG | T | x | 34995_8_2011 | NotInAnnotatedGenome |
| 320 | AATTGAATGC.GAGTGTCATG | T | x | 34996_10_2011 | NotInAnnotatedGenome |
| 320 | AATTGAATGC.GAGTGTCATG | C | x | 34997_39_2011 | NotInAnnotatedGenome |
| 320 | AATTGAATGC.GAGTGTCATG | C | x | 34998_59_2011 | NotInAnnotatedGenome |
| 320 | AATTGAATGC.GAGTGTCATG | C | x | 34999_63_2011 | NotInAnnotatedGenome |
| 320 | AATTGAATGC.GAGTGTCATG | C | x | 35000_75_2011 | NotInAnnotatedGenome |
| 320 | AATTGAATGC.GAGTGTCATG | C | x | 35001_76_2011 | NotInAnnotatedGenome |
| 320 | AATTGAATGC.GAGTGTCATG | C | x | 35002_120_2011 | NotInAnnotatedGenome |
| 320 | AATTGAATGC.GAGTGTCATG | C | x | 35003_403_2011 | NotInAnnotatedGenome |
| 320 | AATTGAATGC.GAGTGTCATG | C | x | 35004_12_15398_XS2_1 | NotInAnnotatedGenome |
| 320 | AATTGAATGC.GAGTGTCATG | C | x | 35005_12_13952_XS2_1 | NotInAnnotatedGenome |
| 320 | AATTGAATGC.GAGTGTCATG | C | x | 35006_12_15317_XS2_1 | NotInAnnotatedGenome |
| 320 | AATTGAATGC.GAGTGTCATG | C | x | 35007_12_15047_XS2_1 | NotInAnnotatedGenome |
| 320 | AATTGAATGC.GAGTGTCATG | C | x | 35008_12_15661_XS2_1 | NotInAnnotatedGenome |
| 320 | AATTGAATGC.GAGTGTCATG | C | x | 35009_12_15657_XS2_1 | NotInAnnotatedGenome |
| 320 | AATTGAATGC.GAGTGTCATG | C | x | 35010_12_17186_XS2_1 | NotInAnnotatedGenome |
| 320 | AATTGAATGC.GAGTGTCATG | C | x | 35011_12_17194_XS2_1 | NotInAnnotatedGenome |
| 320 | AATTGAATGC.GAGTGTCATG | C | x | 35012_12_17009_XS2_1 | NotInAnnotatedGenome |
| 320 | AATTGAATGC.GAGTGTCATG | C | x | 35013_12_14973_XS2_1 | NotInAnnotatedGenome |
|  |  |  |  |  |  |
| 324 | AATTGGCGGT.GGGATAGATG | C | x | 34990_Tchad54_11 | NotInAnnotatedGenome |
| 324 | AATTGGCGGT.GGGATAGATG | C | x | 34991_Tchad78_11 | NotInAnnotatedGenome |
| 324 | AATTGGCGGT.GGGATAGATG | C | x | 34992_Tchad95_11 | NotInAnnotatedGenome |
| 324 | AATTGGCGGT.GGGATAGATG | T | x | 34994_Tchad106_11 | NotInAnnotatedGenome |
| 324 | AATTGGCGGT.GGGATAGATG | C | x | 34995_8_2011 | NotInAnnotatedGenome |
| 324 | AATTGGCGGT.GGGATAGATG | C | x | 34996_10_2011 | NotInAnnotatedGenome |
| 324 | AATTGGCGGT.GGGATAGATG | C | x | 34997_39_2011 | NotInAnnotatedGenome |
| 324 | AATTGGCGGT.GGGATAGATG | C | x | 34998_59_2011 | NotInAnnotatedGenome |
| 324 | AATTGGCGGT.GGGATAGATG | C | x | 34999_63_2011 | NotInAnnotatedGenome |
| 324 | AATTGGCGGT.GGGATAGATG | C | x | 35000_75_2011 | NotInAnnotatedGenome |
| 324 | AATTGGCGGT.GGGATAGATG | C | x | 35001_76_2011 | NotInAnnotatedGenome |
| 324 | AATTGGCGGT.GGGATAGATG | C | x | 35002_120_2011 | NotInAnnotatedGenome |
| 324 | AATTGGCGGT.GGGATAGATG | C | x | 35003_403_2011 | NotInAnnotatedGenome |
| 324 | AATTGGCGGT.GGGATAGATG | C | x | 35004_12_15398_XS2_1 | NotInAnnotatedGenome |
| 324 | AATTGGCGGT.GGGATAGATG | C | x | 35005_12_13952_XS2_1 | NotInAnnotatedGenome |
| 324 | AATTGGCGGT.GGGATAGATG | C | x | 35006_12_15317_XS2_1 | NotInAnnotatedGenome |
| 324 | AATTGGCGGT.GGGATAGATG | C | x | 35007_12_15047_XS2_1 | NotInAnnotatedGenome |
| 324 | AATTGGCGGT.GGGATAGATG | C | x | 35008_12_15661_XS2_1 | NotInAnnotatedGenome |
| 324 | AATTGGCGGT.GGGATAGATG | C | x | 35009_12_15657_XS2_1 | NotInAnnotatedGenome |
| 324 | AATTGGCGGT.GGGATAGATG | C | x | 35010_12_17186_XS2_1 | NotInAnnotatedGenome |
| 324 | AATTGGCGGT.GGGATAGATG | C | x | 35011_12_17194_XS2_1 | NotInAnnotatedGenome |
| 324 | AATTGGCGGT.GGGATAGATG | C | x | 35012_12_17009_XS2_1 | NotInAnnotatedGenome |
| 324 | AATTGGCGGT.GGGATAGATG | C | x | 35013_12_14973_XS2_1 | NotInAnnotatedGenome |
|  |  |  |  |  |  |
| 333 | ACAAAAGGAA.CCGATATGCG | G | 1331834 R | 19260_WUE_2594 | UnannotatedRegion |
| 333 | ACAAAAGGAA.CCGATATGCG | G | x | 34990_Tchad54_11 | UnannotatedRegion |
| 333 | ACAAAAGGAA.CCGATATGCG | G | x | 34991_Tchad78_11 | UnannotatedRegion |
| 333 | ACAAAAGGAA.CCGATATGCG | G | x | 34992_Tchad95_11 | UnannotatedRegion |
| 333 | ACAAAAGGAA.CCGATATGCG | G | x | 34994_Tchad106_11 | UnannotatedRegion |
| 333 | ACAAAAGGAA.CCGATATGCG | A | x | 34995_8_2011 | UnannotatedRegion |
| 333 | ACAAAAGGAA.CCGATATGCG | A | x | 34996_10_2011 | UnannotatedRegion |
| 333 | ACAAAAGGAA.CCGATATGCG | G | x | 34997_39_2011 | UnannotatedRegion |
| 333 | ACAAAAGGAA.CCGATATGCG | G | x | 34998_59_2011 | UnannotatedRegion |
| 333 | ACAAAAGGAA.CCGATATGCG | G | x | 34999_63_2011 | UnannotatedRegion |
| 333 | ACAAAAGGAA.CCGATATGCG | G | x | 35000_75_2011 | UnannotatedRegion |
| 333 | ACAAAAGGAA.CCGATATGCG | G | x | 35001_76_2011 | UnannotatedRegion |
| 333 | ACAAAAGGAA.CCGATATGCG | G | x | 35002_120_2011 | UnannotatedRegion |
| 333 | ACAAAAGGAA.CCGATATGCG | G | x | 35003_403_2011 | UnannotatedRegion |
| 333 | ACAAAAGGAA.CCGATATGCG | G | x | 35004_12_15398_XS2_1 | UnannotatedRegion |
| 333 | ACAAAAGGAA.CCGATATGCG | G | x | 35005_12_13952_XS2_1 | UnannotatedRegion |
| 333 | ACAAAAGGAA.CCGATATGCG | G | x | 35006_12_15317_XS2_1 | UnannotatedRegion |
| 333 | ACAAAAGGAA.CCGATATGCG | G | x | 35007_12_15047_XS2_1 | UnannotatedRegion |
| 333 | ACAAAAGGAA.CCGATATGCG | G | x | 35008_12_15661_XS2_1 | UnannotatedRegion |
| 333 | ACAAAAGGAA.CCGATATGCG | G | x | 35009_12_15657_XS2_1 | UnannotatedRegion |
| 333 | ACAAAAGGAA.CCGATATGCG | G | x | 35010_12_17186_XS2_1 | UnannotatedRegion |
| 333 | ACAAAAGGAA.CCGATATGCG | G | x | 35011_12_17194_XS2_1 | UnannotatedRegion |
| 333 | ACAAAAGGAA.CCGATATGCG | G | x | 35012_12_17009_XS2_1 | UnannotatedRegion |
| 333 | ACAAAAGGAA.CCGATATGCG | G | x | 35013_12_14973_XS2_1 | UnannotatedRegion |
|  |  |  |  |  |  |
| 387 | ACATTAAAAT.ACTGATGAGG | A | 1270322 F | 19260_WUE_2594 | UnannotatedRegion |
| 387 | ACATTAAAAT.ACTGATGAGG | G | x | 34990_Tchad54_11 | UnannotatedRegion |
| 387 | ACATTAAAAT.ACTGATGAGG | G | x | 34991_Tchad78_11 | UnannotatedRegion |
| 387 | ACATTAAAAT.ACTGATGAGG | G | x | 34992_Tchad95_11 | UnannotatedRegion |
| 387 | ACATTAAAAT.ACTGATGAGG | G | x | 34994_Tchad106_11 | UnannotatedRegion |
| 387 | ACATTAAAAT.ACTGATGAGG | G | x | 34995_8_2011 | UnannotatedRegion |
| 387 | ACATTAAAAT.ACTGATGAGG | G | x | 34996_10_2011 | UnannotatedRegion |
| 387 | ACATTAAAAT.ACTGATGAGG | G | x | 34997_39_2011 | UnannotatedRegion |
| 387 | ACATTAAAAT.ACTGATGAGG | G | x | 34998_59_2011 | UnannotatedRegion |
| 387 | ACATTAAAAT.ACTGATGAGG | G | x | 34999_63_2011 | UnannotatedRegion |
| 387 | ACATTAAAAT.ACTGATGAGG | G | x | 35000_75_2011 | UnannotatedRegion |
| 387 | ACATTAAAAT.ACTGATGAGG | G | x | 35001_76_2011 | UnannotatedRegion |
| 387 | ACATTAAAAT.ACTGATGAGG | G | x | 35002_120_2011 | UnannotatedRegion |
| 387 | ACATTAAAAT.ACTGATGAGG | G | x | 35003_403_2011 | UnannotatedRegion |
| 387 | ACATTAAAAT.ACTGATGAGG | G | x | 35004_12_15398_XS2_1 | UnannotatedRegion |
| 387 | ACATTAAAAT.ACTGATGAGG | G | x | 35005_12_13952_XS2_1 | UnannotatedRegion |
| 387 | ACATTAAAAT.ACTGATGAGG | G | x | 35006_12_15317_XS2_1 | UnannotatedRegion |
| 387 | ACATTAAAAT.ACTGATGAGG | G | x | 35007_12_15047_XS2_1 | UnannotatedRegion |
| 387 | ACATTAAAAT.ACTGATGAGG | G | x | 35008_12_15661_XS2_1 | UnannotatedRegion |
| 387 | ACATTAAAAT.ACTGATGAGG | G | x | 35009_12_15657_XS2_1 | UnannotatedRegion |
| 387 | ACATTAAAAT.ACTGATGAGG | G | x | 35010_12_17186_XS2_1 | UnannotatedRegion |
| 387 | ACATTAAAAT.ACTGATGAGG | G | x | 35011_12_17194_XS2_1 | UnannotatedRegion |
| 387 | ACATTAAAAT.ACTGATGAGG | G | x | 35012_12_17009_XS2_1 | UnannotatedRegion |
| 387 | ACATTAAAAT.ACTGATGAGG | G | x | 35013_12_14973_XS2_1 | UnannotatedRegion |
|  |  |  |  |  |  |
| 404 | ACCATTGAGC.ACACCCGCAT | T | 2129775 F | 19260_WUE_2594 | NotProteinCoding |
| 404 | ACCATTGAGC.ACACCCGCAT | C | x | 34990_Tchad54_11 | NotProteinCoding |
| 404 | ACCATTGAGC.ACACCCGCAT | C | x | 34991_Tchad78_11 | NotProteinCoding |
| 404 | ACCATTGAGC.ACACCCGCAT | C | x | 34992_Tchad95_11 | NotProteinCoding |
| 404 | ACCATTGAGC.ACACCCGCAT | C | x | 34994_Tchad106_11 | NotProteinCoding |
| 404 | ACCATTGAGC.ACACCCGCAT | T | x | 34995_8_2011 | NotProteinCoding |
| 404 | ACCATTGAGC.ACACCCGCAT | T | x | 34996_10_2011 | NotProteinCoding |
| 404 | ACCATTGAGC.ACACCCGCAT | C | x | 34997_39_2011 | NotProteinCoding |
| 404 | ACCATTGAGC.ACACCCGCAT | C | x | 34998_59_2011 | NotProteinCoding |
| 404 | ACCATTGAGC.ACACCCGCAT | C | x | 34999_63_2011 | NotProteinCoding |
| 404 | ACCATTGAGC.ACACCCGCAT | C | x | 35000_75_2011 | NotProteinCoding |
| 404 | ACCATTGAGC.ACACCCGCAT | C | x | 35001_76_2011 | NotProteinCoding |
| 404 | ACCATTGAGC.ACACCCGCAT | C | x | 35002_120_2011 | NotProteinCoding |
| 404 | ACCATTGAGC.ACACCCGCAT | C | x | 35003_403_2011 | NotProteinCoding |
| 404 | ACCATTGAGC.ACACCCGCAT | C | x | 35004_12_15398_XS2_1 | NotProteinCoding |
| 404 | ACCATTGAGC.ACACCCGCAT | C | x | 35005_12_13952_XS2_1 | NotProteinCoding |
| 404 | ACCATTGAGC.ACACCCGCAT | C | x | 35006_12_15317_XS2_1 | NotProteinCoding |
| 404 | ACCATTGAGC.ACACCCGCAT | C | x | 35007_12_15047_XS2_1 | NotProteinCoding |
| 404 | ACCATTGAGC.ACACCCGCAT | C | x | 35008_12_15661_XS2_1 | NotProteinCoding |
| 404 | ACCATTGAGC.ACACCCGCAT | C | x | 35009_12_15657_XS2_1 | NotProteinCoding |
| 404 | ACCATTGAGC.ACACCCGCAT | C | x | 35010_12_17186_XS2_1 | NotProteinCoding |
| 404 | ACCATTGAGC.ACACCCGCAT | C | x | 35011_12_17194_XS2_1 | NotProteinCoding |
| 404 | ACCATTGAGC.ACACCCGCAT | C | x | 35012_12_17009_XS2_1 | NotProteinCoding |
| 404 | ACCATTGAGC.ACACCCGCAT | C | x | 35013_12_14973_XS2_1 | NotProteinCoding |
|  |  |  |  |  |  |
| 428 | ACCGTTAAGG.ACAGGGTTAA | A | 1303265 F | 19260_WUE_2594 | UnannotatedRegion |
| 428 | ACCGTTAAGG.ACAGGGTTAA | C | x | 34990_Tchad54_11 | UnannotatedRegion |
| 428 | ACCGTTAAGG.ACAGGGTTAA | C | x | 34991_Tchad78_11 | UnannotatedRegion |
| 428 | ACCGTTAAGG.ACAGGGTTAA | C | x | 34992_Tchad95_11 | UnannotatedRegion |
| 428 | ACCGTTAAGG.ACAGGGTTAA | C | x | 34994_Tchad106_11 | UnannotatedRegion |
| 428 | ACCGTTAAGG.ACAGGGTTAA | C | x | 34995_8_2011 | UnannotatedRegion |
| 428 | ACCGTTAAGG.ACAGGGTTAA | C | x | 34996_10_2011 | UnannotatedRegion |
| 428 | ACCGTTAAGG.ACAGGGTTAA | C | x | 34997_39_2011 | UnannotatedRegion |
| 428 | ACCGTTAAGG.ACAGGGTTAA | C | x | 34998_59_2011 | UnannotatedRegion |
| 428 | ACCGTTAAGG.ACAGGGTTAA | C | x | 34999_63_2011 | UnannotatedRegion |
| 428 | ACCGTTAAGG.ACAGGGTTAA | C | x | 35000_75_2011 | UnannotatedRegion |
| 428 | ACCGTTAAGG.ACAGGGTTAA | C | x | 35001_76_2011 | UnannotatedRegion |
| 428 | ACCGTTAAGG.ACAGGGTTAA | C | x | 35002_120_2011 | UnannotatedRegion |
| 428 | ACCGTTAAGG.ACAGGGTTAA | C | x | 35003_403_2011 | UnannotatedRegion |
| 428 | ACCGTTAAGG.ACAGGGTTAA | C | x | 35004_12_15398_XS2_1 | UnannotatedRegion |
| 428 | ACCGTTAAGG.ACAGGGTTAA | C | x | 35005_12_13952_XS2_1 | UnannotatedRegion |
| 428 | ACCGTTAAGG.ACAGGGTTAA | C | x | 35006_12_15317_XS2_1 | UnannotatedRegion |
| 428 | ACCGTTAAGG.ACAGGGTTAA | C | x | 35007_12_15047_XS2_1 | UnannotatedRegion |
| 428 | ACCGTTAAGG.ACAGGGTTAA | C | x | 35008_12_15661_XS2_1 | UnannotatedRegion |
| 428 | ACCGTTAAGG.ACAGGGTTAA | C | x | 35009_12_15657_XS2_1 | UnannotatedRegion |
| 428 | ACCGTTAAGG.ACAGGGTTAA | C | x | 35010_12_17186_XS2_1 | UnannotatedRegion |
| 428 | ACCGTTAAGG.ACAGGGTTAA | C | x | 35011_12_17194_XS2_1 | UnannotatedRegion |
| 428 | ACCGTTAAGG.ACAGGGTTAA | C | x | 35012_12_17009_XS2_1 | UnannotatedRegion |
| 428 | ACCGTTAAGG.ACAGGGTTAA | C | x | 35013_12_14973_XS2_1 | UnannotatedRegion |
|  |  |  |  |  |  |
| 438 | ACCTTTGCTC.ATCAAACATC | C | 1937775 F | 19260_WUE_2594 | UnannotatedRegion |
| 438 | ACCTTTGCTC.ATCAAACATC | T | x | 34990_Tchad54_11 | UnannotatedRegion |
| 438 | ACCTTTGCTC.ATCAAACATC | T | x | 34991_Tchad78_11 | UnannotatedRegion |
| 438 | ACCTTTGCTC.ATCAAACATC | T | x | 34992_Tchad95_11 | UnannotatedRegion |
| 438 | ACCTTTGCTC.ATCAAACATC | T | x | 34994_Tchad106_11 | UnannotatedRegion |
| 438 | ACCTTTGCTC.ATCAAACATC | C | x | 34995_8_2011 | UnannotatedRegion |
| 438 | ACCTTTGCTC.ATCAAACATC | C | x | 34996_10_2011 | UnannotatedRegion |
| 438 | ACCTTTGCTC.ATCAAACATC | T | x | 34997_39_2011 | UnannotatedRegion |
| 438 | ACCTTTGCTC.ATCAAACATC | T | x | 34998_59_2011 | UnannotatedRegion |
| 438 | ACCTTTGCTC.ATCAAACATC | T | x | 34999_63_2011 | UnannotatedRegion |
| 438 | ACCTTTGCTC.ATCAAACATC | T | x | 35000_75_2011 | UnannotatedRegion |
| 438 | ACCTTTGCTC.ATCAAACATC | T | x | 35001_76_2011 | UnannotatedRegion |
| 438 | ACCTTTGCTC.ATCAAACATC | T | x | 35002_120_2011 | UnannotatedRegion |
| 438 | ACCTTTGCTC.ATCAAACATC | T | x | 35003_403_2011 | UnannotatedRegion |
| 438 | ACCTTTGCTC.ATCAAACATC | T | x | 35004_12_15398_XS2_1 | UnannotatedRegion |
| 438 | ACCTTTGCTC.ATCAAACATC | T | x | 35005_12_13952_XS2_1 | UnannotatedRegion |
| 438 | ACCTTTGCTC.ATCAAACATC | T | x | 35006_12_15317_XS2_1 | UnannotatedRegion |
| 438 | ACCTTTGCTC.ATCAAACATC | T | x | 35007_12_15047_XS2_1 | UnannotatedRegion |
| 438 | ACCTTTGCTC.ATCAAACATC | T | x | 35008_12_15661_XS2_1 | UnannotatedRegion |
| 438 | ACCTTTGCTC.ATCAAACATC | T | x | 35009_12_15657_XS2_1 | UnannotatedRegion |
| 438 | ACCTTTGCTC.ATCAAACATC | T | x | 35010_12_17186_XS2_1 | UnannotatedRegion |
| 438 | ACCTTTGCTC.ATCAAACATC | T | x | 35011_12_17194_XS2_1 | UnannotatedRegion |
| 438 | ACCTTTGCTC.ATCAAACATC | T | x | 35012_12_17009_XS2_1 | UnannotatedRegion |
| 438 | ACCTTTGCTC.ATCAAACATC | T | x | 35013_12_14973_XS2_1 | UnannotatedRegion |
|  |  |  |  |  |  |
|  |  |  |  |  |  |
| 463 | ACGATGCAAA.GCAATGATGC | G | 2162420 F | 19260_WUE_2594 | UnannotatedRegion |
| 463 | ACGATGCAAA.GCAATGATGC | G | x | 34990_Tchad54_11 | UnannotatedRegion |
| 463 | ACGATGCAAA.GCAATGATGC | G | x | 34991_Tchad78_11 | UnannotatedRegion |
| 463 | ACGATGCAAA.GCAATGATGC | A | x | 34992_Tchad95_11 | UnannotatedRegion |
| 463 | ACGATGCAAA.GCAATGATGC | G | x | 34995_8_2011 | UnannotatedRegion |
| 463 | ACGATGCAAA.GCAATGATGC | G | x | 34996_10_2011 | UnannotatedRegion |
| 463 | ACGATGCAAA.GCAATGATGC | G | x | 34997_39_2011 | UnannotatedRegion |
| 463 | ACGATGCAAA.GCAATGATGC | G | x | 34998_59_2011 | UnannotatedRegion |
| 463 | ACGATGCAAA.GCAATGATGC | G | x | 34999_63_2011 | UnannotatedRegion |
| 463 | ACGATGCAAA.GCAATGATGC | G | x | 35000_75_2011 | UnannotatedRegion |
| 463 | ACGATGCAAA.GCAATGATGC | G | x | 35001_76_2011 | UnannotatedRegion |
| 463 | ACGATGCAAA.GCAATGATGC | G | x | 35002_120_2011 | UnannotatedRegion |
| 463 | ACGATGCAAA.GCAATGATGC | G | x | 35003_403_2011 | UnannotatedRegion |
| 463 | ACGATGCAAA.GCAATGATGC | G | x | 35004_12_15398_XS2_1 | UnannotatedRegion |
| 463 | ACGATGCAAA.GCAATGATGC | G | x | 35005_12_13952_XS2_1 | UnannotatedRegion |
| 463 | ACGATGCAAA.GCAATGATGC | G | x | 35006_12_15317_XS2_1 | UnannotatedRegion |
| 463 | ACGATGCAAA.GCAATGATGC | A | x | 35007_12_15047_XS2_1 | UnannotatedRegion |
| 463 | ACGATGCAAA.GCAATGATGC | A | x | 35008_12_15661_XS2_1 | UnannotatedRegion |
| 463 | ACGATGCAAA.GCAATGATGC | A | x | 35009_12_15657_XS2_1 | UnannotatedRegion |
| 463 | ACGATGCAAA.GCAATGATGC | G | x | 35010_12_17186_XS2_1 | UnannotatedRegion |
| 463 | ACGATGCAAA.GCAATGATGC | G | x | 35011_12_17194_XS2_1 | UnannotatedRegion |
| 463 | ACGATGCAAA.GCAATGATGC | G | x | 35012_12_17009_XS2_1 | UnannotatedRegion |
| 463 | ACGATGCAAA.GCAATGATGC | A | x | 35013_12_14973_XS2_1 | UnannotatedRegion |
|  |  |  |  |  |  |
| 489 | ACGGCATCGG.TGTTTATTTG | G | 1118268 F | 19260_WUE_2594 | UnannotatedRegion |
| 489 | ACGGCATCGG.TGTTTATTTG | A | x | 34990_Tchad54_11 | UnannotatedRegion |
| 489 | ACGGCATCGG.TGTTTATTTG | A | x | 34991_Tchad78_11 | UnannotatedRegion |
| 489 | ACGGCATCGG.TGTTTATTTG | A | x | 34992_Tchad95_11 | UnannotatedRegion |
| 489 | ACGGCATCGG.TGTTTATTTG | A | x | 34994_Tchad106_11 | UnannotatedRegion |
| 489 | ACGGCATCGG.TGTTTATTTG | A | x | 34995_8_2011 | UnannotatedRegion |
| 489 | ACGGCATCGG.TGTTTATTTG | A | x | 34996_10_2011 | UnannotatedRegion |
| 489 | ACGGCATCGG.TGTTTATTTG | A | x | 34997_39_2011 | UnannotatedRegion |
| 489 | ACGGCATCGG.TGTTTATTTG | A | x | 34998_59_2011 | UnannotatedRegion |
| 489 | ACGGCATCGG.TGTTTATTTG | A | x | 34999_63_2011 | UnannotatedRegion |
| 489 | ACGGCATCGG.TGTTTATTTG | A | x | 35000_75_2011 | UnannotatedRegion |
| 489 | ACGGCATCGG.TGTTTATTTG | A | x | 35001_76_2011 | UnannotatedRegion |
| 489 | ACGGCATCGG.TGTTTATTTG | A | x | 35002_120_2011 | UnannotatedRegion |
| 489 | ACGGCATCGG.TGTTTATTTG | A | x | 35003_403_2011 | UnannotatedRegion |
| 489 | ACGGCATCGG.TGTTTATTTG | A | x | 35004_12_15398_XS2_1 | UnannotatedRegion |
| 489 | ACGGCATCGG.TGTTTATTTG | A | x | 35005_12_13952_XS2_1 | UnannotatedRegion |
| 489 | ACGGCATCGG.TGTTTATTTG | A | x | 35006_12_15317_XS2_1 | UnannotatedRegion |
| 489 | ACGGCATCGG.TGTTTATTTG | A | x | 35007_12_15047_XS2_1 | UnannotatedRegion |
| 489 | ACGGCATCGG.TGTTTATTTG | A | x | 35008_12_15661_XS2_1 | UnannotatedRegion |
| 489 | ACGGCATCGG.TGTTTATTTG | A | x | 35009_12_15657_XS2_1 | UnannotatedRegion |
| 489 | ACGGCATCGG.TGTTTATTTG | A | x | 35010_12_17186_XS2_1 | UnannotatedRegion |
| 489 | ACGGCATCGG.TGTTTATTTG | A | x | 35011_12_17194_XS2_1 | UnannotatedRegion |
| 489 | ACGGCATCGG.TGTTTATTTG | A | x | 35012_12_17009_XS2_1 | UnannotatedRegion |
| 489 | ACGGCATCGG.TGTTTATTTG | A | x | 35013_12_14973_XS2_1 | UnannotatedRegion |
|  |  |  |  |  |  |
| 497 | ACGGCGGGAA.GGAAAGGGGC | A | 1214778 R | 19260_WUE_2594 | UnannotatedRegion |
| 497 | ACGGCGGGAA.GGAAAGGGGC | G | x | 34990_Tchad54_11 | UnannotatedRegion |
| 497 | ACGGCGGGAA.GGAAAGGGGC | G | x | 34991_Tchad78_11 | UnannotatedRegion |
| 497 | ACGGCGGGAA.GGAAAGGGGC | G | x | 34992_Tchad95_11 | UnannotatedRegion |
| 497 | ACGGCGGGAA.GGAAAGGGGC | G | x | 34994_Tchad106_11 | UnannotatedRegion |
| 497 | ACGGCGGGAA.GGAAAGGGGC | G | x | 34995_8_2011 | UnannotatedRegion |
| 497 | ACGGCGGGAA.GGAAAGGGGC | G | x | 34996_10_2011 | UnannotatedRegion |
| 497 | ACGGCGGGAA.GGAAAGGGGC | G | x | 34997_39_2011 | UnannotatedRegion |
| 497 | ACGGCGGGAA.GGAAAGGGGC | G | x | 34998_59_2011 | UnannotatedRegion |
| 497 | ACGGCGGGAA.GGAAAGGGGC | G | x | 34999_63_2011 | UnannotatedRegion |
| 497 | ACGGCGGGAA.GGAAAGGGGC | G | x | 35000_75_2011 | UnannotatedRegion |
| 497 | ACGGCGGGAA.GGAAAGGGGC | G | x | 35001_76_2011 | UnannotatedRegion |
| 497 | ACGGCGGGAA.GGAAAGGGGC | G | x | 35002_120_2011 | UnannotatedRegion |
| 497 | ACGGCGGGAA.GGAAAGGGGC | G | x | 35003_403_2011 | UnannotatedRegion |
| 497 | ACGGCGGGAA.GGAAAGGGGC | G | x | 35004_12_15398_XS2_1 | UnannotatedRegion |
| 497 | ACGGCGGGAA.GGAAAGGGGC | G | x | 35005_12_13952_XS2_1 | UnannotatedRegion |
| 497 | ACGGCGGGAA.GGAAAGGGGC | G | x | 35006_12_15317_XS2_1 | UnannotatedRegion |
| 497 | ACGGCGGGAA.GGAAAGGGGC | G | x | 35007_12_15047_XS2_1 | UnannotatedRegion |
| 497 | ACGGCGGGAA.GGAAAGGGGC | G | x | 35008_12_15661_XS2_1 | UnannotatedRegion |
| 497 | ACGGCGGGAA.GGAAAGGGGC | G | x | 35009_12_15657_XS2_1 | UnannotatedRegion |
| 497 | ACGGCGGGAA.GGAAAGGGGC | G | x | 35010_12_17186_XS2_1 | UnannotatedRegion |
| 497 | ACGGCGGGAA.GGAAAGGGGC | G | x | 35011_12_17194_XS2_1 | UnannotatedRegion |
| 497 | ACGGCGGGAA.GGAAAGGGGC | G | x | 35012_12_17009_XS2_1 | UnannotatedRegion |
| 497 | ACGGCGGGAA.GGAAAGGGGC | G | x | 35013_12_14973_XS2_1 | UnannotatedRegion |
|  |  |  |  |  |  |
| 521 | ACTATGGATG.GACTATACGC | C | 2034527 R | 19260_WUE_2594 | UnannotatedRegion |
| 521 | ACTATGGATG.GACTATACGC | A | x | 34990_Tchad54_11 | UnannotatedRegion |
| 521 | ACTATGGATG.GACTATACGC | A | x | 34991_Tchad78_11 | UnannotatedRegion |
| 521 | ACTATGGATG.GACTATACGC | A | x | 34992_Tchad95_11 | UnannotatedRegion |
| 521 | ACTATGGATG.GACTATACGC | A | x | 34995_8_2011 | UnannotatedRegion |
| 521 | ACTATGGATG.GACTATACGC | A | x | 34996_10_2011 | UnannotatedRegion |
| 521 | ACTATGGATG.GACTATACGC | A | x | 34997_39_2011 | UnannotatedRegion |
| 521 | ACTATGGATG.GACTATACGC | A | x | 34998_59_2011 | UnannotatedRegion |
| 521 | ACTATGGATG.GACTATACGC | A | x | 34999_63_2011 | UnannotatedRegion |
| 521 | ACTATGGATG.GACTATACGC | A | x | 35000_75_2011 | UnannotatedRegion |
| 521 | ACTATGGATG.GACTATACGC | A | x | 35001_76_2011 | UnannotatedRegion |
| 521 | ACTATGGATG.GACTATACGC | A | x | 35002_120_2011 | UnannotatedRegion |
| 521 | ACTATGGATG.GACTATACGC | A | x | 35003_403_2011 | UnannotatedRegion |
| 521 | ACTATGGATG.GACTATACGC | A | x | 35004_12_15398_XS2_1 | UnannotatedRegion |
| 521 | ACTATGGATG.GACTATACGC | A | x | 35005_12_13952_XS2_1 | UnannotatedRegion |
| 521 | ACTATGGATG.GACTATACGC | A | x | 35006_12_15317_XS2_1 | UnannotatedRegion |
| 521 | ACTATGGATG.GACTATACGC | A | x | 35007_12_15047_XS2_1 | UnannotatedRegion |
| 521 | ACTATGGATG.GACTATACGC | A | x | 35008_12_15661_XS2_1 | UnannotatedRegion |
| 521 | ACTATGGATG.GACTATACGC | A | x | 35009_12_15657_XS2_1 | UnannotatedRegion |
| 521 | ACTATGGATG.GACTATACGC | A | x | 35010_12_17186_XS2_1 | UnannotatedRegion |
| 521 | ACTATGGATG.GACTATACGC | A | x | 35011_12_17194_XS2_1 | UnannotatedRegion |
| 521 | ACTATGGATG.GACTATACGC | A | x | 35012_12_17009_XS2_1 | UnannotatedRegion |
| 521 | ACTATGGATG.GACTATACGC | A | x | 35013_12_14973_XS2_1 | UnannotatedRegion |
|  |  |  |  |  |  |
| 522 | ACTCAATTTA.CAAAACAACC | A | 1950992 F | 19260_WUE_2594 | UnannotatedRegion |
| 522 | ACTCAATTTA.CAAAACAACC | A | x | 34990_Tchad54_11 | UnannotatedRegion |
| 522 | ACTCAATTTA.CAAAACAACC | A | x | 34991_Tchad78_11 | UnannotatedRegion |
| 522 | ACTCAATTTA.CAAAACAACC | A | x | 34992_Tchad95_11 | UnannotatedRegion |
| 522 | ACTCAATTTA.CAAAACAACC | A | x | 34994_Tchad106_11 | UnannotatedRegion |
| 522 | ACTCAATTTA.CAAAACAACC | G | x | 34995_8_2011 | UnannotatedRegion |
| 522 | ACTCAATTTA.CAAAACAACC | G | x | 34996_10_2011 | UnannotatedRegion |
| 522 | ACTCAATTTA.CAAAACAACC | A | x | 34997_39_2011 | UnannotatedRegion |
| 522 | ACTCAATTTA.CAAAACAACC | A | x | 34998_59_2011 | UnannotatedRegion |
| 522 | ACTCAATTTA.CAAAACAACC | A | x | 34999_63_2011 | UnannotatedRegion |
| 522 | ACTCAATTTA.CAAAACAACC | A | x | 35000_75_2011 | UnannotatedRegion |
| 522 | ACTCAATTTA.CAAAACAACC | A | x | 35001_76_2011 | UnannotatedRegion |
| 522 | ACTCAATTTA.CAAAACAACC | A | x | 35002_120_2011 | UnannotatedRegion |
| 522 | ACTCAATTTA.CAAAACAACC | A | x | 35003_403_2011 | UnannotatedRegion |
| 522 | ACTCAATTTA.CAAAACAACC | A | x | 35004_12_15398_XS2_1 | UnannotatedRegion |
| 522 | ACTCAATTTA.CAAAACAACC | A | x | 35005_12_13952_XS2_1 | UnannotatedRegion |
| 522 | ACTCAATTTA.CAAAACAACC | A | x | 35006_12_15317_XS2_1 | UnannotatedRegion |
| 522 | ACTCAATTTA.CAAAACAACC | A | x | 35007_12_15047_XS2_1 | UnannotatedRegion |
| 522 | ACTCAATTTA.CAAAACAACC | A | x | 35008_12_15661_XS2_1 | UnannotatedRegion |
| 522 | ACTCAATTTA.CAAAACAACC | A | x | 35009_12_15657_XS2_1 | UnannotatedRegion |
| 522 | ACTCAATTTA.CAAAACAACC | A | x | 35010_12_17186_XS2_1 | UnannotatedRegion |
| 522 | ACTCAATTTA.CAAAACAACC | A | x | 35011_12_17194_XS2_1 | UnannotatedRegion |
| 522 | ACTCAATTTA.CAAAACAACC | A | x | 35012_12_17009_XS2_1 | UnannotatedRegion |
| 522 | ACTCAATTTA.CAAAACAACC | A | x | 35013_12_14973_XS2_1 | UnannotatedRegion |
|  |  |  |  |  |  |
| 551 | AGAATAAGGC.TCAGACGGCA | C | 1731044 R | 19260_WUE_2594 | UnannotatedRegion |
| 551 | AGAATAAGGC.TCAGACGGCA | C | x | 34990_Tchad54_11 | UnannotatedRegion |
| 551 | AGAATAAGGC.TCAGACGGCA | C | x | 34991_Tchad78_11 | UnannotatedRegion |
| 551 | AGAATAAGGC.TCAGACGGCA | C | x | 34992_Tchad95_11 | UnannotatedRegion |
| 551 | AGAATAAGGC.TCAGACGGCA | C | x | 34994_Tchad106_11 | UnannotatedRegion |
| 551 | AGAATAAGGC.TCAGACGGCA | C | x | 34995_8_2011 | UnannotatedRegion |
| 551 | AGAATAAGGC.TCAGACGGCA | C | x | 34996_10_2011 | UnannotatedRegion |
| 551 | AGAATAAGGC.TCAGACGGCA | C | x | 34997_39_2011 | UnannotatedRegion |
| 551 | AGAATAAGGC.TCAGACGGCA | C | x | 34998_59_2011 | UnannotatedRegion |
| 551 | AGAATAAGGC.TCAGACGGCA | C | x | 34999_63_2011 | UnannotatedRegion |
| 551 | AGAATAAGGC.TCAGACGGCA | C | x | 35000_75_2011 | UnannotatedRegion |
| 551 | AGAATAAGGC.TCAGACGGCA | C | x | 35001_76_2011 | UnannotatedRegion |
| 551 | AGAATAAGGC.TCAGACGGCA | C | x | 35002_120_2011 | UnannotatedRegion |
| 551 | AGAATAAGGC.TCAGACGGCA | C | x | 35003_403_2011 | UnannotatedRegion |
| 551 | AGAATAAGGC.TCAGACGGCA | C | x | 35004_12_15398_XS2_1 | UnannotatedRegion |
| 551 | AGAATAAGGC.TCAGACGGCA | C | x | 35005_12_13952_XS2_1 | UnannotatedRegion |
| 551 | AGAATAAGGC.TCAGACGGCA | C | x | 35006_12_15317_XS2_1 | UnannotatedRegion |
| 551 | AGAATAAGGC.TCAGACGGCA | T | x | 35007_12_15047_XS2_1 | UnannotatedRegion |
| 551 | AGAATAAGGC.TCAGACGGCA | C | x | 35008_12_15661_XS2_1 | UnannotatedRegion |
| 551 | AGAATAAGGC.TCAGACGGCA | C | x | 35009_12_15657_XS2_1 | UnannotatedRegion |
| 551 | AGAATAAGGC.TCAGACGGCA | C | x | 35010_12_17186_XS2_1 | UnannotatedRegion |
| 551 | AGAATAAGGC.TCAGACGGCA | C | x | 35011_12_17194_XS2_1 | UnannotatedRegion |
| 551 | AGAATAAGGC.TCAGACGGCA | C | x | 35012_12_17009_XS2_1 | UnannotatedRegion |
| 551 | AGAATAAGGC.TCAGACGGCA | C | x | 35013_12_14973_XS2_1 | UnannotatedRegion |
|  |  |  |  |  |  |
| 554 | AGACAGGAAA.ACACACACTG | A | 1144637 R | 19260_WUE_2594 | UnannotatedRegion |
| 554 | AGACAGGAAA.ACACACACTG | G | x | 34990_Tchad54_11 | UnannotatedRegion |
| 554 | AGACAGGAAA.ACACACACTG | G | x | 34991_Tchad78_11 | UnannotatedRegion |
| 554 | AGACAGGAAA.ACACACACTG | G | x | 34992_Tchad95_11 | UnannotatedRegion |
| 554 | AGACAGGAAA.ACACACACTG | G | x | 34994_Tchad106_11 | UnannotatedRegion |
| 554 | AGACAGGAAA.ACACACACTG | G | x | 34995_8_2011 | UnannotatedRegion |
| 554 | AGACAGGAAA.ACACACACTG | G | x | 34996_10_2011 | UnannotatedRegion |
| 554 | AGACAGGAAA.ACACACACTG | G | x | 34997_39_2011 | UnannotatedRegion |
| 554 | AGACAGGAAA.ACACACACTG | G | x | 34998_59_2011 | UnannotatedRegion |
| 554 | AGACAGGAAA.ACACACACTG | G | x | 34999_63_2011 | UnannotatedRegion |
| 554 | AGACAGGAAA.ACACACACTG | G | x | 35000_75_2011 | UnannotatedRegion |
| 554 | AGACAGGAAA.ACACACACTG | G | x | 35001_76_2011 | UnannotatedRegion |
| 554 | AGACAGGAAA.ACACACACTG | G | x | 35002_120_2011 | UnannotatedRegion |
| 554 | AGACAGGAAA.ACACACACTG | G | x | 35003_403_2011 | UnannotatedRegion |
| 554 | AGACAGGAAA.ACACACACTG | G | x | 35004_12_15398_XS2_1 | UnannotatedRegion |
| 554 | AGACAGGAAA.ACACACACTG | G | x | 35005_12_13952_XS2_1 | UnannotatedRegion |
| 554 | AGACAGGAAA.ACACACACTG | G | x | 35006_12_15317_XS2_1 | UnannotatedRegion |
| 554 | AGACAGGAAA.ACACACACTG | G | x | 35007_12_15047_XS2_1 | UnannotatedRegion |
| 554 | AGACAGGAAA.ACACACACTG | G | x | 35008_12_15661_XS2_1 | UnannotatedRegion |
| 554 | AGACAGGAAA.ACACACACTG | G | x | 35009_12_15657_XS2_1 | UnannotatedRegion |
| 554 | AGACAGGAAA.ACACACACTG | G | x | 35010_12_17186_XS2_1 | UnannotatedRegion |
| 554 | AGACAGGAAA.ACACACACTG | G | x | 35011_12_17194_XS2_1 | UnannotatedRegion |
| 554 | AGACAGGAAA.ACACACACTG | G | x | 35012_12_17009_XS2_1 | UnannotatedRegion |
| 554 | AGACAGGAAA.ACACACACTG | G | x | 35013_12_14973_XS2_1 | UnannotatedRegion |
|  |  |  |  |  |  |
| 557 | AGACGGCATC.CCAATCCGCC | C | 2072539 F | 19260_WUE_2594 | UnannotatedRegion |
| 557 | AGACGGCATC.CCAATCCGCC | T | x | 34990_Tchad54_11 | UnannotatedRegion |
| 557 | AGACGGCATC.CCAATCCGCC | T | x | 34991_Tchad78_11 | UnannotatedRegion |
| 557 | AGACGGCATC.CCAATCCGCC | T | x | 34992_Tchad95_11 | UnannotatedRegion |
| 557 | AGACGGCATC.CCAATCCGCC | T | x | 34994_Tchad106_11 | UnannotatedRegion |
| 557 | AGACGGCATC.CCAATCCGCC | T | x | 34995_8_2011 | UnannotatedRegion |
| 557 | AGACGGCATC.CCAATCCGCC | T | x | 34996_10_2011 | UnannotatedRegion |
| 557 | AGACGGCATC.CCAATCCGCC | T | x | 34997_39_2011 | UnannotatedRegion |
| 557 | AGACGGCATC.CCAATCCGCC | T | x | 34998_59_2011 | UnannotatedRegion |
| 557 | AGACGGCATC.CCAATCCGCC | T | x | 34999_63_2011 | UnannotatedRegion |
| 557 | AGACGGCATC.CCAATCCGCC | T | x | 35000_75_2011 | UnannotatedRegion |
| 557 | AGACGGCATC.CCAATCCGCC | T | x | 35001_76_2011 | UnannotatedRegion |
| 557 | AGACGGCATC.CCAATCCGCC | T | x | 35002_120_2011 | UnannotatedRegion |
| 557 | AGACGGCATC.CCAATCCGCC | T | x | 35003_403_2011 | UnannotatedRegion |
| 557 | AGACGGCATC.CCAATCCGCC | T | x | 35004_12_15398_XS2_1 | UnannotatedRegion |
| 557 | AGACGGCATC.CCAATCCGCC | T | x | 35005_12_13952_XS2_1 | UnannotatedRegion |
| 557 | AGACGGCATC.CCAATCCGCC | T | x | 35006_12_15317_XS2_1 | UnannotatedRegion |
| 557 | AGACGGCATC.CCAATCCGCC | T | x | 35007_12_15047_XS2_1 | UnannotatedRegion |
| 557 | AGACGGCATC.CCAATCCGCC | T | x | 35008_12_15661_XS2_1 | UnannotatedRegion |
| 557 | AGACGGCATC.CCAATCCGCC | T | x | 35009_12_15657_XS2_1 | UnannotatedRegion |
| 557 | AGACGGCATC.CCAATCCGCC | T | x | 35010_12_17186_XS2_1 | UnannotatedRegion |
| 557 | AGACGGCATC.CCAATCCGCC | T | x | 35011_12_17194_XS2_1 | UnannotatedRegion |
| 557 | AGACGGCATC.CCAATCCGCC | T | x | 35012_12_17009_XS2_1 | UnannotatedRegion |
| 557 | AGACGGCATC.CCAATCCGCC | T | x | 35013_12_14973_XS2_1 | UnannotatedRegion |
|  |  |  |  |  |  |
| 558 | AGACGGCATC.GGGAGGCGCG | G | 1146379 F | 19260_WUE_2594 | UnannotatedRegion |
| 558 | AGACGGCATC.GGGAGGCGCG | T | x | 34990_Tchad54_11 | UnannotatedRegion |
| 558 | AGACGGCATC.GGGAGGCGCG | T | x | 34991_Tchad78_11 | UnannotatedRegion |
| 558 | AGACGGCATC.GGGAGGCGCG | T | x | 34992_Tchad95_11 | UnannotatedRegion |
| 558 | AGACGGCATC.GGGAGGCGCG | T | x | 34994_Tchad106_11 | UnannotatedRegion |
| 558 | AGACGGCATC.GGGAGGCGCG | T | x | 34995_8_2011 | UnannotatedRegion |
| 558 | AGACGGCATC.GGGAGGCGCG | T | x | 34996_10_2011 | UnannotatedRegion |
| 558 | AGACGGCATC.GGGAGGCGCG | T | x | 34997_39_2011 | UnannotatedRegion |
| 558 | AGACGGCATC.GGGAGGCGCG | T | x | 34998_59_2011 | UnannotatedRegion |
| 558 | AGACGGCATC.GGGAGGCGCG | T | x | 34999_63_2011 | UnannotatedRegion |
| 558 | AGACGGCATC.GGGAGGCGCG | T | x | 35000_75_2011 | UnannotatedRegion |
| 558 | AGACGGCATC.GGGAGGCGCG | T | x | 35001_76_2011 | UnannotatedRegion |
| 558 | AGACGGCATC.GGGAGGCGCG | T | x | 35002_120_2011 | UnannotatedRegion |
| 558 | AGACGGCATC.GGGAGGCGCG | T | x | 35003_403_2011 | UnannotatedRegion |
| 558 | AGACGGCATC.GGGAGGCGCG | T | x | 35004_12_15398_XS2_1 | UnannotatedRegion |
| 558 | AGACGGCATC.GGGAGGCGCG | T | x | 35005_12_13952_XS2_1 | UnannotatedRegion |
| 558 | AGACGGCATC.GGGAGGCGCG | T | x | 35006_12_15317_XS2_1 | UnannotatedRegion |
| 558 | AGACGGCATC.GGGAGGCGCG | T | x | 35007_12_15047_XS2_1 | UnannotatedRegion |
| 558 | AGACGGCATC.GGGAGGCGCG | T | x | 35008_12_15661_XS2_1 | UnannotatedRegion |
| 558 | AGACGGCATC.GGGAGGCGCG | T | x | 35009_12_15657_XS2_1 | UnannotatedRegion |
| 558 | AGACGGCATC.GGGAGGCGCG | T | x | 35010_12_17186_XS2_1 | UnannotatedRegion |
| 558 | AGACGGCATC.GGGAGGCGCG | T | x | 35011_12_17194_XS2_1 | UnannotatedRegion |
| 558 | AGACGGCATC.GGGAGGCGCG | T | x | 35012_12_17009_XS2_1 | UnannotatedRegion |
| 558 | AGACGGCATC.GGGAGGCGCG | T | x | 35013_12_14973_XS2_1 | UnannotatedRegion |
|  |  |  |  |  |  |
| 559 | AGACGGCATC.GGTTCGGCAT | G | 1118237 R | 19260_WUE_2594 | UnannotatedRegion |
| 559 | AGACGGCATC.GGTTCGGCAT | A | x | 34990_Tchad54_11 | UnannotatedRegion |
| 559 | AGACGGCATC.GGTTCGGCAT | A | x | 34991_Tchad78_11 | UnannotatedRegion |
| 559 | AGACGGCATC.GGTTCGGCAT | A | x | 34992_Tchad95_11 | UnannotatedRegion |
| 559 | AGACGGCATC.GGTTCGGCAT | A | x | 34994_Tchad106_11 | UnannotatedRegion |
| 559 | AGACGGCATC.GGTTCGGCAT | A | x | 34995_8_2011 | UnannotatedRegion |
| 559 | AGACGGCATC.GGTTCGGCAT | A | x | 34996_10_2011 | UnannotatedRegion |
| 559 | AGACGGCATC.GGTTCGGCAT | A | x | 34997_39_2011 | UnannotatedRegion |
| 559 | AGACGGCATC.GGTTCGGCAT | A | x | 34998_59_2011 | UnannotatedRegion |
| 559 | AGACGGCATC.GGTTCGGCAT | A | x | 34999_63_2011 | UnannotatedRegion |
| 559 | AGACGGCATC.GGTTCGGCAT | A | x | 35000_75_2011 | UnannotatedRegion |
| 559 | AGACGGCATC.GGTTCGGCAT | A | x | 35001_76_2011 | UnannotatedRegion |
| 559 | AGACGGCATC.GGTTCGGCAT | A | x | 35002_120_2011 | UnannotatedRegion |
| 559 | AGACGGCATC.GGTTCGGCAT | A | x | 35003_403_2011 | UnannotatedRegion |
| 559 | AGACGGCATC.GGTTCGGCAT | A | x | 35004_12_15398_XS2_1 | UnannotatedRegion |
| 559 | AGACGGCATC.GGTTCGGCAT | A | x | 35005_12_13952_XS2_1 | UnannotatedRegion |
| 559 | AGACGGCATC.GGTTCGGCAT | A | x | 35006_12_15317_XS2_1 | UnannotatedRegion |
| 559 | AGACGGCATC.GGTTCGGCAT | A | x | 35007_12_15047_XS2_1 | UnannotatedRegion |
| 559 | AGACGGCATC.GGTTCGGCAT | A | x | 35008_12_15661_XS2_1 | UnannotatedRegion |
| 559 | AGACGGCATC.GGTTCGGCAT | A | x | 35009_12_15657_XS2_1 | UnannotatedRegion |
| 559 | AGACGGCATC.GGTTCGGCAT | A | x | 35010_12_17186_XS2_1 | UnannotatedRegion |
| 559 | AGACGGCATC.GGTTCGGCAT | A | x | 35011_12_17194_XS2_1 | UnannotatedRegion |
| 559 | AGACGGCATC.GGTTCGGCAT | A | x | 35012_12_17009_XS2_1 | UnannotatedRegion |
| 559 | AGACGGCATC.GGTTCGGCAT | A | x | 35013_12_14973_XS2_1 | UnannotatedRegion |
|  |  |  |  |  |  |
| 560 | AGACGGCATT.TGGCTCAGGG | T | 1621314 R | 19260_WUE_2594 | UnannotatedRegion |
| 560 | AGACGGCATT.TGGCTCAGGG | G | x | 34990_Tchad54_11 | UnannotatedRegion |
| 560 | AGACGGCATT.TGGCTCAGGG | G | x | 34991_Tchad78_11 | UnannotatedRegion |
| 560 | AGACGGCATT.TGGCTCAGGG | G | x | 34992_Tchad95_11 | UnannotatedRegion |
| 560 | AGACGGCATT.TGGCTCAGGG | G | x | 34994_Tchad106_11 | UnannotatedRegion |
| 560 | AGACGGCATT.TGGCTCAGGG | G | x | 34995_8_2011 | UnannotatedRegion |
| 560 | AGACGGCATT.TGGCTCAGGG | G | x | 34996_10_2011 | UnannotatedRegion |
| 560 | AGACGGCATT.TGGCTCAGGG | G | x | 34997_39_2011 | UnannotatedRegion |
| 560 | AGACGGCATT.TGGCTCAGGG | G | x | 34998_59_2011 | UnannotatedRegion |
| 560 | AGACGGCATT.TGGCTCAGGG | G | x | 34999_63_2011 | UnannotatedRegion |
| 560 | AGACGGCATT.TGGCTCAGGG | G | x | 35000_75_2011 | UnannotatedRegion |
| 560 | AGACGGCATT.TGGCTCAGGG | G | x | 35001_76_2011 | UnannotatedRegion |
| 560 | AGACGGCATT.TGGCTCAGGG | G | x | 35002_120_2011 | UnannotatedRegion |
| 560 | AGACGGCATT.TGGCTCAGGG | G | x | 35003_403_2011 | UnannotatedRegion |
| 560 | AGACGGCATT.TGGCTCAGGG | G | x | 35004_12_15398_XS2_1 | UnannotatedRegion |
| 560 | AGACGGCATT.TGGCTCAGGG | G | x | 35005_12_13952_XS2_1 | UnannotatedRegion |
| 560 | AGACGGCATT.TGGCTCAGGG | G | x | 35006_12_15317_XS2_1 | UnannotatedRegion |
| 560 | AGACGGCATT.TGGCTCAGGG | G | x | 35007_12_15047_XS2_1 | UnannotatedRegion |
| 560 | AGACGGCATT.TGGCTCAGGG | G | x | 35008_12_15661_XS2_1 | UnannotatedRegion |
| 560 | AGACGGCATT.TGGCTCAGGG | G | x | 35009_12_15657_XS2_1 | UnannotatedRegion |
| 560 | AGACGGCATT.TGGCTCAGGG | G | x | 35010_12_17186_XS2_1 | UnannotatedRegion |
| 560 | AGACGGCATT.TGGCTCAGGG | G | x | 35011_12_17194_XS2_1 | UnannotatedRegion |
| 560 | AGACGGCATT.TGGCTCAGGG | G | x | 35012_12_17009_XS2_1 | UnannotatedRegion |
| 560 | AGACGGCATT.TGGCTCAGGG | G | x | 35013_12_14973_XS2_1 | UnannotatedRegion |
|  |  |  |  |  |  |
| 565 | AGAGGCGGTT.ATAATCAGCG | G | 856365 R | 19260_WUE_2594 | UnannotatedRegion |
| 565 | AGAGGCGGTT.ATAATCAGCG | C | x | 34990_Tchad54_11 | UnannotatedRegion |
| 565 | AGAGGCGGTT.ATAATCAGCG | C | x | 34991_Tchad78_11 | UnannotatedRegion |
| 565 | AGAGGCGGTT.ATAATCAGCG | C | x | 34992_Tchad95_11 | UnannotatedRegion |
| 565 | AGAGGCGGTT.ATAATCAGCG | C | x | 34994_Tchad106_11 | UnannotatedRegion |
| 565 | AGAGGCGGTT.ATAATCAGCG | C | x | 34995_8_2011 | UnannotatedRegion |
| 565 | AGAGGCGGTT.ATAATCAGCG | C | x | 34996_10_2011 | UnannotatedRegion |
| 565 | AGAGGCGGTT.ATAATCAGCG | C | x | 34997_39_2011 | UnannotatedRegion |
| 565 | AGAGGCGGTT.ATAATCAGCG | C | x | 34998_59_2011 | UnannotatedRegion |
| 565 | AGAGGCGGTT.ATAATCAGCG | C | x | 34999_63_2011 | UnannotatedRegion |
| 565 | AGAGGCGGTT.ATAATCAGCG | C | x | 35000_75_2011 | UnannotatedRegion |
| 565 | AGAGGCGGTT.ATAATCAGCG | C | x | 35001_76_2011 | UnannotatedRegion |
| 565 | AGAGGCGGTT.ATAATCAGCG | C | x | 35002_120_2011 | UnannotatedRegion |
| 565 | AGAGGCGGTT.ATAATCAGCG | C | x | 35003_403_2011 | UnannotatedRegion |
| 565 | AGAGGCGGTT.ATAATCAGCG | C | x | 35004_12_15398_XS2_1 | UnannotatedRegion |
| 565 | AGAGGCGGTT.ATAATCAGCG | C | x | 35005_12_13952_XS2_1 | UnannotatedRegion |
| 565 | AGAGGCGGTT.ATAATCAGCG | C | x | 35006_12_15317_XS2_1 | UnannotatedRegion |
| 565 | AGAGGCGGTT.ATAATCAGCG | C | x | 35007_12_15047_XS2_1 | UnannotatedRegion |
| 565 | AGAGGCGGTT.ATAATCAGCG | C | x | 35008_12_15661_XS2_1 | UnannotatedRegion |
| 565 | AGAGGCGGTT.ATAATCAGCG | C | x | 35009_12_15657_XS2_1 | UnannotatedRegion |
| 565 | AGAGGCGGTT.ATAATCAGCG | C | x | 35010_12_17186_XS2_1 | UnannotatedRegion |
| 565 | AGAGGCGGTT.ATAATCAGCG | C | x | 35011_12_17194_XS2_1 | UnannotatedRegion |
| 565 | AGAGGCGGTT.ATAATCAGCG | C | x | 35012_12_17009_XS2_1 | UnannotatedRegion |
| 565 | AGAGGCGGTT.ATAATCAGCG | C | x | 35013_12_14973_XS2_1 | UnannotatedRegion |
|  |  |  |  |  |  |
| 572 | AGATGATGCC.GGAAACATCA | A | x | 34990_Tchad54_11 | NotInAnnotatedGenome |
| 572 | AGATGATGCC.GGAAACATCA | G | x | 34991_Tchad78_11 | NotInAnnotatedGenome |
| 572 | AGATGATGCC.GGAAACATCA | G | x | 34992_Tchad95_11 | NotInAnnotatedGenome |
| 572 | AGATGATGCC.GGAAACATCA | G | x | 34994_Tchad106_11 | NotInAnnotatedGenome |
| 572 | AGATGATGCC.GGAAACATCA | G | x | 34995_8_2011 | NotInAnnotatedGenome |
| 572 | AGATGATGCC.GGAAACATCA | G | x | 34996_10_2011 | NotInAnnotatedGenome |
| 572 | AGATGATGCC.GGAAACATCA | A | x | 34997_39_2011 | NotInAnnotatedGenome |
| 572 | AGATGATGCC.GGAAACATCA | A | x | 34998_59_2011 | NotInAnnotatedGenome |
| 572 | AGATGATGCC.GGAAACATCA | A | x | 34999_63_2011 | NotInAnnotatedGenome |
| 572 | AGATGATGCC.GGAAACATCA | A | x | 35000_75_2011 | NotInAnnotatedGenome |
| 572 | AGATGATGCC.GGAAACATCA | A | x | 35001_76_2011 | NotInAnnotatedGenome |
| 572 | AGATGATGCC.GGAAACATCA | A | x | 35002_120_2011 | NotInAnnotatedGenome |
| 572 | AGATGATGCC.GGAAACATCA | G | x | 35003_403_2011 | NotInAnnotatedGenome |
| 572 | AGATGATGCC.GGAAACATCA | A | x | 35004_12_15398_XS2_1 | NotInAnnotatedGenome |
| 572 | AGATGATGCC.GGAAACATCA | A | x | 35005_12_13952_XS2_1 | NotInAnnotatedGenome |
| 572 | AGATGATGCC.GGAAACATCA | A | x | 35006_12_15317_XS2_1 | NotInAnnotatedGenome |
| 572 | AGATGATGCC.GGAAACATCA | G | x | 35007_12_15047_XS2_1 | NotInAnnotatedGenome |
| 572 | AGATGATGCC.GGAAACATCA | G | x | 35008_12_15661_XS2_1 | NotInAnnotatedGenome |
| 572 | AGATGATGCC.GGAAACATCA | G | x | 35009_12_15657_XS2_1 | NotInAnnotatedGenome |
| 572 | AGATGATGCC.GGAAACATCA | A | x | 35010_12_17186_XS2_1 | NotInAnnotatedGenome |
| 572 | AGATGATGCC.GGAAACATCA | A | x | 35011_12_17194_XS2_1 | NotInAnnotatedGenome |
| 572 | AGATGATGCC.GGAAACATCA | A | x | 35012_12_17009_XS2_1 | NotInAnnotatedGenome |
| 572 | AGATGATGCC.GGAAACATCA | G | x | 35013_12_14973_XS2_1 | NotInAnnotatedGenome |
|  |  |  |  |  |  |
| 589 | AGCCCTTCCA.AGTACATAGA | T | 460182 F | 19260_WUE_2594 | UnannotatedRegion |
| 589 | AGCCCTTCCA.AGTACATAGA | C | x | 34990_Tchad54_11 | UnannotatedRegion |
| 589 | AGCCCTTCCA.AGTACATAGA | C | x | 34991_Tchad78_11 | UnannotatedRegion |
| 589 | AGCCCTTCCA.AGTACATAGA | C | x | 34992_Tchad95_11 | UnannotatedRegion |
| 589 | AGCCCTTCCA.AGTACATAGA | C | x | 34994_Tchad106_11 | UnannotatedRegion |
| 589 | AGCCCTTCCA.AGTACATAGA | C | x | 34995_8_2011 | UnannotatedRegion |
| 589 | AGCCCTTCCA.AGTACATAGA | C | x | 34996_10_2011 | UnannotatedRegion |
| 589 | AGCCCTTCCA.AGTACATAGA | C | x | 34997_39_2011 | UnannotatedRegion |
| 589 | AGCCCTTCCA.AGTACATAGA | C | x | 34998_59_2011 | UnannotatedRegion |
| 589 | AGCCCTTCCA.AGTACATAGA | C | x | 34999_63_2011 | UnannotatedRegion |
| 589 | AGCCCTTCCA.AGTACATAGA | C | x | 35000_75_2011 | UnannotatedRegion |
| 589 | AGCCCTTCCA.AGTACATAGA | C | x | 35001_76_2011 | UnannotatedRegion |
| 589 | AGCCCTTCCA.AGTACATAGA | C | x | 35002_120_2011 | UnannotatedRegion |
| 589 | AGCCCTTCCA.AGTACATAGA | C | x | 35003_403_2011 | UnannotatedRegion |
| 589 | AGCCCTTCCA.AGTACATAGA | C | x | 35004_12_15398_XS2_1 | UnannotatedRegion |
| 589 | AGCCCTTCCA.AGTACATAGA | C | x | 35005_12_13952_XS2_1 | UnannotatedRegion |
| 589 | AGCCCTTCCA.AGTACATAGA | C | x | 35006_12_15317_XS2_1 | UnannotatedRegion |
| 589 | AGCCCTTCCA.AGTACATAGA | C | x | 35007_12_15047_XS2_1 | UnannotatedRegion |
| 589 | AGCCCTTCCA.AGTACATAGA | C | x | 35008_12_15661_XS2_1 | UnannotatedRegion |
| 589 | AGCCCTTCCA.AGTACATAGA | C | x | 35009_12_15657_XS2_1 | UnannotatedRegion |
| 589 | AGCCCTTCCA.AGTACATAGA | C | x | 35010_12_17186_XS2_1 | UnannotatedRegion |
| 589 | AGCCCTTCCA.AGTACATAGA | C | x | 35011_12_17194_XS2_1 | UnannotatedRegion |
| 589 | AGCCCTTCCA.AGTACATAGA | C | x | 35012_12_17009_XS2_1 | UnannotatedRegion |
| 589 | AGCCCTTCCA.AGTACATAGA | C | x | 35013_12_14973_XS2_1 | UnannotatedRegion |
|  |  |  |  |  |  |
| 600 | AGCCTGAAAC.GTGTGGGCAT | T | 1303649 F | 19260_WUE_2594 | UnannotatedRegion |
| 600 | AGCCTGAAAC.GTGTGGGCAT | C | x | 34990_Tchad54_11 | UnannotatedRegion |
| 600 | AGCCTGAAAC.GTGTGGGCAT | C | x | 34991_Tchad78_11 | UnannotatedRegion |
| 600 | AGCCTGAAAC.GTGTGGGCAT | C | x | 34992_Tchad95_11 | UnannotatedRegion |
| 600 | AGCCTGAAAC.GTGTGGGCAT | C | x | 34994_Tchad106_11 | UnannotatedRegion |
| 600 | AGCCTGAAAC.GTGTGGGCAT | C | x | 34995_8_2011 | UnannotatedRegion |
| 600 | AGCCTGAAAC.GTGTGGGCAT | C | x | 34996_10_2011 | UnannotatedRegion |
| 600 | AGCCTGAAAC.GTGTGGGCAT | C | x | 34997_39_2011 | UnannotatedRegion |
| 600 | AGCCTGAAAC.GTGTGGGCAT | C | x | 34998_59_2011 | UnannotatedRegion |
| 600 | AGCCTGAAAC.GTGTGGGCAT | C | x | 34999_63_2011 | UnannotatedRegion |
| 600 | AGCCTGAAAC.GTGTGGGCAT | C | x | 35000_75_2011 | UnannotatedRegion |
| 600 | AGCCTGAAAC.GTGTGGGCAT | C | x | 35001_76_2011 | UnannotatedRegion |
| 600 | AGCCTGAAAC.GTGTGGGCAT | C | x | 35002_120_2011 | UnannotatedRegion |
| 600 | AGCCTGAAAC.GTGTGGGCAT | C | x | 35003_403_2011 | UnannotatedRegion |
| 600 | AGCCTGAAAC.GTGTGGGCAT | C | x | 35004_12_15398_XS2_1 | UnannotatedRegion |
| 600 | AGCCTGAAAC.GTGTGGGCAT | C | x | 35005_12_13952_XS2_1 | UnannotatedRegion |
| 600 | AGCCTGAAAC.GTGTGGGCAT | C | x | 35006_12_15317_XS2_1 | UnannotatedRegion |
| 600 | AGCCTGAAAC.GTGTGGGCAT | C | x | 35007_12_15047_XS2_1 | UnannotatedRegion |
| 600 | AGCCTGAAAC.GTGTGGGCAT | C | x | 35008_12_15661_XS2_1 | UnannotatedRegion |
| 600 | AGCCTGAAAC.GTGTGGGCAT | C | x | 35009_12_15657_XS2_1 | UnannotatedRegion |
| 600 | AGCCTGAAAC.GTGTGGGCAT | C | x | 35010_12_17186_XS2_1 | UnannotatedRegion |
| 600 | AGCCTGAAAC.GTGTGGGCAT | C | x | 35011_12_17194_XS2_1 | UnannotatedRegion |
| 600 | AGCCTGAAAC.GTGTGGGCAT | C | x | 35012_12_17009_XS2_1 | UnannotatedRegion |
| 600 | AGCCTGAAAC.GTGTGGGCAT | C | x | 35013_12_14973_XS2_1 | UnannotatedRegion |
|  |  |  |  |  |  |
| 613 | AGCGGTTCAG.CGGCATTTCC | G | 1108539 R | 19260_WUE_2594 | UnannotatedRegion |
| 613 | AGCGGTTCAG.CGGCATTTCC | G | x | 34990_Tchad54_11 | UnannotatedRegion |
| 613 | AGCGGTTCAG.CGGCATTTCC | A | x | 34991_Tchad78_11 | UnannotatedRegion |
| 613 | AGCGGTTCAG.CGGCATTTCC | G | x | 34992_Tchad95_11 | UnannotatedRegion |
| 613 | AGCGGTTCAG.CGGCATTTCC | G | x | 34994_Tchad106_11 | UnannotatedRegion |
| 613 | AGCGGTTCAG.CGGCATTTCC | G | x | 34995_8_2011 | UnannotatedRegion |
| 613 | AGCGGTTCAG.CGGCATTTCC | G | x | 34996_10_2011 | UnannotatedRegion |
| 613 | AGCGGTTCAG.CGGCATTTCC | G | x | 34997_39_2011 | UnannotatedRegion |
| 613 | AGCGGTTCAG.CGGCATTTCC | G | x | 34998_59_2011 | UnannotatedRegion |
| 613 | AGCGGTTCAG.CGGCATTTCC | G | x | 34999_63_2011 | UnannotatedRegion |
| 613 | AGCGGTTCAG.CGGCATTTCC | G | x | 35000_75_2011 | UnannotatedRegion |
| 613 | AGCGGTTCAG.CGGCATTTCC | G | x | 35001_76_2011 | UnannotatedRegion |
| 613 | AGCGGTTCAG.CGGCATTTCC | G | x | 35002_120_2011 | UnannotatedRegion |
| 613 | AGCGGTTCAG.CGGCATTTCC | G | x | 35003_403_2011 | UnannotatedRegion |
| 613 | AGCGGTTCAG.CGGCATTTCC | G | x | 35004_12_15398_XS2_1 | UnannotatedRegion |
| 613 | AGCGGTTCAG.CGGCATTTCC | G | x | 35005_12_13952_XS2_1 | UnannotatedRegion |
| 613 | AGCGGTTCAG.CGGCATTTCC | G | x | 35006_12_15317_XS2_1 | UnannotatedRegion |
| 613 | AGCGGTTCAG.CGGCATTTCC | G | x | 35007_12_15047_XS2_1 | UnannotatedRegion |
| 613 | AGCGGTTCAG.CGGCATTTCC | G | x | 35008_12_15661_XS2_1 | UnannotatedRegion |
| 613 | AGCGGTTCAG.CGGCATTTCC | G | x | 35009_12_15657_XS2_1 | UnannotatedRegion |
| 613 | AGCGGTTCAG.CGGCATTTCC | G | x | 35010_12_17186_XS2_1 | UnannotatedRegion |
| 613 | AGCGGTTCAG.CGGCATTTCC | G | x | 35011_12_17194_XS2_1 | UnannotatedRegion |
| 613 | AGCGGTTCAG.CGGCATTTCC | G | x | 35012_12_17009_XS2_1 | UnannotatedRegion |
| 613 | AGCGGTTCAG.CGGCATTTCC | G | x | 35013_12_14973_XS2_1 | UnannotatedRegion |
|  |  |  |  |  |  |
| 644 | AGGGAACAAA.ACGCCTGATA | T | 504969 R | 19260_WUE_2594 | UnannotatedRegion |
| 644 | AGGGAACAAA.ACGCCTGATA | G | x | 34990_Tchad54_11 | UnannotatedRegion |
| 644 | AGGGAACAAA.ACGCCTGATA | T | x | 34991_Tchad78_11 | UnannotatedRegion |
| 644 | AGGGAACAAA.ACGCCTGATA | T | x | 34992_Tchad95_11 | UnannotatedRegion |
| 644 | AGGGAACAAA.ACGCCTGATA | T | x | 34994_Tchad106_11 | UnannotatedRegion |
| 644 | AGGGAACAAA.ACGCCTGATA | T | x | 34995_8_2011 | UnannotatedRegion |
| 644 | AGGGAACAAA.ACGCCTGATA | T | x | 34996_10_2011 | UnannotatedRegion |
| 644 | AGGGAACAAA.ACGCCTGATA | G | x | 34997_39_2011 | UnannotatedRegion |
| 644 | AGGGAACAAA.ACGCCTGATA | G | x | 34998_59_2011 | UnannotatedRegion |
| 644 | AGGGAACAAA.ACGCCTGATA | G | x | 34999_63_2011 | UnannotatedRegion |
| 644 | AGGGAACAAA.ACGCCTGATA | G | x | 35000_75_2011 | UnannotatedRegion |
| 644 | AGGGAACAAA.ACGCCTGATA | G | x | 35001_76_2011 | UnannotatedRegion |
| 644 | AGGGAACAAA.ACGCCTGATA | G | x | 35002_120_2011 | UnannotatedRegion |
| 644 | AGGGAACAAA.ACGCCTGATA | T | x | 35003_403_2011 | UnannotatedRegion |
| 644 | AGGGAACAAA.ACGCCTGATA | G | x | 35004_12_15398_XS2_1 | UnannotatedRegion |
| 644 | AGGGAACAAA.ACGCCTGATA | G | x | 35005_12_13952_XS2_1 | UnannotatedRegion |
| 644 | AGGGAACAAA.ACGCCTGATA | G | x | 35006_12_15317_XS2_1 | UnannotatedRegion |
| 644 | AGGGAACAAA.ACGCCTGATA | T | x | 35007_12_15047_XS2_1 | UnannotatedRegion |
| 644 | AGGGAACAAA.ACGCCTGATA | T | x | 35008_12_15661_XS2_1 | UnannotatedRegion |
| 644 | AGGGAACAAA.ACGCCTGATA | T | x | 35009_12_15657_XS2_1 | UnannotatedRegion |
| 644 | AGGGAACAAA.ACGCCTGATA | G | x | 35010_12_17186_XS2_1 | UnannotatedRegion |
| 644 | AGGGAACAAA.ACGCCTGATA | G | x | 35011_12_17194_XS2_1 | UnannotatedRegion |
| 644 | AGGGAACAAA.ACGCCTGATA | G | x | 35012_12_17009_XS2_1 | UnannotatedRegion |
| 644 | AGGGAACAAA.ACGCCTGATA | T | x | 35013_12_14973_XS2_1 | UnannotatedRegion |
|  |  |  |  |  |  |
| 652 | AGGTATAATC.CCCGCATTGC | A | 1186388 F | 19260_WUE_2594 | UnannotatedRegion |
| 652 | AGGTATAATC.CCCGCATTGC | G | x | 34990_Tchad54_11 | UnannotatedRegion |
| 652 | AGGTATAATC.CCCGCATTGC | G | x | 34991_Tchad78_11 | UnannotatedRegion |
| 652 | AGGTATAATC.CCCGCATTGC | G | x | 34992_Tchad95_11 | UnannotatedRegion |
| 652 | AGGTATAATC.CCCGCATTGC | G | x | 34994_Tchad106_11 | UnannotatedRegion |
| 652 | AGGTATAATC.CCCGCATTGC | G | x | 34995_8_2011 | UnannotatedRegion |
| 652 | AGGTATAATC.CCCGCATTGC | G | x | 34996_10_2011 | UnannotatedRegion |
| 652 | AGGTATAATC.CCCGCATTGC | G | x | 34997_39_2011 | UnannotatedRegion |
| 652 | AGGTATAATC.CCCGCATTGC | G | x | 34998_59_2011 | UnannotatedRegion |
| 652 | AGGTATAATC.CCCGCATTGC | G | x | 34999_63_2011 | UnannotatedRegion |
| 652 | AGGTATAATC.CCCGCATTGC | G | x | 35000_75_2011 | UnannotatedRegion |
| 652 | AGGTATAATC.CCCGCATTGC | G | x | 35001_76_2011 | UnannotatedRegion |
| 652 | AGGTATAATC.CCCGCATTGC | G | x | 35002_120_2011 | UnannotatedRegion |
| 652 | AGGTATAATC.CCCGCATTGC | G | x | 35003_403_2011 | UnannotatedRegion |
| 652 | AGGTATAATC.CCCGCATTGC | G | x | 35004_12_15398_XS2_1 | UnannotatedRegion |
| 652 | AGGTATAATC.CCCGCATTGC | G | x | 35005_12_13952_XS2_1 | UnannotatedRegion |
| 652 | AGGTATAATC.CCCGCATTGC | G | x | 35006_12_15317_XS2_1 | UnannotatedRegion |
| 652 | AGGTATAATC.CCCGCATTGC | G | x | 35007_12_15047_XS2_1 | UnannotatedRegion |
| 652 | AGGTATAATC.CCCGCATTGC | G | x | 35008_12_15661_XS2_1 | UnannotatedRegion |
| 652 | AGGTATAATC.CCCGCATTGC | G | x | 35009_12_15657_XS2_1 | UnannotatedRegion |
| 652 | AGGTATAATC.CCCGCATTGC | G | x | 35010_12_17186_XS2_1 | UnannotatedRegion |
| 652 | AGGTATAATC.CCCGCATTGC | G | x | 35011_12_17194_XS2_1 | UnannotatedRegion |
| 652 | AGGTATAATC.CCCGCATTGC | G | x | 35012_12_17009_XS2_1 | UnannotatedRegion |
| 652 | AGGTATAATC.CCCGCATTGC | G | x | 35013_12_14973_XS2_1 | UnannotatedRegion |
|  |  |  |  |  |  |
| 662 | AGTACAAAAC.TTAAAAAATA | C | 1303786 F | 19260_WUE_2594 | UnannotatedRegion |
| 662 | AGTACAAAAC.TTAAAAAATA | A | x | 34990_Tchad54_11 | UnannotatedRegion |
| 662 | AGTACAAAAC.TTAAAAAATA | A | x | 34991_Tchad78_11 | UnannotatedRegion |
| 662 | AGTACAAAAC.TTAAAAAATA | A | x | 34992_Tchad95_11 | UnannotatedRegion |
| 662 | AGTACAAAAC.TTAAAAAATA | A | x | 34994_Tchad106_11 | UnannotatedRegion |
| 662 | AGTACAAAAC.TTAAAAAATA | A | x | 34995_8_2011 | UnannotatedRegion |
| 662 | AGTACAAAAC.TTAAAAAATA | A | x | 34996_10_2011 | UnannotatedRegion |
| 662 | AGTACAAAAC.TTAAAAAATA | A | x | 34997_39_2011 | UnannotatedRegion |
| 662 | AGTACAAAAC.TTAAAAAATA | A | x | 34998_59_2011 | UnannotatedRegion |
| 662 | AGTACAAAAC.TTAAAAAATA | A | x | 34999_63_2011 | UnannotatedRegion |
| 662 | AGTACAAAAC.TTAAAAAATA | A | x | 35000_75_2011 | UnannotatedRegion |
| 662 | AGTACAAAAC.TTAAAAAATA | A | x | 35001_76_2011 | UnannotatedRegion |
| 662 | AGTACAAAAC.TTAAAAAATA | A | x | 35002_120_2011 | UnannotatedRegion |
| 662 | AGTACAAAAC.TTAAAAAATA | A | x | 35003_403_2011 | UnannotatedRegion |
| 662 | AGTACAAAAC.TTAAAAAATA | A | x | 35004_12_15398_XS2_1 | UnannotatedRegion |
| 662 | AGTACAAAAC.TTAAAAAATA | A | x | 35005_12_13952_XS2_1 | UnannotatedRegion |
| 662 | AGTACAAAAC.TTAAAAAATA | A | x | 35006_12_15317_XS2_1 | UnannotatedRegion |
| 662 | AGTACAAAAC.TTAAAAAATA | A | x | 35007_12_15047_XS2_1 | UnannotatedRegion |
| 662 | AGTACAAAAC.TTAAAAAATA | A | x | 35008_12_15661_XS2_1 | UnannotatedRegion |
| 662 | AGTACAAAAC.TTAAAAAATA | A | x | 35009_12_15657_XS2_1 | UnannotatedRegion |
| 662 | AGTACAAAAC.TTAAAAAATA | A | x | 35010_12_17186_XS2_1 | UnannotatedRegion |
| 662 | AGTACAAAAC.TTAAAAAATA | A | x | 35011_12_17194_XS2_1 | UnannotatedRegion |
| 662 | AGTACAAAAC.TTAAAAAATA | A | x | 35012_12_17009_XS2_1 | UnannotatedRegion |
| 662 | AGTACAAAAC.TTAAAAAATA | A | x | 35013_12_14973_XS2_1 | UnannotatedRegion |
|  |  |  |  |  |  |
| 672 | AGTCTGTCTT.GAGATAAACC | C | 265479 F | 19260_WUE_2594 | UnannotatedRegion |
| 672 | AGTCTGTCTT.GAGATAAACC | T | x | 34990_Tchad54_11 | UnannotatedRegion |
| 672 | AGTCTGTCTT.GAGATAAACC | T | x | 34991_Tchad78_11 | UnannotatedRegion |
| 672 | AGTCTGTCTT.GAGATAAACC | T | x | 34992_Tchad95_11 | UnannotatedRegion |
| 672 | AGTCTGTCTT.GAGATAAACC | T | x | 34994_Tchad106_11 | UnannotatedRegion |
| 672 | AGTCTGTCTT.GAGATAAACC | T | x | 34995_8_2011 | UnannotatedRegion |
| 672 | AGTCTGTCTT.GAGATAAACC | T | x | 34996_10_2011 | UnannotatedRegion |
| 672 | AGTCTGTCTT.GAGATAAACC | T | x | 34997_39_2011 | UnannotatedRegion |
| 672 | AGTCTGTCTT.GAGATAAACC | T | x | 34998_59_2011 | UnannotatedRegion |
| 672 | AGTCTGTCTT.GAGATAAACC | T | x | 34999_63_2011 | UnannotatedRegion |
| 672 | AGTCTGTCTT.GAGATAAACC | T | x | 35000_75_2011 | UnannotatedRegion |
| 672 | AGTCTGTCTT.GAGATAAACC | T | x | 35001_76_2011 | UnannotatedRegion |
| 672 | AGTCTGTCTT.GAGATAAACC | T | x | 35002_120_2011 | UnannotatedRegion |
| 672 | AGTCTGTCTT.GAGATAAACC | T | x | 35003_403_2011 | UnannotatedRegion |
| 672 | AGTCTGTCTT.GAGATAAACC | T | x | 35004_12_15398_XS2_1 | UnannotatedRegion |
| 672 | AGTCTGTCTT.GAGATAAACC | T | x | 35005_12_13952_XS2_1 | UnannotatedRegion |
| 672 | AGTCTGTCTT.GAGATAAACC | T | x | 35006_12_15317_XS2_1 | UnannotatedRegion |
| 672 | AGTCTGTCTT.GAGATAAACC | T | x | 35007_12_15047_XS2_1 | UnannotatedRegion |
| 672 | AGTCTGTCTT.GAGATAAACC | T | x | 35008_12_15661_XS2_1 | UnannotatedRegion |
| 672 | AGTCTGTCTT.GAGATAAACC | T | x | 35009_12_15657_XS2_1 | UnannotatedRegion |
| 672 | AGTCTGTCTT.GAGATAAACC | T | x | 35010_12_17186_XS2_1 | UnannotatedRegion |
| 672 | AGTCTGTCTT.GAGATAAACC | T | x | 35011_12_17194_XS2_1 | UnannotatedRegion |
| 672 | AGTCTGTCTT.GAGATAAACC | T | x | 35012_12_17009_XS2_1 | UnannotatedRegion |
| 672 | AGTCTGTCTT.GAGATAAACC | T | x | 35013_12_14973_XS2_1 | UnannotatedRegion |
|  |  |  |  |  |  |
| 686 | ATAAAAGGAA.TATAACTTTA | C | 1694283 F | 19260_WUE_2594 | UnannotatedRegion |
| 686 | ATAAAAGGAA.TATAACTTTA | C | x | 34990_Tchad54_11 | UnannotatedRegion |
| 686 | ATAAAAGGAA.TATAACTTTA | C | x | 34991_Tchad78_11 | UnannotatedRegion |
| 686 | ATAAAAGGAA.TATAACTTTA | T | x | 34992_Tchad95_11 | UnannotatedRegion |
| 686 | ATAAAAGGAA.TATAACTTTA | T | x | 34994_Tchad106_11 | UnannotatedRegion |
| 686 | ATAAAAGGAA.TATAACTTTA | C | x | 34995_8_2011 | UnannotatedRegion |
| 686 | ATAAAAGGAA.TATAACTTTA | C | x | 34996_10_2011 | UnannotatedRegion |
| 686 | ATAAAAGGAA.TATAACTTTA | C | x | 34997_39_2011 | UnannotatedRegion |
| 686 | ATAAAAGGAA.TATAACTTTA | C | x | 34998_59_2011 | UnannotatedRegion |
| 686 | ATAAAAGGAA.TATAACTTTA | C | x | 34999_63_2011 | UnannotatedRegion |
| 686 | ATAAAAGGAA.TATAACTTTA | C | x | 35000_75_2011 | UnannotatedRegion |
| 686 | ATAAAAGGAA.TATAACTTTA | C | x | 35001_76_2011 | UnannotatedRegion |
| 686 | ATAAAAGGAA.TATAACTTTA | C | x | 35002_120_2011 | UnannotatedRegion |
| 686 | ATAAAAGGAA.TATAACTTTA | C | x | 35003_403_2011 | UnannotatedRegion |
| 686 | ATAAAAGGAA.TATAACTTTA | C | x | 35004_12_15398_XS2_1 | UnannotatedRegion |
| 686 | ATAAAAGGAA.TATAACTTTA | C | x | 35005_12_13952_XS2_1 | UnannotatedRegion |
| 686 | ATAAAAGGAA.TATAACTTTA | C | x | 35006_12_15317_XS2_1 | UnannotatedRegion |
| 686 | ATAAAAGGAA.TATAACTTTA | T | x | 35007_12_15047_XS2_1 | UnannotatedRegion |
| 686 | ATAAAAGGAA.TATAACTTTA | T | x | 35008_12_15661_XS2_1 | UnannotatedRegion |
| 686 | ATAAAAGGAA.TATAACTTTA | T | x | 35009_12_15657_XS2_1 | UnannotatedRegion |
| 686 | ATAAAAGGAA.TATAACTTTA | C | x | 35010_12_17186_XS2_1 | UnannotatedRegion |
| 686 | ATAAAAGGAA.TATAACTTTA | C | x | 35011_12_17194_XS2_1 | UnannotatedRegion |
| 686 | ATAAAAGGAA.TATAACTTTA | C | x | 35012_12_17009_XS2_1 | UnannotatedRegion |
| 686 | ATAAAAGGAA.TATAACTTTA | T | x | 35013_12_14973_XS2_1 | UnannotatedRegion |
|  |  |  |  |  |  |
| 687 | ATAAACAACA.ACAGATGCCG | G | 110903 F | 19260_WUE_2594 | UnannotatedRegion |
| 687 | ATAAACAACA.ACAGATGCCG | G | x | 34990_Tchad54_11 | UnannotatedRegion |
| 687 | ATAAACAACA.ACAGATGCCG | G | x | 34991_Tchad78_11 | UnannotatedRegion |
| 687 | ATAAACAACA.ACAGATGCCG | G | x | 34992_Tchad95_11 | UnannotatedRegion |
| 687 | ATAAACAACA.ACAGATGCCG | G | x | 34994_Tchad106_11 | UnannotatedRegion |
| 687 | ATAAACAACA.ACAGATGCCG | A | x | 34995_8_2011 | UnannotatedRegion |
| 687 | ATAAACAACA.ACAGATGCCG | A | x | 34996_10_2011 | UnannotatedRegion |
| 687 | ATAAACAACA.ACAGATGCCG | G | x | 34997_39_2011 | UnannotatedRegion |
| 687 | ATAAACAACA.ACAGATGCCG | G | x | 34998_59_2011 | UnannotatedRegion |
| 687 | ATAAACAACA.ACAGATGCCG | G | x | 34999_63_2011 | UnannotatedRegion |
| 687 | ATAAACAACA.ACAGATGCCG | G | x | 35000_75_2011 | UnannotatedRegion |
| 687 | ATAAACAACA.ACAGATGCCG | G | x | 35001_76_2011 | UnannotatedRegion |
| 687 | ATAAACAACA.ACAGATGCCG | G | x | 35002_120_2011 | UnannotatedRegion |
| 687 | ATAAACAACA.ACAGATGCCG | G | x | 35003_403_2011 | UnannotatedRegion |
| 687 | ATAAACAACA.ACAGATGCCG | G | x | 35004_12_15398_XS2_1 | UnannotatedRegion |
| 687 | ATAAACAACA.ACAGATGCCG | G | x | 35005_12_13952_XS2_1 | UnannotatedRegion |
| 687 | ATAAACAACA.ACAGATGCCG | G | x | 35006_12_15317_XS2_1 | UnannotatedRegion |
| 687 | ATAAACAACA.ACAGATGCCG | G | x | 35007_12_15047_XS2_1 | UnannotatedRegion |
| 687 | ATAAACAACA.ACAGATGCCG | G | x | 35008_12_15661_XS2_1 | UnannotatedRegion |
| 687 | ATAAACAACA.ACAGATGCCG | G | x | 35009_12_15657_XS2_1 | UnannotatedRegion |
| 687 | ATAAACAACA.ACAGATGCCG | G | x | 35010_12_17186_XS2_1 | UnannotatedRegion |
| 687 | ATAAACAACA.ACAGATGCCG | G | x | 35011_12_17194_XS2_1 | UnannotatedRegion |
| 687 | ATAAACAACA.ACAGATGCCG | G | x | 35012_12_17009_XS2_1 | UnannotatedRegion |
| 687 | ATAAACAACA.ACAGATGCCG | G | x | 35013_12_14973_XS2_1 | UnannotatedRegion |
|  |  |  |  |  |  |
| 695 | ATAAGCACGG.TGCCGAACAA | T | 1731068 R | 19260_WUE_2594 | UnannotatedRegion |
| 695 | ATAAGCACGG.TGCCGAACAA | T | x | 34990_Tchad54_11 | UnannotatedRegion |
| 695 | ATAAGCACGG.TGCCGAACAA | T | x | 34991_Tchad78_11 | UnannotatedRegion |
| 695 | ATAAGCACGG.TGCCGAACAA | T | x | 34992_Tchad95_11 | UnannotatedRegion |
| 695 | ATAAGCACGG.TGCCGAACAA | T | x | 34994_Tchad106_11 | UnannotatedRegion |
| 695 | ATAAGCACGG.TGCCGAACAA | T | x | 34995_8_2011 | UnannotatedRegion |
| 695 | ATAAGCACGG.TGCCGAACAA | T | x | 34996_10_2011 | UnannotatedRegion |
| 695 | ATAAGCACGG.TGCCGAACAA | T | x | 34997_39_2011 | UnannotatedRegion |
| 695 | ATAAGCACGG.TGCCGAACAA | T | x | 34998_59_2011 | UnannotatedRegion |
| 695 | ATAAGCACGG.TGCCGAACAA | T | x | 34999_63_2011 | UnannotatedRegion |
| 695 | ATAAGCACGG.TGCCGAACAA | T | x | 35000_75_2011 | UnannotatedRegion |
| 695 | ATAAGCACGG.TGCCGAACAA | T | x | 35001_76_2011 | UnannotatedRegion |
| 695 | ATAAGCACGG.TGCCGAACAA | T | x | 35002_120_2011 | UnannotatedRegion |
| 695 | ATAAGCACGG.TGCCGAACAA | T | x | 35003_403_2011 | UnannotatedRegion |
| 695 | ATAAGCACGG.TGCCGAACAA | T | x | 35004_12_15398_XS2_1 | UnannotatedRegion |
| 695 | ATAAGCACGG.TGCCGAACAA | T | x | 35005_12_13952_XS2_1 | UnannotatedRegion |
| 695 | ATAAGCACGG.TGCCGAACAA | T | x | 35006_12_15317_XS2_1 | UnannotatedRegion |
| 695 | ATAAGCACGG.TGCCGAACAA | C | x | 35007_12_15047_XS2_1 | UnannotatedRegion |
| 695 | ATAAGCACGG.TGCCGAACAA | T | x | 35008_12_15661_XS2_1 | UnannotatedRegion |
| 695 | ATAAGCACGG.TGCCGAACAA | T | x | 35009_12_15657_XS2_1 | UnannotatedRegion |
| 695 | ATAAGCACGG.TGCCGAACAA | T | x | 35010_12_17186_XS2_1 | UnannotatedRegion |
| 695 | ATAAGCACGG.TGCCGAACAA | T | x | 35011_12_17194_XS2_1 | UnannotatedRegion |
| 695 | ATAAGCACGG.TGCCGAACAA | T | x | 35012_12_17009_XS2_1 | UnannotatedRegion |
| 695 | ATAAGCACGG.TGCCGAACAA | T | x | 35013_12_14973_XS2_1 | UnannotatedRegion |
|  |  |  |  |  |  |
| 696 | ATAAGCGGGG.GGGTGTCCGA | C | 1270203 R | 19260_WUE_2594 | UnannotatedRegion |
| 696 | ATAAGCGGGG.GGGTGTCCGA | A | x | 34990_Tchad54_11 | UnannotatedRegion |
| 696 | ATAAGCGGGG.GGGTGTCCGA | A | x | 34991_Tchad78_11 | UnannotatedRegion |
| 696 | ATAAGCGGGG.GGGTGTCCGA | A | x | 34992_Tchad95_11 | UnannotatedRegion |
| 696 | ATAAGCGGGG.GGGTGTCCGA | A | x | 34994_Tchad106_11 | UnannotatedRegion |
| 696 | ATAAGCGGGG.GGGTGTCCGA | A | x | 34995_8_2011 | UnannotatedRegion |
| 696 | ATAAGCGGGG.GGGTGTCCGA | A | x | 34996_10_2011 | UnannotatedRegion |
| 696 | ATAAGCGGGG.GGGTGTCCGA | A | x | 34997_39_2011 | UnannotatedRegion |
| 696 | ATAAGCGGGG.GGGTGTCCGA | A | x | 34998_59_2011 | UnannotatedRegion |
| 696 | ATAAGCGGGG.GGGTGTCCGA | A | x | 34999_63_2011 | UnannotatedRegion |
| 696 | ATAAGCGGGG.GGGTGTCCGA | A | x | 35000_75_2011 | UnannotatedRegion |
| 696 | ATAAGCGGGG.GGGTGTCCGA | A | x | 35001_76_2011 | UnannotatedRegion |
| 696 | ATAAGCGGGG.GGGTGTCCGA | A | x | 35002_120_2011 | UnannotatedRegion |
| 696 | ATAAGCGGGG.GGGTGTCCGA | A | x | 35003_403_2011 | UnannotatedRegion |
| 696 | ATAAGCGGGG.GGGTGTCCGA | A | x | 35004_12_15398_XS2_1 | UnannotatedRegion |
| 696 | ATAAGCGGGG.GGGTGTCCGA | A | x | 35005_12_13952_XS2_1 | UnannotatedRegion |
| 696 | ATAAGCGGGG.GGGTGTCCGA | A | x | 35006_12_15317_XS2_1 | UnannotatedRegion |
| 696 | ATAAGCGGGG.GGGTGTCCGA | A | x | 35007_12_15047_XS2_1 | UnannotatedRegion |
| 696 | ATAAGCGGGG.GGGTGTCCGA | A | x | 35008_12_15661_XS2_1 | UnannotatedRegion |
| 696 | ATAAGCGGGG.GGGTGTCCGA | A | x | 35009_12_15657_XS2_1 | UnannotatedRegion |
| 696 | ATAAGCGGGG.GGGTGTCCGA | A | x | 35010_12_17186_XS2_1 | UnannotatedRegion |
| 696 | ATAAGCGGGG.GGGTGTCCGA | A | x | 35011_12_17194_XS2_1 | UnannotatedRegion |
| 696 | ATAAGCGGGG.GGGTGTCCGA | A | x | 35012_12_17009_XS2_1 | UnannotatedRegion |
| 696 | ATAAGCGGGG.GGGTGTCCGA | A | x | 35013_12_14973_XS2_1 | UnannotatedRegion |
|  |  |  |  |  |  |
| 708 | ATAGTTTTAA.TAATTTATAT | A | 860835 F | 19260_WUE_2594 | UnannotatedRegion |
| 708 | ATAGTTTTAA.TAATTTATAT | G | x | 34990_Tchad54_11 | UnannotatedRegion |
| 708 | ATAGTTTTAA.TAATTTATAT | G | x | 34991_Tchad78_11 | UnannotatedRegion |
| 708 | ATAGTTTTAA.TAATTTATAT | G | x | 34992_Tchad95_11 | UnannotatedRegion |
| 708 | ATAGTTTTAA.TAATTTATAT | G | x | 34994_Tchad106_11 | UnannotatedRegion |
| 708 | ATAGTTTTAA.TAATTTATAT | G | x | 34995_8_2011 | UnannotatedRegion |
| 708 | ATAGTTTTAA.TAATTTATAT | G | x | 34996_10_2011 | UnannotatedRegion |
| 708 | ATAGTTTTAA.TAATTTATAT | G | x | 34997_39_2011 | UnannotatedRegion |
| 708 | ATAGTTTTAA.TAATTTATAT | G | x | 34998_59_2011 | UnannotatedRegion |
| 708 | ATAGTTTTAA.TAATTTATAT | G | x | 34999_63_2011 | UnannotatedRegion |
| 708 | ATAGTTTTAA.TAATTTATAT | G | x | 35000_75_2011 | UnannotatedRegion |
| 708 | ATAGTTTTAA.TAATTTATAT | G | x | 35001_76_2011 | UnannotatedRegion |
| 708 | ATAGTTTTAA.TAATTTATAT | G | x | 35002_120_2011 | UnannotatedRegion |
| 708 | ATAGTTTTAA.TAATTTATAT | G | x | 35003_403_2011 | UnannotatedRegion |
| 708 | ATAGTTTTAA.TAATTTATAT | G | x | 35004_12_15398_XS2_1 | UnannotatedRegion |
| 708 | ATAGTTTTAA.TAATTTATAT | G | x | 35005_12_13952_XS2_1 | UnannotatedRegion |
| 708 | ATAGTTTTAA.TAATTTATAT | G | x | 35006_12_15317_XS2_1 | UnannotatedRegion |
| 708 | ATAGTTTTAA.TAATTTATAT | G | x | 35007_12_15047_XS2_1 | UnannotatedRegion |
| 708 | ATAGTTTTAA.TAATTTATAT | G | x | 35008_12_15661_XS2_1 | UnannotatedRegion |
| 708 | ATAGTTTTAA.TAATTTATAT | G | x | 35009_12_15657_XS2_1 | UnannotatedRegion |
| 708 | ATAGTTTTAA.TAATTTATAT | G | x | 35010_12_17186_XS2_1 | UnannotatedRegion |
| 708 | ATAGTTTTAA.TAATTTATAT | G | x | 35011_12_17194_XS2_1 | UnannotatedRegion |
| 708 | ATAGTTTTAA.TAATTTATAT | G | x | 35012_12_17009_XS2_1 | UnannotatedRegion |
| 708 | ATAGTTTTAA.TAATTTATAT | G | x | 35013_12_14973_XS2_1 | UnannotatedRegion |
|  |  |  |  |  |  |
| 709 | ATATCGCCCC.GTATGTCCCG | C | 1959692 F | 19260_WUE_2594 | UnannotatedRegion |
| 709 | ATATCGCCCC.GTATGTCCCG | T | x | 34990_Tchad54_11 | UnannotatedRegion |
| 709 | ATATCGCCCC.GTATGTCCCG | T | x | 34991_Tchad78_11 | UnannotatedRegion |
| 709 | ATATCGCCCC.GTATGTCCCG | T | x | 34992_Tchad95_11 | UnannotatedRegion |
| 709 | ATATCGCCCC.GTATGTCCCG | T | x | 34994_Tchad106_11 | UnannotatedRegion |
| 709 | ATATCGCCCC.GTATGTCCCG | T | x | 34995_8_2011 | UnannotatedRegion |
| 709 | ATATCGCCCC.GTATGTCCCG | T | x | 34996_10_2011 | UnannotatedRegion |
| 709 | ATATCGCCCC.GTATGTCCCG | T | x | 34997_39_2011 | UnannotatedRegion |
| 709 | ATATCGCCCC.GTATGTCCCG | T | x | 34998_59_2011 | UnannotatedRegion |
| 709 | ATATCGCCCC.GTATGTCCCG | T | x | 34999_63_2011 | UnannotatedRegion |
| 709 | ATATCGCCCC.GTATGTCCCG | T | x | 35000_75_2011 | UnannotatedRegion |
| 709 | ATATCGCCCC.GTATGTCCCG | T | x | 35001_76_2011 | UnannotatedRegion |
| 709 | ATATCGCCCC.GTATGTCCCG | T | x | 35002_120_2011 | UnannotatedRegion |
| 709 | ATATCGCCCC.GTATGTCCCG | T | x | 35003_403_2011 | UnannotatedRegion |
| 709 | ATATCGCCCC.GTATGTCCCG | T | x | 35004_12_15398_XS2_1 | UnannotatedRegion |
| 709 | ATATCGCCCC.GTATGTCCCG | T | x | 35005_12_13952_XS2_1 | UnannotatedRegion |
| 709 | ATATCGCCCC.GTATGTCCCG | T | x | 35006_12_15317_XS2_1 | UnannotatedRegion |
| 709 | ATATCGCCCC.GTATGTCCCG | T | x | 35007_12_15047_XS2_1 | UnannotatedRegion |
| 709 | ATATCGCCCC.GTATGTCCCG | T | x | 35008_12_15661_XS2_1 | UnannotatedRegion |
| 709 | ATATCGCCCC.GTATGTCCCG | T | x | 35009_12_15657_XS2_1 | UnannotatedRegion |
| 709 | ATATCGCCCC.GTATGTCCCG | T | x | 35010_12_17186_XS2_1 | UnannotatedRegion |
| 709 | ATATCGCCCC.GTATGTCCCG | T | x | 35011_12_17194_XS2_1 | UnannotatedRegion |
| 709 | ATATCGCCCC.GTATGTCCCG | T | x | 35012_12_17009_XS2_1 | UnannotatedRegion |
| 709 | ATATCGCCCC.GTATGTCCCG | T | x | 35013_12_14973_XS2_1 | UnannotatedRegion |
|  |  |  |  |  |  |
| 719 | ATCAAATTTT.AATAGATAAA | T | 72397 R | 19260_WUE_2594 | UnannotatedRegion |
| 719 | ATCAAATTTT.AATAGATAAA | T | x | 34990_Tchad54_11 | UnannotatedRegion |
| 719 | ATCAAATTTT.AATAGATAAA | C | x | 34991_Tchad78_11 | UnannotatedRegion |
| 719 | ATCAAATTTT.AATAGATAAA | T | x | 34992_Tchad95_11 | UnannotatedRegion |
| 719 | ATCAAATTTT.AATAGATAAA | T | x | 34994_Tchad106_11 | UnannotatedRegion |
| 719 | ATCAAATTTT.AATAGATAAA | T | x | 34995_8_2011 | UnannotatedRegion |
| 719 | ATCAAATTTT.AATAGATAAA | T | x | 34996_10_2011 | UnannotatedRegion |
| 719 | ATCAAATTTT.AATAGATAAA | T | x | 34997_39_2011 | UnannotatedRegion |
| 719 | ATCAAATTTT.AATAGATAAA | T | x | 34998_59_2011 | UnannotatedRegion |
| 719 | ATCAAATTTT.AATAGATAAA | T | x | 34999_63_2011 | UnannotatedRegion |
| 719 | ATCAAATTTT.AATAGATAAA | T | x | 35000_75_2011 | UnannotatedRegion |
| 719 | ATCAAATTTT.AATAGATAAA | T | x | 35001_76_2011 | UnannotatedRegion |
| 719 | ATCAAATTTT.AATAGATAAA | T | x | 35002_120_2011 | UnannotatedRegion |
| 719 | ATCAAATTTT.AATAGATAAA | C | x | 35003_403_2011 | UnannotatedRegion |
| 719 | ATCAAATTTT.AATAGATAAA | T | x | 35004_12_15398_XS2_1 | UnannotatedRegion |
| 719 | ATCAAATTTT.AATAGATAAA | T | x | 35005_12_13952_XS2_1 | UnannotatedRegion |
| 719 | ATCAAATTTT.AATAGATAAA | T | x | 35006_12_15317_XS2_1 | UnannotatedRegion |
| 719 | ATCAAATTTT.AATAGATAAA | T | x | 35007_12_15047_XS2_1 | UnannotatedRegion |
| 719 | ATCAAATTTT.AATAGATAAA | T | x | 35008_12_15661_XS2_1 | UnannotatedRegion |
| 719 | ATCAAATTTT.AATAGATAAA | T | x | 35009_12_15657_XS2_1 | UnannotatedRegion |
| 719 | ATCAAATTTT.AATAGATAAA | T | x | 35010_12_17186_XS2_1 | UnannotatedRegion |
| 719 | ATCAAATTTT.AATAGATAAA | T | x | 35011_12_17194_XS2_1 | UnannotatedRegion |
| 719 | ATCAAATTTT.AATAGATAAA | T | x | 35012_12_17009_XS2_1 | UnannotatedRegion |
| 719 | ATCAAATTTT.AATAGATAAA | T | x | 35013_12_14973_XS2_1 | UnannotatedRegion |
|  |  |  |  |  |  |
| 729 | ATCCGATAAA.ACGGATACAA | T | 255448 F | 19260_WUE_2594 | UnannotatedRegion |
| 729 | ATCCGATAAA.ACGGATACAA | C | x | 34990_Tchad54_11 | UnannotatedRegion |
| 729 | ATCCGATAAA.ACGGATACAA | C | x | 34991_Tchad78_11 | UnannotatedRegion |
| 729 | ATCCGATAAA.ACGGATACAA | C | x | 34992_Tchad95_11 | UnannotatedRegion |
| 729 | ATCCGATAAA.ACGGATACAA | C | x | 34994_Tchad106_11 | UnannotatedRegion |
| 729 | ATCCGATAAA.ACGGATACAA | C | x | 34995_8_2011 | UnannotatedRegion |
| 729 | ATCCGATAAA.ACGGATACAA | C | x | 34996_10_2011 | UnannotatedRegion |
| 729 | ATCCGATAAA.ACGGATACAA | C | x | 34997_39_2011 | UnannotatedRegion |
| 729 | ATCCGATAAA.ACGGATACAA | C | x | 34998_59_2011 | UnannotatedRegion |
| 729 | ATCCGATAAA.ACGGATACAA | C | x | 34999_63_2011 | UnannotatedRegion |
| 729 | ATCCGATAAA.ACGGATACAA | C | x | 35000_75_2011 | UnannotatedRegion |
| 729 | ATCCGATAAA.ACGGATACAA | C | x | 35001_76_2011 | UnannotatedRegion |
| 729 | ATCCGATAAA.ACGGATACAA | C | x | 35002_120_2011 | UnannotatedRegion |
| 729 | ATCCGATAAA.ACGGATACAA | C | x | 35003_403_2011 | UnannotatedRegion |
| 729 | ATCCGATAAA.ACGGATACAA | C | x | 35004_12_15398_XS2_1 | UnannotatedRegion |
| 729 | ATCCGATAAA.ACGGATACAA | C | x | 35005_12_13952_XS2_1 | UnannotatedRegion |
| 729 | ATCCGATAAA.ACGGATACAA | C | x | 35006_12_15317_XS2_1 | UnannotatedRegion |
| 729 | ATCCGATAAA.ACGGATACAA | C | x | 35007_12_15047_XS2_1 | UnannotatedRegion |
| 729 | ATCCGATAAA.ACGGATACAA | C | x | 35008_12_15661_XS2_1 | UnannotatedRegion |
| 729 | ATCCGATAAA.ACGGATACAA | C | x | 35009_12_15657_XS2_1 | UnannotatedRegion |
| 729 | ATCCGATAAA.ACGGATACAA | C | x | 35010_12_17186_XS2_1 | UnannotatedRegion |
| 729 | ATCCGATAAA.ACGGATACAA | C | x | 35011_12_17194_XS2_1 | UnannotatedRegion |
| 729 | ATCCGATAAA.ACGGATACAA | C | x | 35012_12_17009_XS2_1 | UnannotatedRegion |
| 729 | ATCCGATAAA.ACGGATACAA | C | x | 35013_12_14973_XS2_1 | UnannotatedRegion |
|  |  |  |  |  |  |
| 740 | ATCGGTTTTT.CGAATCTGCG | G | 741993 R | 19260_WUE_2594 | UnannotatedRegion |
| 740 | ATCGGTTTTT.CGAATCTGCG | A | x | 34990_Tchad54_11 | UnannotatedRegion |
| 740 | ATCGGTTTTT.CGAATCTGCG | A | x | 34991_Tchad78_11 | UnannotatedRegion |
| 740 | ATCGGTTTTT.CGAATCTGCG | A | x | 34992_Tchad95_11 | UnannotatedRegion |
| 740 | ATCGGTTTTT.CGAATCTGCG | A | x | 34994_Tchad106_11 | UnannotatedRegion |
| 740 | ATCGGTTTTT.CGAATCTGCG | A | x | 34995_8_2011 | UnannotatedRegion |
| 740 | ATCGGTTTTT.CGAATCTGCG | A | x | 34996_10_2011 | UnannotatedRegion |
| 740 | ATCGGTTTTT.CGAATCTGCG | A | x | 34997_39_2011 | UnannotatedRegion |
| 740 | ATCGGTTTTT.CGAATCTGCG | A | x | 34998_59_2011 | UnannotatedRegion |
| 740 | ATCGGTTTTT.CGAATCTGCG | A | x | 34999_63_2011 | UnannotatedRegion |
| 740 | ATCGGTTTTT.CGAATCTGCG | A | x | 35000_75_2011 | UnannotatedRegion |
| 740 | ATCGGTTTTT.CGAATCTGCG | A | x | 35001_76_2011 | UnannotatedRegion |
| 740 | ATCGGTTTTT.CGAATCTGCG | A | x | 35002_120_2011 | UnannotatedRegion |
| 740 | ATCGGTTTTT.CGAATCTGCG | A | x | 35003_403_2011 | UnannotatedRegion |
| 740 | ATCGGTTTTT.CGAATCTGCG | A | x | 35004_12_15398_XS2_1 | UnannotatedRegion |
| 740 | ATCGGTTTTT.CGAATCTGCG | A | x | 35005_12_13952_XS2_1 | UnannotatedRegion |
| 740 | ATCGGTTTTT.CGAATCTGCG | A | x | 35006_12_15317_XS2_1 | UnannotatedRegion |
| 740 | ATCGGTTTTT.CGAATCTGCG | A | x | 35007_12_15047_XS2_1 | UnannotatedRegion |
| 740 | ATCGGTTTTT.CGAATCTGCG | A | x | 35008_12_15661_XS2_1 | UnannotatedRegion |
| 740 | ATCGGTTTTT.CGAATCTGCG | A | x | 35009_12_15657_XS2_1 | UnannotatedRegion |
| 740 | ATCGGTTTTT.CGAATCTGCG | A | x | 35010_12_17186_XS2_1 | UnannotatedRegion |
| 740 | ATCGGTTTTT.CGAATCTGCG | A | x | 35011_12_17194_XS2_1 | UnannotatedRegion |
| 740 | ATCGGTTTTT.CGAATCTGCG | A | x | 35012_12_17009_XS2_1 | UnannotatedRegion |
| 740 | ATCGGTTTTT.CGAATCTGCG | A | x | 35013_12_14973_XS2_1 | UnannotatedRegion |
|  |  |  |  |  |  |
| 741 | ATCGTCGCTG.GGCTTTGGGG | T | 1253301 R | 19260_WUE_2594 | UnannotatedRegion |
| 741 | ATCGTCGCTG.GGCTTTGGGG | C | x | 34990_Tchad54_11 | UnannotatedRegion |
| 741 | ATCGTCGCTG.GGCTTTGGGG | C | x | 34991_Tchad78_11 | UnannotatedRegion |
| 741 | ATCGTCGCTG.GGCTTTGGGG | C | x | 34992_Tchad95_11 | UnannotatedRegion |
| 741 | ATCGTCGCTG.GGCTTTGGGG | C | x | 34994_Tchad106_11 | UnannotatedRegion |
| 741 | ATCGTCGCTG.GGCTTTGGGG | C | x | 34995_8_2011 | UnannotatedRegion |
| 741 | ATCGTCGCTG.GGCTTTGGGG | C | x | 34996_10_2011 | UnannotatedRegion |
| 741 | ATCGTCGCTG.GGCTTTGGGG | C | x | 34997_39_2011 | UnannotatedRegion |
| 741 | ATCGTCGCTG.GGCTTTGGGG | C | x | 34998_59_2011 | UnannotatedRegion |
| 741 | ATCGTCGCTG.GGCTTTGGGG | C | x | 34999_63_2011 | UnannotatedRegion |
| 741 | ATCGTCGCTG.GGCTTTGGGG | C | x | 35000_75_2011 | UnannotatedRegion |
| 741 | ATCGTCGCTG.GGCTTTGGGG | C | x | 35001_76_2011 | UnannotatedRegion |
| 741 | ATCGTCGCTG.GGCTTTGGGG | C | x | 35002_120_2011 | UnannotatedRegion |
| 741 | ATCGTCGCTG.GGCTTTGGGG | C | x | 35003_403_2011 | UnannotatedRegion |
| 741 | ATCGTCGCTG.GGCTTTGGGG | C | x | 35004_12_15398_XS2_1 | UnannotatedRegion |
| 741 | ATCGTCGCTG.GGCTTTGGGG | C | x | 35005_12_13952_XS2_1 | UnannotatedRegion |
| 741 | ATCGTCGCTG.GGCTTTGGGG | C | x | 35006_12_15317_XS2_1 | UnannotatedRegion |
| 741 | ATCGTCGCTG.GGCTTTGGGG | C | x | 35007_12_15047_XS2_1 | UnannotatedRegion |
| 741 | ATCGTCGCTG.GGCTTTGGGG | C | x | 35008_12_15661_XS2_1 | UnannotatedRegion |
| 741 | ATCGTCGCTG.GGCTTTGGGG | C | x | 35009_12_15657_XS2_1 | UnannotatedRegion |
| 741 | ATCGTCGCTG.GGCTTTGGGG | C | x | 35010_12_17186_XS2_1 | UnannotatedRegion |
| 741 | ATCGTCGCTG.GGCTTTGGGG | C | x | 35011_12_17194_XS2_1 | UnannotatedRegion |
| 741 | ATCGTCGCTG.GGCTTTGGGG | C | x | 35012_12_17009_XS2_1 | UnannotatedRegion |
| 741 | ATCGTCGCTG.GGCTTTGGGG | C | x | 35013_12_14973_XS2_1 | UnannotatedRegion |
|  |  |  |  |  |  |
| 750 | ATGAAAAGAA.ACCCTATTCC | G | 230324 R | 19260_WUE_2594 | UnannotatedRegion |
| 750 | ATGAAAAGAA.ACCCTATTCC | A | x | 34990_Tchad54_11 | UnannotatedRegion |
| 750 | ATGAAAAGAA.ACCCTATTCC | A | x | 34991_Tchad78_11 | UnannotatedRegion |
| 750 | ATGAAAAGAA.ACCCTATTCC | A | x | 34992_Tchad95_11 | UnannotatedRegion |
| 750 | ATGAAAAGAA.ACCCTATTCC | A | x | 34994_Tchad106_11 | UnannotatedRegion |
| 750 | ATGAAAAGAA.ACCCTATTCC | A | x | 34995_8_2011 | UnannotatedRegion |
| 750 | ATGAAAAGAA.ACCCTATTCC | A | x | 34996_10_2011 | UnannotatedRegion |
| 750 | ATGAAAAGAA.ACCCTATTCC | A | x | 34997_39_2011 | UnannotatedRegion |
| 750 | ATGAAAAGAA.ACCCTATTCC | A | x | 34998_59_2011 | UnannotatedRegion |
| 750 | ATGAAAAGAA.ACCCTATTCC | A | x | 34999_63_2011 | UnannotatedRegion |
| 750 | ATGAAAAGAA.ACCCTATTCC | A | x | 35000_75_2011 | UnannotatedRegion |
| 750 | ATGAAAAGAA.ACCCTATTCC | A | x | 35001_76_2011 | UnannotatedRegion |
| 750 | ATGAAAAGAA.ACCCTATTCC | A | x | 35002_120_2011 | UnannotatedRegion |
| 750 | ATGAAAAGAA.ACCCTATTCC | A | x | 35003_403_2011 | UnannotatedRegion |
| 750 | ATGAAAAGAA.ACCCTATTCC | A | x | 35004_12_15398_XS2_1 | UnannotatedRegion |
| 750 | ATGAAAAGAA.ACCCTATTCC | A | x | 35005_12_13952_XS2_1 | UnannotatedRegion |
| 750 | ATGAAAAGAA.ACCCTATTCC | A | x | 35006_12_15317_XS2_1 | UnannotatedRegion |
| 750 | ATGAAAAGAA.ACCCTATTCC | A | x | 35007_12_15047_XS2_1 | UnannotatedRegion |
| 750 | ATGAAAAGAA.ACCCTATTCC | A | x | 35008_12_15661_XS2_1 | UnannotatedRegion |
| 750 | ATGAAAAGAA.ACCCTATTCC | A | x | 35009_12_15657_XS2_1 | UnannotatedRegion |
| 750 | ATGAAAAGAA.ACCCTATTCC | A | x | 35010_12_17186_XS2_1 | UnannotatedRegion |
| 750 | ATGAAAAGAA.ACCCTATTCC | A | x | 35011_12_17194_XS2_1 | UnannotatedRegion |
| 750 | ATGAAAAGAA.ACCCTATTCC | A | x | 35012_12_17009_XS2_1 | UnannotatedRegion |
| 750 | ATGAAAAGAA.ACCCTATTCC | A | x | 35013_12_14973_XS2_1 | UnannotatedRegion |
|  |  |  |  |  |  |
| 751 | ATGAAAATCC.TTTTCACAAT | A | 229113 R | 19260_WUE_2594 | UnannotatedRegion |
| 751 | ATGAAAATCC.TTTTCACAAT | G | x | 34990_Tchad54_11 | UnannotatedRegion |
| 751 | ATGAAAATCC.TTTTCACAAT | G | x | 34991_Tchad78_11 | UnannotatedRegion |
| 751 | ATGAAAATCC.TTTTCACAAT | G | x | 34992_Tchad95_11 | UnannotatedRegion |
| 751 | ATGAAAATCC.TTTTCACAAT | G | x | 34994_Tchad106_11 | UnannotatedRegion |
| 751 | ATGAAAATCC.TTTTCACAAT | G | x | 34995_8_2011 | UnannotatedRegion |
| 751 | ATGAAAATCC.TTTTCACAAT | G | x | 34996_10_2011 | UnannotatedRegion |
| 751 | ATGAAAATCC.TTTTCACAAT | G | x | 34997_39_2011 | UnannotatedRegion |
| 751 | ATGAAAATCC.TTTTCACAAT | G | x | 34998_59_2011 | UnannotatedRegion |
| 751 | ATGAAAATCC.TTTTCACAAT | G | x | 34999_63_2011 | UnannotatedRegion |
| 751 | ATGAAAATCC.TTTTCACAAT | G | x | 35000_75_2011 | UnannotatedRegion |
| 751 | ATGAAAATCC.TTTTCACAAT | G | x | 35001_76_2011 | UnannotatedRegion |
| 751 | ATGAAAATCC.TTTTCACAAT | G | x | 35002_120_2011 | UnannotatedRegion |
| 751 | ATGAAAATCC.TTTTCACAAT | G | x | 35003_403_2011 | UnannotatedRegion |
| 751 | ATGAAAATCC.TTTTCACAAT | G | x | 35004_12_15398_XS2_1 | UnannotatedRegion |
| 751 | ATGAAAATCC.TTTTCACAAT | G | x | 35005_12_13952_XS2_1 | UnannotatedRegion |
| 751 | ATGAAAATCC.TTTTCACAAT | G | x | 35006_12_15317_XS2_1 | UnannotatedRegion |
| 751 | ATGAAAATCC.TTTTCACAAT | G | x | 35007_12_15047_XS2_1 | UnannotatedRegion |
| 751 | ATGAAAATCC.TTTTCACAAT | G | x | 35008_12_15661_XS2_1 | UnannotatedRegion |
| 751 | ATGAAAATCC.TTTTCACAAT | G | x | 35009_12_15657_XS2_1 | UnannotatedRegion |
| 751 | ATGAAAATCC.TTTTCACAAT | G | x | 35010_12_17186_XS2_1 | UnannotatedRegion |
| 751 | ATGAAAATCC.TTTTCACAAT | G | x | 35011_12_17194_XS2_1 | UnannotatedRegion |
| 751 | ATGAAAATCC.TTTTCACAAT | G | x | 35012_12_17009_XS2_1 | UnannotatedRegion |
| 751 | ATGAAAATCC.TTTTCACAAT | G | x | 35013_12_14973_XS2_1 | UnannotatedRegion |
|  |  |  |  |  |  |
| 752 | ATGAATGAAG.ACCTTCGAAT | A | 2170025 F | 19260_WUE_2594 | UnannotatedRegion |
| 752 | ATGAATGAAG.ACCTTCGAAT | G | x | 34990_Tchad54_11 | UnannotatedRegion |
| 752 | ATGAATGAAG.ACCTTCGAAT | G | x | 34991_Tchad78_11 | UnannotatedRegion |
| 752 | ATGAATGAAG.ACCTTCGAAT | G | x | 34992_Tchad95_11 | UnannotatedRegion |
| 752 | ATGAATGAAG.ACCTTCGAAT | G | x | 34994_Tchad106_11 | UnannotatedRegion |
| 752 | ATGAATGAAG.ACCTTCGAAT | G | x | 34995_8_2011 | UnannotatedRegion |
| 752 | ATGAATGAAG.ACCTTCGAAT | G | x | 34996_10_2011 | UnannotatedRegion |
| 752 | ATGAATGAAG.ACCTTCGAAT | G | x | 34997_39_2011 | UnannotatedRegion |
| 752 | ATGAATGAAG.ACCTTCGAAT | G | x | 34998_59_2011 | UnannotatedRegion |
| 752 | ATGAATGAAG.ACCTTCGAAT | G | x | 34999_63_2011 | UnannotatedRegion |
| 752 | ATGAATGAAG.ACCTTCGAAT | G | x | 35000_75_2011 | UnannotatedRegion |
| 752 | ATGAATGAAG.ACCTTCGAAT | G | x | 35001_76_2011 | UnannotatedRegion |
| 752 | ATGAATGAAG.ACCTTCGAAT | G | x | 35002_120_2011 | UnannotatedRegion |
| 752 | ATGAATGAAG.ACCTTCGAAT | G | x | 35003_403_2011 | UnannotatedRegion |
| 752 | ATGAATGAAG.ACCTTCGAAT | G | x | 35004_12_15398_XS2_1 | UnannotatedRegion |
| 752 | ATGAATGAAG.ACCTTCGAAT | G | x | 35005_12_13952_XS2_1 | UnannotatedRegion |
| 752 | ATGAATGAAG.ACCTTCGAAT | G | x | 35006_12_15317_XS2_1 | UnannotatedRegion |
| 752 | ATGAATGAAG.ACCTTCGAAT | G | x | 35007_12_15047_XS2_1 | UnannotatedRegion |
| 752 | ATGAATGAAG.ACCTTCGAAT | G | x | 35008_12_15661_XS2_1 | UnannotatedRegion |
| 752 | ATGAATGAAG.ACCTTCGAAT | G | x | 35009_12_15657_XS2_1 | UnannotatedRegion |
| 752 | ATGAATGAAG.ACCTTCGAAT | G | x | 35010_12_17186_XS2_1 | UnannotatedRegion |
| 752 | ATGAATGAAG.ACCTTCGAAT | G | x | 35011_12_17194_XS2_1 | UnannotatedRegion |
| 752 | ATGAATGAAG.ACCTTCGAAT | G | x | 35012_12_17009_XS2_1 | UnannotatedRegion |
| 752 | ATGAATGAAG.ACCTTCGAAT | G | x | 35013_12_14973_XS2_1 | UnannotatedRegion |
|  |  |  |  |  |  |
| 762 | ATGCCCTGAC.CCCTGTTTTA | A | 1119068 R | 19260_WUE_2594 | UnannotatedRegion |
| 762 | ATGCCCTGAC.CCCTGTTTTA | C | x | 34990_Tchad54_11 | UnannotatedRegion |
| 762 | ATGCCCTGAC.CCCTGTTTTA | C | x | 34991_Tchad78_11 | UnannotatedRegion |
| 762 | ATGCCCTGAC.CCCTGTTTTA | C | x | 34992_Tchad95_11 | UnannotatedRegion |
| 762 | ATGCCCTGAC.CCCTGTTTTA | C | x | 34994_Tchad106_11 | UnannotatedRegion |
| 762 | ATGCCCTGAC.CCCTGTTTTA | C | x | 34995_8_2011 | UnannotatedRegion |
| 762 | ATGCCCTGAC.CCCTGTTTTA | C | x | 34996_10_2011 | UnannotatedRegion |
| 762 | ATGCCCTGAC.CCCTGTTTTA | C | x | 34997_39_2011 | UnannotatedRegion |
| 762 | ATGCCCTGAC.CCCTGTTTTA | C | x | 34998_59_2011 | UnannotatedRegion |
| 762 | ATGCCCTGAC.CCCTGTTTTA | C | x | 34999_63_2011 | UnannotatedRegion |
| 762 | ATGCCCTGAC.CCCTGTTTTA | C | x | 35000_75_2011 | UnannotatedRegion |
| 762 | ATGCCCTGAC.CCCTGTTTTA | C | x | 35001_76_2011 | UnannotatedRegion |
| 762 | ATGCCCTGAC.CCCTGTTTTA | C | x | 35002_120_2011 | UnannotatedRegion |
| 762 | ATGCCCTGAC.CCCTGTTTTA | C | x | 35003_403_2011 | UnannotatedRegion |
| 762 | ATGCCCTGAC.CCCTGTTTTA | C | x | 35004_12_15398_XS2_1 | UnannotatedRegion |
| 762 | ATGCCCTGAC.CCCTGTTTTA | C | x | 35005_12_13952_XS2_1 | UnannotatedRegion |
| 762 | ATGCCCTGAC.CCCTGTTTTA | C | x | 35006_12_15317_XS2_1 | UnannotatedRegion |
| 762 | ATGCCCTGAC.CCCTGTTTTA | C | x | 35007_12_15047_XS2_1 | UnannotatedRegion |
| 762 | ATGCCCTGAC.CCCTGTTTTA | C | x | 35008_12_15661_XS2_1 | UnannotatedRegion |
| 762 | ATGCCCTGAC.CCCTGTTTTA | C | x | 35009_12_15657_XS2_1 | UnannotatedRegion |
| 762 | ATGCCCTGAC.CCCTGTTTTA | C | x | 35010_12_17186_XS2_1 | UnannotatedRegion |
| 762 | ATGCCCTGAC.CCCTGTTTTA | C | x | 35011_12_17194_XS2_1 | UnannotatedRegion |
| 762 | ATGCCCTGAC.CCCTGTTTTA | C | x | 35012_12_17009_XS2_1 | UnannotatedRegion |
| 762 | ATGCCCTGAC.CCCTGTTTTA | C | x | 35013_12_14973_XS2_1 | UnannotatedRegion |
|  |  |  |  |  |  |
| 769 | ATGCCGTCTG.CCCCCTTCGG | A | 1478583 F | 19260_WUE_2594 | UnannotatedRegion |
| 769 | ATGCCGTCTG.CCCCCTTCGG | A | x | 34990_Tchad54_11 | UnannotatedRegion |
| 769 | ATGCCGTCTG.CCCCCTTCGG | A | x | 34991_Tchad78_11 | UnannotatedRegion |
| 769 | ATGCCGTCTG.CCCCCTTCGG | A | x | 34992_Tchad95_11 | UnannotatedRegion |
| 769 | ATGCCGTCTG.CCCCCTTCGG | A | x | 34994_Tchad106_11 | UnannotatedRegion |
| 769 | ATGCCGTCTG.CCCCCTTCGG | C | x | 34995_8_2011 | UnannotatedRegion |
| 769 | ATGCCGTCTG.CCCCCTTCGG | C | x | 34996_10_2011 | UnannotatedRegion |
| 769 | ATGCCGTCTG.CCCCCTTCGG | A | x | 34997_39_2011 | UnannotatedRegion |
| 769 | ATGCCGTCTG.CCCCCTTCGG | A | x | 34998_59_2011 | UnannotatedRegion |
| 769 | ATGCCGTCTG.CCCCCTTCGG | A | x | 34999_63_2011 | UnannotatedRegion |
| 769 | ATGCCGTCTG.CCCCCTTCGG | A | x | 35000_75_2011 | UnannotatedRegion |
| 769 | ATGCCGTCTG.CCCCCTTCGG | A | x | 35001_76_2011 | UnannotatedRegion |
| 769 | ATGCCGTCTG.CCCCCTTCGG | A | x | 35002_120_2011 | UnannotatedRegion |
| 769 | ATGCCGTCTG.CCCCCTTCGG | A | x | 35003_403_2011 | UnannotatedRegion |
| 769 | ATGCCGTCTG.CCCCCTTCGG | A | x | 35004_12_15398_XS2_1 | UnannotatedRegion |
| 769 | ATGCCGTCTG.CCCCCTTCGG | A | x | 35005_12_13952_XS2_1 | UnannotatedRegion |
| 769 | ATGCCGTCTG.CCCCCTTCGG | A | x | 35006_12_15317_XS2_1 | UnannotatedRegion |
| 769 | ATGCCGTCTG.CCCCCTTCGG | A | x | 35007_12_15047_XS2_1 | UnannotatedRegion |
| 769 | ATGCCGTCTG.CCCCCTTCGG | A | x | 35008_12_15661_XS2_1 | UnannotatedRegion |
| 769 | ATGCCGTCTG.CCCCCTTCGG | A | x | 35009_12_15657_XS2_1 | UnannotatedRegion |
| 769 | ATGCCGTCTG.CCCCCTTCGG | A | x | 35010_12_17186_XS2_1 | UnannotatedRegion |
| 769 | ATGCCGTCTG.CCCCCTTCGG | A | x | 35011_12_17194_XS2_1 | UnannotatedRegion |
| 769 | ATGCCGTCTG.CCCCCTTCGG | A | x | 35012_12_17009_XS2_1 | UnannotatedRegion |
| 769 | ATGCCGTCTG.CCCCCTTCGG | A | x | 35013_12_14973_XS2_1 | UnannotatedRegion |
|  |  |  |  |  |  |
| 790 | ATGTGTCGTG.ACCGCTTTTC | C | 1144614 F | 19260_WUE_2594 | UnannotatedRegion |
| 790 | ATGTGTCGTG.ACCGCTTTTC | T | x | 34990_Tchad54_11 | UnannotatedRegion |
| 790 | ATGTGTCGTG.ACCGCTTTTC | T | x | 34991_Tchad78_11 | UnannotatedRegion |
| 790 | ATGTGTCGTG.ACCGCTTTTC | T | x | 34992_Tchad95_11 | UnannotatedRegion |
| 790 | ATGTGTCGTG.ACCGCTTTTC | T | x | 34994_Tchad106_11 | UnannotatedRegion |
| 790 | ATGTGTCGTG.ACCGCTTTTC | T | x | 34995_8_2011 | UnannotatedRegion |
| 790 | ATGTGTCGTG.ACCGCTTTTC | T | x | 34996_10_2011 | UnannotatedRegion |
| 790 | ATGTGTCGTG.ACCGCTTTTC | T | x | 34997_39_2011 | UnannotatedRegion |
| 790 | ATGTGTCGTG.ACCGCTTTTC | T | x | 34998_59_2011 | UnannotatedRegion |
| 790 | ATGTGTCGTG.ACCGCTTTTC | T | x | 34999_63_2011 | UnannotatedRegion |
| 790 | ATGTGTCGTG.ACCGCTTTTC | T | x | 35000_75_2011 | UnannotatedRegion |
| 790 | ATGTGTCGTG.ACCGCTTTTC | T | x | 35001_76_2011 | UnannotatedRegion |
| 790 | ATGTGTCGTG.ACCGCTTTTC | T | x | 35002_120_2011 | UnannotatedRegion |
| 790 | ATGTGTCGTG.ACCGCTTTTC | T | x | 35003_403_2011 | UnannotatedRegion |
| 790 | ATGTGTCGTG.ACCGCTTTTC | T | x | 35004_12_15398_XS2_1 | UnannotatedRegion |
| 790 | ATGTGTCGTG.ACCGCTTTTC | T | x | 35005_12_13952_XS2_1 | UnannotatedRegion |
| 790 | ATGTGTCGTG.ACCGCTTTTC | T | x | 35006_12_15317_XS2_1 | UnannotatedRegion |
| 790 | ATGTGTCGTG.ACCGCTTTTC | T | x | 35007_12_15047_XS2_1 | UnannotatedRegion |
| 790 | ATGTGTCGTG.ACCGCTTTTC | T | x | 35008_12_15661_XS2_1 | UnannotatedRegion |
| 790 | ATGTGTCGTG.ACCGCTTTTC | T | x | 35009_12_15657_XS2_1 | UnannotatedRegion |
| 790 | ATGTGTCGTG.ACCGCTTTTC | T | x | 35010_12_17186_XS2_1 | UnannotatedRegion |
| 790 | ATGTGTCGTG.ACCGCTTTTC | T | x | 35011_12_17194_XS2_1 | UnannotatedRegion |
| 790 | ATGTGTCGTG.ACCGCTTTTC | T | x | 35012_12_17009_XS2_1 | UnannotatedRegion |
| 790 | ATGTGTCGTG.ACCGCTTTTC | T | x | 35013_12_14973_XS2_1 | UnannotatedRegion |
|  |  |  |  |  |  |
| 793 | ATGTTGTTTG.TTTCATTCTC | A | x | 34990_Tchad54_11 | NotInAnnotatedGenome |
| 793 | ATGTTGTTTG.TTTCATTCTC | A | x | 34991_Tchad78_11 | NotInAnnotatedGenome |
| 793 | ATGTTGTTTG.TTTCATTCTC | A | x | 34992_Tchad95_11 | NotInAnnotatedGenome |
| 793 | ATGTTGTTTG.TTTCATTCTC | A | x | 34994_Tchad106_11 | NotInAnnotatedGenome |
| 793 | ATGTTGTTTG.TTTCATTCTC | A | x | 34995_8_2011 | NotInAnnotatedGenome |
| 793 | ATGTTGTTTG.TTTCATTCTC | A | x | 34996_10_2011 | NotInAnnotatedGenome |
| 793 | ATGTTGTTTG.TTTCATTCTC | A | x | 34997_39_2011 | NotInAnnotatedGenome |
| 793 | ATGTTGTTTG.TTTCATTCTC | A | x | 34998_59_2011 | NotInAnnotatedGenome |
| 793 | ATGTTGTTTG.TTTCATTCTC | A | x | 34999_63_2011 | NotInAnnotatedGenome |
| 793 | ATGTTGTTTG.TTTCATTCTC | A | x | 35000_75_2011 | NotInAnnotatedGenome |
| 793 | ATGTTGTTTG.TTTCATTCTC | A | x | 35001_76_2011 | NotInAnnotatedGenome |
| 793 | ATGTTGTTTG.TTTCATTCTC | A | x | 35002_120_2011 | NotInAnnotatedGenome |
| 793 | ATGTTGTTTG.TTTCATTCTC | A | x | 35003_403_2011 | NotInAnnotatedGenome |
| 793 | ATGTTGTTTG.TTTCATTCTC | A | x | 35004_12_15398_XS2_1 | NotInAnnotatedGenome |
| 793 | ATGTTGTTTG.TTTCATTCTC | A | x | 35005_12_13952_XS2_1 | NotInAnnotatedGenome |
| 793 | ATGTTGTTTG.TTTCATTCTC | A | x | 35006_12_15317_XS2_1 | NotInAnnotatedGenome |
| 793 | ATGTTGTTTG.TTTCATTCTC | G | x | 35007_12_15047_XS2_1 | NotInAnnotatedGenome |
| 793 | ATGTTGTTTG.TTTCATTCTC | A | x | 35008_12_15661_XS2_1 | NotInAnnotatedGenome |
| 793 | ATGTTGTTTG.TTTCATTCTC | A | x | 35009_12_15657_XS2_1 | NotInAnnotatedGenome |
| 793 | ATGTTGTTTG.TTTCATTCTC | A | x | 35010_12_17186_XS2_1 | NotInAnnotatedGenome |
| 793 | ATGTTGTTTG.TTTCATTCTC | A | x | 35011_12_17194_XS2_1 | NotInAnnotatedGenome |
| 793 | ATGTTGTTTG.TTTCATTCTC | A | x | 35012_12_17009_XS2_1 | NotInAnnotatedGenome |
| 793 | ATGTTGTTTG.TTTCATTCTC | A | x | 35013_12_14973_XS2_1 | NotInAnnotatedGenome |
|  |  |  |  |  |  |
| 797 | ATTATATCAG.AAAAGCAAAC | G | 1599590 F | 19260_WUE_2594 | UnannotatedRegion |
| 797 | ATTATATCAG.AAAAGCAAAC | A | x | 34990_Tchad54_11 | UnannotatedRegion |
| 797 | ATTATATCAG.AAAAGCAAAC | A | x | 34991_Tchad78_11 | UnannotatedRegion |
| 797 | ATTATATCAG.AAAAGCAAAC | A | x | 34992_Tchad95_11 | UnannotatedRegion |
| 797 | ATTATATCAG.AAAAGCAAAC | A | x | 34995_8_2011 | UnannotatedRegion |
| 797 | ATTATATCAG.AAAAGCAAAC | A | x | 34996_10_2011 | UnannotatedRegion |
| 797 | ATTATATCAG.AAAAGCAAAC | A | x | 34997_39_2011 | UnannotatedRegion |
| 797 | ATTATATCAG.AAAAGCAAAC | A | x | 34998_59_2011 | UnannotatedRegion |
| 797 | ATTATATCAG.AAAAGCAAAC | A | x | 34999_63_2011 | UnannotatedRegion |
| 797 | ATTATATCAG.AAAAGCAAAC | A | x | 35000_75_2011 | UnannotatedRegion |
| 797 | ATTATATCAG.AAAAGCAAAC | A | x | 35001_76_2011 | UnannotatedRegion |
| 797 | ATTATATCAG.AAAAGCAAAC | A | x | 35002_120_2011 | UnannotatedRegion |
| 797 | ATTATATCAG.AAAAGCAAAC | A | x | 35003_403_2011 | UnannotatedRegion |
| 797 | ATTATATCAG.AAAAGCAAAC | A | x | 35004_12_15398_XS2_1 | UnannotatedRegion |
| 797 | ATTATATCAG.AAAAGCAAAC | A | x | 35005_12_13952_XS2_1 | UnannotatedRegion |
| 797 | ATTATATCAG.AAAAGCAAAC | A | x | 35006_12_15317_XS2_1 | UnannotatedRegion |
| 797 | ATTATATCAG.AAAAGCAAAC | A | x | 35007_12_15047_XS2_1 | UnannotatedRegion |
| 797 | ATTATATCAG.AAAAGCAAAC | A | x | 35008_12_15661_XS2_1 | UnannotatedRegion |
| 797 | ATTATATCAG.AAAAGCAAAC | A | x | 35009_12_15657_XS2_1 | UnannotatedRegion |
| 797 | ATTATATCAG.AAAAGCAAAC | A | x | 35010_12_17186_XS2_1 | UnannotatedRegion |
| 797 | ATTATATCAG.AAAAGCAAAC | A | x | 35011_12_17194_XS2_1 | UnannotatedRegion |
| 797 | ATTATATCAG.AAAAGCAAAC | A | x | 35012_12_17009_XS2_1 | UnannotatedRegion |
| 797 | ATTATATCAG.AAAAGCAAAC | A | x | 35013_12_14973_XS2_1 | UnannotatedRegion |
|  |  |  |  |  |  |
| 813 | ATTGCCAAAG.ATTTTACAGC | A | x | 34990_Tchad54_11 | NotInAnnotatedGenome |
| 813 | ATTGCCAAAG.ATTTTACAGC | A | x | 34991_Tchad78_11 | NotInAnnotatedGenome |
| 813 | ATTGCCAAAG.ATTTTACAGC | A | x | 34992_Tchad95_11 | NotInAnnotatedGenome |
| 813 | ATTGCCAAAG.ATTTTACAGC | A | x | 34994_Tchad106_11 | NotInAnnotatedGenome |
| 813 | ATTGCCAAAG.ATTTTACAGC | G | x | 34995_8_2011 | NotInAnnotatedGenome |
| 813 | ATTGCCAAAG.ATTTTACAGC | G | x | 34996_10_2011 | NotInAnnotatedGenome |
| 813 | ATTGCCAAAG.ATTTTACAGC | A | x | 34997_39_2011 | NotInAnnotatedGenome |
| 813 | ATTGCCAAAG.ATTTTACAGC | A | x | 34998_59_2011 | NotInAnnotatedGenome |
| 813 | ATTGCCAAAG.ATTTTACAGC | A | x | 34999_63_2011 | NotInAnnotatedGenome |
| 813 | ATTGCCAAAG.ATTTTACAGC | A | x | 35000_75_2011 | NotInAnnotatedGenome |
| 813 | ATTGCCAAAG.ATTTTACAGC | A | x | 35001_76_2011 | NotInAnnotatedGenome |
| 813 | ATTGCCAAAG.ATTTTACAGC | A | x | 35002_120_2011 | NotInAnnotatedGenome |
| 813 | ATTGCCAAAG.ATTTTACAGC | A | x | 35003_403_2011 | NotInAnnotatedGenome |
| 813 | ATTGCCAAAG.ATTTTACAGC | A | x | 35004_12_15398_XS2_1 | NotInAnnotatedGenome |
| 813 | ATTGCCAAAG.ATTTTACAGC | A | x | 35005_12_13952_XS2_1 | NotInAnnotatedGenome |
| 813 | ATTGCCAAAG.ATTTTACAGC | A | x | 35006_12_15317_XS2_1 | NotInAnnotatedGenome |
| 813 | ATTGCCAAAG.ATTTTACAGC | A | x | 35007_12_15047_XS2_1 | NotInAnnotatedGenome |
| 813 | ATTGCCAAAG.ATTTTACAGC | A | x | 35008_12_15661_XS2_1 | NotInAnnotatedGenome |
| 813 | ATTGCCAAAG.ATTTTACAGC | A | x | 35009_12_15657_XS2_1 | NotInAnnotatedGenome |
| 813 | ATTGCCAAAG.ATTTTACAGC | A | x | 35010_12_17186_XS2_1 | NotInAnnotatedGenome |
| 813 | ATTGCCAAAG.ATTTTACAGC | A | x | 35011_12_17194_XS2_1 | NotInAnnotatedGenome |
| 813 | ATTGCCAAAG.ATTTTACAGC | A | x | 35012_12_17009_XS2_1 | NotInAnnotatedGenome |
| 813 | ATTGCCAAAG.ATTTTACAGC | A | x | 35013_12_14973_XS2_1 | NotInAnnotatedGenome |
|  |  |  |  |  |  |
| 817 | ATTGTTTTGC.GGTTTACGGC | T | 24705 F | 19260_WUE_2594 | UnannotatedRegion |
| 817 | ATTGTTTTGC.GGTTTACGGC | C | x | 34990_Tchad54_11 | UnannotatedRegion |
| 817 | ATTGTTTTGC.GGTTTACGGC | C | x | 34991_Tchad78_11 | UnannotatedRegion |
| 817 | ATTGTTTTGC.GGTTTACGGC | C | x | 34992_Tchad95_11 | UnannotatedRegion |
| 817 | ATTGTTTTGC.GGTTTACGGC | C | x | 34994_Tchad106_11 | UnannotatedRegion |
| 817 | ATTGTTTTGC.GGTTTACGGC | C | x | 34995_8_2011 | UnannotatedRegion |
| 817 | ATTGTTTTGC.GGTTTACGGC | C | x | 34996_10_2011 | UnannotatedRegion |
| 817 | ATTGTTTTGC.GGTTTACGGC | C | x | 34997_39_2011 | UnannotatedRegion |
| 817 | ATTGTTTTGC.GGTTTACGGC | C | x | 34998_59_2011 | UnannotatedRegion |
| 817 | ATTGTTTTGC.GGTTTACGGC | C | x | 34999_63_2011 | UnannotatedRegion |
| 817 | ATTGTTTTGC.GGTTTACGGC | C | x | 35000_75_2011 | UnannotatedRegion |
| 817 | ATTGTTTTGC.GGTTTACGGC | C | x | 35001_76_2011 | UnannotatedRegion |
| 817 | ATTGTTTTGC.GGTTTACGGC | C | x | 35002_120_2011 | UnannotatedRegion |
| 817 | ATTGTTTTGC.GGTTTACGGC | C | x | 35003_403_2011 | UnannotatedRegion |
| 817 | ATTGTTTTGC.GGTTTACGGC | C | x | 35004_12_15398_XS2_1 | UnannotatedRegion |
| 817 | ATTGTTTTGC.GGTTTACGGC | C | x | 35005_12_13952_XS2_1 | UnannotatedRegion |
| 817 | ATTGTTTTGC.GGTTTACGGC | C | x | 35006_12_15317_XS2_1 | UnannotatedRegion |
| 817 | ATTGTTTTGC.GGTTTACGGC | C | x | 35007_12_15047_XS2_1 | UnannotatedRegion |
| 817 | ATTGTTTTGC.GGTTTACGGC | C | x | 35008_12_15661_XS2_1 | UnannotatedRegion |
| 817 | ATTGTTTTGC.GGTTTACGGC | C | x | 35009_12_15657_XS2_1 | UnannotatedRegion |
| 817 | ATTGTTTTGC.GGTTTACGGC | C | x | 35010_12_17186_XS2_1 | UnannotatedRegion |
| 817 | ATTGTTTTGC.GGTTTACGGC | C | x | 35011_12_17194_XS2_1 | UnannotatedRegion |
| 817 | ATTGTTTTGC.GGTTTACGGC | C | x | 35012_12_17009_XS2_1 | UnannotatedRegion |
| 817 | ATTGTTTTGC.GGTTTACGGC | C | x | 35013_12_14973_XS2_1 | UnannotatedRegion |
|  |  |  |  |  |  |
| 818 | ATTTAATATT.TTATAAGCAA | A | 2034475 R | 19260_WUE_2594 | UnannotatedRegion |
| 818 | ATTTAATATT.TTATAAGCAA | G | x | 34990_Tchad54_11 | UnannotatedRegion |
| 818 | ATTTAATATT.TTATAAGCAA | G | x | 34991_Tchad78_11 | UnannotatedRegion |
| 818 | ATTTAATATT.TTATAAGCAA | G | x | 34992_Tchad95_11 | UnannotatedRegion |
| 818 | ATTTAATATT.TTATAAGCAA | G | x | 34994_Tchad106_11 | UnannotatedRegion |
| 818 | ATTTAATATT.TTATAAGCAA | G | x | 34995_8_2011 | UnannotatedRegion |
| 818 | ATTTAATATT.TTATAAGCAA | G | x | 34996_10_2011 | UnannotatedRegion |
| 818 | ATTTAATATT.TTATAAGCAA | G | x | 34997_39_2011 | UnannotatedRegion |
| 818 | ATTTAATATT.TTATAAGCAA | G | x | 34998_59_2011 | UnannotatedRegion |
| 818 | ATTTAATATT.TTATAAGCAA | G | x | 34999_63_2011 | UnannotatedRegion |
| 818 | ATTTAATATT.TTATAAGCAA | G | x | 35000_75_2011 | UnannotatedRegion |
| 818 | ATTTAATATT.TTATAAGCAA | G | x | 35001_76_2011 | UnannotatedRegion |
| 818 | ATTTAATATT.TTATAAGCAA | G | x | 35002_120_2011 | UnannotatedRegion |
| 818 | ATTTAATATT.TTATAAGCAA | G | x | 35003_403_2011 | UnannotatedRegion |
| 818 | ATTTAATATT.TTATAAGCAA | G | x | 35004_12_15398_XS2_1 | UnannotatedRegion |
| 818 | ATTTAATATT.TTATAAGCAA | G | x | 35005_12_13952_XS2_1 | UnannotatedRegion |
| 818 | ATTTAATATT.TTATAAGCAA | G | x | 35006_12_15317_XS2_1 | UnannotatedRegion |
| 818 | ATTTAATATT.TTATAAGCAA | G | x | 35007_12_15047_XS2_1 | UnannotatedRegion |
| 818 | ATTTAATATT.TTATAAGCAA | G | x | 35008_12_15661_XS2_1 | UnannotatedRegion |
| 818 | ATTTAATATT.TTATAAGCAA | G | x | 35009_12_15657_XS2_1 | UnannotatedRegion |
| 818 | ATTTAATATT.TTATAAGCAA | G | x | 35010_12_17186_XS2_1 | UnannotatedRegion |
| 818 | ATTTAATATT.TTATAAGCAA | G | x | 35011_12_17194_XS2_1 | UnannotatedRegion |
| 818 | ATTTAATATT.TTATAAGCAA | G | x | 35012_12_17009_XS2_1 | UnannotatedRegion |
| 818 | ATTTAATATT.TTATAAGCAA | G | x | 35013_12_14973_XS2_1 | UnannotatedRegion |
|  |  |  |  |  |  |
| 819 | ATTTACAATA.CGGCCGATTC | T | 858333 F | 19260_WUE_2594 | UnannotatedRegion |
| 819 | ATTTACAATA.CGGCCGATTC | C | x | 34990_Tchad54_11 | UnannotatedRegion |
| 819 | ATTTACAATA.CGGCCGATTC | C | x | 34991_Tchad78_11 | UnannotatedRegion |
| 819 | ATTTACAATA.CGGCCGATTC | C | x | 34992_Tchad95_11 | UnannotatedRegion |
| 819 | ATTTACAATA.CGGCCGATTC | C | x | 34994_Tchad106_11 | UnannotatedRegion |
| 819 | ATTTACAATA.CGGCCGATTC | C | x | 34995_8_2011 | UnannotatedRegion |
| 819 | ATTTACAATA.CGGCCGATTC | C | x | 34996_10_2011 | UnannotatedRegion |
| 819 | ATTTACAATA.CGGCCGATTC | C | x | 34997_39_2011 | UnannotatedRegion |
| 819 | ATTTACAATA.CGGCCGATTC | C | x | 34998_59_2011 | UnannotatedRegion |
| 819 | ATTTACAATA.CGGCCGATTC | C | x | 34999_63_2011 | UnannotatedRegion |
| 819 | ATTTACAATA.CGGCCGATTC | C | x | 35000_75_2011 | UnannotatedRegion |
| 819 | ATTTACAATA.CGGCCGATTC | C | x | 35001_76_2011 | UnannotatedRegion |
| 819 | ATTTACAATA.CGGCCGATTC | C | x | 35002_120_2011 | UnannotatedRegion |
| 819 | ATTTACAATA.CGGCCGATTC | C | x | 35003_403_2011 | UnannotatedRegion |
| 819 | ATTTACAATA.CGGCCGATTC | C | x | 35004_12_15398_XS2_1 | UnannotatedRegion |
| 819 | ATTTACAATA.CGGCCGATTC | C | x | 35005_12_13952_XS2_1 | UnannotatedRegion |
| 819 | ATTTACAATA.CGGCCGATTC | C | x | 35006_12_15317_XS2_1 | UnannotatedRegion |
| 819 | ATTTACAATA.CGGCCGATTC | C | x | 35007_12_15047_XS2_1 | UnannotatedRegion |
| 819 | ATTTACAATA.CGGCCGATTC | C | x | 35008_12_15661_XS2_1 | UnannotatedRegion |
| 819 | ATTTACAATA.CGGCCGATTC | C | x | 35009_12_15657_XS2_1 | UnannotatedRegion |
| 819 | ATTTACAATA.CGGCCGATTC | C | x | 35010_12_17186_XS2_1 | UnannotatedRegion |
| 819 | ATTTACAATA.CGGCCGATTC | C | x | 35011_12_17194_XS2_1 | UnannotatedRegion |
| 819 | ATTTACAATA.CGGCCGATTC | C | x | 35012_12_17009_XS2_1 | UnannotatedRegion |
| 819 | ATTTACAATA.CGGCCGATTC | C | x | 35013_12_14973_XS2_1 | UnannotatedRegion |
|  |  |  |  |  |  |
| 820 | ATTTATTATA.AAATGAGAAA | T | 1143474 F | 19260_WUE_2594 | UnannotatedRegion |
| 820 | ATTTATTATA.AAATGAGAAA | G | x | 34990_Tchad54_11 | UnannotatedRegion |
| 820 | ATTTATTATA.AAATGAGAAA | G | x | 34991_Tchad78_11 | UnannotatedRegion |
| 820 | ATTTATTATA.AAATGAGAAA | G | x | 34992_Tchad95_11 | UnannotatedRegion |
| 820 | ATTTATTATA.AAATGAGAAA | G | x | 34994_Tchad106_11 | UnannotatedRegion |
| 820 | ATTTATTATA.AAATGAGAAA | G | x | 34995_8_2011 | UnannotatedRegion |
| 820 | ATTTATTATA.AAATGAGAAA | G | x | 34996_10_2011 | UnannotatedRegion |
| 820 | ATTTATTATA.AAATGAGAAA | G | x | 34997_39_2011 | UnannotatedRegion |
| 820 | ATTTATTATA.AAATGAGAAA | G | x | 34998_59_2011 | UnannotatedRegion |
| 820 | ATTTATTATA.AAATGAGAAA | G | x | 34999_63_2011 | UnannotatedRegion |
| 820 | ATTTATTATA.AAATGAGAAA | G | x | 35000_75_2011 | UnannotatedRegion |
| 820 | ATTTATTATA.AAATGAGAAA | G | x | 35001_76_2011 | UnannotatedRegion |
| 820 | ATTTATTATA.AAATGAGAAA | G | x | 35002_120_2011 | UnannotatedRegion |
| 820 | ATTTATTATA.AAATGAGAAA | G | x | 35003_403_2011 | UnannotatedRegion |
| 820 | ATTTATTATA.AAATGAGAAA | G | x | 35004_12_15398_XS2_1 | UnannotatedRegion |
| 820 | ATTTATTATA.AAATGAGAAA | G | x | 35005_12_13952_XS2_1 | UnannotatedRegion |
| 820 | ATTTATTATA.AAATGAGAAA | G | x | 35006_12_15317_XS2_1 | UnannotatedRegion |
| 820 | ATTTATTATA.AAATGAGAAA | G | x | 35007_12_15047_XS2_1 | UnannotatedRegion |
| 820 | ATTTATTATA.AAATGAGAAA | G | x | 35008_12_15661_XS2_1 | UnannotatedRegion |
| 820 | ATTTATTATA.AAATGAGAAA | G | x | 35009_12_15657_XS2_1 | UnannotatedRegion |
| 820 | ATTTATTATA.AAATGAGAAA | G | x | 35010_12_17186_XS2_1 | UnannotatedRegion |
| 820 | ATTTATTATA.AAATGAGAAA | G | x | 35011_12_17194_XS2_1 | UnannotatedRegion |
| 820 | ATTTATTATA.AAATGAGAAA | G | x | 35012_12_17009_XS2_1 | UnannotatedRegion |
| 820 | ATTTATTATA.AAATGAGAAA | G | x | 35013_12_14973_XS2_1 | UnannotatedRegion |
|  |  |  |  |  |  |
| 828 | ATTTGGATGA.TCCTGTTTGA | T | 2037563 R | 19260_WUE_2594 | UnannotatedRegion |
| 828 | ATTTGGATGA.TCCTGTTTGA | C | x | 34990_Tchad54_11 | UnannotatedRegion |
| 828 | ATTTGGATGA.TCCTGTTTGA | C | x | 34991_Tchad78_11 | UnannotatedRegion |
| 828 | ATTTGGATGA.TCCTGTTTGA | C | x | 34992_Tchad95_11 | UnannotatedRegion |
| 828 | ATTTGGATGA.TCCTGTTTGA | C | x | 34994_Tchad106_11 | UnannotatedRegion |
| 828 | ATTTGGATGA.TCCTGTTTGA | C | x | 34995_8_2011 | UnannotatedRegion |
| 828 | ATTTGGATGA.TCCTGTTTGA | C | x | 34996_10_2011 | UnannotatedRegion |
| 828 | ATTTGGATGA.TCCTGTTTGA | C | x | 34997_39_2011 | UnannotatedRegion |
| 828 | ATTTGGATGA.TCCTGTTTGA | C | x | 34998_59_2011 | UnannotatedRegion |
| 828 | ATTTGGATGA.TCCTGTTTGA | C | x | 34999_63_2011 | UnannotatedRegion |
| 828 | ATTTGGATGA.TCCTGTTTGA | C | x | 35000_75_2011 | UnannotatedRegion |
| 828 | ATTTGGATGA.TCCTGTTTGA | C | x | 35001_76_2011 | UnannotatedRegion |
| 828 | ATTTGGATGA.TCCTGTTTGA | C | x | 35002_120_2011 | UnannotatedRegion |
| 828 | ATTTGGATGA.TCCTGTTTGA | C | x | 35003_403_2011 | UnannotatedRegion |
| 828 | ATTTGGATGA.TCCTGTTTGA | C | x | 35004_12_15398_XS2_1 | UnannotatedRegion |
| 828 | ATTTGGATGA.TCCTGTTTGA | C | x | 35005_12_13952_XS2_1 | UnannotatedRegion |
| 828 | ATTTGGATGA.TCCTGTTTGA | C | x | 35006_12_15317_XS2_1 | UnannotatedRegion |
| 828 | ATTTGGATGA.TCCTGTTTGA | T | x | 35007_12_15047_XS2_1 | UnannotatedRegion |
| 828 | ATTTGGATGA.TCCTGTTTGA | C | x | 35008_12_15661_XS2_1 | UnannotatedRegion |
| 828 | ATTTGGATGA.TCCTGTTTGA | C | x | 35009_12_15657_XS2_1 | UnannotatedRegion |
| 828 | ATTTGGATGA.TCCTGTTTGA | C | x | 35010_12_17186_XS2_1 | UnannotatedRegion |
| 828 | ATTTGGATGA.TCCTGTTTGA | C | x | 35011_12_17194_XS2_1 | UnannotatedRegion |
| 828 | ATTTGGATGA.TCCTGTTTGA | C | x | 35012_12_17009_XS2_1 | UnannotatedRegion |
| 828 | ATTTGGATGA.TCCTGTTTGA | C | x | 35013_12_14973_XS2_1 | UnannotatedRegion |
|  |  |  |  |  |  |
| 832 | ATTTTGTAAG.ACCGGTTCAA | A | 858357 R | 19260_WUE_2594 | UnannotatedRegion |
| 832 | ATTTTGTAAG.ACCGGTTCAA | G | x | 34990_Tchad54_11 | UnannotatedRegion |
| 832 | ATTTTGTAAG.ACCGGTTCAA | G | x | 34991_Tchad78_11 | UnannotatedRegion |
| 832 | ATTTTGTAAG.ACCGGTTCAA | G | x | 34992_Tchad95_11 | UnannotatedRegion |
| 832 | ATTTTGTAAG.ACCGGTTCAA | G | x | 34994_Tchad106_11 | UnannotatedRegion |
| 832 | ATTTTGTAAG.ACCGGTTCAA | G | x | 34995_8_2011 | UnannotatedRegion |
| 832 | ATTTTGTAAG.ACCGGTTCAA | G | x | 34996_10_2011 | UnannotatedRegion |
| 832 | ATTTTGTAAG.ACCGGTTCAA | G | x | 34997_39_2011 | UnannotatedRegion |
| 832 | ATTTTGTAAG.ACCGGTTCAA | G | x | 34998_59_2011 | UnannotatedRegion |
| 832 | ATTTTGTAAG.ACCGGTTCAA | G | x | 34999_63_2011 | UnannotatedRegion |
| 832 | ATTTTGTAAG.ACCGGTTCAA | G | x | 35000_75_2011 | UnannotatedRegion |
| 832 | ATTTTGTAAG.ACCGGTTCAA | G | x | 35001_76_2011 | UnannotatedRegion |
| 832 | ATTTTGTAAG.ACCGGTTCAA | G | x | 35002_120_2011 | UnannotatedRegion |
| 832 | ATTTTGTAAG.ACCGGTTCAA | G | x | 35003_403_2011 | UnannotatedRegion |
| 832 | ATTTTGTAAG.ACCGGTTCAA | G | x | 35004_12_15398_XS2_1 | UnannotatedRegion |
| 832 | ATTTTGTAAG.ACCGGTTCAA | G | x | 35005_12_13952_XS2_1 | UnannotatedRegion |
| 832 | ATTTTGTAAG.ACCGGTTCAA | G | x | 35006_12_15317_XS2_1 | UnannotatedRegion |
| 832 | ATTTTGTAAG.ACCGGTTCAA | G | x | 35007_12_15047_XS2_1 | UnannotatedRegion |
| 832 | ATTTTGTAAG.ACCGGTTCAA | G | x | 35008_12_15661_XS2_1 | UnannotatedRegion |
| 832 | ATTTTGTAAG.ACCGGTTCAA | G | x | 35009_12_15657_XS2_1 | UnannotatedRegion |
| 832 | ATTTTGTAAG.ACCGGTTCAA | G | x | 35010_12_17186_XS2_1 | UnannotatedRegion |
| 832 | ATTTTGTAAG.ACCGGTTCAA | G | x | 35011_12_17194_XS2_1 | UnannotatedRegion |
| 832 | ATTTTGTAAG.ACCGGTTCAA | G | x | 35012_12_17009_XS2_1 | UnannotatedRegion |
| 832 | ATTTTGTAAG.ACCGGTTCAA | G | x | 35013_12_14973_XS2_1 | UnannotatedRegion |
|  |  |  |  |  |  |
| 833 | ATTTTTGCAT.CTTTTATGGG | C | 1158286 R | 19260_WUE_2594 | UnannotatedRegion |
| 833 | ATTTTTGCAT.CTTTTATGGG | C | x | 34990_Tchad54_11 | UnannotatedRegion |
| 833 | ATTTTTGCAT.CTTTTATGGG | C | x | 34991_Tchad78_11 | UnannotatedRegion |
| 833 | ATTTTTGCAT.CTTTTATGGG | C | x | 34992_Tchad95_11 | UnannotatedRegion |
| 833 | ATTTTTGCAT.CTTTTATGGG | C | x | 34994_Tchad106_11 | UnannotatedRegion |
| 833 | ATTTTTGCAT.CTTTTATGGG | T | x | 34995_8_2011 | UnannotatedRegion |
| 833 | ATTTTTGCAT.CTTTTATGGG | T | x | 34996_10_2011 | UnannotatedRegion |
| 833 | ATTTTTGCAT.CTTTTATGGG | C | x | 34997_39_2011 | UnannotatedRegion |
| 833 | ATTTTTGCAT.CTTTTATGGG | C | x | 34998_59_2011 | UnannotatedRegion |
| 833 | ATTTTTGCAT.CTTTTATGGG | C | x | 34999_63_2011 | UnannotatedRegion |
| 833 | ATTTTTGCAT.CTTTTATGGG | C | x | 35000_75_2011 | UnannotatedRegion |
| 833 | ATTTTTGCAT.CTTTTATGGG | C | x | 35001_76_2011 | UnannotatedRegion |
| 833 | ATTTTTGCAT.CTTTTATGGG | C | x | 35002_120_2011 | UnannotatedRegion |
| 833 | ATTTTTGCAT.CTTTTATGGG | C | x | 35003_403_2011 | UnannotatedRegion |
| 833 | ATTTTTGCAT.CTTTTATGGG | C | x | 35004_12_15398_XS2_1 | UnannotatedRegion |
| 833 | ATTTTTGCAT.CTTTTATGGG | C | x | 35005_12_13952_XS2_1 | UnannotatedRegion |
| 833 | ATTTTTGCAT.CTTTTATGGG | C | x | 35006_12_15317_XS2_1 | UnannotatedRegion |
| 833 | ATTTTTGCAT.CTTTTATGGG | C | x | 35007_12_15047_XS2_1 | UnannotatedRegion |
| 833 | ATTTTTGCAT.CTTTTATGGG | C | x | 35008_12_15661_XS2_1 | UnannotatedRegion |
| 833 | ATTTTTGCAT.CTTTTATGGG | C | x | 35009_12_15657_XS2_1 | UnannotatedRegion |
| 833 | ATTTTTGCAT.CTTTTATGGG | C | x | 35010_12_17186_XS2_1 | UnannotatedRegion |
| 833 | ATTTTTGCAT.CTTTTATGGG | C | x | 35011_12_17194_XS2_1 | UnannotatedRegion |
| 833 | ATTTTTGCAT.CTTTTATGGG | C | x | 35012_12_17009_XS2_1 | UnannotatedRegion |
| 833 | ATTTTTGCAT.CTTTTATGGG | C | x | 35013_12_14973_XS2_1 | UnannotatedRegion |
|  |  |  |  |  |  |
| 847 | CAAACGACGC.CTTTGTGGGG | A | 1062756 R | 19260_WUE_2594 | UnannotatedRegion |
| 847 | CAAACGACGC.CTTTGTGGGG | G | x | 34990_Tchad54_11 | UnannotatedRegion |
| 847 | CAAACGACGC.CTTTGTGGGG | G | x | 34991_Tchad78_11 | UnannotatedRegion |
| 847 | CAAACGACGC.CTTTGTGGGG | G | x | 34992_Tchad95_11 | UnannotatedRegion |
| 847 | CAAACGACGC.CTTTGTGGGG | G | x | 34994_Tchad106_11 | UnannotatedRegion |
| 847 | CAAACGACGC.CTTTGTGGGG | G | x | 34995_8_2011 | UnannotatedRegion |
| 847 | CAAACGACGC.CTTTGTGGGG | G | x | 34996_10_2011 | UnannotatedRegion |
| 847 | CAAACGACGC.CTTTGTGGGG | G | x | 34997_39_2011 | UnannotatedRegion |
| 847 | CAAACGACGC.CTTTGTGGGG | G | x | 34998_59_2011 | UnannotatedRegion |
| 847 | CAAACGACGC.CTTTGTGGGG | G | x | 34999_63_2011 | UnannotatedRegion |
| 847 | CAAACGACGC.CTTTGTGGGG | G | x | 35000_75_2011 | UnannotatedRegion |
| 847 | CAAACGACGC.CTTTGTGGGG | G | x | 35001_76_2011 | UnannotatedRegion |
| 847 | CAAACGACGC.CTTTGTGGGG | G | x | 35002_120_2011 | UnannotatedRegion |
| 847 | CAAACGACGC.CTTTGTGGGG | G | x | 35003_403_2011 | UnannotatedRegion |
| 847 | CAAACGACGC.CTTTGTGGGG | G | x | 35004_12_15398_XS2_1 | UnannotatedRegion |
| 847 | CAAACGACGC.CTTTGTGGGG | G | x | 35005_12_13952_XS2_1 | UnannotatedRegion |
| 847 | CAAACGACGC.CTTTGTGGGG | G | x | 35006_12_15317_XS2_1 | UnannotatedRegion |
| 847 | CAAACGACGC.CTTTGTGGGG | G | x | 35007_12_15047_XS2_1 | UnannotatedRegion |
| 847 | CAAACGACGC.CTTTGTGGGG | G | x | 35008_12_15661_XS2_1 | UnannotatedRegion |
| 847 | CAAACGACGC.CTTTGTGGGG | G | x | 35009_12_15657_XS2_1 | UnannotatedRegion |
| 847 | CAAACGACGC.CTTTGTGGGG | G | x | 35010_12_17186_XS2_1 | UnannotatedRegion |
| 847 | CAAACGACGC.CTTTGTGGGG | G | x | 35011_12_17194_XS2_1 | UnannotatedRegion |
| 847 | CAAACGACGC.CTTTGTGGGG | G | x | 35012_12_17009_XS2_1 | UnannotatedRegion |
| 847 | CAAACGACGC.CTTTGTGGGG | G | x | 35013_12_14973_XS2_1 | UnannotatedRegion |
|  |  |  |  |  |  |
| 862 | CAACAGAGCA.GGTTTGAAAC | T | 1843525 R | 19260_WUE_2594 | UnannotatedRegion |
| 862 | CAACAGAGCA.GGTTTGAAAC | C | x | 34990_Tchad54_11 | UnannotatedRegion |
| 862 | CAACAGAGCA.GGTTTGAAAC | C | x | 34991_Tchad78_11 | UnannotatedRegion |
| 862 | CAACAGAGCA.GGTTTGAAAC | C | x | 34992_Tchad95_11 | UnannotatedRegion |
| 862 | CAACAGAGCA.GGTTTGAAAC | C | x | 34994_Tchad106_11 | UnannotatedRegion |
| 862 | CAACAGAGCA.GGTTTGAAAC | C | x | 34995_8_2011 | UnannotatedRegion |
| 862 | CAACAGAGCA.GGTTTGAAAC | C | x | 34996_10_2011 | UnannotatedRegion |
| 862 | CAACAGAGCA.GGTTTGAAAC | C | x | 34997_39_2011 | UnannotatedRegion |
| 862 | CAACAGAGCA.GGTTTGAAAC | C | x | 34998_59_2011 | UnannotatedRegion |
| 862 | CAACAGAGCA.GGTTTGAAAC | C | x | 34999_63_2011 | UnannotatedRegion |
| 862 | CAACAGAGCA.GGTTTGAAAC | C | x | 35000_75_2011 | UnannotatedRegion |
| 862 | CAACAGAGCA.GGTTTGAAAC | C | x | 35001_76_2011 | UnannotatedRegion |
| 862 | CAACAGAGCA.GGTTTGAAAC | C | x | 35002_120_2011 | UnannotatedRegion |
| 862 | CAACAGAGCA.GGTTTGAAAC | C | x | 35003_403_2011 | UnannotatedRegion |
| 862 | CAACAGAGCA.GGTTTGAAAC | C | x | 35004_12_15398_XS2_1 | UnannotatedRegion |
| 862 | CAACAGAGCA.GGTTTGAAAC | C | x | 35005_12_13952_XS2_1 | UnannotatedRegion |
| 862 | CAACAGAGCA.GGTTTGAAAC | C | x | 35006_12_15317_XS2_1 | UnannotatedRegion |
| 862 | CAACAGAGCA.GGTTTGAAAC | C | x | 35007_12_15047_XS2_1 | UnannotatedRegion |
| 862 | CAACAGAGCA.GGTTTGAAAC | C | x | 35008_12_15661_XS2_1 | UnannotatedRegion |
| 862 | CAACAGAGCA.GGTTTGAAAC | C | x | 35009_12_15657_XS2_1 | UnannotatedRegion |
| 862 | CAACAGAGCA.GGTTTGAAAC | C | x | 35010_12_17186_XS2_1 | UnannotatedRegion |
| 862 | CAACAGAGCA.GGTTTGAAAC | C | x | 35011_12_17194_XS2_1 | UnannotatedRegion |
| 862 | CAACAGAGCA.GGTTTGAAAC | C | x | 35012_12_17009_XS2_1 | UnannotatedRegion |
| 862 | CAACAGAGCA.GGTTTGAAAC | C | x | 35013_12_14973_XS2_1 | UnannotatedRegion |
|  |  |  |  |  |  |
| 876 | CAAGCGGGCA.TCTAATCGCA | T | 114074 R | 19260_WUE_2594 | UnannotatedRegion |
| 876 | CAAGCGGGCA.TCTAATCGCA | T | x | 34990_Tchad54_11 | UnannotatedRegion |
| 876 | CAAGCGGGCA.TCTAATCGCA | T | x | 34991_Tchad78_11 | UnannotatedRegion |
| 876 | CAAGCGGGCA.TCTAATCGCA | T | x | 34992_Tchad95_11 | UnannotatedRegion |
| 876 | CAAGCGGGCA.TCTAATCGCA | T | x | 34994_Tchad106_11 | UnannotatedRegion |
| 876 | CAAGCGGGCA.TCTAATCGCA | C | x | 34995_8_2011 | UnannotatedRegion |
| 876 | CAAGCGGGCA.TCTAATCGCA | C | x | 34996_10_2011 | UnannotatedRegion |
| 876 | CAAGCGGGCA.TCTAATCGCA | T | x | 34997_39_2011 | UnannotatedRegion |
| 876 | CAAGCGGGCA.TCTAATCGCA | T | x | 34998_59_2011 | UnannotatedRegion |
| 876 | CAAGCGGGCA.TCTAATCGCA | T | x | 34999_63_2011 | UnannotatedRegion |
| 876 | CAAGCGGGCA.TCTAATCGCA | T | x | 35000_75_2011 | UnannotatedRegion |
| 876 | CAAGCGGGCA.TCTAATCGCA | T | x | 35001_76_2011 | UnannotatedRegion |
| 876 | CAAGCGGGCA.TCTAATCGCA | T | x | 35002_120_2011 | UnannotatedRegion |
| 876 | CAAGCGGGCA.TCTAATCGCA | T | x | 35003_403_2011 | UnannotatedRegion |
| 876 | CAAGCGGGCA.TCTAATCGCA | T | x | 35004_12_15398_XS2_1 | UnannotatedRegion |
| 876 | CAAGCGGGCA.TCTAATCGCA | T | x | 35005_12_13952_XS2_1 | UnannotatedRegion |
| 876 | CAAGCGGGCA.TCTAATCGCA | T | x | 35006_12_15317_XS2_1 | UnannotatedRegion |
| 876 | CAAGCGGGCA.TCTAATCGCA | T | x | 35007_12_15047_XS2_1 | UnannotatedRegion |
| 876 | CAAGCGGGCA.TCTAATCGCA | T | x | 35008_12_15661_XS2_1 | UnannotatedRegion |
| 876 | CAAGCGGGCA.TCTAATCGCA | T | x | 35009_12_15657_XS2_1 | UnannotatedRegion |
| 876 | CAAGCGGGCA.TCTAATCGCA | T | x | 35010_12_17186_XS2_1 | UnannotatedRegion |
| 876 | CAAGCGGGCA.TCTAATCGCA | T | x | 35011_12_17194_XS2_1 | UnannotatedRegion |
| 876 | CAAGCGGGCA.TCTAATCGCA | T | x | 35012_12_17009_XS2_1 | UnannotatedRegion |
| 876 | CAAGCGGGCA.TCTAATCGCA | T | x | 35013_12_14973_XS2_1 | UnannotatedRegion |
|  |  |  |  |  |  |
| 878 | CAATAATGTA.AGAAAATAAA | G | 2194506 R | 19260_WUE_2594 | UnannotatedRegion |
| 878 | CAATAATGTA.AGAAAATAAA | G | x | 34990_Tchad54_11 | UnannotatedRegion |
| 878 | CAATAATGTA.AGAAAATAAA | G | x | 34991_Tchad78_11 | UnannotatedRegion |
| 878 | CAATAATGTA.AGAAAATAAA | G | x | 34992_Tchad95_11 | UnannotatedRegion |
| 878 | CAATAATGTA.AGAAAATAAA | A | x | 34995_8_2011 | UnannotatedRegion |
| 878 | CAATAATGTA.AGAAAATAAA | A | x | 34996_10_2011 | UnannotatedRegion |
| 878 | CAATAATGTA.AGAAAATAAA | G | x | 34997_39_2011 | UnannotatedRegion |
| 878 | CAATAATGTA.AGAAAATAAA | G | x | 34998_59_2011 | UnannotatedRegion |
| 878 | CAATAATGTA.AGAAAATAAA | G | x | 34999_63_2011 | UnannotatedRegion |
| 878 | CAATAATGTA.AGAAAATAAA | G | x | 35000_75_2011 | UnannotatedRegion |
| 878 | CAATAATGTA.AGAAAATAAA | G | x | 35001_76_2011 | UnannotatedRegion |
| 878 | CAATAATGTA.AGAAAATAAA | G | x | 35002_120_2011 | UnannotatedRegion |
| 878 | CAATAATGTA.AGAAAATAAA | G | x | 35003_403_2011 | UnannotatedRegion |
| 878 | CAATAATGTA.AGAAAATAAA | G | x | 35004_12_15398_XS2_1 | UnannotatedRegion |
| 878 | CAATAATGTA.AGAAAATAAA | G | x | 35005_12_13952_XS2_1 | UnannotatedRegion |
| 878 | CAATAATGTA.AGAAAATAAA | G | x | 35006_12_15317_XS2_1 | UnannotatedRegion |
| 878 | CAATAATGTA.AGAAAATAAA | G | x | 35007_12_15047_XS2_1 | UnannotatedRegion |
| 878 | CAATAATGTA.AGAAAATAAA | G | x | 35008_12_15661_XS2_1 | UnannotatedRegion |
| 878 | CAATAATGTA.AGAAAATAAA | G | x | 35009_12_15657_XS2_1 | UnannotatedRegion |
| 878 | CAATAATGTA.AGAAAATAAA | G | x | 35010_12_17186_XS2_1 | UnannotatedRegion |
| 878 | CAATAATGTA.AGAAAATAAA | G | x | 35011_12_17194_XS2_1 | UnannotatedRegion |
| 878 | CAATAATGTA.AGAAAATAAA | G | x | 35012_12_17009_XS2_1 | UnannotatedRegion |
| 878 | CAATAATGTA.AGAAAATAAA | G | x | 35013_12_14973_XS2_1 | UnannotatedRegion |
|  |  |  |  |  |  |
| 882 | CAATCCGCCT.CCTTGTCGTC | A | 2072551 F | 19260_WUE_2594 | UnannotatedRegion |
| 882 | CAATCCGCCT.CCTTGTCGTC | G | x | 34990_Tchad54_11 | UnannotatedRegion |
| 882 | CAATCCGCCT.CCTTGTCGTC | G | x | 34991_Tchad78_11 | UnannotatedRegion |
| 882 | CAATCCGCCT.CCTTGTCGTC | G | x | 34992_Tchad95_11 | UnannotatedRegion |
| 882 | CAATCCGCCT.CCTTGTCGTC | G | x | 34994_Tchad106_11 | UnannotatedRegion |
| 882 | CAATCCGCCT.CCTTGTCGTC | G | x | 34995_8_2011 | UnannotatedRegion |
| 882 | CAATCCGCCT.CCTTGTCGTC | G | x | 34996_10_2011 | UnannotatedRegion |
| 882 | CAATCCGCCT.CCTTGTCGTC | G | x | 34997_39_2011 | UnannotatedRegion |
| 882 | CAATCCGCCT.CCTTGTCGTC | G | x | 34998_59_2011 | UnannotatedRegion |
| 882 | CAATCCGCCT.CCTTGTCGTC | G | x | 34999_63_2011 | UnannotatedRegion |
| 882 | CAATCCGCCT.CCTTGTCGTC | G | x | 35000_75_2011 | UnannotatedRegion |
| 882 | CAATCCGCCT.CCTTGTCGTC | G | x | 35001_76_2011 | UnannotatedRegion |
| 882 | CAATCCGCCT.CCTTGTCGTC | G | x | 35002_120_2011 | UnannotatedRegion |
| 882 | CAATCCGCCT.CCTTGTCGTC | G | x | 35003_403_2011 | UnannotatedRegion |
| 882 | CAATCCGCCT.CCTTGTCGTC | G | x | 35004_12_15398_XS2_1 | UnannotatedRegion |
| 882 | CAATCCGCCT.CCTTGTCGTC | G | x | 35005_12_13952_XS2_1 | UnannotatedRegion |
| 882 | CAATCCGCCT.CCTTGTCGTC | G | x | 35006_12_15317_XS2_1 | UnannotatedRegion |
| 882 | CAATCCGCCT.CCTTGTCGTC | G | x | 35007_12_15047_XS2_1 | UnannotatedRegion |
| 882 | CAATCCGCCT.CCTTGTCGTC | G | x | 35008_12_15661_XS2_1 | UnannotatedRegion |
| 882 | CAATCCGCCT.CCTTGTCGTC | G | x | 35009_12_15657_XS2_1 | UnannotatedRegion |
| 882 | CAATCCGCCT.CCTTGTCGTC | G | x | 35010_12_17186_XS2_1 | UnannotatedRegion |
| 882 | CAATCCGCCT.CCTTGTCGTC | G | x | 35011_12_17194_XS2_1 | UnannotatedRegion |
| 882 | CAATCCGCCT.CCTTGTCGTC | G | x | 35012_12_17009_XS2_1 | UnannotatedRegion |
| 882 | CAATCCGCCT.CCTTGTCGTC | G | x | 35013_12_14973_XS2_1 | UnannotatedRegion |
|  |  |  |  |  |  |
| 886 | CAATGCGGCA.TTTGAATGCG | G | 1619651 R | 19260_WUE_2594 | UnannotatedRegion |
| 886 | CAATGCGGCA.TTTGAATGCG | A | x | 34990_Tchad54_11 | UnannotatedRegion |
| 886 | CAATGCGGCA.TTTGAATGCG | A | x | 34991_Tchad78_11 | UnannotatedRegion |
| 886 | CAATGCGGCA.TTTGAATGCG | A | x | 34992_Tchad95_11 | UnannotatedRegion |
| 886 | CAATGCGGCA.TTTGAATGCG | A | x | 34994_Tchad106_11 | UnannotatedRegion |
| 886 | CAATGCGGCA.TTTGAATGCG | A | x | 34995_8_2011 | UnannotatedRegion |
| 886 | CAATGCGGCA.TTTGAATGCG | A | x | 34996_10_2011 | UnannotatedRegion |
| 886 | CAATGCGGCA.TTTGAATGCG | A | x | 34997_39_2011 | UnannotatedRegion |
| 886 | CAATGCGGCA.TTTGAATGCG | A | x | 34998_59_2011 | UnannotatedRegion |
| 886 | CAATGCGGCA.TTTGAATGCG | A | x | 34999_63_2011 | UnannotatedRegion |
| 886 | CAATGCGGCA.TTTGAATGCG | A | x | 35000_75_2011 | UnannotatedRegion |
| 886 | CAATGCGGCA.TTTGAATGCG | A | x | 35001_76_2011 | UnannotatedRegion |
| 886 | CAATGCGGCA.TTTGAATGCG | A | x | 35002_120_2011 | UnannotatedRegion |
| 886 | CAATGCGGCA.TTTGAATGCG | A | x | 35003_403_2011 | UnannotatedRegion |
| 886 | CAATGCGGCA.TTTGAATGCG | A | x | 35004_12_15398_XS2_1 | UnannotatedRegion |
| 886 | CAATGCGGCA.TTTGAATGCG | A | x | 35005_12_13952_XS2_1 | UnannotatedRegion |
| 886 | CAATGCGGCA.TTTGAATGCG | A | x | 35006_12_15317_XS2_1 | UnannotatedRegion |
| 886 | CAATGCGGCA.TTTGAATGCG | A | x | 35007_12_15047_XS2_1 | UnannotatedRegion |
| 886 | CAATGCGGCA.TTTGAATGCG | A | x | 35008_12_15661_XS2_1 | UnannotatedRegion |
| 886 | CAATGCGGCA.TTTGAATGCG | A | x | 35009_12_15657_XS2_1 | UnannotatedRegion |
| 886 | CAATGCGGCA.TTTGAATGCG | A | x | 35010_12_17186_XS2_1 | UnannotatedRegion |
| 886 | CAATGCGGCA.TTTGAATGCG | A | x | 35011_12_17194_XS2_1 | UnannotatedRegion |
| 886 | CAATGCGGCA.TTTGAATGCG | A | x | 35012_12_17009_XS2_1 | UnannotatedRegion |
| 886 | CAATGCGGCA.TTTGAATGCG | A | x | 35013_12_14973_XS2_1 | UnannotatedRegion |
|  |  |  |  |  |  |
| 895 | CACAAGCGCA.GAAAGCATTA | C | 1214837 F | 19260_WUE_2594 | UnannotatedRegion |
| 895 | CACAAGCGCA.GAAAGCATTA | T | x | 34990_Tchad54_11 | UnannotatedRegion |
| 895 | CACAAGCGCA.GAAAGCATTA | T | x | 34991_Tchad78_11 | UnannotatedRegion |
| 895 | CACAAGCGCA.GAAAGCATTA | T | x | 34992_Tchad95_11 | UnannotatedRegion |
| 895 | CACAAGCGCA.GAAAGCATTA | T | x | 34994_Tchad106_11 | UnannotatedRegion |
| 895 | CACAAGCGCA.GAAAGCATTA | T | x | 34995_8_2011 | UnannotatedRegion |
| 895 | CACAAGCGCA.GAAAGCATTA | T | x | 34996_10_2011 | UnannotatedRegion |
| 895 | CACAAGCGCA.GAAAGCATTA | T | x | 34997_39_2011 | UnannotatedRegion |
| 895 | CACAAGCGCA.GAAAGCATTA | T | x | 34998_59_2011 | UnannotatedRegion |
| 895 | CACAAGCGCA.GAAAGCATTA | T | x | 34999_63_2011 | UnannotatedRegion |
| 895 | CACAAGCGCA.GAAAGCATTA | T | x | 35000_75_2011 | UnannotatedRegion |
| 895 | CACAAGCGCA.GAAAGCATTA | T | x | 35001_76_2011 | UnannotatedRegion |
| 895 | CACAAGCGCA.GAAAGCATTA | T | x | 35002_120_2011 | UnannotatedRegion |
| 895 | CACAAGCGCA.GAAAGCATTA | T | x | 35003_403_2011 | UnannotatedRegion |
| 895 | CACAAGCGCA.GAAAGCATTA | T | x | 35004_12_15398_XS2_1 | UnannotatedRegion |
| 895 | CACAAGCGCA.GAAAGCATTA | T | x | 35005_12_13952_XS2_1 | UnannotatedRegion |
| 895 | CACAAGCGCA.GAAAGCATTA | T | x | 35006_12_15317_XS2_1 | UnannotatedRegion |
| 895 | CACAAGCGCA.GAAAGCATTA | T | x | 35007_12_15047_XS2_1 | UnannotatedRegion |
| 895 | CACAAGCGCA.GAAAGCATTA | T | x | 35008_12_15661_XS2_1 | UnannotatedRegion |
| 895 | CACAAGCGCA.GAAAGCATTA | T | x | 35009_12_15657_XS2_1 | UnannotatedRegion |
| 895 | CACAAGCGCA.GAAAGCATTA | T | x | 35010_12_17186_XS2_1 | UnannotatedRegion |
| 895 | CACAAGCGCA.GAAAGCATTA | T | x | 35011_12_17194_XS2_1 | UnannotatedRegion |
| 895 | CACAAGCGCA.GAAAGCATTA | T | x | 35012_12_17009_XS2_1 | UnannotatedRegion |
| 895 | CACAAGCGCA.GAAAGCATTA | T | x | 35013_12_14973_XS2_1 | UnannotatedRegion |
|  |  |  |  |  |  |
| 914 | CACTTTCAGA.AACTTACAGC | T | 2103713 F | 19260_WUE_2594 | UnannotatedRegion |
| 914 | CACTTTCAGA.AACTTACAGC | C | x | 34990_Tchad54_11 | UnannotatedRegion |
| 914 | CACTTTCAGA.AACTTACAGC | T | x | 34991_Tchad78_11 | UnannotatedRegion |
| 914 | CACTTTCAGA.AACTTACAGC | T | x | 34992_Tchad95_11 | UnannotatedRegion |
| 914 | CACTTTCAGA.AACTTACAGC | T | x | 34994_Tchad106_11 | UnannotatedRegion |
| 914 | CACTTTCAGA.AACTTACAGC | T | x | 34995_8_2011 | UnannotatedRegion |
| 914 | CACTTTCAGA.AACTTACAGC | T | x | 34996_10_2011 | UnannotatedRegion |
| 914 | CACTTTCAGA.AACTTACAGC | C | x | 34997_39_2011 | UnannotatedRegion |
| 914 | CACTTTCAGA.AACTTACAGC | C | x | 34998_59_2011 | UnannotatedRegion |
| 914 | CACTTTCAGA.AACTTACAGC | C | x | 34999_63_2011 | UnannotatedRegion |
| 914 | CACTTTCAGA.AACTTACAGC | C | x | 35000_75_2011 | UnannotatedRegion |
| 914 | CACTTTCAGA.AACTTACAGC | C | x | 35001_76_2011 | UnannotatedRegion |
| 914 | CACTTTCAGA.AACTTACAGC | C | x | 35002_120_2011 | UnannotatedRegion |
| 914 | CACTTTCAGA.AACTTACAGC | T | x | 35003_403_2011 | UnannotatedRegion |
| 914 | CACTTTCAGA.AACTTACAGC | C | x | 35004_12_15398_XS2_1 | UnannotatedRegion |
| 914 | CACTTTCAGA.AACTTACAGC | C | x | 35005_12_13952_XS2_1 | UnannotatedRegion |
| 914 | CACTTTCAGA.AACTTACAGC | C | x | 35006_12_15317_XS2_1 | UnannotatedRegion |
| 914 | CACTTTCAGA.AACTTACAGC | T | x | 35007_12_15047_XS2_1 | UnannotatedRegion |
| 914 | CACTTTCAGA.AACTTACAGC | T | x | 35008_12_15661_XS2_1 | UnannotatedRegion |
| 914 | CACTTTCAGA.AACTTACAGC | T | x | 35009_12_15657_XS2_1 | UnannotatedRegion |
| 914 | CACTTTCAGA.AACTTACAGC | C | x | 35010_12_17186_XS2_1 | UnannotatedRegion |
| 914 | CACTTTCAGA.AACTTACAGC | C | x | 35011_12_17194_XS2_1 | UnannotatedRegion |
| 914 | CACTTTCAGA.AACTTACAGC | C | x | 35012_12_17009_XS2_1 | UnannotatedRegion |
| 914 | CACTTTCAGA.AACTTACAGC | T | x | 35013_12_14973_XS2_1 | UnannotatedRegion |
|  |  |  |  |  |  |
| 919 | CAGAATGGCT.GGATGGGGCG | C | 858312 R | 19260_WUE_2594 | UnannotatedRegion |
| 919 | CAGAATGGCT.GGATGGGGCG | A | x | 34990_Tchad54_11 | UnannotatedRegion |
| 919 | CAGAATGGCT.GGATGGGGCG | A | x | 34991_Tchad78_11 | UnannotatedRegion |
| 919 | CAGAATGGCT.GGATGGGGCG | A | x | 34992_Tchad95_11 | UnannotatedRegion |
| 919 | CAGAATGGCT.GGATGGGGCG | A | x | 34994_Tchad106_11 | UnannotatedRegion |
| 919 | CAGAATGGCT.GGATGGGGCG | A | x | 34995_8_2011 | UnannotatedRegion |
| 919 | CAGAATGGCT.GGATGGGGCG | A | x | 34996_10_2011 | UnannotatedRegion |
| 919 | CAGAATGGCT.GGATGGGGCG | A | x | 34997_39_2011 | UnannotatedRegion |
| 919 | CAGAATGGCT.GGATGGGGCG | A | x | 34998_59_2011 | UnannotatedRegion |
| 919 | CAGAATGGCT.GGATGGGGCG | A | x | 34999_63_2011 | UnannotatedRegion |
| 919 | CAGAATGGCT.GGATGGGGCG | A | x | 35000_75_2011 | UnannotatedRegion |
| 919 | CAGAATGGCT.GGATGGGGCG | A | x | 35001_76_2011 | UnannotatedRegion |
| 919 | CAGAATGGCT.GGATGGGGCG | A | x | 35002_120_2011 | UnannotatedRegion |
| 919 | CAGAATGGCT.GGATGGGGCG | A | x | 35003_403_2011 | UnannotatedRegion |
| 919 | CAGAATGGCT.GGATGGGGCG | A | x | 35004_12_15398_XS2_1 | UnannotatedRegion |
| 919 | CAGAATGGCT.GGATGGGGCG | A | x | 35005_12_13952_XS2_1 | UnannotatedRegion |
| 919 | CAGAATGGCT.GGATGGGGCG | A | x | 35006_12_15317_XS2_1 | UnannotatedRegion |
| 919 | CAGAATGGCT.GGATGGGGCG | A | x | 35007_12_15047_XS2_1 | UnannotatedRegion |
| 919 | CAGAATGGCT.GGATGGGGCG | A | x | 35008_12_15661_XS2_1 | UnannotatedRegion |
| 919 | CAGAATGGCT.GGATGGGGCG | A | x | 35009_12_15657_XS2_1 | UnannotatedRegion |
| 919 | CAGAATGGCT.GGATGGGGCG | A | x | 35010_12_17186_XS2_1 | UnannotatedRegion |
| 919 | CAGAATGGCT.GGATGGGGCG | A | x | 35011_12_17194_XS2_1 | UnannotatedRegion |
| 919 | CAGAATGGCT.GGATGGGGCG | A | x | 35012_12_17009_XS2_1 | UnannotatedRegion |
| 919 | CAGAATGGCT.GGATGGGGCG | A | x | 35013_12_14973_XS2_1 | UnannotatedRegion |
|  |  |  |  |  |  |
| 923 | CAGACGGCAT.TGTTTACCAA | C | 1099202 F | 19260_WUE_2594 | UnannotatedRegion |
| 923 | CAGACGGCAT.TGTTTACCAA | T | x | 34990_Tchad54_11 | UnannotatedRegion |
| 923 | CAGACGGCAT.TGTTTACCAA | T | x | 34991_Tchad78_11 | UnannotatedRegion |
| 923 | CAGACGGCAT.TGTTTACCAA | T | x | 34992_Tchad95_11 | UnannotatedRegion |
| 923 | CAGACGGCAT.TGTTTACCAA | T | x | 34994_Tchad106_11 | UnannotatedRegion |
| 923 | CAGACGGCAT.TGTTTACCAA | T | x | 34995_8_2011 | UnannotatedRegion |
| 923 | CAGACGGCAT.TGTTTACCAA | T | x | 34996_10_2011 | UnannotatedRegion |
| 923 | CAGACGGCAT.TGTTTACCAA | T | x | 34997_39_2011 | UnannotatedRegion |
| 923 | CAGACGGCAT.TGTTTACCAA | T | x | 34998_59_2011 | UnannotatedRegion |
| 923 | CAGACGGCAT.TGTTTACCAA | T | x | 34999_63_2011 | UnannotatedRegion |
| 923 | CAGACGGCAT.TGTTTACCAA | T | x | 35000_75_2011 | UnannotatedRegion |
| 923 | CAGACGGCAT.TGTTTACCAA | T | x | 35001_76_2011 | UnannotatedRegion |
| 923 | CAGACGGCAT.TGTTTACCAA | T | x | 35002_120_2011 | UnannotatedRegion |
| 923 | CAGACGGCAT.TGTTTACCAA | T | x | 35003_403_2011 | UnannotatedRegion |
| 923 | CAGACGGCAT.TGTTTACCAA | T | x | 35004_12_15398_XS2_1 | UnannotatedRegion |
| 923 | CAGACGGCAT.TGTTTACCAA | T | x | 35005_12_13952_XS2_1 | UnannotatedRegion |
| 923 | CAGACGGCAT.TGTTTACCAA | T | x | 35006_12_15317_XS2_1 | UnannotatedRegion |
| 923 | CAGACGGCAT.TGTTTACCAA | T | x | 35007_12_15047_XS2_1 | UnannotatedRegion |
| 923 | CAGACGGCAT.TGTTTACCAA | T | x | 35008_12_15661_XS2_1 | UnannotatedRegion |
| 923 | CAGACGGCAT.TGTTTACCAA | T | x | 35009_12_15657_XS2_1 | UnannotatedRegion |
| 923 | CAGACGGCAT.TGTTTACCAA | T | x | 35010_12_17186_XS2_1 | UnannotatedRegion |
| 923 | CAGACGGCAT.TGTTTACCAA | T | x | 35011_12_17194_XS2_1 | UnannotatedRegion |
| 923 | CAGACGGCAT.TGTTTACCAA | T | x | 35012_12_17009_XS2_1 | UnannotatedRegion |
| 923 | CAGACGGCAT.TGTTTACCAA | T | x | 35013_12_14973_XS2_1 | UnannotatedRegion |
|  |  |  |  |  |  |
| 937 | CAGGTCATCC.CCCCGCAAAA | A | 701580 R | 19260_WUE_2594 | UnannotatedRegion |
| 937 | CAGGTCATCC.CCCCGCAAAA | G | x | 34990_Tchad54_11 | UnannotatedRegion |
| 937 | CAGGTCATCC.CCCCGCAAAA | G | x | 34991_Tchad78_11 | UnannotatedRegion |
| 937 | CAGGTCATCC.CCCCGCAAAA | G | x | 34992_Tchad95_11 | UnannotatedRegion |
| 937 | CAGGTCATCC.CCCCGCAAAA | G | x | 34994_Tchad106_11 | UnannotatedRegion |
| 937 | CAGGTCATCC.CCCCGCAAAA | G | x | 34995_8_2011 | UnannotatedRegion |
| 937 | CAGGTCATCC.CCCCGCAAAA | G | x | 34996_10_2011 | UnannotatedRegion |
| 937 | CAGGTCATCC.CCCCGCAAAA | G | x | 34997_39_2011 | UnannotatedRegion |
| 937 | CAGGTCATCC.CCCCGCAAAA | G | x | 34998_59_2011 | UnannotatedRegion |
| 937 | CAGGTCATCC.CCCCGCAAAA | G | x | 34999_63_2011 | UnannotatedRegion |
| 937 | CAGGTCATCC.CCCCGCAAAA | G | x | 35000_75_2011 | UnannotatedRegion |
| 937 | CAGGTCATCC.CCCCGCAAAA | G | x | 35001_76_2011 | UnannotatedRegion |
| 937 | CAGGTCATCC.CCCCGCAAAA | G | x | 35002_120_2011 | UnannotatedRegion |
| 937 | CAGGTCATCC.CCCCGCAAAA | G | x | 35003_403_2011 | UnannotatedRegion |
| 937 | CAGGTCATCC.CCCCGCAAAA | G | x | 35004_12_15398_XS2_1 | UnannotatedRegion |
| 937 | CAGGTCATCC.CCCCGCAAAA | G | x | 35005_12_13952_XS2_1 | UnannotatedRegion |
| 937 | CAGGTCATCC.CCCCGCAAAA | G | x | 35006_12_15317_XS2_1 | UnannotatedRegion |
| 937 | CAGGTCATCC.CCCCGCAAAA | G | x | 35007_12_15047_XS2_1 | UnannotatedRegion |
| 937 | CAGGTCATCC.CCCCGCAAAA | G | x | 35008_12_15661_XS2_1 | UnannotatedRegion |
| 937 | CAGGTCATCC.CCCCGCAAAA | G | x | 35009_12_15657_XS2_1 | UnannotatedRegion |
| 937 | CAGGTCATCC.CCCCGCAAAA | G | x | 35010_12_17186_XS2_1 | UnannotatedRegion |
| 937 | CAGGTCATCC.CCCCGCAAAA | G | x | 35011_12_17194_XS2_1 | UnannotatedRegion |
| 937 | CAGGTCATCC.CCCCGCAAAA | G | x | 35012_12_17009_XS2_1 | UnannotatedRegion |
| 937 | CAGGTCATCC.CCCCGCAAAA | G | x | 35013_12_14973_XS2_1 | UnannotatedRegion |
|  |  |  |  |  |  |
| 946 | CATAGCAGGC.GATAATGGAG | A | 675652 R | 19260_WUE_2594 | UnannotatedRegion |
| 946 | CATAGCAGGC.GATAATGGAG | G | x | 34990_Tchad54_11 | UnannotatedRegion |
| 946 | CATAGCAGGC.GATAATGGAG | G | x | 34991_Tchad78_11 | UnannotatedRegion |
| 946 | CATAGCAGGC.GATAATGGAG | G | x | 34992_Tchad95_11 | UnannotatedRegion |
| 946 | CATAGCAGGC.GATAATGGAG | G | x | 34994_Tchad106_11 | UnannotatedRegion |
| 946 | CATAGCAGGC.GATAATGGAG | G | x | 34995_8_2011 | UnannotatedRegion |
| 946 | CATAGCAGGC.GATAATGGAG | G | x | 34996_10_2011 | UnannotatedRegion |
| 946 | CATAGCAGGC.GATAATGGAG | G | x | 34997_39_2011 | UnannotatedRegion |
| 946 | CATAGCAGGC.GATAATGGAG | G | x | 34998_59_2011 | UnannotatedRegion |
| 946 | CATAGCAGGC.GATAATGGAG | G | x | 34999_63_2011 | UnannotatedRegion |
| 946 | CATAGCAGGC.GATAATGGAG | G | x | 35000_75_2011 | UnannotatedRegion |
| 946 | CATAGCAGGC.GATAATGGAG | G | x | 35001_76_2011 | UnannotatedRegion |
| 946 | CATAGCAGGC.GATAATGGAG | G | x | 35002_120_2011 | UnannotatedRegion |
| 946 | CATAGCAGGC.GATAATGGAG | G | x | 35003_403_2011 | UnannotatedRegion |
| 946 | CATAGCAGGC.GATAATGGAG | G | x | 35004_12_15398_XS2_1 | UnannotatedRegion |
| 946 | CATAGCAGGC.GATAATGGAG | G | x | 35005_12_13952_XS2_1 | UnannotatedRegion |
| 946 | CATAGCAGGC.GATAATGGAG | G | x | 35006_12_15317_XS2_1 | UnannotatedRegion |
| 946 | CATAGCAGGC.GATAATGGAG | G | x | 35007_12_15047_XS2_1 | UnannotatedRegion |
| 946 | CATAGCAGGC.GATAATGGAG | G | x | 35008_12_15661_XS2_1 | UnannotatedRegion |
| 946 | CATAGCAGGC.GATAATGGAG | G | x | 35009_12_15657_XS2_1 | UnannotatedRegion |
| 946 | CATAGCAGGC.GATAATGGAG | G | x | 35010_12_17186_XS2_1 | UnannotatedRegion |
| 946 | CATAGCAGGC.GATAATGGAG | G | x | 35011_12_17194_XS2_1 | UnannotatedRegion |
| 946 | CATAGCAGGC.GATAATGGAG | G | x | 35012_12_17009_XS2_1 | UnannotatedRegion |
| 946 | CATAGCAGGC.GATAATGGAG | G | x | 35013_12_14973_XS2_1 | UnannotatedRegion |
|  |  |  |  |  |  |
| 958 | CATCGCCTTG.CAATCGGCGC | G | 1146818 F | 19260_WUE_2594 | UnannotatedRegion |
| 958 | CATCGCCTTG.CAATCGGCGC | A | x | 34990_Tchad54_11 | UnannotatedRegion |
| 958 | CATCGCCTTG.CAATCGGCGC | A | x | 34991_Tchad78_11 | UnannotatedRegion |
| 958 | CATCGCCTTG.CAATCGGCGC | A | x | 34992_Tchad95_11 | UnannotatedRegion |
| 958 | CATCGCCTTG.CAATCGGCGC | A | x | 34994_Tchad106_11 | UnannotatedRegion |
| 958 | CATCGCCTTG.CAATCGGCGC | A | x | 34995_8_2011 | UnannotatedRegion |
| 958 | CATCGCCTTG.CAATCGGCGC | A | x | 34996_10_2011 | UnannotatedRegion |
| 958 | CATCGCCTTG.CAATCGGCGC | A | x | 34997_39_2011 | UnannotatedRegion |
| 958 | CATCGCCTTG.CAATCGGCGC | A | x | 34998_59_2011 | UnannotatedRegion |
| 958 | CATCGCCTTG.CAATCGGCGC | A | x | 34999_63_2011 | UnannotatedRegion |
| 958 | CATCGCCTTG.CAATCGGCGC | A | x | 35000_75_2011 | UnannotatedRegion |
| 958 | CATCGCCTTG.CAATCGGCGC | A | x | 35001_76_2011 | UnannotatedRegion |
| 958 | CATCGCCTTG.CAATCGGCGC | A | x | 35002_120_2011 | UnannotatedRegion |
| 958 | CATCGCCTTG.CAATCGGCGC | A | x | 35003_403_2011 | UnannotatedRegion |
| 958 | CATCGCCTTG.CAATCGGCGC | A | x | 35004_12_15398_XS2_1 | UnannotatedRegion |
| 958 | CATCGCCTTG.CAATCGGCGC | A | x | 35005_12_13952_XS2_1 | UnannotatedRegion |
| 958 | CATCGCCTTG.CAATCGGCGC | A | x | 35006_12_15317_XS2_1 | UnannotatedRegion |
| 958 | CATCGCCTTG.CAATCGGCGC | A | x | 35007_12_15047_XS2_1 | UnannotatedRegion |
| 958 | CATCGCCTTG.CAATCGGCGC | A | x | 35008_12_15661_XS2_1 | UnannotatedRegion |
| 958 | CATCGCCTTG.CAATCGGCGC | A | x | 35009_12_15657_XS2_1 | UnannotatedRegion |
| 958 | CATCGCCTTG.CAATCGGCGC | A | x | 35010_12_17186_XS2_1 | UnannotatedRegion |
| 958 | CATCGCCTTG.CAATCGGCGC | A | x | 35011_12_17194_XS2_1 | UnannotatedRegion |
| 958 | CATCGCCTTG.CAATCGGCGC | A | x | 35012_12_17009_XS2_1 | UnannotatedRegion |
| 958 | CATCGCCTTG.CAATCGGCGC | A | x | 35013_12_14973_XS2_1 | UnannotatedRegion |
|  |  |  |  |  |  |
| 965 | CATGATTTGT.TGCTTTGTAA | A | 1619810 R | 19260_WUE_2594 | UnannotatedRegion |
| 965 | CATGATTTGT.TGCTTTGTAA | G | x | 34990_Tchad54_11 | UnannotatedRegion |
| 965 | CATGATTTGT.TGCTTTGTAA | G | x | 34991_Tchad78_11 | UnannotatedRegion |
| 965 | CATGATTTGT.TGCTTTGTAA | G | x | 34992_Tchad95_11 | UnannotatedRegion |
| 965 | CATGATTTGT.TGCTTTGTAA | G | x | 34994_Tchad106_11 | UnannotatedRegion |
| 965 | CATGATTTGT.TGCTTTGTAA | G | x | 34995_8_2011 | UnannotatedRegion |
| 965 | CATGATTTGT.TGCTTTGTAA | G | x | 34996_10_2011 | UnannotatedRegion |
| 965 | CATGATTTGT.TGCTTTGTAA | G | x | 34997_39_2011 | UnannotatedRegion |
| 965 | CATGATTTGT.TGCTTTGTAA | G | x | 34998_59_2011 | UnannotatedRegion |
| 965 | CATGATTTGT.TGCTTTGTAA | G | x | 34999_63_2011 | UnannotatedRegion |
| 965 | CATGATTTGT.TGCTTTGTAA | G | x | 35000_75_2011 | UnannotatedRegion |
| 965 | CATGATTTGT.TGCTTTGTAA | G | x | 35001_76_2011 | UnannotatedRegion |
| 965 | CATGATTTGT.TGCTTTGTAA | G | x | 35002_120_2011 | UnannotatedRegion |
| 965 | CATGATTTGT.TGCTTTGTAA | G | x | 35003_403_2011 | UnannotatedRegion |
| 965 | CATGATTTGT.TGCTTTGTAA | G | x | 35004_12_15398_XS2_1 | UnannotatedRegion |
| 965 | CATGATTTGT.TGCTTTGTAA | G | x | 35005_12_13952_XS2_1 | UnannotatedRegion |
| 965 | CATGATTTGT.TGCTTTGTAA | G | x | 35006_12_15317_XS2_1 | UnannotatedRegion |
| 965 | CATGATTTGT.TGCTTTGTAA | G | x | 35007_12_15047_XS2_1 | UnannotatedRegion |
| 965 | CATGATTTGT.TGCTTTGTAA | G | x | 35008_12_15661_XS2_1 | UnannotatedRegion |
| 965 | CATGATTTGT.TGCTTTGTAA | G | x | 35009_12_15657_XS2_1 | UnannotatedRegion |
| 965 | CATGATTTGT.TGCTTTGTAA | G | x | 35010_12_17186_XS2_1 | UnannotatedRegion |
| 965 | CATGATTTGT.TGCTTTGTAA | G | x | 35011_12_17194_XS2_1 | UnannotatedRegion |
| 965 | CATGATTTGT.TGCTTTGTAA | G | x | 35012_12_17009_XS2_1 | UnannotatedRegion |
| 965 | CATGATTTGT.TGCTTTGTAA | G | x | 35013_12_14973_XS2_1 | UnannotatedRegion |
|  |  |  |  |  |  |
| 968 | CATTTATCCT.TCTAAAGCCG | C | 192652 R | 19260_WUE_2594 | UnannotatedRegion |
| 968 | CATTTATCCT.TCTAAAGCCG | T | x | 34990_Tchad54_11 | UnannotatedRegion |
| 968 | CATTTATCCT.TCTAAAGCCG | T | x | 34991_Tchad78_11 | UnannotatedRegion |
| 968 | CATTTATCCT.TCTAAAGCCG | T | x | 34992_Tchad95_11 | UnannotatedRegion |
| 968 | CATTTATCCT.TCTAAAGCCG | T | x | 34994_Tchad106_11 | UnannotatedRegion |
| 968 | CATTTATCCT.TCTAAAGCCG | T | x | 34995_8_2011 | UnannotatedRegion |
| 968 | CATTTATCCT.TCTAAAGCCG | T | x | 34996_10_2011 | UnannotatedRegion |
| 968 | CATTTATCCT.TCTAAAGCCG | T | x | 34997_39_2011 | UnannotatedRegion |
| 968 | CATTTATCCT.TCTAAAGCCG | T | x | 34998_59_2011 | UnannotatedRegion |
| 968 | CATTTATCCT.TCTAAAGCCG | T | x | 34999_63_2011 | UnannotatedRegion |
| 968 | CATTTATCCT.TCTAAAGCCG | T | x | 35000_75_2011 | UnannotatedRegion |
| 968 | CATTTATCCT.TCTAAAGCCG | T | x | 35001_76_2011 | UnannotatedRegion |
| 968 | CATTTATCCT.TCTAAAGCCG | T | x | 35002_120_2011 | UnannotatedRegion |
| 968 | CATTTATCCT.TCTAAAGCCG | T | x | 35003_403_2011 | UnannotatedRegion |
| 968 | CATTTATCCT.TCTAAAGCCG | T | x | 35004_12_15398_XS2_1 | UnannotatedRegion |
| 968 | CATTTATCCT.TCTAAAGCCG | T | x | 35005_12_13952_XS2_1 | UnannotatedRegion |
| 968 | CATTTATCCT.TCTAAAGCCG | T | x | 35006_12_15317_XS2_1 | UnannotatedRegion |
| 968 | CATTTATCCT.TCTAAAGCCG | T | x | 35007_12_15047_XS2_1 | UnannotatedRegion |
| 968 | CATTTATCCT.TCTAAAGCCG | T | x | 35008_12_15661_XS2_1 | UnannotatedRegion |
| 968 | CATTTATCCT.TCTAAAGCCG | T | x | 35009_12_15657_XS2_1 | UnannotatedRegion |
| 968 | CATTTATCCT.TCTAAAGCCG | T | x | 35010_12_17186_XS2_1 | UnannotatedRegion |
| 968 | CATTTATCCT.TCTAAAGCCG | T | x | 35011_12_17194_XS2_1 | UnannotatedRegion |
| 968 | CATTTATCCT.TCTAAAGCCG | T | x | 35012_12_17009_XS2_1 | UnannotatedRegion |
| 968 | CATTTATCCT.TCTAAAGCCG | T | x | 35013_12_14973_XS2_1 | UnannotatedRegion |
|  |  |  |  |  |  |
| 986 | CCAACCGCTT.CTCTCCGATC | C | x | 34990_Tchad54_11 | NotInAnnotatedGenome |
| 986 | CCAACCGCTT.CTCTCCGATC | C | x | 34991_Tchad78_11 | NotInAnnotatedGenome |
| 986 | CCAACCGCTT.CTCTCCGATC | C | x | 34992_Tchad95_11 | NotInAnnotatedGenome |
| 986 | CCAACCGCTT.CTCTCCGATC | C | x | 34994_Tchad106_11 | NotInAnnotatedGenome |
| 986 | CCAACCGCTT.CTCTCCGATC | C | x | 34995_8_2011 | NotInAnnotatedGenome |
| 986 | CCAACCGCTT.CTCTCCGATC | C | x | 34996_10_2011 | NotInAnnotatedGenome |
| 986 | CCAACCGCTT.CTCTCCGATC | C | x | 34997_39_2011 | NotInAnnotatedGenome |
| 986 | CCAACCGCTT.CTCTCCGATC | C | x | 34998_59_2011 | NotInAnnotatedGenome |
| 986 | CCAACCGCTT.CTCTCCGATC | C | x | 34999_63_2011 | NotInAnnotatedGenome |
| 986 | CCAACCGCTT.CTCTCCGATC | C | x | 35000_75_2011 | NotInAnnotatedGenome |
| 986 | CCAACCGCTT.CTCTCCGATC | C | x | 35001_76_2011 | NotInAnnotatedGenome |
| 986 | CCAACCGCTT.CTCTCCGATC | C | x | 35002_120_2011 | NotInAnnotatedGenome |
| 986 | CCAACCGCTT.CTCTCCGATC | C | x | 35003_403_2011 | NotInAnnotatedGenome |
| 986 | CCAACCGCTT.CTCTCCGATC | C | x | 35004_12_15398_XS2_1 | NotInAnnotatedGenome |
| 986 | CCAACCGCTT.CTCTCCGATC | C | x | 35005_12_13952_XS2_1 | NotInAnnotatedGenome |
| 986 | CCAACCGCTT.CTCTCCGATC | C | x | 35006_12_15317_XS2_1 | NotInAnnotatedGenome |
| 986 | CCAACCGCTT.CTCTCCGATC | T | x | 35007_12_15047_XS2_1 | NotInAnnotatedGenome |
| 986 | CCAACCGCTT.CTCTCCGATC | C | x | 35008_12_15661_XS2_1 | NotInAnnotatedGenome |
| 986 | CCAACCGCTT.CTCTCCGATC | C | x | 35009_12_15657_XS2_1 | NotInAnnotatedGenome |
| 986 | CCAACCGCTT.CTCTCCGATC | C | x | 35010_12_17186_XS2_1 | NotInAnnotatedGenome |
| 986 | CCAACCGCTT.CTCTCCGATC | C | x | 35011_12_17194_XS2_1 | NotInAnnotatedGenome |
| 986 | CCAACCGCTT.CTCTCCGATC | C | x | 35012_12_17009_XS2_1 | NotInAnnotatedGenome |
| 986 | CCAACCGCTT.CTCTCCGATC | C | x | 35013_12_14973_XS2_1 | NotInAnnotatedGenome |
|  |  |  |  |  |  |
| 1052 | CCCTTTGTGG.GGCAGGAACA | C | 1533356 R | 19260_WUE_2594 | UnannotatedRegion |
| 1052 | CCCTTTGTGG.GGCAGGAACA | C | x | 34990_Tchad54_11 | UnannotatedRegion |
| 1052 | CCCTTTGTGG.GGCAGGAACA | C | x | 34991_Tchad78_11 | UnannotatedRegion |
| 1052 | CCCTTTGTGG.GGCAGGAACA | C | x | 34992_Tchad95_11 | UnannotatedRegion |
| 1052 | CCCTTTGTGG.GGCAGGAACA | C | x | 34994_Tchad106_11 | UnannotatedRegion |
| 1052 | CCCTTTGTGG.GGCAGGAACA | C | x | 34995_8_2011 | UnannotatedRegion |
| 1052 | CCCTTTGTGG.GGCAGGAACA | C | x | 34996_10_2011 | UnannotatedRegion |
| 1052 | CCCTTTGTGG.GGCAGGAACA | C | x | 34997_39_2011 | UnannotatedRegion |
| 1052 | CCCTTTGTGG.GGCAGGAACA | C | x | 34998_59_2011 | UnannotatedRegion |
| 1052 | CCCTTTGTGG.GGCAGGAACA | C | x | 34999_63_2011 | UnannotatedRegion |
| 1052 | CCCTTTGTGG.GGCAGGAACA | C | x | 35000_75_2011 | UnannotatedRegion |
| 1052 | CCCTTTGTGG.GGCAGGAACA | C | x | 35001_76_2011 | UnannotatedRegion |
| 1052 | CCCTTTGTGG.GGCAGGAACA | C | x | 35002_120_2011 | UnannotatedRegion |
| 1052 | CCCTTTGTGG.GGCAGGAACA | C | x | 35003_403_2011 | UnannotatedRegion |
| 1052 | CCCTTTGTGG.GGCAGGAACA | C | x | 35004_12_15398_XS2_1 | UnannotatedRegion |
| 1052 | CCCTTTGTGG.GGCAGGAACA | C | x | 35005_12_13952_XS2_1 | UnannotatedRegion |
| 1052 | CCCTTTGTGG.GGCAGGAACA | C | x | 35006_12_15317_XS2_1 | UnannotatedRegion |
| 1052 | CCCTTTGTGG.GGCAGGAACA | T | x | 35007_12_15047_XS2_1 | UnannotatedRegion |
| 1052 | CCCTTTGTGG.GGCAGGAACA | T | x | 35008_12_15661_XS2_1 | UnannotatedRegion |
| 1052 | CCCTTTGTGG.GGCAGGAACA | T | x | 35009_12_15657_XS2_1 | UnannotatedRegion |
| 1052 | CCCTTTGTGG.GGCAGGAACA | C | x | 35010_12_17186_XS2_1 | UnannotatedRegion |
| 1052 | CCCTTTGTGG.GGCAGGAACA | C | x | 35011_12_17194_XS2_1 | UnannotatedRegion |
| 1052 | CCCTTTGTGG.GGCAGGAACA | C | x | 35012_12_17009_XS2_1 | UnannotatedRegion |
| 1052 | CCCTTTGTGG.GGCAGGAACA | T | x | 35013_12_14973_XS2_1 | UnannotatedRegion |
|  |  |  |  |  |  |
| 1056 | CCGAACAATC.AGAATAAGGC | A | 1731055 R | 19260_WUE_2594 | UnannotatedRegion |
| 1056 | CCGAACAATC.AGAATAAGGC | A | x | 34990_Tchad54_11 | UnannotatedRegion |
| 1056 | CCGAACAATC.AGAATAAGGC | A | x | 34991_Tchad78_11 | UnannotatedRegion |
| 1056 | CCGAACAATC.AGAATAAGGC | A | x | 34992_Tchad95_11 | UnannotatedRegion |
| 1056 | CCGAACAATC.AGAATAAGGC | A | x | 34994_Tchad106_11 | UnannotatedRegion |
| 1056 | CCGAACAATC.AGAATAAGGC | A | x | 34995_8_2011 | UnannotatedRegion |
| 1056 | CCGAACAATC.AGAATAAGGC | A | x | 34996_10_2011 | UnannotatedRegion |
| 1056 | CCGAACAATC.AGAATAAGGC | A | x | 34997_39_2011 | UnannotatedRegion |
| 1056 | CCGAACAATC.AGAATAAGGC | A | x | 34998_59_2011 | UnannotatedRegion |
| 1056 | CCGAACAATC.AGAATAAGGC | A | x | 34999_63_2011 | UnannotatedRegion |
| 1056 | CCGAACAATC.AGAATAAGGC | A | x | 35000_75_2011 | UnannotatedRegion |
| 1056 | CCGAACAATC.AGAATAAGGC | A | x | 35001_76_2011 | UnannotatedRegion |
| 1056 | CCGAACAATC.AGAATAAGGC | A | x | 35002_120_2011 | UnannotatedRegion |
| 1056 | CCGAACAATC.AGAATAAGGC | A | x | 35003_403_2011 | UnannotatedRegion |
| 1056 | CCGAACAATC.AGAATAAGGC | A | x | 35004_12_15398_XS2_1 | UnannotatedRegion |
| 1056 | CCGAACAATC.AGAATAAGGC | A | x | 35005_12_13952_XS2_1 | UnannotatedRegion |
| 1056 | CCGAACAATC.AGAATAAGGC | A | x | 35006_12_15317_XS2_1 | UnannotatedRegion |
| 1056 | CCGAACAATC.AGAATAAGGC | G | x | 35007_12_15047_XS2_1 | UnannotatedRegion |
| 1056 | CCGAACAATC.AGAATAAGGC | A | x | 35008_12_15661_XS2_1 | UnannotatedRegion |
| 1056 | CCGAACAATC.AGAATAAGGC | A | x | 35009_12_15657_XS2_1 | UnannotatedRegion |
| 1056 | CCGAACAATC.AGAATAAGGC | A | x | 35010_12_17186_XS2_1 | UnannotatedRegion |
| 1056 | CCGAACAATC.AGAATAAGGC | A | x | 35011_12_17194_XS2_1 | UnannotatedRegion |
| 1056 | CCGAACAATC.AGAATAAGGC | A | x | 35012_12_17009_XS2_1 | UnannotatedRegion |
| 1056 | CCGAACAATC.AGAATAAGGC | A | x | 35013_12_14973_XS2_1 | UnannotatedRegion |
|  |  |  |  |  |  |
| 1078 | CCGCAAAATC.CGACACACTC | A | 1270184 F | 19260_WUE_2594 | UnannotatedRegion |
| 1078 | CCGCAAAATC.CGACACACTC | C | x | 34990_Tchad54_11 | UnannotatedRegion |
| 1078 | CCGCAAAATC.CGACACACTC | C | x | 34991_Tchad78_11 | UnannotatedRegion |
| 1078 | CCGCAAAATC.CGACACACTC | C | x | 34992_Tchad95_11 | UnannotatedRegion |
| 1078 | CCGCAAAATC.CGACACACTC | C | x | 34994_Tchad106_11 | UnannotatedRegion |
| 1078 | CCGCAAAATC.CGACACACTC | C | x | 34995_8_2011 | UnannotatedRegion |
| 1078 | CCGCAAAATC.CGACACACTC | C | x | 34996_10_2011 | UnannotatedRegion |
| 1078 | CCGCAAAATC.CGACACACTC | C | x | 34997_39_2011 | UnannotatedRegion |
| 1078 | CCGCAAAATC.CGACACACTC | C | x | 34998_59_2011 | UnannotatedRegion |
| 1078 | CCGCAAAATC.CGACACACTC | C | x | 34999_63_2011 | UnannotatedRegion |
| 1078 | CCGCAAAATC.CGACACACTC | C | x | 35000_75_2011 | UnannotatedRegion |
| 1078 | CCGCAAAATC.CGACACACTC | C | x | 35001_76_2011 | UnannotatedRegion |
| 1078 | CCGCAAAATC.CGACACACTC | C | x | 35002_120_2011 | UnannotatedRegion |
| 1078 | CCGCAAAATC.CGACACACTC | C | x | 35003_403_2011 | UnannotatedRegion |
| 1078 | CCGCAAAATC.CGACACACTC | C | x | 35004_12_15398_XS2_1 | UnannotatedRegion |
| 1078 | CCGCAAAATC.CGACACACTC | C | x | 35005_12_13952_XS2_1 | UnannotatedRegion |
| 1078 | CCGCAAAATC.CGACACACTC | C | x | 35006_12_15317_XS2_1 | UnannotatedRegion |
| 1078 | CCGCAAAATC.CGACACACTC | C | x | 35007_12_15047_XS2_1 | UnannotatedRegion |
| 1078 | CCGCAAAATC.CGACACACTC | C | x | 35008_12_15661_XS2_1 | UnannotatedRegion |
| 1078 | CCGCAAAATC.CGACACACTC | C | x | 35009_12_15657_XS2_1 | UnannotatedRegion |
| 1078 | CCGCAAAATC.CGACACACTC | C | x | 35010_12_17186_XS2_1 | UnannotatedRegion |
| 1078 | CCGCAAAATC.CGACACACTC | C | x | 35011_12_17194_XS2_1 | UnannotatedRegion |
| 1078 | CCGCAAAATC.CGACACACTC | C | x | 35012_12_17009_XS2_1 | UnannotatedRegion |
| 1078 | CCGCAAAATC.CGACACACTC | C | x | 35013_12_14973_XS2_1 | UnannotatedRegion |
|  |  |  |  |  |  |
| 1119 | CCGTCTGAAC.TCTGTCTGCG | G | 1139601 F | 19260_WUE_2594 | UnannotatedRegion |
| 1119 | CCGTCTGAAC.TCTGTCTGCG | A | x | 34990_Tchad54_11 | UnannotatedRegion |
| 1119 | CCGTCTGAAC.TCTGTCTGCG | A | x | 34991_Tchad78_11 | UnannotatedRegion |
| 1119 | CCGTCTGAAC.TCTGTCTGCG | A | x | 34992_Tchad95_11 | UnannotatedRegion |
| 1119 | CCGTCTGAAC.TCTGTCTGCG | A | x | 34994_Tchad106_11 | UnannotatedRegion |
| 1119 | CCGTCTGAAC.TCTGTCTGCG | A | x | 34995_8_2011 | UnannotatedRegion |
| 1119 | CCGTCTGAAC.TCTGTCTGCG | A | x | 34996_10_2011 | UnannotatedRegion |
| 1119 | CCGTCTGAAC.TCTGTCTGCG | A | x | 34997_39_2011 | UnannotatedRegion |
| 1119 | CCGTCTGAAC.TCTGTCTGCG | A | x | 34998_59_2011 | UnannotatedRegion |
| 1119 | CCGTCTGAAC.TCTGTCTGCG | A | x | 34999_63_2011 | UnannotatedRegion |
| 1119 | CCGTCTGAAC.TCTGTCTGCG | A | x | 35000_75_2011 | UnannotatedRegion |
| 1119 | CCGTCTGAAC.TCTGTCTGCG | A | x | 35001_76_2011 | UnannotatedRegion |
| 1119 | CCGTCTGAAC.TCTGTCTGCG | A | x | 35002_120_2011 | UnannotatedRegion |
| 1119 | CCGTCTGAAC.TCTGTCTGCG | A | x | 35003_403_2011 | UnannotatedRegion |
| 1119 | CCGTCTGAAC.TCTGTCTGCG | A | x | 35004_12_15398_XS2_1 | UnannotatedRegion |
| 1119 | CCGTCTGAAC.TCTGTCTGCG | A | x | 35005_12_13952_XS2_1 | UnannotatedRegion |
| 1119 | CCGTCTGAAC.TCTGTCTGCG | A | x | 35006_12_15317_XS2_1 | UnannotatedRegion |
| 1119 | CCGTCTGAAC.TCTGTCTGCG | A | x | 35007_12_15047_XS2_1 | UnannotatedRegion |
| 1119 | CCGTCTGAAC.TCTGTCTGCG | A | x | 35008_12_15661_XS2_1 | UnannotatedRegion |
| 1119 | CCGTCTGAAC.TCTGTCTGCG | A | x | 35009_12_15657_XS2_1 | UnannotatedRegion |
| 1119 | CCGTCTGAAC.TCTGTCTGCG | A | x | 35010_12_17186_XS2_1 | UnannotatedRegion |
| 1119 | CCGTCTGAAC.TCTGTCTGCG | A | x | 35011_12_17194_XS2_1 | UnannotatedRegion |
| 1119 | CCGTCTGAAC.TCTGTCTGCG | A | x | 35012_12_17009_XS2_1 | UnannotatedRegion |
| 1119 | CCGTCTGAAC.TCTGTCTGCG | A | x | 35013_12_14973_XS2_1 | UnannotatedRegion |
|  |  |  |  |  |  |
| 1142 | CCTGCCGGTC.TAAGAGAGGC | A | x | 34990_Tchad54_11 | NotInAnnotatedGenome |
| 1142 | CCTGCCGGTC.TAAGAGAGGC | A | x | 34991_Tchad78_11 | NotInAnnotatedGenome |
| 1142 | CCTGCCGGTC.TAAGAGAGGC | A | x | 34992_Tchad95_11 | NotInAnnotatedGenome |
| 1142 | CCTGCCGGTC.TAAGAGAGGC | A | x | 34994_Tchad106_11 | NotInAnnotatedGenome |
| 1142 | CCTGCCGGTC.TAAGAGAGGC | A | x | 34995_8_2011 | NotInAnnotatedGenome |
| 1142 | CCTGCCGGTC.TAAGAGAGGC | A | x | 34996_10_2011 | NotInAnnotatedGenome |
| 1142 | CCTGCCGGTC.TAAGAGAGGC | A | x | 34997_39_2011 | NotInAnnotatedGenome |
| 1142 | CCTGCCGGTC.TAAGAGAGGC | A | x | 34998_59_2011 | NotInAnnotatedGenome |
| 1142 | CCTGCCGGTC.TAAGAGAGGC | A | x | 34999_63_2011 | NotInAnnotatedGenome |
| 1142 | CCTGCCGGTC.TAAGAGAGGC | A | x | 35000_75_2011 | NotInAnnotatedGenome |
| 1142 | CCTGCCGGTC.TAAGAGAGGC | A | x | 35001_76_2011 | NotInAnnotatedGenome |
| 1142 | CCTGCCGGTC.TAAGAGAGGC | A | x | 35002_120_2011 | NotInAnnotatedGenome |
| 1142 | CCTGCCGGTC.TAAGAGAGGC | A | x | 35003_403_2011 | NotInAnnotatedGenome |
| 1142 | CCTGCCGGTC.TAAGAGAGGC | A | x | 35004_12_15398_XS2_1 | NotInAnnotatedGenome |
| 1142 | CCTGCCGGTC.TAAGAGAGGC | A | x | 35005_12_13952_XS2_1 | NotInAnnotatedGenome |
| 1142 | CCTGCCGGTC.TAAGAGAGGC | A | x | 35006_12_15317_XS2_1 | NotInAnnotatedGenome |
| 1142 | CCTGCCGGTC.TAAGAGAGGC | G | x | 35007_12_15047_XS2_1 | NotInAnnotatedGenome |
| 1142 | CCTGCCGGTC.TAAGAGAGGC | A | x | 35008_12_15661_XS2_1 | NotInAnnotatedGenome |
| 1142 | CCTGCCGGTC.TAAGAGAGGC | A | x | 35009_12_15657_XS2_1 | NotInAnnotatedGenome |
| 1142 | CCTGCCGGTC.TAAGAGAGGC | A | x | 35010_12_17186_XS2_1 | NotInAnnotatedGenome |
| 1142 | CCTGCCGGTC.TAAGAGAGGC | A | x | 35011_12_17194_XS2_1 | NotInAnnotatedGenome |
| 1142 | CCTGCCGGTC.TAAGAGAGGC | A | x | 35012_12_17009_XS2_1 | NotInAnnotatedGenome |
| 1142 | CCTGCCGGTC.TAAGAGAGGC | A | x | 35013_12_14973_XS2_1 | NotInAnnotatedGenome |
|  |  |  |  |  |  |
| 1153 | CCTTTTATTC.ATACACCCGA | A | 507224 R | 19260_WUE_2594 | UnannotatedRegion |
| 1153 | CCTTTTATTC.ATACACCCGA | G | x | 34990_Tchad54_11 | UnannotatedRegion |
| 1153 | CCTTTTATTC.ATACACCCGA | G | x | 34991_Tchad78_11 | UnannotatedRegion |
| 1153 | CCTTTTATTC.ATACACCCGA | G | x | 34992_Tchad95_11 | UnannotatedRegion |
| 1153 | CCTTTTATTC.ATACACCCGA | G | x | 34994_Tchad106_11 | UnannotatedRegion |
| 1153 | CCTTTTATTC.ATACACCCGA | G | x | 34995_8_2011 | UnannotatedRegion |
| 1153 | CCTTTTATTC.ATACACCCGA | G | x | 34996_10_2011 | UnannotatedRegion |
| 1153 | CCTTTTATTC.ATACACCCGA | G | x | 34997_39_2011 | UnannotatedRegion |
| 1153 | CCTTTTATTC.ATACACCCGA | G | x | 34998_59_2011 | UnannotatedRegion |
| 1153 | CCTTTTATTC.ATACACCCGA | G | x | 34999_63_2011 | UnannotatedRegion |
| 1153 | CCTTTTATTC.ATACACCCGA | G | x | 35000_75_2011 | UnannotatedRegion |
| 1153 | CCTTTTATTC.ATACACCCGA | G | x | 35001_76_2011 | UnannotatedRegion |
| 1153 | CCTTTTATTC.ATACACCCGA | G | x | 35002_120_2011 | UnannotatedRegion |
| 1153 | CCTTTTATTC.ATACACCCGA | G | x | 35003_403_2011 | UnannotatedRegion |
| 1153 | CCTTTTATTC.ATACACCCGA | G | x | 35004_12_15398_XS2_1 | UnannotatedRegion |
| 1153 | CCTTTTATTC.ATACACCCGA | G | x | 35005_12_13952_XS2_1 | UnannotatedRegion |
| 1153 | CCTTTTATTC.ATACACCCGA | G | x | 35006_12_15317_XS2_1 | UnannotatedRegion |
| 1153 | CCTTTTATTC.ATACACCCGA | G | x | 35007_12_15047_XS2_1 | UnannotatedRegion |
| 1153 | CCTTTTATTC.ATACACCCGA | G | x | 35008_12_15661_XS2_1 | UnannotatedRegion |
| 1153 | CCTTTTATTC.ATACACCCGA | G | x | 35009_12_15657_XS2_1 | UnannotatedRegion |
| 1153 | CCTTTTATTC.ATACACCCGA | G | x | 35010_12_17186_XS2_1 | UnannotatedRegion |
| 1153 | CCTTTTATTC.ATACACCCGA | G | x | 35011_12_17194_XS2_1 | UnannotatedRegion |
| 1153 | CCTTTTATTC.ATACACCCGA | G | x | 35012_12_17009_XS2_1 | UnannotatedRegion |
| 1153 | CCTTTTATTC.ATACACCCGA | G | x | 35013_12_14973_XS2_1 | UnannotatedRegion |
|  |  |  |  |  |  |
| 1155 | CGAAAATCAA.AACCGATTGC | G | 2034458 F | 19260_WUE_2594 | UnannotatedRegion |
| 1155 | CGAAAATCAA.AACCGATTGC | A | x | 34990_Tchad54_11 | UnannotatedRegion |
| 1155 | CGAAAATCAA.AACCGATTGC | A | x | 34991_Tchad78_11 | UnannotatedRegion |
| 1155 | CGAAAATCAA.AACCGATTGC | A | x | 34992_Tchad95_11 | UnannotatedRegion |
| 1155 | CGAAAATCAA.AACCGATTGC | A | x | 34994_Tchad106_11 | UnannotatedRegion |
| 1155 | CGAAAATCAA.AACCGATTGC | A | x | 34995_8_2011 | UnannotatedRegion |
| 1155 | CGAAAATCAA.AACCGATTGC | A | x | 34996_10_2011 | UnannotatedRegion |
| 1155 | CGAAAATCAA.AACCGATTGC | A | x | 34997_39_2011 | UnannotatedRegion |
| 1155 | CGAAAATCAA.AACCGATTGC | A | x | 34998_59_2011 | UnannotatedRegion |
| 1155 | CGAAAATCAA.AACCGATTGC | A | x | 34999_63_2011 | UnannotatedRegion |
| 1155 | CGAAAATCAA.AACCGATTGC | A | x | 35000_75_2011 | UnannotatedRegion |
| 1155 | CGAAAATCAA.AACCGATTGC | A | x | 35001_76_2011 | UnannotatedRegion |
| 1155 | CGAAAATCAA.AACCGATTGC | A | x | 35002_120_2011 | UnannotatedRegion |
| 1155 | CGAAAATCAA.AACCGATTGC | A | x | 35003_403_2011 | UnannotatedRegion |
| 1155 | CGAAAATCAA.AACCGATTGC | A | x | 35004_12_15398_XS2_1 | UnannotatedRegion |
| 1155 | CGAAAATCAA.AACCGATTGC | A | x | 35005_12_13952_XS2_1 | UnannotatedRegion |
| 1155 | CGAAAATCAA.AACCGATTGC | A | x | 35006_12_15317_XS2_1 | UnannotatedRegion |
| 1155 | CGAAAATCAA.AACCGATTGC | A | x | 35007_12_15047_XS2_1 | UnannotatedRegion |
| 1155 | CGAAAATCAA.AACCGATTGC | A | x | 35008_12_15661_XS2_1 | UnannotatedRegion |
| 1155 | CGAAAATCAA.AACCGATTGC | A | x | 35009_12_15657_XS2_1 | UnannotatedRegion |
| 1155 | CGAAAATCAA.AACCGATTGC | A | x | 35010_12_17186_XS2_1 | UnannotatedRegion |
| 1155 | CGAAAATCAA.AACCGATTGC | A | x | 35011_12_17194_XS2_1 | UnannotatedRegion |
| 1155 | CGAAAATCAA.AACCGATTGC | A | x | 35012_12_17009_XS2_1 | UnannotatedRegion |
| 1155 | CGAAAATCAA.AACCGATTGC | A | x | 35013_12_14973_XS2_1 | UnannotatedRegion |
|  |  |  |  |  |  |
| 1161 | CGAACAAGCC.AAAACCTGCG | G | x | 34990_Tchad54_11 | NotInAnnotatedGenome |
| 1161 | CGAACAAGCC.AAAACCTGCG | G | x | 34991_Tchad78_11 | NotInAnnotatedGenome |
| 1161 | CGAACAAGCC.AAAACCTGCG | G | x | 34992_Tchad95_11 | NotInAnnotatedGenome |
| 1161 | CGAACAAGCC.AAAACCTGCG | G | x | 34994_Tchad106_11 | NotInAnnotatedGenome |
| 1161 | CGAACAAGCC.AAAACCTGCG | A | x | 34995_8_2011 | NotInAnnotatedGenome |
| 1161 | CGAACAAGCC.AAAACCTGCG | A | x | 34996_10_2011 | NotInAnnotatedGenome |
| 1161 | CGAACAAGCC.AAAACCTGCG | G | x | 34997_39_2011 | NotInAnnotatedGenome |
| 1161 | CGAACAAGCC.AAAACCTGCG | G | x | 34998_59_2011 | NotInAnnotatedGenome |
| 1161 | CGAACAAGCC.AAAACCTGCG | G | x | 34999_63_2011 | NotInAnnotatedGenome |
| 1161 | CGAACAAGCC.AAAACCTGCG | G | x | 35000_75_2011 | NotInAnnotatedGenome |
| 1161 | CGAACAAGCC.AAAACCTGCG | G | x | 35001_76_2011 | NotInAnnotatedGenome |
| 1161 | CGAACAAGCC.AAAACCTGCG | G | x | 35002_120_2011 | NotInAnnotatedGenome |
| 1161 | CGAACAAGCC.AAAACCTGCG | G | x | 35003_403_2011 | NotInAnnotatedGenome |
| 1161 | CGAACAAGCC.AAAACCTGCG | G | x | 35004_12_15398_XS2_1 | NotInAnnotatedGenome |
| 1161 | CGAACAAGCC.AAAACCTGCG | G | x | 35005_12_13952_XS2_1 | NotInAnnotatedGenome |
| 1161 | CGAACAAGCC.AAAACCTGCG | G | x | 35006_12_15317_XS2_1 | NotInAnnotatedGenome |
| 1161 | CGAACAAGCC.AAAACCTGCG | G | x | 35007_12_15047_XS2_1 | NotInAnnotatedGenome |
| 1161 | CGAACAAGCC.AAAACCTGCG | G | x | 35008_12_15661_XS2_1 | NotInAnnotatedGenome |
| 1161 | CGAACAAGCC.AAAACCTGCG | G | x | 35009_12_15657_XS2_1 | NotInAnnotatedGenome |
| 1161 | CGAACAAGCC.AAAACCTGCG | G | x | 35010_12_17186_XS2_1 | NotInAnnotatedGenome |
| 1161 | CGAACAAGCC.AAAACCTGCG | G | x | 35011_12_17194_XS2_1 | NotInAnnotatedGenome |
| 1161 | CGAACAAGCC.AAAACCTGCG | G | x | 35012_12_17009_XS2_1 | NotInAnnotatedGenome |
| 1161 | CGAACAAGCC.AAAACCTGCG | G | x | 35013_12_14973_XS2_1 | NotInAnnotatedGenome |
|  |  |  |  |  |  |
| 1188 | CGATAAAATA.CCGATTTAAC | T | 255491 F | 19260_WUE_2594 | UnannotatedRegion |
| 1188 | CGATAAAATA.CCGATTTAAC | C | x | 34990_Tchad54_11 | UnannotatedRegion |
| 1188 | CGATAAAATA.CCGATTTAAC | C | x | 34991_Tchad78_11 | UnannotatedRegion |
| 1188 | CGATAAAATA.CCGATTTAAC | C | x | 34992_Tchad95_11 | UnannotatedRegion |
| 1188 | CGATAAAATA.CCGATTTAAC | C | x | 34994_Tchad106_11 | UnannotatedRegion |
| 1188 | CGATAAAATA.CCGATTTAAC | C | x | 34995_8_2011 | UnannotatedRegion |
| 1188 | CGATAAAATA.CCGATTTAAC | C | x | 34996_10_2011 | UnannotatedRegion |
| 1188 | CGATAAAATA.CCGATTTAAC | C | x | 34997_39_2011 | UnannotatedRegion |
| 1188 | CGATAAAATA.CCGATTTAAC | C | x | 34998_59_2011 | UnannotatedRegion |
| 1188 | CGATAAAATA.CCGATTTAAC | C | x | 34999_63_2011 | UnannotatedRegion |
| 1188 | CGATAAAATA.CCGATTTAAC | C | x | 35000_75_2011 | UnannotatedRegion |
| 1188 | CGATAAAATA.CCGATTTAAC | C | x | 35001_76_2011 | UnannotatedRegion |
| 1188 | CGATAAAATA.CCGATTTAAC | C | x | 35002_120_2011 | UnannotatedRegion |
| 1188 | CGATAAAATA.CCGATTTAAC | C | x | 35003_403_2011 | UnannotatedRegion |
| 1188 | CGATAAAATA.CCGATTTAAC | C | x | 35004_12_15398_XS2_1 | UnannotatedRegion |
| 1188 | CGATAAAATA.CCGATTTAAC | C | x | 35005_12_13952_XS2_1 | UnannotatedRegion |
| 1188 | CGATAAAATA.CCGATTTAAC | C | x | 35006_12_15317_XS2_1 | UnannotatedRegion |
| 1188 | CGATAAAATA.CCGATTTAAC | C | x | 35007_12_15047_XS2_1 | UnannotatedRegion |
| 1188 | CGATAAAATA.CCGATTTAAC | C | x | 35008_12_15661_XS2_1 | UnannotatedRegion |
| 1188 | CGATAAAATA.CCGATTTAAC | C | x | 35009_12_15657_XS2_1 | UnannotatedRegion |
| 1188 | CGATAAAATA.CCGATTTAAC | C | x | 35010_12_17186_XS2_1 | UnannotatedRegion |
| 1188 | CGATAAAATA.CCGATTTAAC | C | x | 35011_12_17194_XS2_1 | UnannotatedRegion |
| 1188 | CGATAAAATA.CCGATTTAAC | C | x | 35012_12_17009_XS2_1 | UnannotatedRegion |
| 1188 | CGATAAAATA.CCGATTTAAC | C | x | 35013_12_14973_XS2_1 | UnannotatedRegion |
|  |  |  |  |  |  |
| 1232 | CGCCATATCC.AACCTGACCG | G | 1147124 F | 19260_WUE_2594 | UnannotatedRegion |
| 1232 | CGCCATATCC.AACCTGACCG | A | x | 34990_Tchad54_11 | UnannotatedRegion |
| 1232 | CGCCATATCC.AACCTGACCG | A | x | 34991_Tchad78_11 | UnannotatedRegion |
| 1232 | CGCCATATCC.AACCTGACCG | A | x | 34992_Tchad95_11 | UnannotatedRegion |
| 1232 | CGCCATATCC.AACCTGACCG | A | x | 34994_Tchad106_11 | UnannotatedRegion |
| 1232 | CGCCATATCC.AACCTGACCG | A | x | 34995_8_2011 | UnannotatedRegion |
| 1232 | CGCCATATCC.AACCTGACCG | A | x | 34996_10_2011 | UnannotatedRegion |
| 1232 | CGCCATATCC.AACCTGACCG | A | x | 34997_39_2011 | UnannotatedRegion |
| 1232 | CGCCATATCC.AACCTGACCG | A | x | 34998_59_2011 | UnannotatedRegion |
| 1232 | CGCCATATCC.AACCTGACCG | A | x | 34999_63_2011 | UnannotatedRegion |
| 1232 | CGCCATATCC.AACCTGACCG | A | x | 35000_75_2011 | UnannotatedRegion |
| 1232 | CGCCATATCC.AACCTGACCG | A | x | 35001_76_2011 | UnannotatedRegion |
| 1232 | CGCCATATCC.AACCTGACCG | A | x | 35002_120_2011 | UnannotatedRegion |
| 1232 | CGCCATATCC.AACCTGACCG | A | x | 35003_403_2011 | UnannotatedRegion |
| 1232 | CGCCATATCC.AACCTGACCG | A | x | 35004_12_15398_XS2_1 | UnannotatedRegion |
| 1232 | CGCCATATCC.AACCTGACCG | A | x | 35005_12_13952_XS2_1 | UnannotatedRegion |
| 1232 | CGCCATATCC.AACCTGACCG | A | x | 35006_12_15317_XS2_1 | UnannotatedRegion |
| 1232 | CGCCATATCC.AACCTGACCG | A | x | 35007_12_15047_XS2_1 | UnannotatedRegion |
| 1232 | CGCCATATCC.AACCTGACCG | A | x | 35008_12_15661_XS2_1 | UnannotatedRegion |
| 1232 | CGCCATATCC.AACCTGACCG | A | x | 35009_12_15657_XS2_1 | UnannotatedRegion |
| 1232 | CGCCATATCC.AACCTGACCG | A | x | 35010_12_17186_XS2_1 | UnannotatedRegion |
| 1232 | CGCCATATCC.AACCTGACCG | A | x | 35011_12_17194_XS2_1 | UnannotatedRegion |
| 1232 | CGCCATATCC.AACCTGACCG | A | x | 35012_12_17009_XS2_1 | UnannotatedRegion |
| 1232 | CGCCATATCC.AACCTGACCG | A | x | 35013_12_14973_XS2_1 | UnannotatedRegion |
|  |  |  |  |  |  |
| 1239 | CGCCCGCTGA.CCTTTCAGAC | C | 2080463 F | 19260_WUE_2594 | UnannotatedRegion |
| 1239 | CGCCCGCTGA.CCTTTCAGAC | C | x | 34990_Tchad54_11 | UnannotatedRegion |
| 1239 | CGCCCGCTGA.CCTTTCAGAC | C | x | 34991_Tchad78_11 | UnannotatedRegion |
| 1239 | CGCCCGCTGA.CCTTTCAGAC | C | x | 34992_Tchad95_11 | UnannotatedRegion |
| 1239 | CGCCCGCTGA.CCTTTCAGAC | C | x | 34994_Tchad106_11 | UnannotatedRegion |
| 1239 | CGCCCGCTGA.CCTTTCAGAC | T | x | 34995_8_2011 | UnannotatedRegion |
| 1239 | CGCCCGCTGA.CCTTTCAGAC | T | x | 34996_10_2011 | UnannotatedRegion |
| 1239 | CGCCCGCTGA.CCTTTCAGAC | C | x | 34997_39_2011 | UnannotatedRegion |
| 1239 | CGCCCGCTGA.CCTTTCAGAC | C | x | 34998_59_2011 | UnannotatedRegion |
| 1239 | CGCCCGCTGA.CCTTTCAGAC | C | x | 34999_63_2011 | UnannotatedRegion |
| 1239 | CGCCCGCTGA.CCTTTCAGAC | C | x | 35000_75_2011 | UnannotatedRegion |
| 1239 | CGCCCGCTGA.CCTTTCAGAC | C | x | 35001_76_2011 | UnannotatedRegion |
| 1239 | CGCCCGCTGA.CCTTTCAGAC | C | x | 35002_120_2011 | UnannotatedRegion |
| 1239 | CGCCCGCTGA.CCTTTCAGAC | C | x | 35003_403_2011 | UnannotatedRegion |
| 1239 | CGCCCGCTGA.CCTTTCAGAC | C | x | 35004_12_15398_XS2_1 | UnannotatedRegion |
| 1239 | CGCCCGCTGA.CCTTTCAGAC | C | x | 35005_12_13952_XS2_1 | UnannotatedRegion |
| 1239 | CGCCCGCTGA.CCTTTCAGAC | C | x | 35006_12_15317_XS2_1 | UnannotatedRegion |
| 1239 | CGCCCGCTGA.CCTTTCAGAC | C | x | 35007_12_15047_XS2_1 | UnannotatedRegion |
| 1239 | CGCCCGCTGA.CCTTTCAGAC | C | x | 35008_12_15661_XS2_1 | UnannotatedRegion |
| 1239 | CGCCCGCTGA.CCTTTCAGAC | C | x | 35009_12_15657_XS2_1 | UnannotatedRegion |
| 1239 | CGCCCGCTGA.CCTTTCAGAC | C | x | 35010_12_17186_XS2_1 | UnannotatedRegion |
| 1239 | CGCCCGCTGA.CCTTTCAGAC | C | x | 35011_12_17194_XS2_1 | UnannotatedRegion |
| 1239 | CGCCCGCTGA.CCTTTCAGAC | C | x | 35012_12_17009_XS2_1 | UnannotatedRegion |
| 1239 | CGCCCGCTGA.CCTTTCAGAC | C | x | 35013_12_14973_XS2_1 | UnannotatedRegion |
|  |  |  |  |  |  |
| 1240 | CGCCCGGACT.AATCGGTTTA | G | 739919 R | 19260_WUE_2594 | UnannotatedRegion |
| 1240 | CGCCCGGACT.AATCGGTTTA | A | x | 34990_Tchad54_11 | UnannotatedRegion |
| 1240 | CGCCCGGACT.AATCGGTTTA | A | x | 34991_Tchad78_11 | UnannotatedRegion |
| 1240 | CGCCCGGACT.AATCGGTTTA | A | x | 34992_Tchad95_11 | UnannotatedRegion |
| 1240 | CGCCCGGACT.AATCGGTTTA | A | x | 34994_Tchad106_11 | UnannotatedRegion |
| 1240 | CGCCCGGACT.AATCGGTTTA | A | x | 34995_8_2011 | UnannotatedRegion |
| 1240 | CGCCCGGACT.AATCGGTTTA | A | x | 34996_10_2011 | UnannotatedRegion |
| 1240 | CGCCCGGACT.AATCGGTTTA | A | x | 34997_39_2011 | UnannotatedRegion |
| 1240 | CGCCCGGACT.AATCGGTTTA | A | x | 34998_59_2011 | UnannotatedRegion |
| 1240 | CGCCCGGACT.AATCGGTTTA | A | x | 34999_63_2011 | UnannotatedRegion |
| 1240 | CGCCCGGACT.AATCGGTTTA | A | x | 35000_75_2011 | UnannotatedRegion |
| 1240 | CGCCCGGACT.AATCGGTTTA | A | x | 35001_76_2011 | UnannotatedRegion |
| 1240 | CGCCCGGACT.AATCGGTTTA | A | x | 35002_120_2011 | UnannotatedRegion |
| 1240 | CGCCCGGACT.AATCGGTTTA | A | x | 35003_403_2011 | UnannotatedRegion |
| 1240 | CGCCCGGACT.AATCGGTTTA | A | x | 35004_12_15398_XS2_1 | UnannotatedRegion |
| 1240 | CGCCCGGACT.AATCGGTTTA | A | x | 35005_12_13952_XS2_1 | UnannotatedRegion |
| 1240 | CGCCCGGACT.AATCGGTTTA | A | x | 35006_12_15317_XS2_1 | UnannotatedRegion |
| 1240 | CGCCCGGACT.AATCGGTTTA | A | x | 35007_12_15047_XS2_1 | UnannotatedRegion |
| 1240 | CGCCCGGACT.AATCGGTTTA | A | x | 35008_12_15661_XS2_1 | UnannotatedRegion |
| 1240 | CGCCCGGACT.AATCGGTTTA | A | x | 35009_12_15657_XS2_1 | UnannotatedRegion |
| 1240 | CGCCCGGACT.AATCGGTTTA | A | x | 35010_12_17186_XS2_1 | UnannotatedRegion |
| 1240 | CGCCCGGACT.AATCGGTTTA | A | x | 35011_12_17194_XS2_1 | UnannotatedRegion |
| 1240 | CGCCCGGACT.AATCGGTTTA | A | x | 35012_12_17009_XS2_1 | UnannotatedRegion |
| 1240 | CGCCCGGACT.AATCGGTTTA | A | x | 35013_12_14973_XS2_1 | UnannotatedRegion |
|  |  |  |  |  |  |
| 1242 | CGCCCGTGCC.GCGATGTACC | G | x | 34990_Tchad54_11 | NotInAnnotatedGenome |
| 1242 | CGCCCGTGCC.GCGATGTACC | G | x | 34991_Tchad78_11 | NotInAnnotatedGenome |
| 1242 | CGCCCGTGCC.GCGATGTACC | G | x | 34992_Tchad95_11 | NotInAnnotatedGenome |
| 1242 | CGCCCGTGCC.GCGATGTACC | G | x | 34994_Tchad106_11 | NotInAnnotatedGenome |
| 1242 | CGCCCGTGCC.GCGATGTACC | G | x | 34995_8_2011 | NotInAnnotatedGenome |
| 1242 | CGCCCGTGCC.GCGATGTACC | G | x | 34996_10_2011 | NotInAnnotatedGenome |
| 1242 | CGCCCGTGCC.GCGATGTACC | G | x | 34997_39_2011 | NotInAnnotatedGenome |
| 1242 | CGCCCGTGCC.GCGATGTACC | A | x | 34998_59_2011 | NotInAnnotatedGenome |
| 1242 | CGCCCGTGCC.GCGATGTACC | G | x | 34999_63_2011 | NotInAnnotatedGenome |
| 1242 | CGCCCGTGCC.GCGATGTACC | G | x | 35000_75_2011 | NotInAnnotatedGenome |
| 1242 | CGCCCGTGCC.GCGATGTACC | G | x | 35001_76_2011 | NotInAnnotatedGenome |
| 1242 | CGCCCGTGCC.GCGATGTACC | G | x | 35002_120_2011 | NotInAnnotatedGenome |
| 1242 | CGCCCGTGCC.GCGATGTACC | G | x | 35003_403_2011 | NotInAnnotatedGenome |
| 1242 | CGCCCGTGCC.GCGATGTACC | G | x | 35004_12_15398_XS2_1 | NotInAnnotatedGenome |
| 1242 | CGCCCGTGCC.GCGATGTACC | G | x | 35005_12_13952_XS2_1 | NotInAnnotatedGenome |
| 1242 | CGCCCGTGCC.GCGATGTACC | G | x | 35006_12_15317_XS2_1 | NotInAnnotatedGenome |
| 1242 | CGCCCGTGCC.GCGATGTACC | G | x | 35007_12_15047_XS2_1 | NotInAnnotatedGenome |
| 1242 | CGCCCGTGCC.GCGATGTACC | G | x | 35008_12_15661_XS2_1 | NotInAnnotatedGenome |
| 1242 | CGCCCGTGCC.GCGATGTACC | G | x | 35009_12_15657_XS2_1 | NotInAnnotatedGenome |
| 1242 | CGCCCGTGCC.GCGATGTACC | G | x | 35010_12_17186_XS2_1 | NotInAnnotatedGenome |
| 1242 | CGCCCGTGCC.GCGATGTACC | G | x | 35011_12_17194_XS2_1 | NotInAnnotatedGenome |
| 1242 | CGCCCGTGCC.GCGATGTACC | G | x | 35012_12_17009_XS2_1 | NotInAnnotatedGenome |
| 1242 | CGCCCGTGCC.GCGATGTACC | G | x | 35013_12_14973_XS2_1 | NotInAnnotatedGenome |
|  |  |  |  |  |  |
| 1243 | CGCCCGTTTC.CAAAAAACGC | C | 1139577 R | 19260_WUE_2594 | UnannotatedRegion |
| 1243 | CGCCCGTTTC.CAAAAAACGC | A | x | 34990_Tchad54_11 | UnannotatedRegion |
| 1243 | CGCCCGTTTC.CAAAAAACGC | A | x | 34991_Tchad78_11 | UnannotatedRegion |
| 1243 | CGCCCGTTTC.CAAAAAACGC | A | x | 34992_Tchad95_11 | UnannotatedRegion |
| 1243 | CGCCCGTTTC.CAAAAAACGC | A | x | 34994_Tchad106_11 | UnannotatedRegion |
| 1243 | CGCCCGTTTC.CAAAAAACGC | A | x | 34995_8_2011 | UnannotatedRegion |
| 1243 | CGCCCGTTTC.CAAAAAACGC | A | x | 34996_10_2011 | UnannotatedRegion |
| 1243 | CGCCCGTTTC.CAAAAAACGC | A | x | 34997_39_2011 | UnannotatedRegion |
| 1243 | CGCCCGTTTC.CAAAAAACGC | A | x | 34998_59_2011 | UnannotatedRegion |
| 1243 | CGCCCGTTTC.CAAAAAACGC | A | x | 34999_63_2011 | UnannotatedRegion |
| 1243 | CGCCCGTTTC.CAAAAAACGC | A | x | 35000_75_2011 | UnannotatedRegion |
| 1243 | CGCCCGTTTC.CAAAAAACGC | A | x | 35001_76_2011 | UnannotatedRegion |
| 1243 | CGCCCGTTTC.CAAAAAACGC | A | x | 35002_120_2011 | UnannotatedRegion |
| 1243 | CGCCCGTTTC.CAAAAAACGC | A | x | 35003_403_2011 | UnannotatedRegion |
| 1243 | CGCCCGTTTC.CAAAAAACGC | A | x | 35004_12_15398_XS2_1 | UnannotatedRegion |
| 1243 | CGCCCGTTTC.CAAAAAACGC | A | x | 35005_12_13952_XS2_1 | UnannotatedRegion |
| 1243 | CGCCCGTTTC.CAAAAAACGC | A | x | 35006_12_15317_XS2_1 | UnannotatedRegion |
| 1243 | CGCCCGTTTC.CAAAAAACGC | A | x | 35007_12_15047_XS2_1 | UnannotatedRegion |
| 1243 | CGCCCGTTTC.CAAAAAACGC | A | x | 35008_12_15661_XS2_1 | UnannotatedRegion |
| 1243 | CGCCCGTTTC.CAAAAAACGC | A | x | 35009_12_15657_XS2_1 | UnannotatedRegion |
| 1243 | CGCCCGTTTC.CAAAAAACGC | A | x | 35010_12_17186_XS2_1 | UnannotatedRegion |
| 1243 | CGCCCGTTTC.CAAAAAACGC | A | x | 35011_12_17194_XS2_1 | UnannotatedRegion |
| 1243 | CGCCCGTTTC.CAAAAAACGC | A | x | 35012_12_17009_XS2_1 | UnannotatedRegion |
| 1243 | CGCCCGTTTC.CAAAAAACGC | A | x | 35013_12_14973_XS2_1 | UnannotatedRegion |
|  |  |  |  |  |  |
| 1259 | CGCCTCAAGG.TTCAGACGGC | G | 19586 F | 19260_WUE_2594 | UnannotatedRegion |
| 1259 | CGCCTCAAGG.TTCAGACGGC | A | x | 34990_Tchad54_11 | UnannotatedRegion |
| 1259 | CGCCTCAAGG.TTCAGACGGC | A | x | 34991_Tchad78_11 | UnannotatedRegion |
| 1259 | CGCCTCAAGG.TTCAGACGGC | A | x | 34992_Tchad95_11 | UnannotatedRegion |
| 1259 | CGCCTCAAGG.TTCAGACGGC | A | x | 34994_Tchad106_11 | UnannotatedRegion |
| 1259 | CGCCTCAAGG.TTCAGACGGC | A | x | 34995_8_2011 | UnannotatedRegion |
| 1259 | CGCCTCAAGG.TTCAGACGGC | A | x | 34996_10_2011 | UnannotatedRegion |
| 1259 | CGCCTCAAGG.TTCAGACGGC | A | x | 34997_39_2011 | UnannotatedRegion |
| 1259 | CGCCTCAAGG.TTCAGACGGC | A | x | 34998_59_2011 | UnannotatedRegion |
| 1259 | CGCCTCAAGG.TTCAGACGGC | A | x | 34999_63_2011 | UnannotatedRegion |
| 1259 | CGCCTCAAGG.TTCAGACGGC | A | x | 35000_75_2011 | UnannotatedRegion |
| 1259 | CGCCTCAAGG.TTCAGACGGC | A | x | 35001_76_2011 | UnannotatedRegion |
| 1259 | CGCCTCAAGG.TTCAGACGGC | A | x | 35002_120_2011 | UnannotatedRegion |
| 1259 | CGCCTCAAGG.TTCAGACGGC | A | x | 35003_403_2011 | UnannotatedRegion |
| 1259 | CGCCTCAAGG.TTCAGACGGC | A | x | 35004_12_15398_XS2_1 | UnannotatedRegion |
| 1259 | CGCCTCAAGG.TTCAGACGGC | A | x | 35005_12_13952_XS2_1 | UnannotatedRegion |
| 1259 | CGCCTCAAGG.TTCAGACGGC | A | x | 35006_12_15317_XS2_1 | UnannotatedRegion |
| 1259 | CGCCTCAAGG.TTCAGACGGC | A | x | 35007_12_15047_XS2_1 | UnannotatedRegion |
| 1259 | CGCCTCAAGG.TTCAGACGGC | A | x | 35008_12_15661_XS2_1 | UnannotatedRegion |
| 1259 | CGCCTCAAGG.TTCAGACGGC | A | x | 35009_12_15657_XS2_1 | UnannotatedRegion |
| 1259 | CGCCTCAAGG.TTCAGACGGC | A | x | 35010_12_17186_XS2_1 | UnannotatedRegion |
| 1259 | CGCCTCAAGG.TTCAGACGGC | A | x | 35011_12_17194_XS2_1 | UnannotatedRegion |
| 1259 | CGCCTCAAGG.TTCAGACGGC | A | x | 35012_12_17009_XS2_1 | UnannotatedRegion |
| 1259 | CGCCTCAAGG.TTCAGACGGC | A | x | 35013_12_14973_XS2_1 | UnannotatedRegion |
|  |  |  |  |  |  |
| 1287 | CGCTTCCCGA.ATGATAGGCA | T | 856308 F | 19260_WUE_2594 | UnannotatedRegion |
| 1287 | CGCTTCCCGA.ATGATAGGCA | C | x | 34990_Tchad54_11 | UnannotatedRegion |
| 1287 | CGCTTCCCGA.ATGATAGGCA | C | x | 34991_Tchad78_11 | UnannotatedRegion |
| 1287 | CGCTTCCCGA.ATGATAGGCA | C | x | 34992_Tchad95_11 | UnannotatedRegion |
| 1287 | CGCTTCCCGA.ATGATAGGCA | C | x | 34994_Tchad106_11 | UnannotatedRegion |
| 1287 | CGCTTCCCGA.ATGATAGGCA | C | x | 34995_8_2011 | UnannotatedRegion |
| 1287 | CGCTTCCCGA.ATGATAGGCA | C | x | 34996_10_2011 | UnannotatedRegion |
| 1287 | CGCTTCCCGA.ATGATAGGCA | C | x | 34997_39_2011 | UnannotatedRegion |
| 1287 | CGCTTCCCGA.ATGATAGGCA | C | x | 34998_59_2011 | UnannotatedRegion |
| 1287 | CGCTTCCCGA.ATGATAGGCA | C | x | 34999_63_2011 | UnannotatedRegion |
| 1287 | CGCTTCCCGA.ATGATAGGCA | C | x | 35000_75_2011 | UnannotatedRegion |
| 1287 | CGCTTCCCGA.ATGATAGGCA | C | x | 35001_76_2011 | UnannotatedRegion |
| 1287 | CGCTTCCCGA.ATGATAGGCA | C | x | 35002_120_2011 | UnannotatedRegion |
| 1287 | CGCTTCCCGA.ATGATAGGCA | C | x | 35003_403_2011 | UnannotatedRegion |
| 1287 | CGCTTCCCGA.ATGATAGGCA | C | x | 35004_12_15398_XS2_1 | UnannotatedRegion |
| 1287 | CGCTTCCCGA.ATGATAGGCA | C | x | 35005_12_13952_XS2_1 | UnannotatedRegion |
| 1287 | CGCTTCCCGA.ATGATAGGCA | C | x | 35006_12_15317_XS2_1 | UnannotatedRegion |
| 1287 | CGCTTCCCGA.ATGATAGGCA | C | x | 35007_12_15047_XS2_1 | UnannotatedRegion |
| 1287 | CGCTTCCCGA.ATGATAGGCA | C | x | 35008_12_15661_XS2_1 | UnannotatedRegion |
| 1287 | CGCTTCCCGA.ATGATAGGCA | C | x | 35009_12_15657_XS2_1 | UnannotatedRegion |
| 1287 | CGCTTCCCGA.ATGATAGGCA | C | x | 35010_12_17186_XS2_1 | UnannotatedRegion |
| 1287 | CGCTTCCCGA.ATGATAGGCA | C | x | 35011_12_17194_XS2_1 | UnannotatedRegion |
| 1287 | CGCTTCCCGA.ATGATAGGCA | C | x | 35012_12_17009_XS2_1 | UnannotatedRegion |
| 1287 | CGCTTCCCGA.ATGATAGGCA | C | x | 35013_12_14973_XS2_1 | UnannotatedRegion |
|  |  |  |  |  |  |
| 1292 | CGCTTTGAAG.CAGGCAATGC | A | 915990 R | 19260_WUE_2594 | UnannotatedRegion |
| 1292 | CGCTTTGAAG.CAGGCAATGC | G | x | 34990_Tchad54_11 | UnannotatedRegion |
| 1292 | CGCTTTGAAG.CAGGCAATGC | G | x | 34991_Tchad78_11 | UnannotatedRegion |
| 1292 | CGCTTTGAAG.CAGGCAATGC | G | x | 34992_Tchad95_11 | UnannotatedRegion |
| 1292 | CGCTTTGAAG.CAGGCAATGC | G | x | 34994_Tchad106_11 | UnannotatedRegion |
| 1292 | CGCTTTGAAG.CAGGCAATGC | G | x | 34995_8_2011 | UnannotatedRegion |
| 1292 | CGCTTTGAAG.CAGGCAATGC | G | x | 34996_10_2011 | UnannotatedRegion |
| 1292 | CGCTTTGAAG.CAGGCAATGC | G | x | 34997_39_2011 | UnannotatedRegion |
| 1292 | CGCTTTGAAG.CAGGCAATGC | G | x | 34998_59_2011 | UnannotatedRegion |
| 1292 | CGCTTTGAAG.CAGGCAATGC | G | x | 34999_63_2011 | UnannotatedRegion |
| 1292 | CGCTTTGAAG.CAGGCAATGC | G | x | 35000_75_2011 | UnannotatedRegion |
| 1292 | CGCTTTGAAG.CAGGCAATGC | G | x | 35001_76_2011 | UnannotatedRegion |
| 1292 | CGCTTTGAAG.CAGGCAATGC | G | x | 35002_120_2011 | UnannotatedRegion |
| 1292 | CGCTTTGAAG.CAGGCAATGC | G | x | 35003_403_2011 | UnannotatedRegion |
| 1292 | CGCTTTGAAG.CAGGCAATGC | G | x | 35004_12_15398_XS2_1 | UnannotatedRegion |
| 1292 | CGCTTTGAAG.CAGGCAATGC | G | x | 35005_12_13952_XS2_1 | UnannotatedRegion |
| 1292 | CGCTTTGAAG.CAGGCAATGC | G | x | 35006_12_15317_XS2_1 | UnannotatedRegion |
| 1292 | CGCTTTGAAG.CAGGCAATGC | G | x | 35007_12_15047_XS2_1 | UnannotatedRegion |
| 1292 | CGCTTTGAAG.CAGGCAATGC | G | x | 35008_12_15661_XS2_1 | UnannotatedRegion |
| 1292 | CGCTTTGAAG.CAGGCAATGC | G | x | 35009_12_15657_XS2_1 | UnannotatedRegion |
| 1292 | CGCTTTGAAG.CAGGCAATGC | G | x | 35010_12_17186_XS2_1 | UnannotatedRegion |
| 1292 | CGCTTTGAAG.CAGGCAATGC | G | x | 35011_12_17194_XS2_1 | UnannotatedRegion |
| 1292 | CGCTTTGAAG.CAGGCAATGC | G | x | 35012_12_17009_XS2_1 | UnannotatedRegion |
| 1292 | CGCTTTGAAG.CAGGCAATGC | G | x | 35013_12_14973_XS2_1 | UnannotatedRegion |
|  |  |  |  |  |  |
| 1303 | CGGATTGCCG.CGGGACTTGC | T | 1144672 F | 19260_WUE_2594 | UnannotatedRegion |
| 1303 | CGGATTGCCG.CGGGACTTGC | C | x | 34990_Tchad54_11 | UnannotatedRegion |
| 1303 | CGGATTGCCG.CGGGACTTGC | C | x | 34991_Tchad78_11 | UnannotatedRegion |
| 1303 | CGGATTGCCG.CGGGACTTGC | C | x | 34992_Tchad95_11 | UnannotatedRegion |
| 1303 | CGGATTGCCG.CGGGACTTGC | C | x | 34994_Tchad106_11 | UnannotatedRegion |
| 1303 | CGGATTGCCG.CGGGACTTGC | C | x | 34995_8_2011 | UnannotatedRegion |
| 1303 | CGGATTGCCG.CGGGACTTGC | C | x | 34996_10_2011 | UnannotatedRegion |
| 1303 | CGGATTGCCG.CGGGACTTGC | C | x | 34997_39_2011 | UnannotatedRegion |
| 1303 | CGGATTGCCG.CGGGACTTGC | C | x | 34998_59_2011 | UnannotatedRegion |
| 1303 | CGGATTGCCG.CGGGACTTGC | C | x | 34999_63_2011 | UnannotatedRegion |
| 1303 | CGGATTGCCG.CGGGACTTGC | C | x | 35000_75_2011 | UnannotatedRegion |
| 1303 | CGGATTGCCG.CGGGACTTGC | C | x | 35001_76_2011 | UnannotatedRegion |
| 1303 | CGGATTGCCG.CGGGACTTGC | C | x | 35002_120_2011 | UnannotatedRegion |
| 1303 | CGGATTGCCG.CGGGACTTGC | C | x | 35003_403_2011 | UnannotatedRegion |
| 1303 | CGGATTGCCG.CGGGACTTGC | C | x | 35004_12_15398_XS2_1 | UnannotatedRegion |
| 1303 | CGGATTGCCG.CGGGACTTGC | C | x | 35005_12_13952_XS2_1 | UnannotatedRegion |
| 1303 | CGGATTGCCG.CGGGACTTGC | C | x | 35006_12_15317_XS2_1 | UnannotatedRegion |
| 1303 | CGGATTGCCG.CGGGACTTGC | C | x | 35007_12_15047_XS2_1 | UnannotatedRegion |
| 1303 | CGGATTGCCG.CGGGACTTGC | C | x | 35008_12_15661_XS2_1 | UnannotatedRegion |
| 1303 | CGGATTGCCG.CGGGACTTGC | C | x | 35009_12_15657_XS2_1 | UnannotatedRegion |
| 1303 | CGGATTGCCG.CGGGACTTGC | C | x | 35010_12_17186_XS2_1 | UnannotatedRegion |
| 1303 | CGGATTGCCG.CGGGACTTGC | C | x | 35011_12_17194_XS2_1 | UnannotatedRegion |
| 1303 | CGGATTGCCG.CGGGACTTGC | C | x | 35012_12_17009_XS2_1 | UnannotatedRegion |
| 1303 | CGGATTGCCG.CGGGACTTGC | C | x | 35013_12_14973_XS2_1 | UnannotatedRegion |
|  |  |  |  |  |  |
| 1330 | CGGCTTCAAA.ACAAAAGGAA | C | 510484 R | 19260_WUE_2594 | UnannotatedRegion |
| 1330 | CGGCTTCAAA.ACAAAAGGAA | T | x | 34990_Tchad54_11 | UnannotatedRegion |
| 1330 | CGGCTTCAAA.ACAAAAGGAA | T | x | 34991_Tchad78_11 | UnannotatedRegion |
| 1330 | CGGCTTCAAA.ACAAAAGGAA | T | x | 34992_Tchad95_11 | UnannotatedRegion |
| 1330 | CGGCTTCAAA.ACAAAAGGAA | T | x | 34994_Tchad106_11 | UnannotatedRegion |
| 1330 | CGGCTTCAAA.ACAAAAGGAA | T | x | 34995_8_2011 | UnannotatedRegion |
| 1330 | CGGCTTCAAA.ACAAAAGGAA | T | x | 34996_10_2011 | UnannotatedRegion |
| 1330 | CGGCTTCAAA.ACAAAAGGAA | T | x | 34997_39_2011 | UnannotatedRegion |
| 1330 | CGGCTTCAAA.ACAAAAGGAA | T | x | 34998_59_2011 | UnannotatedRegion |
| 1330 | CGGCTTCAAA.ACAAAAGGAA | T | x | 34999_63_2011 | UnannotatedRegion |
| 1330 | CGGCTTCAAA.ACAAAAGGAA | T | x | 35000_75_2011 | UnannotatedRegion |
| 1330 | CGGCTTCAAA.ACAAAAGGAA | T | x | 35001_76_2011 | UnannotatedRegion |
| 1330 | CGGCTTCAAA.ACAAAAGGAA | T | x | 35002_120_2011 | UnannotatedRegion |
| 1330 | CGGCTTCAAA.ACAAAAGGAA | T | x | 35003_403_2011 | UnannotatedRegion |
| 1330 | CGGCTTCAAA.ACAAAAGGAA | T | x | 35004_12_15398_XS2_1 | UnannotatedRegion |
| 1330 | CGGCTTCAAA.ACAAAAGGAA | T | x | 35005_12_13952_XS2_1 | UnannotatedRegion |
| 1330 | CGGCTTCAAA.ACAAAAGGAA | T | x | 35006_12_15317_XS2_1 | UnannotatedRegion |
| 1330 | CGGCTTCAAA.ACAAAAGGAA | T | x | 35007_12_15047_XS2_1 | UnannotatedRegion |
| 1330 | CGGCTTCAAA.ACAAAAGGAA | T | x | 35008_12_15661_XS2_1 | UnannotatedRegion |
| 1330 | CGGCTTCAAA.ACAAAAGGAA | T | x | 35009_12_15657_XS2_1 | UnannotatedRegion |
| 1330 | CGGCTTCAAA.ACAAAAGGAA | T | x | 35010_12_17186_XS2_1 | UnannotatedRegion |
| 1330 | CGGCTTCAAA.ACAAAAGGAA | T | x | 35011_12_17194_XS2_1 | UnannotatedRegion |
| 1330 | CGGCTTCAAA.ACAAAAGGAA | T | x | 35012_12_17009_XS2_1 | UnannotatedRegion |
| 1330 | CGGCTTCAAA.ACAAAAGGAA | T | x | 35013_12_14973_XS2_1 | UnannotatedRegion |
|  |  |  |  |  |  |
| 1362 | CGTCAAAAAA.CCGTTACCTC | A | x | 34990_Tchad54_11 | NotInAnnotatedGenome |
| 1362 | CGTCAAAAAA.CCGTTACCTC | A | x | 34991_Tchad78_11 | NotInAnnotatedGenome |
| 1362 | CGTCAAAAAA.CCGTTACCTC | C | x | 34994_Tchad106_11 | NotInAnnotatedGenome |
| 1362 | CGTCAAAAAA.CCGTTACCTC | A | x | 34995_8_2011 | NotInAnnotatedGenome |
| 1362 | CGTCAAAAAA.CCGTTACCTC | A | x | 34996_10_2011 | NotInAnnotatedGenome |
| 1362 | CGTCAAAAAA.CCGTTACCTC | A | x | 34997_39_2011 | NotInAnnotatedGenome |
| 1362 | CGTCAAAAAA.CCGTTACCTC | A | x | 34998_59_2011 | NotInAnnotatedGenome |
| 1362 | CGTCAAAAAA.CCGTTACCTC | A | x | 34999_63_2011 | NotInAnnotatedGenome |
| 1362 | CGTCAAAAAA.CCGTTACCTC | A | x | 35000_75_2011 | NotInAnnotatedGenome |
| 1362 | CGTCAAAAAA.CCGTTACCTC | A | x | 35001_76_2011 | NotInAnnotatedGenome |
| 1362 | CGTCAAAAAA.CCGTTACCTC | A | x | 35002_120_2011 | NotInAnnotatedGenome |
| 1362 | CGTCAAAAAA.CCGTTACCTC | A | x | 35003_403_2011 | NotInAnnotatedGenome |
| 1362 | CGTCAAAAAA.CCGTTACCTC | A | x | 35004_12_15398_XS2_1 | NotInAnnotatedGenome |
| 1362 | CGTCAAAAAA.CCGTTACCTC | A | x | 35005_12_13952_XS2_1 | NotInAnnotatedGenome |
| 1362 | CGTCAAAAAA.CCGTTACCTC | A | x | 35006_12_15317_XS2_1 | NotInAnnotatedGenome |
| 1362 | CGTCAAAAAA.CCGTTACCTC | A | x | 35010_12_17186_XS2_1 | NotInAnnotatedGenome |
| 1362 | CGTCAAAAAA.CCGTTACCTC | A | x | 35011_12_17194_XS2_1 | NotInAnnotatedGenome |
| 1362 | CGTCAAAAAA.CCGTTACCTC | A | x | 35012_12_17009_XS2_1 | NotInAnnotatedGenome |
|  |  |  |  |  |  |
| 1382 | CGTTATAATC.GGGGTTGTGC | C | 211542 R | 19260_WUE_2594 | UnannotatedRegion |
| 1382 | CGTTATAATC.GGGGTTGTGC | C | x | 34990_Tchad54_11 | UnannotatedRegion |
| 1382 | CGTTATAATC.GGGGTTGTGC | T | x | 34991_Tchad78_11 | UnannotatedRegion |
| 1382 | CGTTATAATC.GGGGTTGTGC | C | x | 34992_Tchad95_11 | UnannotatedRegion |
| 1382 | CGTTATAATC.GGGGTTGTGC | C | x | 34994_Tchad106_11 | UnannotatedRegion |
| 1382 | CGTTATAATC.GGGGTTGTGC | C | x | 34995_8_2011 | UnannotatedRegion |
| 1382 | CGTTATAATC.GGGGTTGTGC | C | x | 34996_10_2011 | UnannotatedRegion |
| 1382 | CGTTATAATC.GGGGTTGTGC | C | x | 34997_39_2011 | UnannotatedRegion |
| 1382 | CGTTATAATC.GGGGTTGTGC | C | x | 34998_59_2011 | UnannotatedRegion |
| 1382 | CGTTATAATC.GGGGTTGTGC | C | x | 34999_63_2011 | UnannotatedRegion |
| 1382 | CGTTATAATC.GGGGTTGTGC | C | x | 35000_75_2011 | UnannotatedRegion |
| 1382 | CGTTATAATC.GGGGTTGTGC | C | x | 35001_76_2011 | UnannotatedRegion |
| 1382 | CGTTATAATC.GGGGTTGTGC | C | x | 35002_120_2011 | UnannotatedRegion |
| 1382 | CGTTATAATC.GGGGTTGTGC | T | x | 35003_403_2011 | UnannotatedRegion |
| 1382 | CGTTATAATC.GGGGTTGTGC | C | x | 35004_12_15398_XS2_1 | UnannotatedRegion |
| 1382 | CGTTATAATC.GGGGTTGTGC | C | x | 35005_12_13952_XS2_1 | UnannotatedRegion |
| 1382 | CGTTATAATC.GGGGTTGTGC | C | x | 35006_12_15317_XS2_1 | UnannotatedRegion |
| 1382 | CGTTATAATC.GGGGTTGTGC | C | x | 35007_12_15047_XS2_1 | UnannotatedRegion |
| 1382 | CGTTATAATC.GGGGTTGTGC | C | x | 35008_12_15661_XS2_1 | UnannotatedRegion |
| 1382 | CGTTATAATC.GGGGTTGTGC | C | x | 35009_12_15657_XS2_1 | UnannotatedRegion |
| 1382 | CGTTATAATC.GGGGTTGTGC | C | x | 35010_12_17186_XS2_1 | UnannotatedRegion |
| 1382 | CGTTATAATC.GGGGTTGTGC | C | x | 35011_12_17194_XS2_1 | UnannotatedRegion |
| 1382 | CGTTATAATC.GGGGTTGTGC | C | x | 35012_12_17009_XS2_1 | UnannotatedRegion |
| 1382 | CGTTATAATC.GGGGTTGTGC | C | x | 35013_12_14973_XS2_1 | UnannotatedRegion |
|  |  |  |  |  |  |
| 1385 | CGTTGATACT.GGATTAAGAC | C | 1785322 F | 19260_WUE_2594 | UnannotatedRegion |
| 1385 | CGTTGATACT.GGATTAAGAC | C | x | 34990_Tchad54_11 | UnannotatedRegion |
| 1385 | CGTTGATACT.GGATTAAGAC | C | x | 34991_Tchad78_11 | UnannotatedRegion |
| 1385 | CGTTGATACT.GGATTAAGAC | C | x | 34992_Tchad95_11 | UnannotatedRegion |
| 1385 | CGTTGATACT.GGATTAAGAC | C | x | 34994_Tchad106_11 | UnannotatedRegion |
| 1385 | CGTTGATACT.GGATTAAGAC | T | x | 34995_8_2011 | UnannotatedRegion |
| 1385 | CGTTGATACT.GGATTAAGAC | T | x | 34996_10_2011 | UnannotatedRegion |
| 1385 | CGTTGATACT.GGATTAAGAC | C | x | 34997_39_2011 | UnannotatedRegion |
| 1385 | CGTTGATACT.GGATTAAGAC | C | x | 34998_59_2011 | UnannotatedRegion |
| 1385 | CGTTGATACT.GGATTAAGAC | C | x | 34999_63_2011 | UnannotatedRegion |
| 1385 | CGTTGATACT.GGATTAAGAC | C | x | 35000_75_2011 | UnannotatedRegion |
| 1385 | CGTTGATACT.GGATTAAGAC | C | x | 35001_76_2011 | UnannotatedRegion |
| 1385 | CGTTGATACT.GGATTAAGAC | C | x | 35002_120_2011 | UnannotatedRegion |
| 1385 | CGTTGATACT.GGATTAAGAC | C | x | 35003_403_2011 | UnannotatedRegion |
| 1385 | CGTTGATACT.GGATTAAGAC | C | x | 35004_12_15398_XS2_1 | UnannotatedRegion |
| 1385 | CGTTGATACT.GGATTAAGAC | C | x | 35005_12_13952_XS2_1 | UnannotatedRegion |
| 1385 | CGTTGATACT.GGATTAAGAC | C | x | 35006_12_15317_XS2_1 | UnannotatedRegion |
| 1385 | CGTTGATACT.GGATTAAGAC | C | x | 35007_12_15047_XS2_1 | UnannotatedRegion |
| 1385 | CGTTGATACT.GGATTAAGAC | C | x | 35008_12_15661_XS2_1 | UnannotatedRegion |
| 1385 | CGTTGATACT.GGATTAAGAC | C | x | 35009_12_15657_XS2_1 | UnannotatedRegion |
| 1385 | CGTTGATACT.GGATTAAGAC | C | x | 35010_12_17186_XS2_1 | UnannotatedRegion |
| 1385 | CGTTGATACT.GGATTAAGAC | C | x | 35011_12_17194_XS2_1 | UnannotatedRegion |
| 1385 | CGTTGATACT.GGATTAAGAC | C | x | 35012_12_17009_XS2_1 | UnannotatedRegion |
| 1385 | CGTTGATACT.GGATTAAGAC | C | x | 35013_12_14973_XS2_1 | UnannotatedRegion |
|  |  |  |  |  |  |
| 1400 | CTATGCCCCG.CAATCCTGCC | C | 1010178 F | 19260_WUE_2594 | UnannotatedRegion |
| 1400 | CTATGCCCCG.CAATCCTGCC | C | x | 34990_Tchad54_11 | UnannotatedRegion |
| 1400 | CTATGCCCCG.CAATCCTGCC | C | x | 34991_Tchad78_11 | UnannotatedRegion |
| 1400 | CTATGCCCCG.CAATCCTGCC | C | x | 34992_Tchad95_11 | UnannotatedRegion |
| 1400 | CTATGCCCCG.CAATCCTGCC | C | x | 34994_Tchad106_11 | UnannotatedRegion |
| 1400 | CTATGCCCCG.CAATCCTGCC | C | x | 34995_8_2011 | UnannotatedRegion |
| 1400 | CTATGCCCCG.CAATCCTGCC | C | x | 34996_10_2011 | UnannotatedRegion |
| 1400 | CTATGCCCCG.CAATCCTGCC | C | x | 34997_39_2011 | UnannotatedRegion |
| 1400 | CTATGCCCCG.CAATCCTGCC | C | x | 34998_59_2011 | UnannotatedRegion |
| 1400 | CTATGCCCCG.CAATCCTGCC | C | x | 34999_63_2011 | UnannotatedRegion |
| 1400 | CTATGCCCCG.CAATCCTGCC | C | x | 35000_75_2011 | UnannotatedRegion |
| 1400 | CTATGCCCCG.CAATCCTGCC | C | x | 35001_76_2011 | UnannotatedRegion |
| 1400 | CTATGCCCCG.CAATCCTGCC | C | x | 35002_120_2011 | UnannotatedRegion |
| 1400 | CTATGCCCCG.CAATCCTGCC | C | x | 35003_403_2011 | UnannotatedRegion |
| 1400 | CTATGCCCCG.CAATCCTGCC | C | x | 35004_12_15398_XS2_1 | UnannotatedRegion |
| 1400 | CTATGCCCCG.CAATCCTGCC | C | x | 35005_12_13952_XS2_1 | UnannotatedRegion |
| 1400 | CTATGCCCCG.CAATCCTGCC | C | x | 35006_12_15317_XS2_1 | UnannotatedRegion |
| 1400 | CTATGCCCCG.CAATCCTGCC | C | x | 35007_12_15047_XS2_1 | UnannotatedRegion |
| 1400 | CTATGCCCCG.CAATCCTGCC | C | x | 35008_12_15661_XS2_1 | UnannotatedRegion |
| 1400 | CTATGCCCCG.CAATCCTGCC | C | x | 35009_12_15657_XS2_1 | UnannotatedRegion |
| 1400 | CTATGCCCCG.CAATCCTGCC | C | x | 35010_12_17186_XS2_1 | UnannotatedRegion |
| 1400 | CTATGCCCCG.CAATCCTGCC | C | x | 35011_12_17194_XS2_1 | UnannotatedRegion |
| 1400 | CTATGCCCCG.CAATCCTGCC | C | x | 35012_12_17009_XS2_1 | UnannotatedRegion |
| 1400 | CTATGCCCCG.CAATCCTGCC | A | x | 35013_12_14973_XS2_1 | UnannotatedRegion |
|  |  |  |  |  |  |
| 1412 | CTGATTGCCG.TGAAGCAATA | G | 234757 F | 19260_WUE_2594 | UnannotatedRegion |
| 1412 | CTGATTGCCG.TGAAGCAATA | A | x | 34990_Tchad54_11 | UnannotatedRegion |
| 1412 | CTGATTGCCG.TGAAGCAATA | A | x | 34991_Tchad78_11 | UnannotatedRegion |
| 1412 | CTGATTGCCG.TGAAGCAATA | A | x | 34992_Tchad95_11 | UnannotatedRegion |
| 1412 | CTGATTGCCG.TGAAGCAATA | A | x | 34995_8_2011 | UnannotatedRegion |
| 1412 | CTGATTGCCG.TGAAGCAATA | A | x | 34996_10_2011 | UnannotatedRegion |
| 1412 | CTGATTGCCG.TGAAGCAATA | A | x | 34997_39_2011 | UnannotatedRegion |
| 1412 | CTGATTGCCG.TGAAGCAATA | A | x | 34998_59_2011 | UnannotatedRegion |
| 1412 | CTGATTGCCG.TGAAGCAATA | A | x | 34999_63_2011 | UnannotatedRegion |
| 1412 | CTGATTGCCG.TGAAGCAATA | A | x | 35000_75_2011 | UnannotatedRegion |
| 1412 | CTGATTGCCG.TGAAGCAATA | A | x | 35001_76_2011 | UnannotatedRegion |
| 1412 | CTGATTGCCG.TGAAGCAATA | A | x | 35002_120_2011 | UnannotatedRegion |
| 1412 | CTGATTGCCG.TGAAGCAATA | A | x | 35003_403_2011 | UnannotatedRegion |
| 1412 | CTGATTGCCG.TGAAGCAATA | A | x | 35004_12_15398_XS2_1 | UnannotatedRegion |
| 1412 | CTGATTGCCG.TGAAGCAATA | A | x | 35005_12_13952_XS2_1 | UnannotatedRegion |
| 1412 | CTGATTGCCG.TGAAGCAATA | A | x | 35006_12_15317_XS2_1 | UnannotatedRegion |
| 1412 | CTGATTGCCG.TGAAGCAATA | A | x | 35007_12_15047_XS2_1 | UnannotatedRegion |
| 1412 | CTGATTGCCG.TGAAGCAATA | A | x | 35008_12_15661_XS2_1 | UnannotatedRegion |
| 1412 | CTGATTGCCG.TGAAGCAATA | A | x | 35009_12_15657_XS2_1 | UnannotatedRegion |
| 1412 | CTGATTGCCG.TGAAGCAATA | A | x | 35010_12_17186_XS2_1 | UnannotatedRegion |
| 1412 | CTGATTGCCG.TGAAGCAATA | A | x | 35011_12_17194_XS2_1 | UnannotatedRegion |
| 1412 | CTGATTGCCG.TGAAGCAATA | A | x | 35012_12_17009_XS2_1 | UnannotatedRegion |
| 1412 | CTGATTGCCG.TGAAGCAATA | A | x | 35013_12_14973_XS2_1 | UnannotatedRegion |
|  |  |  |  |  |  |
| 1422 | CTGCTCTACC.ACTGAGCTAA | G | 9266 F | 19260_WUE_2594 | NotProteinCoding |
| 1422 | CTGCTCTACC.ACTGAGCTAA | A | x | 34990_Tchad54_11 | NotProteinCoding |
| 1422 | CTGCTCTACC.ACTGAGCTAA | A | x | 34991_Tchad78_11 | NotProteinCoding |
| 1422 | CTGCTCTACC.ACTGAGCTAA | A | x | 34992_Tchad95_11 | NotProteinCoding |
| 1422 | CTGCTCTACC.ACTGAGCTAA | A | x | 34994_Tchad106_11 | NotProteinCoding |
| 1422 | CTGCTCTACC.ACTGAGCTAA | A | x | 34995_8_2011 | NotProteinCoding |
| 1422 | CTGCTCTACC.ACTGAGCTAA | A | x | 34996_10_2011 | NotProteinCoding |
| 1422 | CTGCTCTACC.ACTGAGCTAA | A | x | 34997_39_2011 | NotProteinCoding |
| 1422 | CTGCTCTACC.ACTGAGCTAA | A | x | 34998_59_2011 | NotProteinCoding |
| 1422 | CTGCTCTACC.ACTGAGCTAA | A | x | 34999_63_2011 | NotProteinCoding |
| 1422 | CTGCTCTACC.ACTGAGCTAA | A | x | 35000_75_2011 | NotProteinCoding |
| 1422 | CTGCTCTACC.ACTGAGCTAA | A | x | 35001_76_2011 | NotProteinCoding |
| 1422 | CTGCTCTACC.ACTGAGCTAA | A | x | 35002_120_2011 | NotProteinCoding |
| 1422 | CTGCTCTACC.ACTGAGCTAA | A | x | 35003_403_2011 | NotProteinCoding |
| 1422 | CTGCTCTACC.ACTGAGCTAA | A | x | 35004_12_15398_XS2_1 | NotProteinCoding |
| 1422 | CTGCTCTACC.ACTGAGCTAA | A | x | 35005_12_13952_XS2_1 | NotProteinCoding |
| 1422 | CTGCTCTACC.ACTGAGCTAA | A | x | 35006_12_15317_XS2_1 | NotProteinCoding |
| 1422 | CTGCTCTACC.ACTGAGCTAA | A | x | 35007_12_15047_XS2_1 | NotProteinCoding |
| 1422 | CTGCTCTACC.ACTGAGCTAA | A | x | 35008_12_15661_XS2_1 | NotProteinCoding |
| 1422 | CTGCTCTACC.ACTGAGCTAA | A | x | 35009_12_15657_XS2_1 | NotProteinCoding |
| 1422 | CTGCTCTACC.ACTGAGCTAA | A | x | 35010_12_17186_XS2_1 | NotProteinCoding |
| 1422 | CTGCTCTACC.ACTGAGCTAA | A | x | 35011_12_17194_XS2_1 | NotProteinCoding |
| 1422 | CTGCTCTACC.ACTGAGCTAA | A | x | 35012_12_17009_XS2_1 | NotProteinCoding |
| 1422 | CTGCTCTACC.ACTGAGCTAA | A | x | 35013_12_14973_XS2_1 | NotProteinCoding |
|  |  |  |  |  |  |
| 1430 | CTTCAATTAT.CCCCCCCCCC | A | 1331900 F | 19260_WUE_2594 | UnannotatedRegion |
| 1430 | CTTCAATTAT.CCCCCCCCCC | A | x | 34990_Tchad54_11 | UnannotatedRegion |
| 1430 | CTTCAATTAT.CCCCCCCCCC | A | x | 34991_Tchad78_11 | UnannotatedRegion |
| 1430 | CTTCAATTAT.CCCCCCCCCC | A | x | 34992_Tchad95_11 | UnannotatedRegion |
| 1430 | CTTCAATTAT.CCCCCCCCCC | A | x | 34994_Tchad106_11 | UnannotatedRegion |
| 1430 | CTTCAATTAT.CCCCCCCCCC | C | x | 34995_8_2011 | UnannotatedRegion |
| 1430 | CTTCAATTAT.CCCCCCCCCC | C | x | 34996_10_2011 | UnannotatedRegion |
| 1430 | CTTCAATTAT.CCCCCCCCCC | A | x | 34997_39_2011 | UnannotatedRegion |
| 1430 | CTTCAATTAT.CCCCCCCCCC | A | x | 34998_59_2011 | UnannotatedRegion |
| 1430 | CTTCAATTAT.CCCCCCCCCC | A | x | 34999_63_2011 | UnannotatedRegion |
| 1430 | CTTCAATTAT.CCCCCCCCCC | A | x | 35000_75_2011 | UnannotatedRegion |
| 1430 | CTTCAATTAT.CCCCCCCCCC | A | x | 35001_76_2011 | UnannotatedRegion |
| 1430 | CTTCAATTAT.CCCCCCCCCC | A | x | 35002_120_2011 | UnannotatedRegion |
| 1430 | CTTCAATTAT.CCCCCCCCCC | A | x | 35003_403_2011 | UnannotatedRegion |
| 1430 | CTTCAATTAT.CCCCCCCCCC | A | x | 35004_12_15398_XS2_1 | UnannotatedRegion |
| 1430 | CTTCAATTAT.CCCCCCCCCC | A | x | 35005_12_13952_XS2_1 | UnannotatedRegion |
| 1430 | CTTCAATTAT.CCCCCCCCCC | A | x | 35006_12_15317_XS2_1 | UnannotatedRegion |
| 1430 | CTTCAATTAT.CCCCCCCCCC | A | x | 35007_12_15047_XS2_1 | UnannotatedRegion |
| 1430 | CTTCAATTAT.CCCCCCCCCC | A | x | 35008_12_15661_XS2_1 | UnannotatedRegion |
| 1430 | CTTCAATTAT.CCCCCCCCCC | A | x | 35009_12_15657_XS2_1 | UnannotatedRegion |
| 1430 | CTTCAATTAT.CCCCCCCCCC | A | x | 35010_12_17186_XS2_1 | UnannotatedRegion |
| 1430 | CTTCAATTAT.CCCCCCCCCC | A | x | 35011_12_17194_XS2_1 | UnannotatedRegion |
| 1430 | CTTCAATTAT.CCCCCCCCCC | A | x | 35012_12_17009_XS2_1 | UnannotatedRegion |
| 1430 | CTTCAATTAT.CCCCCCCCCC | A | x | 35013_12_14973_XS2_1 | UnannotatedRegion |
|  |  |  |  |  |  |
| 1431 | CTTCAGGACG.CTTACGTCGC | A | x | 34990_Tchad54_11 | NotInAnnotatedGenome |
| 1431 | CTTCAGGACG.CTTACGTCGC | A | x | 34991_Tchad78_11 | NotInAnnotatedGenome |
| 1431 | CTTCAGGACG.CTTACGTCGC | A | x | 34992_Tchad95_11 | NotInAnnotatedGenome |
| 1431 | CTTCAGGACG.CTTACGTCGC | A | x | 34994_Tchad106_11 | NotInAnnotatedGenome |
| 1431 | CTTCAGGACG.CTTACGTCGC | G | x | 34995_8_2011 | NotInAnnotatedGenome |
| 1431 | CTTCAGGACG.CTTACGTCGC | G | x | 34996_10_2011 | NotInAnnotatedGenome |
| 1431 | CTTCAGGACG.CTTACGTCGC | A | x | 34997_39_2011 | NotInAnnotatedGenome |
| 1431 | CTTCAGGACG.CTTACGTCGC | A | x | 34998_59_2011 | NotInAnnotatedGenome |
| 1431 | CTTCAGGACG.CTTACGTCGC | A | x | 34999_63_2011 | NotInAnnotatedGenome |
| 1431 | CTTCAGGACG.CTTACGTCGC | A | x | 35000_75_2011 | NotInAnnotatedGenome |
| 1431 | CTTCAGGACG.CTTACGTCGC | A | x | 35001_76_2011 | NotInAnnotatedGenome |
| 1431 | CTTCAGGACG.CTTACGTCGC | A | x | 35002_120_2011 | NotInAnnotatedGenome |
| 1431 | CTTCAGGACG.CTTACGTCGC | A | x | 35003_403_2011 | NotInAnnotatedGenome |
| 1431 | CTTCAGGACG.CTTACGTCGC | A | x | 35004_12_15398_XS2_1 | NotInAnnotatedGenome |
| 1431 | CTTCAGGACG.CTTACGTCGC | A | x | 35005_12_13952_XS2_1 | NotInAnnotatedGenome |
| 1431 | CTTCAGGACG.CTTACGTCGC | A | x | 35006_12_15317_XS2_1 | NotInAnnotatedGenome |
| 1431 | CTTCAGGACG.CTTACGTCGC | A | x | 35007_12_15047_XS2_1 | NotInAnnotatedGenome |
| 1431 | CTTCAGGACG.CTTACGTCGC | A | x | 35008_12_15661_XS2_1 | NotInAnnotatedGenome |
| 1431 | CTTCAGGACG.CTTACGTCGC | A | x | 35009_12_15657_XS2_1 | NotInAnnotatedGenome |
| 1431 | CTTCAGGACG.CTTACGTCGC | A | x | 35010_12_17186_XS2_1 | NotInAnnotatedGenome |
| 1431 | CTTCAGGACG.CTTACGTCGC | A | x | 35011_12_17194_XS2_1 | NotInAnnotatedGenome |
| 1431 | CTTCAGGACG.CTTACGTCGC | A | x | 35012_12_17009_XS2_1 | NotInAnnotatedGenome |
| 1431 | CTTCAGGACG.CTTACGTCGC | A | x | 35013_12_14973_XS2_1 | NotInAnnotatedGenome |
|  |  |  |  |  |  |
| 1448 | CTTGGATGCG.GAATGTCGGC | G | 1284627 R | 19260_WUE_2594 | UnannotatedRegion |
| 1448 | CTTGGATGCG.GAATGTCGGC | G | x | 34990_Tchad54_11 | UnannotatedRegion |
| 1448 | CTTGGATGCG.GAATGTCGGC | G | x | 34991_Tchad78_11 | UnannotatedRegion |
| 1448 | CTTGGATGCG.GAATGTCGGC | G | x | 34992_Tchad95_11 | UnannotatedRegion |
| 1448 | CTTGGATGCG.GAATGTCGGC | G | x | 34994_Tchad106_11 | UnannotatedRegion |
| 1448 | CTTGGATGCG.GAATGTCGGC | G | x | 34995_8_2011 | UnannotatedRegion |
| 1448 | CTTGGATGCG.GAATGTCGGC | G | x | 34996_10_2011 | UnannotatedRegion |
| 1448 | CTTGGATGCG.GAATGTCGGC | G | x | 34997_39_2011 | UnannotatedRegion |
| 1448 | CTTGGATGCG.GAATGTCGGC | G | x | 34998_59_2011 | UnannotatedRegion |
| 1448 | CTTGGATGCG.GAATGTCGGC | G | x | 34999_63_2011 | UnannotatedRegion |
| 1448 | CTTGGATGCG.GAATGTCGGC | G | x | 35000_75_2011 | UnannotatedRegion |
| 1448 | CTTGGATGCG.GAATGTCGGC | G | x | 35001_76_2011 | UnannotatedRegion |
| 1448 | CTTGGATGCG.GAATGTCGGC | G | x | 35003_403_2011 | UnannotatedRegion |
| 1448 | CTTGGATGCG.GAATGTCGGC | G | x | 35004_12_15398_XS2_1 | UnannotatedRegion |
| 1448 | CTTGGATGCG.GAATGTCGGC | G | x | 35005_12_13952_XS2_1 | UnannotatedRegion |
| 1448 | CTTGGATGCG.GAATGTCGGC | G | x | 35006_12_15317_XS2_1 | UnannotatedRegion |
| 1448 | CTTGGATGCG.GAATGTCGGC | G | x | 35007_12_15047_XS2_1 | UnannotatedRegion |
| 1448 | CTTGGATGCG.GAATGTCGGC | G | x | 35008_12_15661_XS2_1 | UnannotatedRegion |
| 1448 | CTTGGATGCG.GAATGTCGGC | G | x | 35009_12_15657_XS2_1 | UnannotatedRegion |
| 1448 | CTTGGATGCG.GAATGTCGGC | G | x | 35010_12_17186_XS2_1 | UnannotatedRegion |
| 1448 | CTTGGATGCG.GAATGTCGGC | A | x | 35011_12_17194_XS2_1 | UnannotatedRegion |
| 1448 | CTTGGATGCG.GAATGTCGGC | G | x | 35012_12_17009_XS2_1 | UnannotatedRegion |
| 1448 | CTTGGATGCG.GAATGTCGGC | G | x | 35013_12_14973_XS2_1 | UnannotatedRegion |
|  |  |  |  |  |  |
| 1452 | CTTGTCAGGT.TCGACCCAAC | T | 1995480 F | 19260_WUE_2594 | UnannotatedRegion |
| 1452 | CTTGTCAGGT.TCGACCCAAC | C | x | 34990_Tchad54_11 | UnannotatedRegion |
| 1452 | CTTGTCAGGT.TCGACCCAAC | C | x | 34991_Tchad78_11 | UnannotatedRegion |
| 1452 | CTTGTCAGGT.TCGACCCAAC | C | x | 34992_Tchad95_11 | UnannotatedRegion |
| 1452 | CTTGTCAGGT.TCGACCCAAC | C | x | 34994_Tchad106_11 | UnannotatedRegion |
| 1452 | CTTGTCAGGT.TCGACCCAAC | C | x | 34995_8_2011 | UnannotatedRegion |
| 1452 | CTTGTCAGGT.TCGACCCAAC | C | x | 34996_10_2011 | UnannotatedRegion |
| 1452 | CTTGTCAGGT.TCGACCCAAC | C | x | 34997_39_2011 | UnannotatedRegion |
| 1452 | CTTGTCAGGT.TCGACCCAAC | C | x | 34998_59_2011 | UnannotatedRegion |
| 1452 | CTTGTCAGGT.TCGACCCAAC | C | x | 34999_63_2011 | UnannotatedRegion |
| 1452 | CTTGTCAGGT.TCGACCCAAC | C | x | 35000_75_2011 | UnannotatedRegion |
| 1452 | CTTGTCAGGT.TCGACCCAAC | C | x | 35001_76_2011 | UnannotatedRegion |
| 1452 | CTTGTCAGGT.TCGACCCAAC | C | x | 35002_120_2011 | UnannotatedRegion |
| 1452 | CTTGTCAGGT.TCGACCCAAC | C | x | 35003_403_2011 | UnannotatedRegion |
| 1452 | CTTGTCAGGT.TCGACCCAAC | C | x | 35004_12_15398_XS2_1 | UnannotatedRegion |
| 1452 | CTTGTCAGGT.TCGACCCAAC | C | x | 35005_12_13952_XS2_1 | UnannotatedRegion |
| 1452 | CTTGTCAGGT.TCGACCCAAC | C | x | 35006_12_15317_XS2_1 | UnannotatedRegion |
| 1452 | CTTGTCAGGT.TCGACCCAAC | C | x | 35007_12_15047_XS2_1 | UnannotatedRegion |
| 1452 | CTTGTCAGGT.TCGACCCAAC | C | x | 35008_12_15661_XS2_1 | UnannotatedRegion |
| 1452 | CTTGTCAGGT.TCGACCCAAC | C | x | 35009_12_15657_XS2_1 | UnannotatedRegion |
| 1452 | CTTGTCAGGT.TCGACCCAAC | C | x | 35010_12_17186_XS2_1 | UnannotatedRegion |
| 1452 | CTTGTCAGGT.TCGACCCAAC | C | x | 35011_12_17194_XS2_1 | UnannotatedRegion |
| 1452 | CTTGTCAGGT.TCGACCCAAC | C | x | 35012_12_17009_XS2_1 | UnannotatedRegion |
| 1452 | CTTGTCAGGT.TCGACCCAAC | C | x | 35013_12_14973_XS2_1 | UnannotatedRegion |
|  |  |  |  |  |  |
| 1463 | CTTTTAAAAT.CCGTCTGAAA | G | 1134555 R | 19260_WUE_2594 | UnannotatedRegion |
| 1463 | CTTTTAAAAT.CCGTCTGAAA | T | x | 34990_Tchad54_11 | UnannotatedRegion |
| 1463 | CTTTTAAAAT.CCGTCTGAAA | T | x | 34991_Tchad78_11 | UnannotatedRegion |
| 1463 | CTTTTAAAAT.CCGTCTGAAA | T | x | 34992_Tchad95_11 | UnannotatedRegion |
| 1463 | CTTTTAAAAT.CCGTCTGAAA | T | x | 34994_Tchad106_11 | UnannotatedRegion |
| 1463 | CTTTTAAAAT.CCGTCTGAAA | T | x | 34995_8_2011 | UnannotatedRegion |
| 1463 | CTTTTAAAAT.CCGTCTGAAA | T | x | 34996_10_2011 | UnannotatedRegion |
| 1463 | CTTTTAAAAT.CCGTCTGAAA | T | x | 34997_39_2011 | UnannotatedRegion |
| 1463 | CTTTTAAAAT.CCGTCTGAAA | T | x | 34998_59_2011 | UnannotatedRegion |
| 1463 | CTTTTAAAAT.CCGTCTGAAA | T | x | 34999_63_2011 | UnannotatedRegion |
| 1463 | CTTTTAAAAT.CCGTCTGAAA | T | x | 35000_75_2011 | UnannotatedRegion |
| 1463 | CTTTTAAAAT.CCGTCTGAAA | T | x | 35001_76_2011 | UnannotatedRegion |
| 1463 | CTTTTAAAAT.CCGTCTGAAA | T | x | 35002_120_2011 | UnannotatedRegion |
| 1463 | CTTTTAAAAT.CCGTCTGAAA | T | x | 35003_403_2011 | UnannotatedRegion |
| 1463 | CTTTTAAAAT.CCGTCTGAAA | T | x | 35004_12_15398_XS2_1 | UnannotatedRegion |
| 1463 | CTTTTAAAAT.CCGTCTGAAA | T | x | 35005_12_13952_XS2_1 | UnannotatedRegion |
| 1463 | CTTTTAAAAT.CCGTCTGAAA | T | x | 35006_12_15317_XS2_1 | UnannotatedRegion |
| 1463 | CTTTTAAAAT.CCGTCTGAAA | T | x | 35007_12_15047_XS2_1 | UnannotatedRegion |
| 1463 | CTTTTAAAAT.CCGTCTGAAA | T | x | 35008_12_15661_XS2_1 | UnannotatedRegion |
| 1463 | CTTTTAAAAT.CCGTCTGAAA | T | x | 35009_12_15657_XS2_1 | UnannotatedRegion |
| 1463 | CTTTTAAAAT.CCGTCTGAAA | T | x | 35010_12_17186_XS2_1 | UnannotatedRegion |
| 1463 | CTTTTAAAAT.CCGTCTGAAA | T | x | 35011_12_17194_XS2_1 | UnannotatedRegion |
| 1463 | CTTTTAAAAT.CCGTCTGAAA | T | x | 35012_12_17009_XS2_1 | UnannotatedRegion |
| 1463 | CTTTTAAAAT.CCGTCTGAAA | T | x | 35013_12_14973_XS2_1 | UnannotatedRegion |
|  |  |  |  |  |  |
| 1468 | GAAACACACT.CATCCGGAAC | A | 2159141 R | 19260_WUE_2594 | UnannotatedRegion |
| 1468 | GAAACACACT.CATCCGGAAC | A | x | 34990_Tchad54_11 | UnannotatedRegion |
| 1468 | GAAACACACT.CATCCGGAAC | A | x | 34991_Tchad78_11 | UnannotatedRegion |
| 1468 | GAAACACACT.CATCCGGAAC | G | x | 34992_Tchad95_11 | UnannotatedRegion |
| 1468 | GAAACACACT.CATCCGGAAC | A | x | 34995_8_2011 | UnannotatedRegion |
| 1468 | GAAACACACT.CATCCGGAAC | A | x | 34996_10_2011 | UnannotatedRegion |
| 1468 | GAAACACACT.CATCCGGAAC | A | x | 34997_39_2011 | UnannotatedRegion |
| 1468 | GAAACACACT.CATCCGGAAC | A | x | 34998_59_2011 | UnannotatedRegion |
| 1468 | GAAACACACT.CATCCGGAAC | A | x | 34999_63_2011 | UnannotatedRegion |
| 1468 | GAAACACACT.CATCCGGAAC | A | x | 35000_75_2011 | UnannotatedRegion |
| 1468 | GAAACACACT.CATCCGGAAC | A | x | 35001_76_2011 | UnannotatedRegion |
| 1468 | GAAACACACT.CATCCGGAAC | A | x | 35002_120_2011 | UnannotatedRegion |
| 1468 | GAAACACACT.CATCCGGAAC | A | x | 35003_403_2011 | UnannotatedRegion |
| 1468 | GAAACACACT.CATCCGGAAC | A | x | 35004_12_15398_XS2_1 | UnannotatedRegion |
| 1468 | GAAACACACT.CATCCGGAAC | A | x | 35005_12_13952_XS2_1 | UnannotatedRegion |
| 1468 | GAAACACACT.CATCCGGAAC | A | x | 35006_12_15317_XS2_1 | UnannotatedRegion |
| 1468 | GAAACACACT.CATCCGGAAC | G | x | 35007_12_15047_XS2_1 | UnannotatedRegion |
| 1468 | GAAACACACT.CATCCGGAAC | G | x | 35008_12_15661_XS2_1 | UnannotatedRegion |
| 1468 | GAAACACACT.CATCCGGAAC | G | x | 35009_12_15657_XS2_1 | UnannotatedRegion |
| 1468 | GAAACACACT.CATCCGGAAC | A | x | 35010_12_17186_XS2_1 | UnannotatedRegion |
| 1468 | GAAACACACT.CATCCGGAAC | A | x | 35011_12_17194_XS2_1 | UnannotatedRegion |
| 1468 | GAAACACACT.CATCCGGAAC | A | x | 35012_12_17009_XS2_1 | UnannotatedRegion |
| 1468 | GAAACACACT.CATCCGGAAC | G | x | 35013_12_14973_XS2_1 | UnannotatedRegion |
|  |  |  |  |  |  |
| 1470 | GAAACCCTTT.AGACGGCATC | C | 1142200 F | 19260_WUE_2594 | UnannotatedRegion |
| 1470 | GAAACCCTTT.AGACGGCATC | T | x | 34990_Tchad54_11 | UnannotatedRegion |
| 1470 | GAAACCCTTT.AGACGGCATC | T | x | 34991_Tchad78_11 | UnannotatedRegion |
| 1470 | GAAACCCTTT.AGACGGCATC | T | x | 34992_Tchad95_11 | UnannotatedRegion |
| 1470 | GAAACCCTTT.AGACGGCATC | T | x | 34994_Tchad106_11 | UnannotatedRegion |
| 1470 | GAAACCCTTT.AGACGGCATC | T | x | 34995_8_2011 | UnannotatedRegion |
| 1470 | GAAACCCTTT.AGACGGCATC | T | x | 34996_10_2011 | UnannotatedRegion |
| 1470 | GAAACCCTTT.AGACGGCATC | T | x | 34997_39_2011 | UnannotatedRegion |
| 1470 | GAAACCCTTT.AGACGGCATC | T | x | 34998_59_2011 | UnannotatedRegion |
| 1470 | GAAACCCTTT.AGACGGCATC | T | x | 34999_63_2011 | UnannotatedRegion |
| 1470 | GAAACCCTTT.AGACGGCATC | T | x | 35000_75_2011 | UnannotatedRegion |
| 1470 | GAAACCCTTT.AGACGGCATC | T | x | 35001_76_2011 | UnannotatedRegion |
| 1470 | GAAACCCTTT.AGACGGCATC | T | x | 35002_120_2011 | UnannotatedRegion |
| 1470 | GAAACCCTTT.AGACGGCATC | T | x | 35003_403_2011 | UnannotatedRegion |
| 1470 | GAAACCCTTT.AGACGGCATC | T | x | 35004_12_15398_XS2_1 | UnannotatedRegion |
| 1470 | GAAACCCTTT.AGACGGCATC | T | x | 35005_12_13952_XS2_1 | UnannotatedRegion |
| 1470 | GAAACCCTTT.AGACGGCATC | T | x | 35006_12_15317_XS2_1 | UnannotatedRegion |
| 1470 | GAAACCCTTT.AGACGGCATC | T | x | 35007_12_15047_XS2_1 | UnannotatedRegion |
| 1470 | GAAACCCTTT.AGACGGCATC | T | x | 35008_12_15661_XS2_1 | UnannotatedRegion |
| 1470 | GAAACCCTTT.AGACGGCATC | T | x | 35009_12_15657_XS2_1 | UnannotatedRegion |
| 1470 | GAAACCCTTT.AGACGGCATC | T | x | 35010_12_17186_XS2_1 | UnannotatedRegion |
| 1470 | GAAACCCTTT.AGACGGCATC | T | x | 35011_12_17194_XS2_1 | UnannotatedRegion |
| 1470 | GAAACCCTTT.AGACGGCATC | T | x | 35012_12_17009_XS2_1 | UnannotatedRegion |
| 1470 | GAAACCCTTT.AGACGGCATC | T | x | 35013_12_14973_XS2_1 | UnannotatedRegion |
|  |  |  |  |  |  |
| 1490 | GAATGCAGCA.CATCCTGACC | T | x | 34990_Tchad54_11 | NotInAnnotatedGenome |
| 1490 | GAATGCAGCA.CATCCTGACC | T | x | 34991_Tchad78_11 | NotInAnnotatedGenome |
| 1490 | GAATGCAGCA.CATCCTGACC | T | x | 34992_Tchad95_11 | NotInAnnotatedGenome |
| 1490 | GAATGCAGCA.CATCCTGACC | T | x | 34994_Tchad106_11 | NotInAnnotatedGenome |
| 1490 | GAATGCAGCA.CATCCTGACC | T | x | 34995_8_2011 | NotInAnnotatedGenome |
| 1490 | GAATGCAGCA.CATCCTGACC | T | x | 34996_10_2011 | NotInAnnotatedGenome |
| 1490 | GAATGCAGCA.CATCCTGACC | T | x | 34997_39_2011 | NotInAnnotatedGenome |
| 1490 | GAATGCAGCA.CATCCTGACC | T | x | 34998_59_2011 | NotInAnnotatedGenome |
| 1490 | GAATGCAGCA.CATCCTGACC | T | x | 34999_63_2011 | NotInAnnotatedGenome |
| 1490 | GAATGCAGCA.CATCCTGACC | T | x | 35000_75_2011 | NotInAnnotatedGenome |
| 1490 | GAATGCAGCA.CATCCTGACC | T | x | 35001_76_2011 | NotInAnnotatedGenome |
| 1490 | GAATGCAGCA.CATCCTGACC | T | x | 35002_120_2011 | NotInAnnotatedGenome |
| 1490 | GAATGCAGCA.CATCCTGACC | T | x | 35003_403_2011 | NotInAnnotatedGenome |
| 1490 | GAATGCAGCA.CATCCTGACC | T | x | 35004_12_15398_XS2_1 | NotInAnnotatedGenome |
| 1490 | GAATGCAGCA.CATCCTGACC | T | x | 35005_12_13952_XS2_1 | NotInAnnotatedGenome |
| 1490 | GAATGCAGCA.CATCCTGACC | T | x | 35006_12_15317_XS2_1 | NotInAnnotatedGenome |
| 1490 | GAATGCAGCA.CATCCTGACC | T | x | 35007_12_15047_XS2_1 | NotInAnnotatedGenome |
| 1490 | GAATGCAGCA.CATCCTGACC | T | x | 35008_12_15661_XS2_1 | NotInAnnotatedGenome |
| 1490 | GAATGCAGCA.CATCCTGACC | T | x | 35009_12_15657_XS2_1 | NotInAnnotatedGenome |
| 1490 | GAATGCAGCA.CATCCTGACC | T | x | 35010_12_17186_XS2_1 | NotInAnnotatedGenome |
| 1490 | GAATGCAGCA.CATCCTGACC | T | x | 35011_12_17194_XS2_1 | NotInAnnotatedGenome |
| 1490 | GAATGCAGCA.CATCCTGACC | G | x | 35012_12_17009_XS2_1 | NotInAnnotatedGenome |
| 1490 | GAATGCAGCA.CATCCTGACC | T | x | 35013_12_14973_XS2_1 | NotInAnnotatedGenome |
|  |  |  |  |  |  |
| 1491 | GAATTACGAA.CGTGTAAAGC | G | x | 34990_Tchad54_11 | NotInAnnotatedGenome |
| 1491 | GAATTACGAA.CGTGTAAAGC | G | x | 34991_Tchad78_11 | NotInAnnotatedGenome |
| 1491 | GAATTACGAA.CGTGTAAAGC | G | x | 34992_Tchad95_11 | NotInAnnotatedGenome |
| 1491 | GAATTACGAA.CGTGTAAAGC | G | x | 34994_Tchad106_11 | NotInAnnotatedGenome |
| 1491 | GAATTACGAA.CGTGTAAAGC | G | x | 34995_8_2011 | NotInAnnotatedGenome |
| 1491 | GAATTACGAA.CGTGTAAAGC | G | x | 34996_10_2011 | NotInAnnotatedGenome |
| 1491 | GAATTACGAA.CGTGTAAAGC | G | x | 34997_39_2011 | NotInAnnotatedGenome |
| 1491 | GAATTACGAA.CGTGTAAAGC | G | x | 34998_59_2011 | NotInAnnotatedGenome |
| 1491 | GAATTACGAA.CGTGTAAAGC | G | x | 34999_63_2011 | NotInAnnotatedGenome |
| 1491 | GAATTACGAA.CGTGTAAAGC | G | x | 35000_75_2011 | NotInAnnotatedGenome |
| 1491 | GAATTACGAA.CGTGTAAAGC | G | x | 35001_76_2011 | NotInAnnotatedGenome |
| 1491 | GAATTACGAA.CGTGTAAAGC | G | x | 35002_120_2011 | NotInAnnotatedGenome |
| 1491 | GAATTACGAA.CGTGTAAAGC | G | x | 35003_403_2011 | NotInAnnotatedGenome |
| 1491 | GAATTACGAA.CGTGTAAAGC | G | x | 35004_12_15398_XS2_1 | NotInAnnotatedGenome |
| 1491 | GAATTACGAA.CGTGTAAAGC | G | x | 35005_12_13952_XS2_1 | NotInAnnotatedGenome |
| 1491 | GAATTACGAA.CGTGTAAAGC | G | x | 35006_12_15317_XS2_1 | NotInAnnotatedGenome |
| 1491 | GAATTACGAA.CGTGTAAAGC | A | x | 35007_12_15047_XS2_1 | NotInAnnotatedGenome |
| 1491 | GAATTACGAA.CGTGTAAAGC | G | x | 35008_12_15661_XS2_1 | NotInAnnotatedGenome |
| 1491 | GAATTACGAA.CGTGTAAAGC | G | x | 35009_12_15657_XS2_1 | NotInAnnotatedGenome |
| 1491 | GAATTACGAA.CGTGTAAAGC | G | x | 35010_12_17186_XS2_1 | NotInAnnotatedGenome |
| 1491 | GAATTACGAA.CGTGTAAAGC | G | x | 35011_12_17194_XS2_1 | NotInAnnotatedGenome |
| 1491 | GAATTACGAA.CGTGTAAAGC | G | x | 35012_12_17009_XS2_1 | NotInAnnotatedGenome |
| 1491 | GAATTACGAA.CGTGTAAAGC | G | x | 35013_12_14973_XS2_1 | NotInAnnotatedGenome |
|  |  |  |  |  |  |
| 1494 | GACCGTTAAC.CCGAAACAAA | A | 509050 R | 19260_WUE_2594 | UnannotatedRegion |
| 1494 | GACCGTTAAC.CCGAAACAAA | G | x | 34990_Tchad54_11 | UnannotatedRegion |
| 1494 | GACCGTTAAC.CCGAAACAAA | G | x | 34991_Tchad78_11 | UnannotatedRegion |
| 1494 | GACCGTTAAC.CCGAAACAAA | G | x | 34992_Tchad95_11 | UnannotatedRegion |
| 1494 | GACCGTTAAC.CCGAAACAAA | G | x | 34994_Tchad106_11 | UnannotatedRegion |
| 1494 | GACCGTTAAC.CCGAAACAAA | G | x | 34995_8_2011 | UnannotatedRegion |
| 1494 | GACCGTTAAC.CCGAAACAAA | G | x | 34996_10_2011 | UnannotatedRegion |
| 1494 | GACCGTTAAC.CCGAAACAAA | G | x | 34997_39_2011 | UnannotatedRegion |
| 1494 | GACCGTTAAC.CCGAAACAAA | G | x | 34998_59_2011 | UnannotatedRegion |
| 1494 | GACCGTTAAC.CCGAAACAAA | G | x | 34999_63_2011 | UnannotatedRegion |
| 1494 | GACCGTTAAC.CCGAAACAAA | G | x | 35000_75_2011 | UnannotatedRegion |
| 1494 | GACCGTTAAC.CCGAAACAAA | G | x | 35001_76_2011 | UnannotatedRegion |
| 1494 | GACCGTTAAC.CCGAAACAAA | G | x | 35002_120_2011 | UnannotatedRegion |
| 1494 | GACCGTTAAC.CCGAAACAAA | G | x | 35003_403_2011 | UnannotatedRegion |
| 1494 | GACCGTTAAC.CCGAAACAAA | G | x | 35004_12_15398_XS2_1 | UnannotatedRegion |
| 1494 | GACCGTTAAC.CCGAAACAAA | G | x | 35005_12_13952_XS2_1 | UnannotatedRegion |
| 1494 | GACCGTTAAC.CCGAAACAAA | G | x | 35006_12_15317_XS2_1 | UnannotatedRegion |
| 1494 | GACCGTTAAC.CCGAAACAAA | G | x | 35007_12_15047_XS2_1 | UnannotatedRegion |
| 1494 | GACCGTTAAC.CCGAAACAAA | G | x | 35008_12_15661_XS2_1 | UnannotatedRegion |
| 1494 | GACCGTTAAC.CCGAAACAAA | G | x | 35009_12_15657_XS2_1 | UnannotatedRegion |
| 1494 | GACCGTTAAC.CCGAAACAAA | G | x | 35010_12_17186_XS2_1 | UnannotatedRegion |
| 1494 | GACCGTTAAC.CCGAAACAAA | G | x | 35011_12_17194_XS2_1 | UnannotatedRegion |
| 1494 | GACCGTTAAC.CCGAAACAAA | G | x | 35012_12_17009_XS2_1 | UnannotatedRegion |
| 1494 | GACCGTTAAC.CCGAAACAAA | G | x | 35013_12_14973_XS2_1 | UnannotatedRegion |
|  |  |  |  |  |  |
| 1500 | GACGGGTCGC.GGAACAATAA | G | x | 34990_Tchad54_11 | NotInAnnotatedGenome |
| 1500 | GACGGGTCGC.GGAACAATAA | A | x | 34991_Tchad78_11 | NotInAnnotatedGenome |
| 1500 | GACGGGTCGC.GGAACAATAA | A | x | 34992_Tchad95_11 | NotInAnnotatedGenome |
| 1500 | GACGGGTCGC.GGAACAATAA | A | x | 34994_Tchad106_11 | NotInAnnotatedGenome |
| 1500 | GACGGGTCGC.GGAACAATAA | G | x | 34995_8_2011 | NotInAnnotatedGenome |
| 1500 | GACGGGTCGC.GGAACAATAA | G | x | 34996_10_2011 | NotInAnnotatedGenome |
| 1500 | GACGGGTCGC.GGAACAATAA | G | x | 34997_39_2011 | NotInAnnotatedGenome |
| 1500 | GACGGGTCGC.GGAACAATAA | G | x | 34998_59_2011 | NotInAnnotatedGenome |
| 1500 | GACGGGTCGC.GGAACAATAA | G | x | 34999_63_2011 | NotInAnnotatedGenome |
| 1500 | GACGGGTCGC.GGAACAATAA | G | x | 35000_75_2011 | NotInAnnotatedGenome |
| 1500 | GACGGGTCGC.GGAACAATAA | G | x | 35001_76_2011 | NotInAnnotatedGenome |
| 1500 | GACGGGTCGC.GGAACAATAA | G | x | 35002_120_2011 | NotInAnnotatedGenome |
| 1500 | GACGGGTCGC.GGAACAATAA | A | x | 35003_403_2011 | NotInAnnotatedGenome |
| 1500 | GACGGGTCGC.GGAACAATAA | G | x | 35004_12_15398_XS2_1 | NotInAnnotatedGenome |
| 1500 | GACGGGTCGC.GGAACAATAA | G | x | 35005_12_13952_XS2_1 | NotInAnnotatedGenome |
| 1500 | GACGGGTCGC.GGAACAATAA | G | x | 35006_12_15317_XS2_1 | NotInAnnotatedGenome |
| 1500 | GACGGGTCGC.GGAACAATAA | A | x | 35007_12_15047_XS2_1 | NotInAnnotatedGenome |
| 1500 | GACGGGTCGC.GGAACAATAA | A | x | 35008_12_15661_XS2_1 | NotInAnnotatedGenome |
| 1500 | GACGGGTCGC.GGAACAATAA | A | x | 35009_12_15657_XS2_1 | NotInAnnotatedGenome |
| 1500 | GACGGGTCGC.GGAACAATAA | G | x | 35010_12_17186_XS2_1 | NotInAnnotatedGenome |
| 1500 | GACGGGTCGC.GGAACAATAA | G | x | 35011_12_17194_XS2_1 | NotInAnnotatedGenome |
| 1500 | GACGGGTCGC.GGAACAATAA | G | x | 35012_12_17009_XS2_1 | NotInAnnotatedGenome |
| 1500 | GACGGGTCGC.GGAACAATAA | A | x | 35013_12_14973_XS2_1 | NotInAnnotatedGenome |
|  |  |  |  |  |  |
| 1504 | GAGAAGGGAA.ATTAACAAAA | G | 692112 F | 19260_WUE_2594 | UnannotatedRegion |
| 1504 | GAGAAGGGAA.ATTAACAAAA | G | x | 34990_Tchad54_11 | UnannotatedRegion |
| 1504 | GAGAAGGGAA.ATTAACAAAA | G | x | 34991_Tchad78_11 | UnannotatedRegion |
| 1504 | GAGAAGGGAA.ATTAACAAAA | G | x | 34992_Tchad95_11 | UnannotatedRegion |
| 1504 | GAGAAGGGAA.ATTAACAAAA | G | x | 34994_Tchad106_11 | UnannotatedRegion |
| 1504 | GAGAAGGGAA.ATTAACAAAA | A | x | 34995_8_2011 | UnannotatedRegion |
| 1504 | GAGAAGGGAA.ATTAACAAAA | A | x | 34996_10_2011 | UnannotatedRegion |
| 1504 | GAGAAGGGAA.ATTAACAAAA | G | x | 34997_39_2011 | UnannotatedRegion |
| 1504 | GAGAAGGGAA.ATTAACAAAA | G | x | 34998_59_2011 | UnannotatedRegion |
| 1504 | GAGAAGGGAA.ATTAACAAAA | G | x | 34999_63_2011 | UnannotatedRegion |
| 1504 | GAGAAGGGAA.ATTAACAAAA | G | x | 35000_75_2011 | UnannotatedRegion |
| 1504 | GAGAAGGGAA.ATTAACAAAA | G | x | 35001_76_2011 | UnannotatedRegion |
| 1504 | GAGAAGGGAA.ATTAACAAAA | G | x | 35002_120_2011 | UnannotatedRegion |
| 1504 | GAGAAGGGAA.ATTAACAAAA | G | x | 35003_403_2011 | UnannotatedRegion |
| 1504 | GAGAAGGGAA.ATTAACAAAA | G | x | 35004_12_15398_XS2_1 | UnannotatedRegion |
| 1504 | GAGAAGGGAA.ATTAACAAAA | G | x | 35005_12_13952_XS2_1 | UnannotatedRegion |
| 1504 | GAGAAGGGAA.ATTAACAAAA | G | x | 35006_12_15317_XS2_1 | UnannotatedRegion |
| 1504 | GAGAAGGGAA.ATTAACAAAA | G | x | 35007_12_15047_XS2_1 | UnannotatedRegion |
| 1504 | GAGAAGGGAA.ATTAACAAAA | G | x | 35008_12_15661_XS2_1 | UnannotatedRegion |
| 1504 | GAGAAGGGAA.ATTAACAAAA | G | x | 35009_12_15657_XS2_1 | UnannotatedRegion |
| 1504 | GAGAAGGGAA.ATTAACAAAA | G | x | 35010_12_17186_XS2_1 | UnannotatedRegion |
| 1504 | GAGAAGGGAA.ATTAACAAAA | G | x | 35011_12_17194_XS2_1 | UnannotatedRegion |
| 1504 | GAGAAGGGAA.ATTAACAAAA | G | x | 35012_12_17009_XS2_1 | UnannotatedRegion |
| 1504 | GAGAAGGGAA.ATTAACAAAA | G | x | 35013_12_14973_XS2_1 | UnannotatedRegion |
|  |  |  |  |  |  |
| 1514 | GATGCAGTAA.GTGGGTTATA | C | 867252 F | 19260_WUE_2594 | UnannotatedRegion |
| 1514 | GATGCAGTAA.GTGGGTTATA | T | x | 34990_Tchad54_11 | UnannotatedRegion |
| 1514 | GATGCAGTAA.GTGGGTTATA | T | x | 34991_Tchad78_11 | UnannotatedRegion |
| 1514 | GATGCAGTAA.GTGGGTTATA | T | x | 34992_Tchad95_11 | UnannotatedRegion |
| 1514 | GATGCAGTAA.GTGGGTTATA | T | x | 34994_Tchad106_11 | UnannotatedRegion |
| 1514 | GATGCAGTAA.GTGGGTTATA | T | x | 34995_8_2011 | UnannotatedRegion |
| 1514 | GATGCAGTAA.GTGGGTTATA | T | x | 34996_10_2011 | UnannotatedRegion |
| 1514 | GATGCAGTAA.GTGGGTTATA | T | x | 34997_39_2011 | UnannotatedRegion |
| 1514 | GATGCAGTAA.GTGGGTTATA | T | x | 34998_59_2011 | UnannotatedRegion |
| 1514 | GATGCAGTAA.GTGGGTTATA | T | x | 34999_63_2011 | UnannotatedRegion |
| 1514 | GATGCAGTAA.GTGGGTTATA | T | x | 35000_75_2011 | UnannotatedRegion |
| 1514 | GATGCAGTAA.GTGGGTTATA | T | x | 35001_76_2011 | UnannotatedRegion |
| 1514 | GATGCAGTAA.GTGGGTTATA | T | x | 35002_120_2011 | UnannotatedRegion |
| 1514 | GATGCAGTAA.GTGGGTTATA | T | x | 35003_403_2011 | UnannotatedRegion |
| 1514 | GATGCAGTAA.GTGGGTTATA | T | x | 35004_12_15398_XS2_1 | UnannotatedRegion |
| 1514 | GATGCAGTAA.GTGGGTTATA | T | x | 35005_12_13952_XS2_1 | UnannotatedRegion |
| 1514 | GATGCAGTAA.GTGGGTTATA | T | x | 35006_12_15317_XS2_1 | UnannotatedRegion |
| 1514 | GATGCAGTAA.GTGGGTTATA | T | x | 35007_12_15047_XS2_1 | UnannotatedRegion |
| 1514 | GATGCAGTAA.GTGGGTTATA | T | x | 35008_12_15661_XS2_1 | UnannotatedRegion |
| 1514 | GATGCAGTAA.GTGGGTTATA | T | x | 35009_12_15657_XS2_1 | UnannotatedRegion |
| 1514 | GATGCAGTAA.GTGGGTTATA | T | x | 35010_12_17186_XS2_1 | UnannotatedRegion |
| 1514 | GATGCAGTAA.GTGGGTTATA | T | x | 35011_12_17194_XS2_1 | UnannotatedRegion |
| 1514 | GATGCAGTAA.GTGGGTTATA | T | x | 35012_12_17009_XS2_1 | UnannotatedRegion |
| 1514 | GATGCAGTAA.GTGGGTTATA | T | x | 35013_12_14973_XS2_1 | UnannotatedRegion |
|  |  |  |  |  |  |
| 1519 | GATGTTGTGG.GGTTTCAAAC | C | 253859 R | 19260_WUE_2594 | UnannotatedRegion |
| 1519 | GATGTTGTGG.GGTTTCAAAC | T | x | 34990_Tchad54_11 | UnannotatedRegion |
| 1519 | GATGTTGTGG.GGTTTCAAAC | T | x | 34991_Tchad78_11 | UnannotatedRegion |
| 1519 | GATGTTGTGG.GGTTTCAAAC | T | x | 34992_Tchad95_11 | UnannotatedRegion |
| 1519 | GATGTTGTGG.GGTTTCAAAC | T | x | 34994_Tchad106_11 | UnannotatedRegion |
| 1519 | GATGTTGTGG.GGTTTCAAAC | T | x | 34995_8_2011 | UnannotatedRegion |
| 1519 | GATGTTGTGG.GGTTTCAAAC | T | x | 34996_10_2011 | UnannotatedRegion |
| 1519 | GATGTTGTGG.GGTTTCAAAC | T | x | 34997_39_2011 | UnannotatedRegion |
| 1519 | GATGTTGTGG.GGTTTCAAAC | T | x | 34998_59_2011 | UnannotatedRegion |
| 1519 | GATGTTGTGG.GGTTTCAAAC | T | x | 34999_63_2011 | UnannotatedRegion |
| 1519 | GATGTTGTGG.GGTTTCAAAC | T | x | 35000_75_2011 | UnannotatedRegion |
| 1519 | GATGTTGTGG.GGTTTCAAAC | T | x | 35001_76_2011 | UnannotatedRegion |
| 1519 | GATGTTGTGG.GGTTTCAAAC | T | x | 35002_120_2011 | UnannotatedRegion |
| 1519 | GATGTTGTGG.GGTTTCAAAC | T | x | 35003_403_2011 | UnannotatedRegion |
| 1519 | GATGTTGTGG.GGTTTCAAAC | T | x | 35004_12_15398_XS2_1 | UnannotatedRegion |
| 1519 | GATGTTGTGG.GGTTTCAAAC | T | x | 35005_12_13952_XS2_1 | UnannotatedRegion |
| 1519 | GATGTTGTGG.GGTTTCAAAC | T | x | 35006_12_15317_XS2_1 | UnannotatedRegion |
| 1519 | GATGTTGTGG.GGTTTCAAAC | T | x | 35007_12_15047_XS2_1 | UnannotatedRegion |
| 1519 | GATGTTGTGG.GGTTTCAAAC | T | x | 35008_12_15661_XS2_1 | UnannotatedRegion |
| 1519 | GATGTTGTGG.GGTTTCAAAC | T | x | 35009_12_15657_XS2_1 | UnannotatedRegion |
| 1519 | GATGTTGTGG.GGTTTCAAAC | T | x | 35010_12_17186_XS2_1 | UnannotatedRegion |
| 1519 | GATGTTGTGG.GGTTTCAAAC | T | x | 35011_12_17194_XS2_1 | UnannotatedRegion |
| 1519 | GATGTTGTGG.GGTTTCAAAC | T | x | 35012_12_17009_XS2_1 | UnannotatedRegion |
| 1519 | GATGTTGTGG.GGTTTCAAAC | T | x | 35013_12_14973_XS2_1 | UnannotatedRegion |
|  |  |  |  |  |  |
| 1521 | GATTATTATT.TCAAAACGCC | T | x | 34990_Tchad54_11 | NotInAnnotatedGenome |
| 1521 | GATTATTATT.TCAAAACGCC | T | x | 34991_Tchad78_11 | NotInAnnotatedGenome |
| 1521 | GATTATTATT.TCAAAACGCC | T | x | 34992_Tchad95_11 | NotInAnnotatedGenome |
| 1521 | GATTATTATT.TCAAAACGCC | T | x | 34994_Tchad106_11 | NotInAnnotatedGenome |
| 1521 | GATTATTATT.TCAAAACGCC | T | x | 34995_8_2011 | NotInAnnotatedGenome |
| 1521 | GATTATTATT.TCAAAACGCC | T | x | 34996_10_2011 | NotInAnnotatedGenome |
| 1521 | GATTATTATT.TCAAAACGCC | T | x | 34997_39_2011 | NotInAnnotatedGenome |
| 1521 | GATTATTATT.TCAAAACGCC | T | x | 34998_59_2011 | NotInAnnotatedGenome |
| 1521 | GATTATTATT.TCAAAACGCC | T | x | 34999_63_2011 | NotInAnnotatedGenome |
| 1521 | GATTATTATT.TCAAAACGCC | T | x | 35000_75_2011 | NotInAnnotatedGenome |
| 1521 | GATTATTATT.TCAAAACGCC | T | x | 35001_76_2011 | NotInAnnotatedGenome |
| 1521 | GATTATTATT.TCAAAACGCC | T | x | 35002_120_2011 | NotInAnnotatedGenome |
| 1521 | GATTATTATT.TCAAAACGCC | T | x | 35003_403_2011 | NotInAnnotatedGenome |
| 1521 | GATTATTATT.TCAAAACGCC | T | x | 35004_12_15398_XS2_1 | NotInAnnotatedGenome |
| 1521 | GATTATTATT.TCAAAACGCC | T | x | 35005_12_13952_XS2_1 | NotInAnnotatedGenome |
| 1521 | GATTATTATT.TCAAAACGCC | T | x | 35006_12_15317_XS2_1 | NotInAnnotatedGenome |
| 1521 | GATTATTATT.TCAAAACGCC | A | x | 35007_12_15047_XS2_1 | NotInAnnotatedGenome |
| 1521 | GATTATTATT.TCAAAACGCC | T | x | 35008_12_15661_XS2_1 | NotInAnnotatedGenome |
| 1521 | GATTATTATT.TCAAAACGCC | T | x | 35009_12_15657_XS2_1 | NotInAnnotatedGenome |
| 1521 | GATTATTATT.TCAAAACGCC | T | x | 35010_12_17186_XS2_1 | NotInAnnotatedGenome |
| 1521 | GATTATTATT.TCAAAACGCC | T | x | 35011_12_17194_XS2_1 | NotInAnnotatedGenome |
| 1521 | GATTATTATT.TCAAAACGCC | T | x | 35012_12_17009_XS2_1 | NotInAnnotatedGenome |
| 1521 | GATTATTATT.TCAAAACGCC | T | x | 35013_12_14973_XS2_1 | NotInAnnotatedGenome |
|  |  |  |  |  |  |
| 1554 | GCATAAACAG.GAATTTATGA | G | 1141633 R | 19260_WUE_2594 | UnannotatedRegion |
| 1554 | GCATAAACAG.GAATTTATGA | A | x | 34990_Tchad54_11 | UnannotatedRegion |
| 1554 | GCATAAACAG.GAATTTATGA | A | x | 34991_Tchad78_11 | UnannotatedRegion |
| 1554 | GCATAAACAG.GAATTTATGA | A | x | 34992_Tchad95_11 | UnannotatedRegion |
| 1554 | GCATAAACAG.GAATTTATGA | A | x | 34994_Tchad106_11 | UnannotatedRegion |
| 1554 | GCATAAACAG.GAATTTATGA | A | x | 34995_8_2011 | UnannotatedRegion |
| 1554 | GCATAAACAG.GAATTTATGA | A | x | 34996_10_2011 | UnannotatedRegion |
| 1554 | GCATAAACAG.GAATTTATGA | A | x | 34997_39_2011 | UnannotatedRegion |
| 1554 | GCATAAACAG.GAATTTATGA | A | x | 34998_59_2011 | UnannotatedRegion |
| 1554 | GCATAAACAG.GAATTTATGA | A | x | 34999_63_2011 | UnannotatedRegion |
| 1554 | GCATAAACAG.GAATTTATGA | A | x | 35000_75_2011 | UnannotatedRegion |
| 1554 | GCATAAACAG.GAATTTATGA | A | x | 35001_76_2011 | UnannotatedRegion |
| 1554 | GCATAAACAG.GAATTTATGA | A | x | 35002_120_2011 | UnannotatedRegion |
| 1554 | GCATAAACAG.GAATTTATGA | A | x | 35003_403_2011 | UnannotatedRegion |
| 1554 | GCATAAACAG.GAATTTATGA | A | x | 35004_12_15398_XS2_1 | UnannotatedRegion |
| 1554 | GCATAAACAG.GAATTTATGA | A | x | 35005_12_13952_XS2_1 | UnannotatedRegion |
| 1554 | GCATAAACAG.GAATTTATGA | A | x | 35006_12_15317_XS2_1 | UnannotatedRegion |
| 1554 | GCATAAACAG.GAATTTATGA | A | x | 35007_12_15047_XS2_1 | UnannotatedRegion |
| 1554 | GCATAAACAG.GAATTTATGA | A | x | 35008_12_15661_XS2_1 | UnannotatedRegion |
| 1554 | GCATAAACAG.GAATTTATGA | A | x | 35009_12_15657_XS2_1 | UnannotatedRegion |
| 1554 | GCATAAACAG.GAATTTATGA | A | x | 35010_12_17186_XS2_1 | UnannotatedRegion |
| 1554 | GCATAAACAG.GAATTTATGA | A | x | 35011_12_17194_XS2_1 | UnannotatedRegion |
| 1554 | GCATAAACAG.GAATTTATGA | A | x | 35012_12_17009_XS2_1 | UnannotatedRegion |
| 1554 | GCATAAACAG.GAATTTATGA | A | x | 35013_12_14973_XS2_1 | UnannotatedRegion |
|  |  |  |  |  |  |
| 1556 | GCATAACCGC.GGTTGGAACA | C | 1085026 F | 19260_WUE_2594 | UnannotatedRegion |
| 1556 | GCATAACCGC.GGTTGGAACA | A | x | 34990_Tchad54_11 | UnannotatedRegion |
| 1556 | GCATAACCGC.GGTTGGAACA | A | x | 34991_Tchad78_11 | UnannotatedRegion |
| 1556 | GCATAACCGC.GGTTGGAACA | A | x | 34992_Tchad95_11 | UnannotatedRegion |
| 1556 | GCATAACCGC.GGTTGGAACA | A | x | 34994_Tchad106_11 | UnannotatedRegion |
| 1556 | GCATAACCGC.GGTTGGAACA | C | x | 34995_8_2011 | UnannotatedRegion |
| 1556 | GCATAACCGC.GGTTGGAACA | C | x | 34996_10_2011 | UnannotatedRegion |
| 1556 | GCATAACCGC.GGTTGGAACA | A | x | 34997_39_2011 | UnannotatedRegion |
| 1556 | GCATAACCGC.GGTTGGAACA | A | x | 34998_59_2011 | UnannotatedRegion |
| 1556 | GCATAACCGC.GGTTGGAACA | A | x | 34999_63_2011 | UnannotatedRegion |
| 1556 | GCATAACCGC.GGTTGGAACA | A | x | 35000_75_2011 | UnannotatedRegion |
| 1556 | GCATAACCGC.GGTTGGAACA | A | x | 35001_76_2011 | UnannotatedRegion |
| 1556 | GCATAACCGC.GGTTGGAACA | A | x | 35002_120_2011 | UnannotatedRegion |
| 1556 | GCATAACCGC.GGTTGGAACA | A | x | 35003_403_2011 | UnannotatedRegion |
| 1556 | GCATAACCGC.GGTTGGAACA | A | x | 35004_12_15398_XS2_1 | UnannotatedRegion |
| 1556 | GCATAACCGC.GGTTGGAACA | A | x | 35005_12_13952_XS2_1 | UnannotatedRegion |
| 1556 | GCATAACCGC.GGTTGGAACA | A | x | 35006_12_15317_XS2_1 | UnannotatedRegion |
| 1556 | GCATAACCGC.GGTTGGAACA | A | x | 35007_12_15047_XS2_1 | UnannotatedRegion |
| 1556 | GCATAACCGC.GGTTGGAACA | A | x | 35008_12_15661_XS2_1 | UnannotatedRegion |
| 1556 | GCATAACCGC.GGTTGGAACA | A | x | 35009_12_15657_XS2_1 | UnannotatedRegion |
| 1556 | GCATAACCGC.GGTTGGAACA | A | x | 35010_12_17186_XS2_1 | UnannotatedRegion |
| 1556 | GCATAACCGC.GGTTGGAACA | A | x | 35011_12_17194_XS2_1 | UnannotatedRegion |
| 1556 | GCATAACCGC.GGTTGGAACA | A | x | 35012_12_17009_XS2_1 | UnannotatedRegion |
| 1556 | GCATAACCGC.GGTTGGAACA | A | x | 35013_12_14973_XS2_1 | UnannotatedRegion |
|  |  |  |  |  |  |
| 1557 | GCATACCGGC.ATGCCGATAC | A | 1147062 R | 19260_WUE_2594 | UnannotatedRegion |
| 1557 | GCATACCGGC.ATGCCGATAC | G | x | 34990_Tchad54_11 | UnannotatedRegion |
| 1557 | GCATACCGGC.ATGCCGATAC | G | x | 34991_Tchad78_11 | UnannotatedRegion |
| 1557 | GCATACCGGC.ATGCCGATAC | G | x | 34992_Tchad95_11 | UnannotatedRegion |
| 1557 | GCATACCGGC.ATGCCGATAC | G | x | 34994_Tchad106_11 | UnannotatedRegion |
| 1557 | GCATACCGGC.ATGCCGATAC | G | x | 34995_8_2011 | UnannotatedRegion |
| 1557 | GCATACCGGC.ATGCCGATAC | G | x | 34996_10_2011 | UnannotatedRegion |
| 1557 | GCATACCGGC.ATGCCGATAC | G | x | 34997_39_2011 | UnannotatedRegion |
| 1557 | GCATACCGGC.ATGCCGATAC | G | x | 34998_59_2011 | UnannotatedRegion |
| 1557 | GCATACCGGC.ATGCCGATAC | G | x | 34999_63_2011 | UnannotatedRegion |
| 1557 | GCATACCGGC.ATGCCGATAC | G | x | 35000_75_2011 | UnannotatedRegion |
| 1557 | GCATACCGGC.ATGCCGATAC | G | x | 35001_76_2011 | UnannotatedRegion |
| 1557 | GCATACCGGC.ATGCCGATAC | G | x | 35002_120_2011 | UnannotatedRegion |
| 1557 | GCATACCGGC.ATGCCGATAC | G | x | 35003_403_2011 | UnannotatedRegion |
| 1557 | GCATACCGGC.ATGCCGATAC | G | x | 35004_12_15398_XS2_1 | UnannotatedRegion |
| 1557 | GCATACCGGC.ATGCCGATAC | G | x | 35005_12_13952_XS2_1 | UnannotatedRegion |
| 1557 | GCATACCGGC.ATGCCGATAC | G | x | 35006_12_15317_XS2_1 | UnannotatedRegion |
| 1557 | GCATACCGGC.ATGCCGATAC | G | x | 35007_12_15047_XS2_1 | UnannotatedRegion |
| 1557 | GCATACCGGC.ATGCCGATAC | G | x | 35008_12_15661_XS2_1 | UnannotatedRegion |
| 1557 | GCATACCGGC.ATGCCGATAC | G | x | 35009_12_15657_XS2_1 | UnannotatedRegion |
| 1557 | GCATACCGGC.ATGCCGATAC | G | x | 35010_12_17186_XS2_1 | UnannotatedRegion |
| 1557 | GCATACCGGC.ATGCCGATAC | G | x | 35011_12_17194_XS2_1 | UnannotatedRegion |
| 1557 | GCATACCGGC.ATGCCGATAC | G | x | 35012_12_17009_XS2_1 | UnannotatedRegion |
| 1557 | GCATACCGGC.ATGCCGATAC | G | x | 35013_12_14973_XS2_1 | UnannotatedRegion |
|  |  |  |  |  |  |
| 1560 | GCATATGCGC.CCTACCAGCC | T | 2109393 F | 19260_WUE_2594 | UnannotatedRegion |
| 1560 | GCATATGCGC.CCTACCAGCC | C | x | 34990_Tchad54_11 | UnannotatedRegion |
| 1560 | GCATATGCGC.CCTACCAGCC | C | x | 34991_Tchad78_11 | UnannotatedRegion |
| 1560 | GCATATGCGC.CCTACCAGCC | C | x | 34992_Tchad95_11 | UnannotatedRegion |
| 1560 | GCATATGCGC.CCTACCAGCC | C | x | 34994_Tchad106_11 | UnannotatedRegion |
| 1560 | GCATATGCGC.CCTACCAGCC | C | x | 34995_8_2011 | UnannotatedRegion |
| 1560 | GCATATGCGC.CCTACCAGCC | C | x | 34996_10_2011 | UnannotatedRegion |
| 1560 | GCATATGCGC.CCTACCAGCC | C | x | 34997_39_2011 | UnannotatedRegion |
| 1560 | GCATATGCGC.CCTACCAGCC | C | x | 34998_59_2011 | UnannotatedRegion |
| 1560 | GCATATGCGC.CCTACCAGCC | C | x | 34999_63_2011 | UnannotatedRegion |
| 1560 | GCATATGCGC.CCTACCAGCC | C | x | 35000_75_2011 | UnannotatedRegion |
| 1560 | GCATATGCGC.CCTACCAGCC | C | x | 35001_76_2011 | UnannotatedRegion |
| 1560 | GCATATGCGC.CCTACCAGCC | C | x | 35002_120_2011 | UnannotatedRegion |
| 1560 | GCATATGCGC.CCTACCAGCC | C | x | 35003_403_2011 | UnannotatedRegion |
| 1560 | GCATATGCGC.CCTACCAGCC | C | x | 35004_12_15398_XS2_1 | UnannotatedRegion |
| 1560 | GCATATGCGC.CCTACCAGCC | C | x | 35005_12_13952_XS2_1 | UnannotatedRegion |
| 1560 | GCATATGCGC.CCTACCAGCC | C | x | 35006_12_15317_XS2_1 | UnannotatedRegion |
| 1560 | GCATATGCGC.CCTACCAGCC | C | x | 35007_12_15047_XS2_1 | UnannotatedRegion |
| 1560 | GCATATGCGC.CCTACCAGCC | C | x | 35008_12_15661_XS2_1 | UnannotatedRegion |
| 1560 | GCATATGCGC.CCTACCAGCC | C | x | 35009_12_15657_XS2_1 | UnannotatedRegion |
| 1560 | GCATATGCGC.CCTACCAGCC | C | x | 35010_12_17186_XS2_1 | UnannotatedRegion |
| 1560 | GCATATGCGC.CCTACCAGCC | C | x | 35011_12_17194_XS2_1 | UnannotatedRegion |
| 1560 | GCATATGCGC.CCTACCAGCC | C | x | 35012_12_17009_XS2_1 | UnannotatedRegion |
| 1560 | GCATATGCGC.CCTACCAGCC | C | x | 35013_12_14973_XS2_1 | UnannotatedRegion |
|  |  |  |  |  |  |
| 1577 | GCCCCATGCC.TTTTTTTACA | A | 956185 R | 19260_WUE_2594 | UnannotatedRegion |
| 1577 | GCCCCATGCC.TTTTTTTACA | G | x | 34990_Tchad54_11 | UnannotatedRegion |
| 1577 | GCCCCATGCC.TTTTTTTACA | G | x | 34991_Tchad78_11 | UnannotatedRegion |
| 1577 | GCCCCATGCC.TTTTTTTACA | G | x | 34992_Tchad95_11 | UnannotatedRegion |
| 1577 | GCCCCATGCC.TTTTTTTACA | G | x | 34994_Tchad106_11 | UnannotatedRegion |
| 1577 | GCCCCATGCC.TTTTTTTACA | G | x | 34995_8_2011 | UnannotatedRegion |
| 1577 | GCCCCATGCC.TTTTTTTACA | G | x | 34996_10_2011 | UnannotatedRegion |
| 1577 | GCCCCATGCC.TTTTTTTACA | G | x | 34997_39_2011 | UnannotatedRegion |
| 1577 | GCCCCATGCC.TTTTTTTACA | G | x | 34998_59_2011 | UnannotatedRegion |
| 1577 | GCCCCATGCC.TTTTTTTACA | G | x | 34999_63_2011 | UnannotatedRegion |
| 1577 | GCCCCATGCC.TTTTTTTACA | G | x | 35000_75_2011 | UnannotatedRegion |
| 1577 | GCCCCATGCC.TTTTTTTACA | G | x | 35001_76_2011 | UnannotatedRegion |
| 1577 | GCCCCATGCC.TTTTTTTACA | G | x | 35002_120_2011 | UnannotatedRegion |
| 1577 | GCCCCATGCC.TTTTTTTACA | G | x | 35003_403_2011 | UnannotatedRegion |
| 1577 | GCCCCATGCC.TTTTTTTACA | G | x | 35004_12_15398_XS2_1 | UnannotatedRegion |
| 1577 | GCCCCATGCC.TTTTTTTACA | G | x | 35005_12_13952_XS2_1 | UnannotatedRegion |
| 1577 | GCCCCATGCC.TTTTTTTACA | G | x | 35006_12_15317_XS2_1 | UnannotatedRegion |
| 1577 | GCCCCATGCC.TTTTTTTACA | G | x | 35007_12_15047_XS2_1 | UnannotatedRegion |
| 1577 | GCCCCATGCC.TTTTTTTACA | G | x | 35008_12_15661_XS2_1 | UnannotatedRegion |
| 1577 | GCCCCATGCC.TTTTTTTACA | G | x | 35009_12_15657_XS2_1 | UnannotatedRegion |
| 1577 | GCCCCATGCC.TTTTTTTACA | G | x | 35010_12_17186_XS2_1 | UnannotatedRegion |
| 1577 | GCCCCATGCC.TTTTTTTACA | G | x | 35011_12_17194_XS2_1 | UnannotatedRegion |
| 1577 | GCCCCATGCC.TTTTTTTACA | G | x | 35012_12_17009_XS2_1 | UnannotatedRegion |
| 1577 | GCCCCATGCC.TTTTTTTACA | G | x | 35013_12_14973_XS2_1 | UnannotatedRegion |
|  |  |  |  |  |  |
| 1640 | GCGGTTCGGC.TTCAAATGCC | G | 2073262 R | 19260_WUE_2594 | UnannotatedRegion |
| 1640 | GCGGTTCGGC.TTCAAATGCC | A | x | 34990_Tchad54_11 | UnannotatedRegion |
| 1640 | GCGGTTCGGC.TTCAAATGCC | A | x | 34991_Tchad78_11 | UnannotatedRegion |
| 1640 | GCGGTTCGGC.TTCAAATGCC | A | x | 34992_Tchad95_11 | UnannotatedRegion |
| 1640 | GCGGTTCGGC.TTCAAATGCC | A | x | 34994_Tchad106_11 | UnannotatedRegion |
| 1640 | GCGGTTCGGC.TTCAAATGCC | A | x | 34995_8_2011 | UnannotatedRegion |
| 1640 | GCGGTTCGGC.TTCAAATGCC | A | x | 34996_10_2011 | UnannotatedRegion |
| 1640 | GCGGTTCGGC.TTCAAATGCC | A | x | 34997_39_2011 | UnannotatedRegion |
| 1640 | GCGGTTCGGC.TTCAAATGCC | A | x | 34998_59_2011 | UnannotatedRegion |
| 1640 | GCGGTTCGGC.TTCAAATGCC | A | x | 34999_63_2011 | UnannotatedRegion |
| 1640 | GCGGTTCGGC.TTCAAATGCC | A | x | 35000_75_2011 | UnannotatedRegion |
| 1640 | GCGGTTCGGC.TTCAAATGCC | A | x | 35001_76_2011 | UnannotatedRegion |
| 1640 | GCGGTTCGGC.TTCAAATGCC | A | x | 35002_120_2011 | UnannotatedRegion |
| 1640 | GCGGTTCGGC.TTCAAATGCC | A | x | 35003_403_2011 | UnannotatedRegion |
| 1640 | GCGGTTCGGC.TTCAAATGCC | A | x | 35004_12_15398_XS2_1 | UnannotatedRegion |
| 1640 | GCGGTTCGGC.TTCAAATGCC | A | x | 35005_12_13952_XS2_1 | UnannotatedRegion |
| 1640 | GCGGTTCGGC.TTCAAATGCC | A | x | 35006_12_15317_XS2_1 | UnannotatedRegion |
| 1640 | GCGGTTCGGC.TTCAAATGCC | A | x | 35007_12_15047_XS2_1 | UnannotatedRegion |
| 1640 | GCGGTTCGGC.TTCAAATGCC | A | x | 35008_12_15661_XS2_1 | UnannotatedRegion |
| 1640 | GCGGTTCGGC.TTCAAATGCC | A | x | 35009_12_15657_XS2_1 | UnannotatedRegion |
| 1640 | GCGGTTCGGC.TTCAAATGCC | A | x | 35010_12_17186_XS2_1 | UnannotatedRegion |
| 1640 | GCGGTTCGGC.TTCAAATGCC | A | x | 35011_12_17194_XS2_1 | UnannotatedRegion |
| 1640 | GCGGTTCGGC.TTCAAATGCC | A | x | 35012_12_17009_XS2_1 | UnannotatedRegion |
| 1640 | GCGGTTCGGC.TTCAAATGCC | A | x | 35013_12_14973_XS2_1 | UnannotatedRegion |
|  |  |  |  |  |  |
| 1667 | GCTTCTTCCT.GCGCGAAGCC | G | x | 34990_Tchad54_11 | NotInAnnotatedGenome |
| 1667 | GCTTCTTCCT.GCGCGAAGCC | A | x | 34991_Tchad78_11 | NotInAnnotatedGenome |
| 1667 | GCTTCTTCCT.GCGCGAAGCC | A | x | 34992_Tchad95_11 | NotInAnnotatedGenome |
| 1667 | GCTTCTTCCT.GCGCGAAGCC | A | x | 34994_Tchad106_11 | NotInAnnotatedGenome |
| 1667 | GCTTCTTCCT.GCGCGAAGCC | G | x | 34995_8_2011 | NotInAnnotatedGenome |
| 1667 | GCTTCTTCCT.GCGCGAAGCC | G | x | 34996_10_2011 | NotInAnnotatedGenome |
| 1667 | GCTTCTTCCT.GCGCGAAGCC | G | x | 34997_39_2011 | NotInAnnotatedGenome |
| 1667 | GCTTCTTCCT.GCGCGAAGCC | G | x | 34998_59_2011 | NotInAnnotatedGenome |
| 1667 | GCTTCTTCCT.GCGCGAAGCC | G | x | 34999_63_2011 | NotInAnnotatedGenome |
| 1667 | GCTTCTTCCT.GCGCGAAGCC | G | x | 35000_75_2011 | NotInAnnotatedGenome |
| 1667 | GCTTCTTCCT.GCGCGAAGCC | G | x | 35001_76_2011 | NotInAnnotatedGenome |
| 1667 | GCTTCTTCCT.GCGCGAAGCC | G | x | 35002_120_2011 | NotInAnnotatedGenome |
| 1667 | GCTTCTTCCT.GCGCGAAGCC | A | x | 35003_403_2011 | NotInAnnotatedGenome |
| 1667 | GCTTCTTCCT.GCGCGAAGCC | G | x | 35004_12_15398_XS2_1 | NotInAnnotatedGenome |
| 1667 | GCTTCTTCCT.GCGCGAAGCC | G | x | 35005_12_13952_XS2_1 | NotInAnnotatedGenome |
| 1667 | GCTTCTTCCT.GCGCGAAGCC | G | x | 35006_12_15317_XS2_1 | NotInAnnotatedGenome |
| 1667 | GCTTCTTCCT.GCGCGAAGCC | A | x | 35007_12_15047_XS2_1 | NotInAnnotatedGenome |
| 1667 | GCTTCTTCCT.GCGCGAAGCC | A | x | 35008_12_15661_XS2_1 | NotInAnnotatedGenome |
| 1667 | GCTTCTTCCT.GCGCGAAGCC | A | x | 35009_12_15657_XS2_1 | NotInAnnotatedGenome |
| 1667 | GCTTCTTCCT.GCGCGAAGCC | G | x | 35010_12_17186_XS2_1 | NotInAnnotatedGenome |
| 1667 | GCTTCTTCCT.GCGCGAAGCC | G | x | 35011_12_17194_XS2_1 | NotInAnnotatedGenome |
| 1667 | GCTTCTTCCT.GCGCGAAGCC | G | x | 35012_12_17009_XS2_1 | NotInAnnotatedGenome |
| 1667 | GCTTCTTCCT.GCGCGAAGCC | A | x | 35013_12_14973_XS2_1 | NotInAnnotatedGenome |
|  |  |  |  |  |  |
| 1680 | GGAAGGAGCG.TGCCGTCTGA | G | 1731033 F | 19260_WUE_2594 | UnannotatedRegion |
| 1680 | GGAAGGAGCG.TGCCGTCTGA | G | x | 34990_Tchad54_11 | UnannotatedRegion |
| 1680 | GGAAGGAGCG.TGCCGTCTGA | G | x | 34991_Tchad78_11 | UnannotatedRegion |
| 1680 | GGAAGGAGCG.TGCCGTCTGA | G | x | 34992_Tchad95_11 | UnannotatedRegion |
| 1680 | GGAAGGAGCG.TGCCGTCTGA | G | x | 34994_Tchad106_11 | UnannotatedRegion |
| 1680 | GGAAGGAGCG.TGCCGTCTGA | G | x | 34995_8_2011 | UnannotatedRegion |
| 1680 | GGAAGGAGCG.TGCCGTCTGA | G | x | 34996_10_2011 | UnannotatedRegion |
| 1680 | GGAAGGAGCG.TGCCGTCTGA | G | x | 34997_39_2011 | UnannotatedRegion |
| 1680 | GGAAGGAGCG.TGCCGTCTGA | G | x | 34998_59_2011 | UnannotatedRegion |
| 1680 | GGAAGGAGCG.TGCCGTCTGA | G | x | 34999_63_2011 | UnannotatedRegion |
| 1680 | GGAAGGAGCG.TGCCGTCTGA | G | x | 35000_75_2011 | UnannotatedRegion |
| 1680 | GGAAGGAGCG.TGCCGTCTGA | G | x | 35001_76_2011 | UnannotatedRegion |
| 1680 | GGAAGGAGCG.TGCCGTCTGA | G | x | 35002_120_2011 | UnannotatedRegion |
| 1680 | GGAAGGAGCG.TGCCGTCTGA | G | x | 35003_403_2011 | UnannotatedRegion |
| 1680 | GGAAGGAGCG.TGCCGTCTGA | G | x | 35004_12_15398_XS2_1 | UnannotatedRegion |
| 1680 | GGAAGGAGCG.TGCCGTCTGA | G | x | 35005_12_13952_XS2_1 | UnannotatedRegion |
| 1680 | GGAAGGAGCG.TGCCGTCTGA | G | x | 35006_12_15317_XS2_1 | UnannotatedRegion |
| 1680 | GGAAGGAGCG.TGCCGTCTGA | A | x | 35007_12_15047_XS2_1 | UnannotatedRegion |
| 1680 | GGAAGGAGCG.TGCCGTCTGA | G | x | 35008_12_15661_XS2_1 | UnannotatedRegion |
| 1680 | GGAAGGAGCG.TGCCGTCTGA | G | x | 35009_12_15657_XS2_1 | UnannotatedRegion |
| 1680 | GGAAGGAGCG.TGCCGTCTGA | G | x | 35010_12_17186_XS2_1 | UnannotatedRegion |
| 1680 | GGAAGGAGCG.TGCCGTCTGA | G | x | 35011_12_17194_XS2_1 | UnannotatedRegion |
| 1680 | GGAAGGAGCG.TGCCGTCTGA | G | x | 35012_12_17009_XS2_1 | UnannotatedRegion |
| 1680 | GGAAGGAGCG.TGCCGTCTGA | G | x | 35013_12_14973_XS2_1 | UnannotatedRegion |
|  |  |  |  |  |  |
| 1705 | GGCGTTATCC.CCGAGTGTCA | G | 2159169 F | 19260_WUE_2594 | UnannotatedRegion |
| 1705 | GGCGTTATCC.CCGAGTGTCA | G | x | 34990_Tchad54_11 | UnannotatedRegion |
| 1705 | GGCGTTATCC.CCGAGTGTCA | G | x | 34991_Tchad78_11 | UnannotatedRegion |
| 1705 | GGCGTTATCC.CCGAGTGTCA | A | x | 34992_Tchad95_11 | UnannotatedRegion |
| 1705 | GGCGTTATCC.CCGAGTGTCA | G | x | 34995_8_2011 | UnannotatedRegion |
| 1705 | GGCGTTATCC.CCGAGTGTCA | G | x | 34996_10_2011 | UnannotatedRegion |
| 1705 | GGCGTTATCC.CCGAGTGTCA | G | x | 34997_39_2011 | UnannotatedRegion |
| 1705 | GGCGTTATCC.CCGAGTGTCA | G | x | 34998_59_2011 | UnannotatedRegion |
| 1705 | GGCGTTATCC.CCGAGTGTCA | G | x | 34999_63_2011 | UnannotatedRegion |
| 1705 | GGCGTTATCC.CCGAGTGTCA | G | x | 35000_75_2011 | UnannotatedRegion |
| 1705 | GGCGTTATCC.CCGAGTGTCA | G | x | 35001_76_2011 | UnannotatedRegion |
| 1705 | GGCGTTATCC.CCGAGTGTCA | G | x | 35002_120_2011 | UnannotatedRegion |
| 1705 | GGCGTTATCC.CCGAGTGTCA | G | x | 35003_403_2011 | UnannotatedRegion |
| 1705 | GGCGTTATCC.CCGAGTGTCA | G | x | 35004_12_15398_XS2_1 | UnannotatedRegion |
| 1705 | GGCGTTATCC.CCGAGTGTCA | G | x | 35005_12_13952_XS2_1 | UnannotatedRegion |
| 1705 | GGCGTTATCC.CCGAGTGTCA | G | x | 35006_12_15317_XS2_1 | UnannotatedRegion |
| 1705 | GGCGTTATCC.CCGAGTGTCA | A | x | 35007_12_15047_XS2_1 | UnannotatedRegion |
| 1705 | GGCGTTATCC.CCGAGTGTCA | A | x | 35008_12_15661_XS2_1 | UnannotatedRegion |
| 1705 | GGCGTTATCC.CCGAGTGTCA | A | x | 35009_12_15657_XS2_1 | UnannotatedRegion |
| 1705 | GGCGTTATCC.CCGAGTGTCA | G | x | 35010_12_17186_XS2_1 | UnannotatedRegion |
| 1705 | GGCGTTATCC.CCGAGTGTCA | G | x | 35011_12_17194_XS2_1 | UnannotatedRegion |
| 1705 | GGCGTTATCC.CCGAGTGTCA | G | x | 35012_12_17009_XS2_1 | UnannotatedRegion |
| 1705 | GGCGTTATCC.CCGAGTGTCA | A | x | 35013_12_14973_XS2_1 | UnannotatedRegion |
|  |  |  |  |  |  |
| 1728 | GGTGCATAGG.GGGAAGATAA | C | 1695155 R | 19260_WUE_2594 | UnannotatedRegion |
| 1728 | GGTGCATAGG.GGGAAGATAA | T | x | 34990_Tchad54_11 | UnannotatedRegion |
| 1728 | GGTGCATAGG.GGGAAGATAA | T | x | 34991_Tchad78_11 | UnannotatedRegion |
| 1728 | GGTGCATAGG.GGGAAGATAA | T | x | 34992_Tchad95_11 | UnannotatedRegion |
| 1728 | GGTGCATAGG.GGGAAGATAA | T | x | 34994_Tchad106_11 | UnannotatedRegion |
| 1728 | GGTGCATAGG.GGGAAGATAA | T | x | 34995_8_2011 | UnannotatedRegion |
| 1728 | GGTGCATAGG.GGGAAGATAA | T | x | 34996_10_2011 | UnannotatedRegion |
| 1728 | GGTGCATAGG.GGGAAGATAA | T | x | 34997_39_2011 | UnannotatedRegion |
| 1728 | GGTGCATAGG.GGGAAGATAA | T | x | 34998_59_2011 | UnannotatedRegion |
| 1728 | GGTGCATAGG.GGGAAGATAA | T | x | 34999_63_2011 | UnannotatedRegion |
| 1728 | GGTGCATAGG.GGGAAGATAA | T | x | 35000_75_2011 | UnannotatedRegion |
| 1728 | GGTGCATAGG.GGGAAGATAA | T | x | 35001_76_2011 | UnannotatedRegion |
| 1728 | GGTGCATAGG.GGGAAGATAA | T | x | 35002_120_2011 | UnannotatedRegion |
| 1728 | GGTGCATAGG.GGGAAGATAA | T | x | 35003_403_2011 | UnannotatedRegion |
| 1728 | GGTGCATAGG.GGGAAGATAA | T | x | 35004_12_15398_XS2_1 | UnannotatedRegion |
| 1728 | GGTGCATAGG.GGGAAGATAA | T | x | 35005_12_13952_XS2_1 | UnannotatedRegion |
| 1728 | GGTGCATAGG.GGGAAGATAA | T | x | 35006_12_15317_XS2_1 | UnannotatedRegion |
| 1728 | GGTGCATAGG.GGGAAGATAA | T | x | 35007_12_15047_XS2_1 | UnannotatedRegion |
| 1728 | GGTGCATAGG.GGGAAGATAA | T | x | 35008_12_15661_XS2_1 | UnannotatedRegion |
| 1728 | GGTGCATAGG.GGGAAGATAA | T | x | 35009_12_15657_XS2_1 | UnannotatedRegion |
| 1728 | GGTGCATAGG.GGGAAGATAA | T | x | 35010_12_17186_XS2_1 | UnannotatedRegion |
| 1728 | GGTGCATAGG.GGGAAGATAA | T | x | 35011_12_17194_XS2_1 | UnannotatedRegion |
| 1728 | GGTGCATAGG.GGGAAGATAA | T | x | 35012_12_17009_XS2_1 | UnannotatedRegion |
| 1728 | GGTGCATAGG.GGGAAGATAA | T | x | 35013_12_14973_XS2_1 | UnannotatedRegion |
|  |  |  |  |  |  |
| 1738 | GTAAGCAAAC.TGCTTCAGAC | A | 1215599 R | 19260_WUE_2594 | UnannotatedRegion |
| 1738 | GTAAGCAAAC.TGCTTCAGAC | G | x | 34990_Tchad54_11 | UnannotatedRegion |
| 1738 | GTAAGCAAAC.TGCTTCAGAC | G | x | 34991_Tchad78_11 | UnannotatedRegion |
| 1738 | GTAAGCAAAC.TGCTTCAGAC | G | x | 34992_Tchad95_11 | UnannotatedRegion |
| 1738 | GTAAGCAAAC.TGCTTCAGAC | G | x | 34994_Tchad106_11 | UnannotatedRegion |
| 1738 | GTAAGCAAAC.TGCTTCAGAC | G | x | 34995_8_2011 | UnannotatedRegion |
| 1738 | GTAAGCAAAC.TGCTTCAGAC | G | x | 34996_10_2011 | UnannotatedRegion |
| 1738 | GTAAGCAAAC.TGCTTCAGAC | G | x | 34997_39_2011 | UnannotatedRegion |
| 1738 | GTAAGCAAAC.TGCTTCAGAC | G | x | 34998_59_2011 | UnannotatedRegion |
| 1738 | GTAAGCAAAC.TGCTTCAGAC | G | x | 34999_63_2011 | UnannotatedRegion |
| 1738 | GTAAGCAAAC.TGCTTCAGAC | G | x | 35000_75_2011 | UnannotatedRegion |
| 1738 | GTAAGCAAAC.TGCTTCAGAC | G | x | 35001_76_2011 | UnannotatedRegion |
| 1738 | GTAAGCAAAC.TGCTTCAGAC | G | x | 35002_120_2011 | UnannotatedRegion |
| 1738 | GTAAGCAAAC.TGCTTCAGAC | G | x | 35003_403_2011 | UnannotatedRegion |
| 1738 | GTAAGCAAAC.TGCTTCAGAC | G | x | 35004_12_15398_XS2_1 | UnannotatedRegion |
| 1738 | GTAAGCAAAC.TGCTTCAGAC | G | x | 35005_12_13952_XS2_1 | UnannotatedRegion |
| 1738 | GTAAGCAAAC.TGCTTCAGAC | G | x | 35006_12_15317_XS2_1 | UnannotatedRegion |
| 1738 | GTAAGCAAAC.TGCTTCAGAC | G | x | 35007_12_15047_XS2_1 | UnannotatedRegion |
| 1738 | GTAAGCAAAC.TGCTTCAGAC | G | x | 35008_12_15661_XS2_1 | UnannotatedRegion |
| 1738 | GTAAGCAAAC.TGCTTCAGAC | G | x | 35009_12_15657_XS2_1 | UnannotatedRegion |
| 1738 | GTAAGCAAAC.TGCTTCAGAC | G | x | 35010_12_17186_XS2_1 | UnannotatedRegion |
| 1738 | GTAAGCAAAC.TGCTTCAGAC | G | x | 35011_12_17194_XS2_1 | UnannotatedRegion |
| 1738 | GTAAGCAAAC.TGCTTCAGAC | G | x | 35012_12_17009_XS2_1 | UnannotatedRegion |
| 1738 | GTAAGCAAAC.TGCTTCAGAC | G | x | 35013_12_14973_XS2_1 | UnannotatedRegion |
|  |  |  |  |  |  |
| 1753 | GTCTAACGGA.GCGCCGGAGA | T | 851310 R | 19260_WUE_2594 | UnannotatedRegion |
| 1753 | GTCTAACGGA.GCGCCGGAGA | C | x | 34990_Tchad54_11 | UnannotatedRegion |
| 1753 | GTCTAACGGA.GCGCCGGAGA | C | x | 34991_Tchad78_11 | UnannotatedRegion |
| 1753 | GTCTAACGGA.GCGCCGGAGA | C | x | 34992_Tchad95_11 | UnannotatedRegion |
| 1753 | GTCTAACGGA.GCGCCGGAGA | C | x | 34994_Tchad106_11 | UnannotatedRegion |
| 1753 | GTCTAACGGA.GCGCCGGAGA | C | x | 34995_8_2011 | UnannotatedRegion |
| 1753 | GTCTAACGGA.GCGCCGGAGA | C | x | 34996_10_2011 | UnannotatedRegion |
| 1753 | GTCTAACGGA.GCGCCGGAGA | C | x | 34997_39_2011 | UnannotatedRegion |
| 1753 | GTCTAACGGA.GCGCCGGAGA | C | x | 34998_59_2011 | UnannotatedRegion |
| 1753 | GTCTAACGGA.GCGCCGGAGA | C | x | 34999_63_2011 | UnannotatedRegion |
| 1753 | GTCTAACGGA.GCGCCGGAGA | C | x | 35000_75_2011 | UnannotatedRegion |
| 1753 | GTCTAACGGA.GCGCCGGAGA | C | x | 35001_76_2011 | UnannotatedRegion |
| 1753 | GTCTAACGGA.GCGCCGGAGA | C | x | 35002_120_2011 | UnannotatedRegion |
| 1753 | GTCTAACGGA.GCGCCGGAGA | C | x | 35003_403_2011 | UnannotatedRegion |
| 1753 | GTCTAACGGA.GCGCCGGAGA | C | x | 35004_12_15398_XS2_1 | UnannotatedRegion |
| 1753 | GTCTAACGGA.GCGCCGGAGA | C | x | 35005_12_13952_XS2_1 | UnannotatedRegion |
| 1753 | GTCTAACGGA.GCGCCGGAGA | C | x | 35006_12_15317_XS2_1 | UnannotatedRegion |
| 1753 | GTCTAACGGA.GCGCCGGAGA | C | x | 35007_12_15047_XS2_1 | UnannotatedRegion |
| 1753 | GTCTAACGGA.GCGCCGGAGA | C | x | 35008_12_15661_XS2_1 | UnannotatedRegion |
| 1753 | GTCTAACGGA.GCGCCGGAGA | C | x | 35009_12_15657_XS2_1 | UnannotatedRegion |
| 1753 | GTCTAACGGA.GCGCCGGAGA | C | x | 35010_12_17186_XS2_1 | UnannotatedRegion |
| 1753 | GTCTAACGGA.GCGCCGGAGA | C | x | 35011_12_17194_XS2_1 | UnannotatedRegion |
| 1753 | GTCTAACGGA.GCGCCGGAGA | C | x | 35012_12_17009_XS2_1 | UnannotatedRegion |
| 1753 | GTCTAACGGA.GCGCCGGAGA | C | x | 35013_12_14973_XS2_1 | UnannotatedRegion |
|  |  |  |  |  |  |
| 1754 | GTCTGAAAAC.GGATGGGAAA | A | 742054 F | 19260_WUE_2594 | UnannotatedRegion |
| 1754 | GTCTGAAAAC.GGATGGGAAA | G | x | 34990_Tchad54_11 | UnannotatedRegion |
| 1754 | GTCTGAAAAC.GGATGGGAAA | G | x | 34991_Tchad78_11 | UnannotatedRegion |
| 1754 | GTCTGAAAAC.GGATGGGAAA | G | x | 34992_Tchad95_11 | UnannotatedRegion |
| 1754 | GTCTGAAAAC.GGATGGGAAA | G | x | 34994_Tchad106_11 | UnannotatedRegion |
| 1754 | GTCTGAAAAC.GGATGGGAAA | G | x | 34995_8_2011 | UnannotatedRegion |
| 1754 | GTCTGAAAAC.GGATGGGAAA | G | x | 34996_10_2011 | UnannotatedRegion |
| 1754 | GTCTGAAAAC.GGATGGGAAA | G | x | 34997_39_2011 | UnannotatedRegion |
| 1754 | GTCTGAAAAC.GGATGGGAAA | G | x | 34998_59_2011 | UnannotatedRegion |
| 1754 | GTCTGAAAAC.GGATGGGAAA | G | x | 34999_63_2011 | UnannotatedRegion |
| 1754 | GTCTGAAAAC.GGATGGGAAA | G | x | 35000_75_2011 | UnannotatedRegion |
| 1754 | GTCTGAAAAC.GGATGGGAAA | G | x | 35001_76_2011 | UnannotatedRegion |
| 1754 | GTCTGAAAAC.GGATGGGAAA | G | x | 35002_120_2011 | UnannotatedRegion |
| 1754 | GTCTGAAAAC.GGATGGGAAA | G | x | 35003_403_2011 | UnannotatedRegion |
| 1754 | GTCTGAAAAC.GGATGGGAAA | G | x | 35004_12_15398_XS2_1 | UnannotatedRegion |
| 1754 | GTCTGAAAAC.GGATGGGAAA | G | x | 35005_12_13952_XS2_1 | UnannotatedRegion |
| 1754 | GTCTGAAAAC.GGATGGGAAA | G | x | 35006_12_15317_XS2_1 | UnannotatedRegion |
| 1754 | GTCTGAAAAC.GGATGGGAAA | G | x | 35007_12_15047_XS2_1 | UnannotatedRegion |
| 1754 | GTCTGAAAAC.GGATGGGAAA | G | x | 35008_12_15661_XS2_1 | UnannotatedRegion |
| 1754 | GTCTGAAAAC.GGATGGGAAA | G | x | 35009_12_15657_XS2_1 | UnannotatedRegion |
| 1754 | GTCTGAAAAC.GGATGGGAAA | G | x | 35010_12_17186_XS2_1 | UnannotatedRegion |
| 1754 | GTCTGAAAAC.GGATGGGAAA | G | x | 35011_12_17194_XS2_1 | UnannotatedRegion |
| 1754 | GTCTGAAAAC.GGATGGGAAA | G | x | 35012_12_17009_XS2_1 | UnannotatedRegion |
| 1754 | GTCTGAAAAC.GGATGGGAAA | G | x | 35013_12_14973_XS2_1 | UnannotatedRegion |
|  |  |  |  |  |  |
| 1764 | GTTATTATAT.GCAAACGGCA | A | 856189 F | 19260_WUE_2594 | UnannotatedRegion |
| 1764 | GTTATTATAT.GCAAACGGCA | C | x | 34990_Tchad54_11 | UnannotatedRegion |
| 1764 | GTTATTATAT.GCAAACGGCA | C | x | 34991_Tchad78_11 | UnannotatedRegion |
| 1764 | GTTATTATAT.GCAAACGGCA | C | x | 34992_Tchad95_11 | UnannotatedRegion |
| 1764 | GTTATTATAT.GCAAACGGCA | C | x | 34994_Tchad106_11 | UnannotatedRegion |
| 1764 | GTTATTATAT.GCAAACGGCA | C | x | 34995_8_2011 | UnannotatedRegion |
| 1764 | GTTATTATAT.GCAAACGGCA | C | x | 34996_10_2011 | UnannotatedRegion |
| 1764 | GTTATTATAT.GCAAACGGCA | C | x | 34997_39_2011 | UnannotatedRegion |
| 1764 | GTTATTATAT.GCAAACGGCA | C | x | 34998_59_2011 | UnannotatedRegion |
| 1764 | GTTATTATAT.GCAAACGGCA | C | x | 34999_63_2011 | UnannotatedRegion |
| 1764 | GTTATTATAT.GCAAACGGCA | C | x | 35000_75_2011 | UnannotatedRegion |
| 1764 | GTTATTATAT.GCAAACGGCA | C | x | 35001_76_2011 | UnannotatedRegion |
| 1764 | GTTATTATAT.GCAAACGGCA | C | x | 35002_120_2011 | UnannotatedRegion |
| 1764 | GTTATTATAT.GCAAACGGCA | C | x | 35003_403_2011 | UnannotatedRegion |
| 1764 | GTTATTATAT.GCAAACGGCA | C | x | 35004_12_15398_XS2_1 | UnannotatedRegion |
| 1764 | GTTATTATAT.GCAAACGGCA | C | x | 35005_12_13952_XS2_1 | UnannotatedRegion |
| 1764 | GTTATTATAT.GCAAACGGCA | C | x | 35006_12_15317_XS2_1 | UnannotatedRegion |
| 1764 | GTTATTATAT.GCAAACGGCA | C | x | 35007_12_15047_XS2_1 | UnannotatedRegion |
| 1764 | GTTATTATAT.GCAAACGGCA | C | x | 35008_12_15661_XS2_1 | UnannotatedRegion |
| 1764 | GTTATTATAT.GCAAACGGCA | C | x | 35009_12_15657_XS2_1 | UnannotatedRegion |
| 1764 | GTTATTATAT.GCAAACGGCA | C | x | 35010_12_17186_XS2_1 | UnannotatedRegion |
| 1764 | GTTATTATAT.GCAAACGGCA | C | x | 35011_12_17194_XS2_1 | UnannotatedRegion |
| 1764 | GTTATTATAT.GCAAACGGCA | C | x | 35012_12_17009_XS2_1 | UnannotatedRegion |
| 1764 | GTTATTATAT.GCAAACGGCA | C | x | 35013_12_14973_XS2_1 | UnannotatedRegion |
|  |  |  |  |  |  |
| 1765 | GTTCTACAAA.TAAAGTGGTA | A | 2172965 F | 19260_WUE_2594 | UnannotatedRegion |
| 1765 | GTTCTACAAA.TAAAGTGGTA | A | x | 34990_Tchad54_11 | UnannotatedRegion |
| 1765 | GTTCTACAAA.TAAAGTGGTA | A | x | 34991_Tchad78_11 | UnannotatedRegion |
| 1765 | GTTCTACAAA.TAAAGTGGTA | A | x | 34992_Tchad95_11 | UnannotatedRegion |
| 1765 | GTTCTACAAA.TAAAGTGGTA | A | x | 34994_Tchad106_11 | UnannotatedRegion |
| 1765 | GTTCTACAAA.TAAAGTGGTA | G | x | 34995_8_2011 | UnannotatedRegion |
| 1765 | GTTCTACAAA.TAAAGTGGTA | G | x | 34996_10_2011 | UnannotatedRegion |
| 1765 | GTTCTACAAA.TAAAGTGGTA | A | x | 34997_39_2011 | UnannotatedRegion |
| 1765 | GTTCTACAAA.TAAAGTGGTA | A | x | 34998_59_2011 | UnannotatedRegion |
| 1765 | GTTCTACAAA.TAAAGTGGTA | A | x | 34999_63_2011 | UnannotatedRegion |
| 1765 | GTTCTACAAA.TAAAGTGGTA | A | x | 35000_75_2011 | UnannotatedRegion |
| 1765 | GTTCTACAAA.TAAAGTGGTA | A | x | 35001_76_2011 | UnannotatedRegion |
| 1765 | GTTCTACAAA.TAAAGTGGTA | A | x | 35002_120_2011 | UnannotatedRegion |
| 1765 | GTTCTACAAA.TAAAGTGGTA | A | x | 35003_403_2011 | UnannotatedRegion |
| 1765 | GTTCTACAAA.TAAAGTGGTA | A | x | 35004_12_15398_XS2_1 | UnannotatedRegion |
| 1765 | GTTCTACAAA.TAAAGTGGTA | A | x | 35005_12_13952_XS2_1 | UnannotatedRegion |
| 1765 | GTTCTACAAA.TAAAGTGGTA | A | x | 35006_12_15317_XS2_1 | UnannotatedRegion |
| 1765 | GTTCTACAAA.TAAAGTGGTA | A | x | 35007_12_15047_XS2_1 | UnannotatedRegion |
| 1765 | GTTCTACAAA.TAAAGTGGTA | A | x | 35008_12_15661_XS2_1 | UnannotatedRegion |
| 1765 | GTTCTACAAA.TAAAGTGGTA | A | x | 35009_12_15657_XS2_1 | UnannotatedRegion |
| 1765 | GTTCTACAAA.TAAAGTGGTA | A | x | 35010_12_17186_XS2_1 | UnannotatedRegion |
| 1765 | GTTCTACAAA.TAAAGTGGTA | A | x | 35011_12_17194_XS2_1 | UnannotatedRegion |
| 1765 | GTTCTACAAA.TAAAGTGGTA | A | x | 35012_12_17009_XS2_1 | UnannotatedRegion |
| 1765 | GTTCTACAAA.TAAAGTGGTA | A | x | 35013_12_14973_XS2_1 | UnannotatedRegion |
|  |  |  |  |  |  |
| 1771 | GTTTCCATTG.GCAAATGAAA | G | 1256139 R | 19260_WUE_2594 | UnannotatedRegion |
| 1771 | GTTTCCATTG.GCAAATGAAA | A | x | 34990_Tchad54_11 | UnannotatedRegion |
| 1771 | GTTTCCATTG.GCAAATGAAA | A | x | 34991_Tchad78_11 | UnannotatedRegion |
| 1771 | GTTTCCATTG.GCAAATGAAA | A | x | 34992_Tchad95_11 | UnannotatedRegion |
| 1771 | GTTTCCATTG.GCAAATGAAA | A | x | 34994_Tchad106_11 | UnannotatedRegion |
| 1771 | GTTTCCATTG.GCAAATGAAA | A | x | 34995_8_2011 | UnannotatedRegion |
| 1771 | GTTTCCATTG.GCAAATGAAA | A | x | 34996_10_2011 | UnannotatedRegion |
| 1771 | GTTTCCATTG.GCAAATGAAA | A | x | 34997_39_2011 | UnannotatedRegion |
| 1771 | GTTTCCATTG.GCAAATGAAA | A | x | 34998_59_2011 | UnannotatedRegion |
| 1771 | GTTTCCATTG.GCAAATGAAA | A | x | 34999_63_2011 | UnannotatedRegion |
| 1771 | GTTTCCATTG.GCAAATGAAA | A | x | 35000_75_2011 | UnannotatedRegion |
| 1771 | GTTTCCATTG.GCAAATGAAA | A | x | 35001_76_2011 | UnannotatedRegion |
| 1771 | GTTTCCATTG.GCAAATGAAA | A | x | 35002_120_2011 | UnannotatedRegion |
| 1771 | GTTTCCATTG.GCAAATGAAA | A | x | 35003_403_2011 | UnannotatedRegion |
| 1771 | GTTTCCATTG.GCAAATGAAA | A | x | 35004_12_15398_XS2_1 | UnannotatedRegion |
| 1771 | GTTTCCATTG.GCAAATGAAA | A | x | 35005_12_13952_XS2_1 | UnannotatedRegion |
| 1771 | GTTTCCATTG.GCAAATGAAA | A | x | 35006_12_15317_XS2_1 | UnannotatedRegion |
| 1771 | GTTTCCATTG.GCAAATGAAA | A | x | 35007_12_15047_XS2_1 | UnannotatedRegion |
| 1771 | GTTTCCATTG.GCAAATGAAA | A | x | 35008_12_15661_XS2_1 | UnannotatedRegion |
| 1771 | GTTTCCATTG.GCAAATGAAA | A | x | 35009_12_15657_XS2_1 | UnannotatedRegion |
| 1771 | GTTTCCATTG.GCAAATGAAA | A | x | 35010_12_17186_XS2_1 | UnannotatedRegion |
| 1771 | GTTTCCATTG.GCAAATGAAA | A | x | 35011_12_17194_XS2_1 | UnannotatedRegion |
| 1771 | GTTTCCATTG.GCAAATGAAA | A | x | 35012_12_17009_XS2_1 | UnannotatedRegion |
| 1771 | GTTTCCATTG.GCAAATGAAA | A | x | 35013_12_14973_XS2_1 | UnannotatedRegion |
|  |  |  |  |  |  |
| 1786 | TAAATTAATG.AATAATGCAA | T | 1124059 F | 19260_WUE_2594 | UnannotatedRegion |
| 1786 | TAAATTAATG.AATAATGCAA | C | x | 34990_Tchad54_11 | UnannotatedRegion |
| 1786 | TAAATTAATG.AATAATGCAA | C | x | 34991_Tchad78_11 | UnannotatedRegion |
| 1786 | TAAATTAATG.AATAATGCAA | C | x | 34992_Tchad95_11 | UnannotatedRegion |
| 1786 | TAAATTAATG.AATAATGCAA | C | x | 34994_Tchad106_11 | UnannotatedRegion |
| 1786 | TAAATTAATG.AATAATGCAA | C | x | 34995_8_2011 | UnannotatedRegion |
| 1786 | TAAATTAATG.AATAATGCAA | C | x | 34996_10_2011 | UnannotatedRegion |
| 1786 | TAAATTAATG.AATAATGCAA | C | x | 34997_39_2011 | UnannotatedRegion |
| 1786 | TAAATTAATG.AATAATGCAA | C | x | 34998_59_2011 | UnannotatedRegion |
| 1786 | TAAATTAATG.AATAATGCAA | C | x | 34999_63_2011 | UnannotatedRegion |
| 1786 | TAAATTAATG.AATAATGCAA | C | x | 35000_75_2011 | UnannotatedRegion |
| 1786 | TAAATTAATG.AATAATGCAA | C | x | 35001_76_2011 | UnannotatedRegion |
| 1786 | TAAATTAATG.AATAATGCAA | C | x | 35002_120_2011 | UnannotatedRegion |
| 1786 | TAAATTAATG.AATAATGCAA | C | x | 35003_403_2011 | UnannotatedRegion |
| 1786 | TAAATTAATG.AATAATGCAA | C | x | 35004_12_15398_XS2_1 | UnannotatedRegion |
| 1786 | TAAATTAATG.AATAATGCAA | C | x | 35005_12_13952_XS2_1 | UnannotatedRegion |
| 1786 | TAAATTAATG.AATAATGCAA | C | x | 35006_12_15317_XS2_1 | UnannotatedRegion |
| 1786 | TAAATTAATG.AATAATGCAA | C | x | 35007_12_15047_XS2_1 | UnannotatedRegion |
| 1786 | TAAATTAATG.AATAATGCAA | C | x | 35008_12_15661_XS2_1 | UnannotatedRegion |
| 1786 | TAAATTAATG.AATAATGCAA | C | x | 35009_12_15657_XS2_1 | UnannotatedRegion |
| 1786 | TAAATTAATG.AATAATGCAA | C | x | 35010_12_17186_XS2_1 | UnannotatedRegion |
| 1786 | TAAATTAATG.AATAATGCAA | C | x | 35011_12_17194_XS2_1 | UnannotatedRegion |
| 1786 | TAAATTAATG.AATAATGCAA | C | x | 35012_12_17009_XS2_1 | UnannotatedRegion |
| 1786 | TAAATTAATG.AATAATGCAA | C | x | 35013_12_14973_XS2_1 | UnannotatedRegion |
|  |  |  |  |  |  |
| 1790 | TAATCCTGTT.CTTATGGAAA | G | 529244 R | 19260_WUE_2594 | UnannotatedRegion |
| 1790 | TAATCCTGTT.CTTATGGAAA | A | x | 34990_Tchad54_11 | UnannotatedRegion |
| 1790 | TAATCCTGTT.CTTATGGAAA | G | x | 34991_Tchad78_11 | UnannotatedRegion |
| 1790 | TAATCCTGTT.CTTATGGAAA | G | x | 34992_Tchad95_11 | UnannotatedRegion |
| 1790 | TAATCCTGTT.CTTATGGAAA | G | x | 34994_Tchad106_11 | UnannotatedRegion |
| 1790 | TAATCCTGTT.CTTATGGAAA | G | x | 34995_8_2011 | UnannotatedRegion |
| 1790 | TAATCCTGTT.CTTATGGAAA | G | x | 34996_10_2011 | UnannotatedRegion |
| 1790 | TAATCCTGTT.CTTATGGAAA | A | x | 34997_39_2011 | UnannotatedRegion |
| 1790 | TAATCCTGTT.CTTATGGAAA | A | x | 34998_59_2011 | UnannotatedRegion |
| 1790 | TAATCCTGTT.CTTATGGAAA | A | x | 34999_63_2011 | UnannotatedRegion |
| 1790 | TAATCCTGTT.CTTATGGAAA | A | x | 35000_75_2011 | UnannotatedRegion |
| 1790 | TAATCCTGTT.CTTATGGAAA | A | x | 35001_76_2011 | UnannotatedRegion |
| 1790 | TAATCCTGTT.CTTATGGAAA | A | x | 35002_120_2011 | UnannotatedRegion |
| 1790 | TAATCCTGTT.CTTATGGAAA | G | x | 35003_403_2011 | UnannotatedRegion |
| 1790 | TAATCCTGTT.CTTATGGAAA | A | x | 35004_12_15398_XS2_1 | UnannotatedRegion |
| 1790 | TAATCCTGTT.CTTATGGAAA | A | x | 35005_12_13952_XS2_1 | UnannotatedRegion |
| 1790 | TAATCCTGTT.CTTATGGAAA | A | x | 35006_12_15317_XS2_1 | UnannotatedRegion |
| 1790 | TAATCCTGTT.CTTATGGAAA | G | x | 35007_12_15047_XS2_1 | UnannotatedRegion |
| 1790 | TAATCCTGTT.CTTATGGAAA | G | x | 35008_12_15661_XS2_1 | UnannotatedRegion |
| 1790 | TAATCCTGTT.CTTATGGAAA | G | x | 35009_12_15657_XS2_1 | UnannotatedRegion |
| 1790 | TAATCCTGTT.CTTATGGAAA | A | x | 35010_12_17186_XS2_1 | UnannotatedRegion |
| 1790 | TAATCCTGTT.CTTATGGAAA | A | x | 35011_12_17194_XS2_1 | UnannotatedRegion |
| 1790 | TAATCCTGTT.CTTATGGAAA | A | x | 35012_12_17009_XS2_1 | UnannotatedRegion |
| 1790 | TAATCCTGTT.CTTATGGAAA | G | x | 35013_12_14973_XS2_1 | UnannotatedRegion |
|  |  |  |  |  |  |
| 1793 | TACCAATACC.AACAGACAAA | A | 2037386 F | 19260_WUE_2594 | UnannotatedRegion |
| 1793 | TACCAATACC.AACAGACAAA | C | x | 34990_Tchad54_11 | UnannotatedRegion |
| 1793 | TACCAATACC.AACAGACAAA | A | x | 34995_8_2011 | UnannotatedRegion |
| 1793 | TACCAATACC.AACAGACAAA | A | x | 34996_10_2011 | UnannotatedRegion |
| 1793 | TACCAATACC.AACAGACAAA | C | x | 34997_39_2011 | UnannotatedRegion |
| 1793 | TACCAATACC.AACAGACAAA | C | x | 34998_59_2011 | UnannotatedRegion |
| 1793 | TACCAATACC.AACAGACAAA | C | x | 34999_63_2011 | UnannotatedRegion |
| 1793 | TACCAATACC.AACAGACAAA | C | x | 35000_75_2011 | UnannotatedRegion |
| 1793 | TACCAATACC.AACAGACAAA | C | x | 35001_76_2011 | UnannotatedRegion |
| 1793 | TACCAATACC.AACAGACAAA | C | x | 35002_120_2011 | UnannotatedRegion |
| 1793 | TACCAATACC.AACAGACAAA | C | x | 35004_12_15398_XS2_1 | UnannotatedRegion |
| 1793 | TACCAATACC.AACAGACAAA | C | x | 35005_12_13952_XS2_1 | UnannotatedRegion |
| 1793 | TACCAATACC.AACAGACAAA | C | x | 35006_12_15317_XS2_1 | UnannotatedRegion |
| 1793 | TACCAATACC.AACAGACAAA | C | x | 35010_12_17186_XS2_1 | UnannotatedRegion |
| 1793 | TACCAATACC.AACAGACAAA | C | x | 35011_12_17194_XS2_1 | UnannotatedRegion |
| 1793 | TACCAATACC.AACAGACAAA | C | x | 35012_12_17009_XS2_1 | UnannotatedRegion |
|  |  |  |  |  |  |
| 1803 | TATAAAAAAT.TTTTTCTTAA | C | 1398299 R | 19260_WUE_2594 | UnannotatedRegion |
| 1803 | TATAAAAAAT.TTTTTCTTAA | A | x | 34990_Tchad54_11 | UnannotatedRegion |
| 1803 | TATAAAAAAT.TTTTTCTTAA | A | x | 34991_Tchad78_11 | UnannotatedRegion |
| 1803 | TATAAAAAAT.TTTTTCTTAA | A | x | 34992_Tchad95_11 | UnannotatedRegion |
| 1803 | TATAAAAAAT.TTTTTCTTAA | A | x | 34994_Tchad106_11 | UnannotatedRegion |
| 1803 | TATAAAAAAT.TTTTTCTTAA | A | x | 34995_8_2011 | UnannotatedRegion |
| 1803 | TATAAAAAAT.TTTTTCTTAA | A | x | 34996_10_2011 | UnannotatedRegion |
| 1803 | TATAAAAAAT.TTTTTCTTAA | A | x | 34997_39_2011 | UnannotatedRegion |
| 1803 | TATAAAAAAT.TTTTTCTTAA | A | x | 34998_59_2011 | UnannotatedRegion |
| 1803 | TATAAAAAAT.TTTTTCTTAA | A | x | 34999_63_2011 | UnannotatedRegion |
| 1803 | TATAAAAAAT.TTTTTCTTAA | A | x | 35000_75_2011 | UnannotatedRegion |
| 1803 | TATAAAAAAT.TTTTTCTTAA | A | x | 35001_76_2011 | UnannotatedRegion |
| 1803 | TATAAAAAAT.TTTTTCTTAA | A | x | 35002_120_2011 | UnannotatedRegion |
| 1803 | TATAAAAAAT.TTTTTCTTAA | A | x | 35003_403_2011 | UnannotatedRegion |
| 1803 | TATAAAAAAT.TTTTTCTTAA | A | x | 35004_12_15398_XS2_1 | UnannotatedRegion |
| 1803 | TATAAAAAAT.TTTTTCTTAA | A | x | 35005_12_13952_XS2_1 | UnannotatedRegion |
| 1803 | TATAAAAAAT.TTTTTCTTAA | A | x | 35006_12_15317_XS2_1 | UnannotatedRegion |
| 1803 | TATAAAAAAT.TTTTTCTTAA | A | x | 35007_12_15047_XS2_1 | UnannotatedRegion |
| 1803 | TATAAAAAAT.TTTTTCTTAA | A | x | 35008_12_15661_XS2_1 | UnannotatedRegion |
| 1803 | TATAAAAAAT.TTTTTCTTAA | A | x | 35009_12_15657_XS2_1 | UnannotatedRegion |
| 1803 | TATAAAAAAT.TTTTTCTTAA | A | x | 35010_12_17186_XS2_1 | UnannotatedRegion |
| 1803 | TATAAAAAAT.TTTTTCTTAA | A | x | 35011_12_17194_XS2_1 | UnannotatedRegion |
| 1803 | TATAAAAAAT.TTTTTCTTAA | A | x | 35012_12_17009_XS2_1 | UnannotatedRegion |
| 1803 | TATAAAAAAT.TTTTTCTTAA | A | x | 35013_12_14973_XS2_1 | UnannotatedRegion |
|  |  |  |  |  |  |
| 1809 | TATCGCCGCC.ATATGCCGCA | C | 2160757 R | 19260_WUE_2594 | UnannotatedRegion |
| 1809 | TATCGCCGCC.ATATGCCGCA | C | x | 34990_Tchad54_11 | UnannotatedRegion |
| 1809 | TATCGCCGCC.ATATGCCGCA | C | x | 34991_Tchad78_11 | UnannotatedRegion |
| 1809 | TATCGCCGCC.ATATGCCGCA | T | x | 34992_Tchad95_11 | UnannotatedRegion |
| 1809 | TATCGCCGCC.ATATGCCGCA | T | x | 34994_Tchad106_11 | UnannotatedRegion |
| 1809 | TATCGCCGCC.ATATGCCGCA | C | x | 34995_8_2011 | UnannotatedRegion |
| 1809 | TATCGCCGCC.ATATGCCGCA | C | x | 34996_10_2011 | UnannotatedRegion |
| 1809 | TATCGCCGCC.ATATGCCGCA | C | x | 34997_39_2011 | UnannotatedRegion |
| 1809 | TATCGCCGCC.ATATGCCGCA | C | x | 34998_59_2011 | UnannotatedRegion |
| 1809 | TATCGCCGCC.ATATGCCGCA | C | x | 34999_63_2011 | UnannotatedRegion |
| 1809 | TATCGCCGCC.ATATGCCGCA | C | x | 35000_75_2011 | UnannotatedRegion |
| 1809 | TATCGCCGCC.ATATGCCGCA | C | x | 35001_76_2011 | UnannotatedRegion |
| 1809 | TATCGCCGCC.ATATGCCGCA | C | x | 35002_120_2011 | UnannotatedRegion |
| 1809 | TATCGCCGCC.ATATGCCGCA | C | x | 35003_403_2011 | UnannotatedRegion |
| 1809 | TATCGCCGCC.ATATGCCGCA | C | x | 35004_12_15398_XS2_1 | UnannotatedRegion |
| 1809 | TATCGCCGCC.ATATGCCGCA | C | x | 35005_12_13952_XS2_1 | UnannotatedRegion |
| 1809 | TATCGCCGCC.ATATGCCGCA | C | x | 35006_12_15317_XS2_1 | UnannotatedRegion |
| 1809 | TATCGCCGCC.ATATGCCGCA | T | x | 35007_12_15047_XS2_1 | UnannotatedRegion |
| 1809 | TATCGCCGCC.ATATGCCGCA | T | x | 35008_12_15661_XS2_1 | UnannotatedRegion |
| 1809 | TATCGCCGCC.ATATGCCGCA | T | x | 35009_12_15657_XS2_1 | UnannotatedRegion |
| 1809 | TATCGCCGCC.ATATGCCGCA | C | x | 35010_12_17186_XS2_1 | UnannotatedRegion |
| 1809 | TATCGCCGCC.ATATGCCGCA | C | x | 35011_12_17194_XS2_1 | UnannotatedRegion |
| 1809 | TATCGCCGCC.ATATGCCGCA | C | x | 35012_12_17009_XS2_1 | UnannotatedRegion |
| 1809 | TATCGCCGCC.ATATGCCGCA | T | x | 35013_12_14973_XS2_1 | UnannotatedRegion |
|  |  |  |  |  |  |
| 1841 | TCCTCACCCT.CGGGCCGAAA | G | x | 34990_Tchad54_11 | NotInAnnotatedGenome |
| 1841 | TCCTCACCCT.CGGGCCGAAA | G | x | 34991_Tchad78_11 | NotInAnnotatedGenome |
| 1841 | TCCTCACCCT.CGGGCCGAAA | G | x | 34992_Tchad95_11 | NotInAnnotatedGenome |
| 1841 | TCCTCACCCT.CGGGCCGAAA | G | x | 34994_Tchad106_11 | NotInAnnotatedGenome |
| 1841 | TCCTCACCCT.CGGGCCGAAA | G | x | 34995_8_2011 | NotInAnnotatedGenome |
| 1841 | TCCTCACCCT.CGGGCCGAAA | G | x | 34996_10_2011 | NotInAnnotatedGenome |
| 1841 | TCCTCACCCT.CGGGCCGAAA | G | x | 34997_39_2011 | NotInAnnotatedGenome |
| 1841 | TCCTCACCCT.CGGGCCGAAA | G | x | 34998_59_2011 | NotInAnnotatedGenome |
| 1841 | TCCTCACCCT.CGGGCCGAAA | G | x | 34999_63_2011 | NotInAnnotatedGenome |
| 1841 | TCCTCACCCT.CGGGCCGAAA | G | x | 35000_75_2011 | NotInAnnotatedGenome |
| 1841 | TCCTCACCCT.CGGGCCGAAA | G | x | 35001_76_2011 | NotInAnnotatedGenome |
| 1841 | TCCTCACCCT.CGGGCCGAAA | G | x | 35002_120_2011 | NotInAnnotatedGenome |
| 1841 | TCCTCACCCT.CGGGCCGAAA | G | x | 35003_403_2011 | NotInAnnotatedGenome |
| 1841 | TCCTCACCCT.CGGGCCGAAA | G | x | 35004_12_15398_XS2_1 | NotInAnnotatedGenome |
| 1841 | TCCTCACCCT.CGGGCCGAAA | G | x | 35005_12_13952_XS2_1 | NotInAnnotatedGenome |
| 1841 | TCCTCACCCT.CGGGCCGAAA | G | x | 35006_12_15317_XS2_1 | NotInAnnotatedGenome |
| 1841 | TCCTCACCCT.CGGGCCGAAA | T | x | 35007_12_15047_XS2_1 | NotInAnnotatedGenome |
| 1841 | TCCTCACCCT.CGGGCCGAAA | G | x | 35008_12_15661_XS2_1 | NotInAnnotatedGenome |
| 1841 | TCCTCACCCT.CGGGCCGAAA | G | x | 35009_12_15657_XS2_1 | NotInAnnotatedGenome |
| 1841 | TCCTCACCCT.CGGGCCGAAA | G | x | 35010_12_17186_XS2_1 | NotInAnnotatedGenome |
| 1841 | TCCTCACCCT.CGGGCCGAAA | G | x | 35011_12_17194_XS2_1 | NotInAnnotatedGenome |
| 1841 | TCCTCACCCT.CGGGCCGAAA | G | x | 35012_12_17009_XS2_1 | NotInAnnotatedGenome |
| 1841 | TCCTCACCCT.CGGGCCGAAA | G | x | 35013_12_14973_XS2_1 | NotInAnnotatedGenome |
|  |  |  |  |  |  |
| 1843 | TCCTGCCGCA.ACGCCGCAGA | C | 1253393 F | 19260_WUE_2594 | UnannotatedRegion |
| 1843 | TCCTGCCGCA.ACGCCGCAGA | T | x | 34990_Tchad54_11 | UnannotatedRegion |
| 1843 | TCCTGCCGCA.ACGCCGCAGA | T | x | 34991_Tchad78_11 | UnannotatedRegion |
| 1843 | TCCTGCCGCA.ACGCCGCAGA | T | x | 34992_Tchad95_11 | UnannotatedRegion |
| 1843 | TCCTGCCGCA.ACGCCGCAGA | T | x | 34994_Tchad106_11 | UnannotatedRegion |
| 1843 | TCCTGCCGCA.ACGCCGCAGA | T | x | 34995_8_2011 | UnannotatedRegion |
| 1843 | TCCTGCCGCA.ACGCCGCAGA | T | x | 34996_10_2011 | UnannotatedRegion |
| 1843 | TCCTGCCGCA.ACGCCGCAGA | T | x | 34997_39_2011 | UnannotatedRegion |
| 1843 | TCCTGCCGCA.ACGCCGCAGA | T | x | 34998_59_2011 | UnannotatedRegion |
| 1843 | TCCTGCCGCA.ACGCCGCAGA | T | x | 34999_63_2011 | UnannotatedRegion |
| 1843 | TCCTGCCGCA.ACGCCGCAGA | T | x | 35000_75_2011 | UnannotatedRegion |
| 1843 | TCCTGCCGCA.ACGCCGCAGA | T | x | 35001_76_2011 | UnannotatedRegion |
| 1843 | TCCTGCCGCA.ACGCCGCAGA | T | x | 35002_120_2011 | UnannotatedRegion |
| 1843 | TCCTGCCGCA.ACGCCGCAGA | T | x | 35003_403_2011 | UnannotatedRegion |
| 1843 | TCCTGCCGCA.ACGCCGCAGA | T | x | 35004_12_15398_XS2_1 | UnannotatedRegion |
| 1843 | TCCTGCCGCA.ACGCCGCAGA | T | x | 35005_12_13952_XS2_1 | UnannotatedRegion |
| 1843 | TCCTGCCGCA.ACGCCGCAGA | T | x | 35006_12_15317_XS2_1 | UnannotatedRegion |
| 1843 | TCCTGCCGCA.ACGCCGCAGA | T | x | 35007_12_15047_XS2_1 | UnannotatedRegion |
| 1843 | TCCTGCCGCA.ACGCCGCAGA | T | x | 35008_12_15661_XS2_1 | UnannotatedRegion |
| 1843 | TCCTGCCGCA.ACGCCGCAGA | T | x | 35009_12_15657_XS2_1 | UnannotatedRegion |
| 1843 | TCCTGCCGCA.ACGCCGCAGA | T | x | 35010_12_17186_XS2_1 | UnannotatedRegion |
| 1843 | TCCTGCCGCA.ACGCCGCAGA | T | x | 35011_12_17194_XS2_1 | UnannotatedRegion |
| 1843 | TCCTGCCGCA.ACGCCGCAGA | T | x | 35012_12_17009_XS2_1 | UnannotatedRegion |
| 1843 | TCCTGCCGCA.ACGCCGCAGA | T | x | 35013_12_14973_XS2_1 | UnannotatedRegion |
|  |  |  |  |  |  |
| 1844 | TCCTTCCGAT.CCGTCTGAAA | A | 1731019 R | 19260_WUE_2594 | UnannotatedRegion |
| 1844 | TCCTTCCGAT.CCGTCTGAAA | A | x | 34990_Tchad54_11 | UnannotatedRegion |
| 1844 | TCCTTCCGAT.CCGTCTGAAA | A | x | 34991_Tchad78_11 | UnannotatedRegion |
| 1844 | TCCTTCCGAT.CCGTCTGAAA | A | x | 34992_Tchad95_11 | UnannotatedRegion |
| 1844 | TCCTTCCGAT.CCGTCTGAAA | A | x | 34994_Tchad106_11 | UnannotatedRegion |
| 1844 | TCCTTCCGAT.CCGTCTGAAA | A | x | 34995_8_2011 | UnannotatedRegion |
| 1844 | TCCTTCCGAT.CCGTCTGAAA | A | x | 34996_10_2011 | UnannotatedRegion |
| 1844 | TCCTTCCGAT.CCGTCTGAAA | A | x | 34997_39_2011 | UnannotatedRegion |
| 1844 | TCCTTCCGAT.CCGTCTGAAA | A | x | 34998_59_2011 | UnannotatedRegion |
| 1844 | TCCTTCCGAT.CCGTCTGAAA | A | x | 34999_63_2011 | UnannotatedRegion |
| 1844 | TCCTTCCGAT.CCGTCTGAAA | A | x | 35000_75_2011 | UnannotatedRegion |
| 1844 | TCCTTCCGAT.CCGTCTGAAA | A | x | 35001_76_2011 | UnannotatedRegion |
| 1844 | TCCTTCCGAT.CCGTCTGAAA | A | x | 35002_120_2011 | UnannotatedRegion |
| 1844 | TCCTTCCGAT.CCGTCTGAAA | A | x | 35003_403_2011 | UnannotatedRegion |
| 1844 | TCCTTCCGAT.CCGTCTGAAA | A | x | 35004_12_15398_XS2_1 | UnannotatedRegion |
| 1844 | TCCTTCCGAT.CCGTCTGAAA | A | x | 35005_12_13952_XS2_1 | UnannotatedRegion |
| 1844 | TCCTTCCGAT.CCGTCTGAAA | A | x | 35006_12_15317_XS2_1 | UnannotatedRegion |
| 1844 | TCCTTCCGAT.CCGTCTGAAA | G | x | 35007_12_15047_XS2_1 | UnannotatedRegion |
| 1844 | TCCTTCCGAT.CCGTCTGAAA | A | x | 35008_12_15661_XS2_1 | UnannotatedRegion |
| 1844 | TCCTTCCGAT.CCGTCTGAAA | A | x | 35009_12_15657_XS2_1 | UnannotatedRegion |
| 1844 | TCCTTCCGAT.CCGTCTGAAA | A | x | 35010_12_17186_XS2_1 | UnannotatedRegion |
| 1844 | TCCTTCCGAT.CCGTCTGAAA | A | x | 35011_12_17194_XS2_1 | UnannotatedRegion |
| 1844 | TCCTTCCGAT.CCGTCTGAAA | A | x | 35012_12_17009_XS2_1 | UnannotatedRegion |
| 1844 | TCCTTCCGAT.CCGTCTGAAA | A | x | 35013_12_14973_XS2_1 | UnannotatedRegion |
|  |  |  |  |  |  |
| 1854 | TCGCGGCGGC.GCACCGAGCA | T | 856443 R | 19260_WUE_2594 | UnannotatedRegion |
| 1854 | TCGCGGCGGC.GCACCGAGCA | C | x | 34990_Tchad54_11 | UnannotatedRegion |
| 1854 | TCGCGGCGGC.GCACCGAGCA | C | x | 34991_Tchad78_11 | UnannotatedRegion |
| 1854 | TCGCGGCGGC.GCACCGAGCA | C | x | 34992_Tchad95_11 | UnannotatedRegion |
| 1854 | TCGCGGCGGC.GCACCGAGCA | C | x | 34994_Tchad106_11 | UnannotatedRegion |
| 1854 | TCGCGGCGGC.GCACCGAGCA | C | x | 34995_8_2011 | UnannotatedRegion |
| 1854 | TCGCGGCGGC.GCACCGAGCA | C | x | 34996_10_2011 | UnannotatedRegion |
| 1854 | TCGCGGCGGC.GCACCGAGCA | C | x | 34997_39_2011 | UnannotatedRegion |
| 1854 | TCGCGGCGGC.GCACCGAGCA | C | x | 34998_59_2011 | UnannotatedRegion |
| 1854 | TCGCGGCGGC.GCACCGAGCA | C | x | 34999_63_2011 | UnannotatedRegion |
| 1854 | TCGCGGCGGC.GCACCGAGCA | C | x | 35000_75_2011 | UnannotatedRegion |
| 1854 | TCGCGGCGGC.GCACCGAGCA | C | x | 35001_76_2011 | UnannotatedRegion |
| 1854 | TCGCGGCGGC.GCACCGAGCA | C | x | 35002_120_2011 | UnannotatedRegion |
| 1854 | TCGCGGCGGC.GCACCGAGCA | C | x | 35003_403_2011 | UnannotatedRegion |
| 1854 | TCGCGGCGGC.GCACCGAGCA | C | x | 35004_12_15398_XS2_1 | UnannotatedRegion |
| 1854 | TCGCGGCGGC.GCACCGAGCA | C | x | 35005_12_13952_XS2_1 | UnannotatedRegion |
| 1854 | TCGCGGCGGC.GCACCGAGCA | C | x | 35006_12_15317_XS2_1 | UnannotatedRegion |
| 1854 | TCGCGGCGGC.GCACCGAGCA | C | x | 35007_12_15047_XS2_1 | UnannotatedRegion |
| 1854 | TCGCGGCGGC.GCACCGAGCA | C | x | 35008_12_15661_XS2_1 | UnannotatedRegion |
| 1854 | TCGCGGCGGC.GCACCGAGCA | C | x | 35009_12_15657_XS2_1 | UnannotatedRegion |
| 1854 | TCGCGGCGGC.GCACCGAGCA | C | x | 35010_12_17186_XS2_1 | UnannotatedRegion |
| 1854 | TCGCGGCGGC.GCACCGAGCA | C | x | 35011_12_17194_XS2_1 | UnannotatedRegion |
| 1854 | TCGCGGCGGC.GCACCGAGCA | C | x | 35012_12_17009_XS2_1 | UnannotatedRegion |
| 1854 | TCGCGGCGGC.GCACCGAGCA | C | x | 35013_12_14973_XS2_1 | UnannotatedRegion |
|  |  |  |  |  |  |
| 1870 | TCGTTCATAT.GCTTCCTTAA | C | 2063679 R | 19260_WUE_2594 | UnannotatedRegion |
| 1870 | TCGTTCATAT.GCTTCCTTAA | T | x | 34990_Tchad54_11 | UnannotatedRegion |
| 1870 | TCGTTCATAT.GCTTCCTTAA | C | x | 34991_Tchad78_11 | UnannotatedRegion |
| 1870 | TCGTTCATAT.GCTTCCTTAA | C | x | 34992_Tchad95_11 | UnannotatedRegion |
| 1870 | TCGTTCATAT.GCTTCCTTAA | C | x | 34994_Tchad106_11 | UnannotatedRegion |
| 1870 | TCGTTCATAT.GCTTCCTTAA | C | x | 34995_8_2011 | UnannotatedRegion |
| 1870 | TCGTTCATAT.GCTTCCTTAA | C | x | 34996_10_2011 | UnannotatedRegion |
| 1870 | TCGTTCATAT.GCTTCCTTAA | T | x | 34997_39_2011 | UnannotatedRegion |
| 1870 | TCGTTCATAT.GCTTCCTTAA | T | x | 34998_59_2011 | UnannotatedRegion |
| 1870 | TCGTTCATAT.GCTTCCTTAA | T | x | 34999_63_2011 | UnannotatedRegion |
| 1870 | TCGTTCATAT.GCTTCCTTAA | T | x | 35000_75_2011 | UnannotatedRegion |
| 1870 | TCGTTCATAT.GCTTCCTTAA | T | x | 35001_76_2011 | UnannotatedRegion |
| 1870 | TCGTTCATAT.GCTTCCTTAA | T | x | 35002_120_2011 | UnannotatedRegion |
| 1870 | TCGTTCATAT.GCTTCCTTAA | C | x | 35003_403_2011 | UnannotatedRegion |
| 1870 | TCGTTCATAT.GCTTCCTTAA | T | x | 35004_12_15398_XS2_1 | UnannotatedRegion |
| 1870 | TCGTTCATAT.GCTTCCTTAA | T | x | 35005_12_13952_XS2_1 | UnannotatedRegion |
| 1870 | TCGTTCATAT.GCTTCCTTAA | T | x | 35006_12_15317_XS2_1 | UnannotatedRegion |
| 1870 | TCGTTCATAT.GCTTCCTTAA | C | x | 35007_12_15047_XS2_1 | UnannotatedRegion |
| 1870 | TCGTTCATAT.GCTTCCTTAA | C | x | 35008_12_15661_XS2_1 | UnannotatedRegion |
| 1870 | TCGTTCATAT.GCTTCCTTAA | C | x | 35009_12_15657_XS2_1 | UnannotatedRegion |
| 1870 | TCGTTCATAT.GCTTCCTTAA | T | x | 35010_12_17186_XS2_1 | UnannotatedRegion |
| 1870 | TCGTTCATAT.GCTTCCTTAA | T | x | 35011_12_17194_XS2_1 | UnannotatedRegion |
| 1870 | TCGTTCATAT.GCTTCCTTAA | T | x | 35012_12_17009_XS2_1 | UnannotatedRegion |
| 1870 | TCGTTCATAT.GCTTCCTTAA | C | x | 35013_12_14973_XS2_1 | UnannotatedRegion |
|  |  |  |  |  |  |
| 1872 | TCTGAAACAG.AGATGTTTCA | T | 1254523 R | 19260_WUE_2594 | UnannotatedRegion |
| 1872 | TCTGAAACAG.AGATGTTTCA | C | x | 34990_Tchad54_11 | UnannotatedRegion |
| 1872 | TCTGAAACAG.AGATGTTTCA | C | x | 34991_Tchad78_11 | UnannotatedRegion |
| 1872 | TCTGAAACAG.AGATGTTTCA | C | x | 34992_Tchad95_11 | UnannotatedRegion |
| 1872 | TCTGAAACAG.AGATGTTTCA | C | x | 34994_Tchad106_11 | UnannotatedRegion |
| 1872 | TCTGAAACAG.AGATGTTTCA | C | x | 34995_8_2011 | UnannotatedRegion |
| 1872 | TCTGAAACAG.AGATGTTTCA | C | x | 34996_10_2011 | UnannotatedRegion |
| 1872 | TCTGAAACAG.AGATGTTTCA | C | x | 34997_39_2011 | UnannotatedRegion |
| 1872 | TCTGAAACAG.AGATGTTTCA | C | x | 34998_59_2011 | UnannotatedRegion |
| 1872 | TCTGAAACAG.AGATGTTTCA | C | x | 34999_63_2011 | UnannotatedRegion |
| 1872 | TCTGAAACAG.AGATGTTTCA | C | x | 35000_75_2011 | UnannotatedRegion |
| 1872 | TCTGAAACAG.AGATGTTTCA | C | x | 35001_76_2011 | UnannotatedRegion |
| 1872 | TCTGAAACAG.AGATGTTTCA | C | x | 35002_120_2011 | UnannotatedRegion |
| 1872 | TCTGAAACAG.AGATGTTTCA | C | x | 35003_403_2011 | UnannotatedRegion |
| 1872 | TCTGAAACAG.AGATGTTTCA | C | x | 35004_12_15398_XS2_1 | UnannotatedRegion |
| 1872 | TCTGAAACAG.AGATGTTTCA | C | x | 35005_12_13952_XS2_1 | UnannotatedRegion |
| 1872 | TCTGAAACAG.AGATGTTTCA | C | x | 35006_12_15317_XS2_1 | UnannotatedRegion |
| 1872 | TCTGAAACAG.AGATGTTTCA | C | x | 35007_12_15047_XS2_1 | UnannotatedRegion |
| 1872 | TCTGAAACAG.AGATGTTTCA | C | x | 35008_12_15661_XS2_1 | UnannotatedRegion |
| 1872 | TCTGAAACAG.AGATGTTTCA | C | x | 35009_12_15657_XS2_1 | UnannotatedRegion |
| 1872 | TCTGAAACAG.AGATGTTTCA | C | x | 35010_12_17186_XS2_1 | UnannotatedRegion |
| 1872 | TCTGAAACAG.AGATGTTTCA | C | x | 35011_12_17194_XS2_1 | UnannotatedRegion |
| 1872 | TCTGAAACAG.AGATGTTTCA | C | x | 35012_12_17009_XS2_1 | UnannotatedRegion |
| 1872 | TCTGAAACAG.AGATGTTTCA | C | x | 35013_12_14973_XS2_1 | UnannotatedRegion |
|  |  |  |  |  |  |
| 1880 | TGAATTGGGT.TATATGAACA | T | 1733175 R | 19260_WUE_2594 | UnannotatedRegion |
| 1880 | TGAATTGGGT.TATATGAACA | C | x | 34990_Tchad54_11 | UnannotatedRegion |
| 1880 | TGAATTGGGT.TATATGAACA | C | x | 34991_Tchad78_11 | UnannotatedRegion |
| 1880 | TGAATTGGGT.TATATGAACA | C | x | 34992_Tchad95_11 | UnannotatedRegion |
| 1880 | TGAATTGGGT.TATATGAACA | C | x | 34994_Tchad106_11 | UnannotatedRegion |
| 1880 | TGAATTGGGT.TATATGAACA | C | x | 34995_8_2011 | UnannotatedRegion |
| 1880 | TGAATTGGGT.TATATGAACA | C | x | 34996_10_2011 | UnannotatedRegion |
| 1880 | TGAATTGGGT.TATATGAACA | C | x | 34997_39_2011 | UnannotatedRegion |
| 1880 | TGAATTGGGT.TATATGAACA | C | x | 34998_59_2011 | UnannotatedRegion |
| 1880 | TGAATTGGGT.TATATGAACA | C | x | 34999_63_2011 | UnannotatedRegion |
| 1880 | TGAATTGGGT.TATATGAACA | C | x | 35000_75_2011 | UnannotatedRegion |
| 1880 | TGAATTGGGT.TATATGAACA | C | x | 35001_76_2011 | UnannotatedRegion |
| 1880 | TGAATTGGGT.TATATGAACA | C | x | 35002_120_2011 | UnannotatedRegion |
| 1880 | TGAATTGGGT.TATATGAACA | C | x | 35003_403_2011 | UnannotatedRegion |
| 1880 | TGAATTGGGT.TATATGAACA | C | x | 35004_12_15398_XS2_1 | UnannotatedRegion |
| 1880 | TGAATTGGGT.TATATGAACA | C | x | 35005_12_13952_XS2_1 | UnannotatedRegion |
| 1880 | TGAATTGGGT.TATATGAACA | C | x | 35006_12_15317_XS2_1 | UnannotatedRegion |
| 1880 | TGAATTGGGT.TATATGAACA | C | x | 35007_12_15047_XS2_1 | UnannotatedRegion |
| 1880 | TGAATTGGGT.TATATGAACA | C | x | 35008_12_15661_XS2_1 | UnannotatedRegion |
| 1880 | TGAATTGGGT.TATATGAACA | C | x | 35009_12_15657_XS2_1 | UnannotatedRegion |
| 1880 | TGAATTGGGT.TATATGAACA | C | x | 35010_12_17186_XS2_1 | UnannotatedRegion |
| 1880 | TGAATTGGGT.TATATGAACA | C | x | 35011_12_17194_XS2_1 | UnannotatedRegion |
| 1880 | TGAATTGGGT.TATATGAACA | C | x | 35012_12_17009_XS2_1 | UnannotatedRegion |
| 1880 | TGAATTGGGT.TATATGAACA | C | x | 35013_12_14973_XS2_1 | UnannotatedRegion |
|  |  |  |  |  |  |
| 1889 | TGCCGCAGCA.CCGTCTTCCA | T | 2160743 R | 19260_WUE_2594 | UnannotatedRegion |
| 1889 | TGCCGCAGCA.CCGTCTTCCA | T | x | 34990_Tchad54_11 | UnannotatedRegion |
| 1889 | TGCCGCAGCA.CCGTCTTCCA | T | x | 34991_Tchad78_11 | UnannotatedRegion |
| 1889 | TGCCGCAGCA.CCGTCTTCCA | C | x | 34992_Tchad95_11 | UnannotatedRegion |
| 1889 | TGCCGCAGCA.CCGTCTTCCA | C | x | 34994_Tchad106_11 | UnannotatedRegion |
| 1889 | TGCCGCAGCA.CCGTCTTCCA | T | x | 34995_8_2011 | UnannotatedRegion |
| 1889 | TGCCGCAGCA.CCGTCTTCCA | T | x | 34996_10_2011 | UnannotatedRegion |
| 1889 | TGCCGCAGCA.CCGTCTTCCA | T | x | 34997_39_2011 | UnannotatedRegion |
| 1889 | TGCCGCAGCA.CCGTCTTCCA | T | x | 34998_59_2011 | UnannotatedRegion |
| 1889 | TGCCGCAGCA.CCGTCTTCCA | T | x | 34999_63_2011 | UnannotatedRegion |
| 1889 | TGCCGCAGCA.CCGTCTTCCA | T | x | 35000_75_2011 | UnannotatedRegion |
| 1889 | TGCCGCAGCA.CCGTCTTCCA | T | x | 35001_76_2011 | UnannotatedRegion |
| 1889 | TGCCGCAGCA.CCGTCTTCCA | T | x | 35002_120_2011 | UnannotatedRegion |
| 1889 | TGCCGCAGCA.CCGTCTTCCA | T | x | 35003_403_2011 | UnannotatedRegion |
| 1889 | TGCCGCAGCA.CCGTCTTCCA | T | x | 35004_12_15398_XS2_1 | UnannotatedRegion |
| 1889 | TGCCGCAGCA.CCGTCTTCCA | T | x | 35005_12_13952_XS2_1 | UnannotatedRegion |
| 1889 | TGCCGCAGCA.CCGTCTTCCA | T | x | 35006_12_15317_XS2_1 | UnannotatedRegion |
| 1889 | TGCCGCAGCA.CCGTCTTCCA | C | x | 35007_12_15047_XS2_1 | UnannotatedRegion |
| 1889 | TGCCGCAGCA.CCGTCTTCCA | C | x | 35008_12_15661_XS2_1 | UnannotatedRegion |
| 1889 | TGCCGCAGCA.CCGTCTTCCA | C | x | 35009_12_15657_XS2_1 | UnannotatedRegion |
| 1889 | TGCCGCAGCA.CCGTCTTCCA | T | x | 35010_12_17186_XS2_1 | UnannotatedRegion |
| 1889 | TGCCGCAGCA.CCGTCTTCCA | T | x | 35011_12_17194_XS2_1 | UnannotatedRegion |
| 1889 | TGCCGCAGCA.CCGTCTTCCA | T | x | 35012_12_17009_XS2_1 | UnannotatedRegion |
| 1889 | TGCCGCAGCA.CCGTCTTCCA | C | x | 35013_12_14973_XS2_1 | UnannotatedRegion |
|  |  |  |  |  |  |
| 1895 | TGGAAAGAAG.CGAACCGAAA | G | 2037462 F | 19260_WUE_2594 | UnannotatedRegion |
| 1895 | TGGAAAGAAG.CGAACCGAAA | G | x | 34990_Tchad54_11 | UnannotatedRegion |
| 1895 | TGGAAAGAAG.CGAACCGAAA | A | x | 34991_Tchad78_11 | UnannotatedRegion |
| 1895 | TGGAAAGAAG.CGAACCGAAA | A | x | 34992_Tchad95_11 | UnannotatedRegion |
| 1895 | TGGAAAGAAG.CGAACCGAAA | A | x | 34994_Tchad106_11 | UnannotatedRegion |
| 1895 | TGGAAAGAAG.CGAACCGAAA | G | x | 34995_8_2011 | UnannotatedRegion |
| 1895 | TGGAAAGAAG.CGAACCGAAA | G | x | 34996_10_2011 | UnannotatedRegion |
| 1895 | TGGAAAGAAG.CGAACCGAAA | G | x | 34997_39_2011 | UnannotatedRegion |
| 1895 | TGGAAAGAAG.CGAACCGAAA | G | x | 34998_59_2011 | UnannotatedRegion |
| 1895 | TGGAAAGAAG.CGAACCGAAA | G | x | 34999_63_2011 | UnannotatedRegion |
| 1895 | TGGAAAGAAG.CGAACCGAAA | G | x | 35000_75_2011 | UnannotatedRegion |
| 1895 | TGGAAAGAAG.CGAACCGAAA | G | x | 35001_76_2011 | UnannotatedRegion |
| 1895 | TGGAAAGAAG.CGAACCGAAA | G | x | 35002_120_2011 | UnannotatedRegion |
| 1895 | TGGAAAGAAG.CGAACCGAAA | A | x | 35003_403_2011 | UnannotatedRegion |
| 1895 | TGGAAAGAAG.CGAACCGAAA | G | x | 35004_12_15398_XS2_1 | UnannotatedRegion |
| 1895 | TGGAAAGAAG.CGAACCGAAA | G | x | 35005_12_13952_XS2_1 | UnannotatedRegion |
| 1895 | TGGAAAGAAG.CGAACCGAAA | G | x | 35006_12_15317_XS2_1 | UnannotatedRegion |
| 1895 | TGGAAAGAAG.CGAACCGAAA | A | x | 35007_12_15047_XS2_1 | UnannotatedRegion |
| 1895 | TGGAAAGAAG.CGAACCGAAA | A | x | 35008_12_15661_XS2_1 | UnannotatedRegion |
| 1895 | TGGAAAGAAG.CGAACCGAAA | A | x | 35009_12_15657_XS2_1 | UnannotatedRegion |
| 1895 | TGGAAAGAAG.CGAACCGAAA | G | x | 35010_12_17186_XS2_1 | UnannotatedRegion |
| 1895 | TGGAAAGAAG.CGAACCGAAA | G | x | 35011_12_17194_XS2_1 | UnannotatedRegion |
| 1895 | TGGAAAGAAG.CGAACCGAAA | G | x | 35012_12_17009_XS2_1 | UnannotatedRegion |
| 1895 | TGGAAAGAAG.CGAACCGAAA | A | x | 35013_12_14973_XS2_1 | UnannotatedRegion |
|  |  |  |  |  |  |
| 1910 | TTAATTACAC.CCACTACAAA | G | 2051759 F | 19260_WUE_2594 | UnannotatedRegion |
| 1910 | TTAATTACAC.CCACTACAAA | A | x | 34990_Tchad54_11 | UnannotatedRegion |
| 1910 | TTAATTACAC.CCACTACAAA | A | x | 34991_Tchad78_11 | UnannotatedRegion |
| 1910 | TTAATTACAC.CCACTACAAA | A | x | 34992_Tchad95_11 | UnannotatedRegion |
| 1910 | TTAATTACAC.CCACTACAAA | A | x | 34995_8_2011 | UnannotatedRegion |
| 1910 | TTAATTACAC.CCACTACAAA | A | x | 34996_10_2011 | UnannotatedRegion |
| 1910 | TTAATTACAC.CCACTACAAA | A | x | 34997_39_2011 | UnannotatedRegion |
| 1910 | TTAATTACAC.CCACTACAAA | A | x | 34998_59_2011 | UnannotatedRegion |
| 1910 | TTAATTACAC.CCACTACAAA | A | x | 34999_63_2011 | UnannotatedRegion |
| 1910 | TTAATTACAC.CCACTACAAA | A | x | 35000_75_2011 | UnannotatedRegion |
| 1910 | TTAATTACAC.CCACTACAAA | A | x | 35001_76_2011 | UnannotatedRegion |
| 1910 | TTAATTACAC.CCACTACAAA | A | x | 35002_120_2011 | UnannotatedRegion |
| 1910 | TTAATTACAC.CCACTACAAA | A | x | 35003_403_2011 | UnannotatedRegion |
| 1910 | TTAATTACAC.CCACTACAAA | A | x | 35004_12_15398_XS2_1 | UnannotatedRegion |
| 1910 | TTAATTACAC.CCACTACAAA | A | x | 35005_12_13952_XS2_1 | UnannotatedRegion |
| 1910 | TTAATTACAC.CCACTACAAA | A | x | 35006_12_15317_XS2_1 | UnannotatedRegion |
| 1910 | TTAATTACAC.CCACTACAAA | A | x | 35007_12_15047_XS2_1 | UnannotatedRegion |
| 1910 | TTAATTACAC.CCACTACAAA | A | x | 35008_12_15661_XS2_1 | UnannotatedRegion |
| 1910 | TTAATTACAC.CCACTACAAA | A | x | 35009_12_15657_XS2_1 | UnannotatedRegion |
| 1910 | TTAATTACAC.CCACTACAAA | A | x | 35010_12_17186_XS2_1 | UnannotatedRegion |
| 1910 | TTAATTACAC.CCACTACAAA | A | x | 35011_12_17194_XS2_1 | UnannotatedRegion |
| 1910 | TTAATTACAC.CCACTACAAA | A | x | 35012_12_17009_XS2_1 | UnannotatedRegion |
| 1910 | TTAATTACAC.CCACTACAAA | A | x | 35013_12_14973_XS2_1 | UnannotatedRegion |
|  |  |  |  |  |  |
| 1912 | TTATTAGAAT.TATTTGCAAA | C | 1270275 F | 19260_WUE_2594 | UnannotatedRegion |
| 1912 | TTATTAGAAT.TATTTGCAAA | T | x | 34990_Tchad54_11 | UnannotatedRegion |
| 1912 | TTATTAGAAT.TATTTGCAAA | T | x | 34991_Tchad78_11 | UnannotatedRegion |
| 1912 | TTATTAGAAT.TATTTGCAAA | T | x | 34992_Tchad95_11 | UnannotatedRegion |
| 1912 | TTATTAGAAT.TATTTGCAAA | T | x | 34995_8_2011 | UnannotatedRegion |
| 1912 | TTATTAGAAT.TATTTGCAAA | T | x | 34996_10_2011 | UnannotatedRegion |
| 1912 | TTATTAGAAT.TATTTGCAAA | T | x | 34997_39_2011 | UnannotatedRegion |
| 1912 | TTATTAGAAT.TATTTGCAAA | T | x | 34998_59_2011 | UnannotatedRegion |
| 1912 | TTATTAGAAT.TATTTGCAAA | T | x | 34999_63_2011 | UnannotatedRegion |
| 1912 | TTATTAGAAT.TATTTGCAAA | T | x | 35000_75_2011 | UnannotatedRegion |
| 1912 | TTATTAGAAT.TATTTGCAAA | T | x | 35001_76_2011 | UnannotatedRegion |
| 1912 | TTATTAGAAT.TATTTGCAAA | T | x | 35002_120_2011 | UnannotatedRegion |
| 1912 | TTATTAGAAT.TATTTGCAAA | T | x | 35003_403_2011 | UnannotatedRegion |
| 1912 | TTATTAGAAT.TATTTGCAAA | T | x | 35004_12_15398_XS2_1 | UnannotatedRegion |
| 1912 | TTATTAGAAT.TATTTGCAAA | T | x | 35005_12_13952_XS2_1 | UnannotatedRegion |
| 1912 | TTATTAGAAT.TATTTGCAAA | T | x | 35006_12_15317_XS2_1 | UnannotatedRegion |
| 1912 | TTATTAGAAT.TATTTGCAAA | T | x | 35007_12_15047_XS2_1 | UnannotatedRegion |
| 1912 | TTATTAGAAT.TATTTGCAAA | T | x | 35008_12_15661_XS2_1 | UnannotatedRegion |
| 1912 | TTATTAGAAT.TATTTGCAAA | T | x | 35009_12_15657_XS2_1 | UnannotatedRegion |
| 1912 | TTATTAGAAT.TATTTGCAAA | T | x | 35010_12_17186_XS2_1 | UnannotatedRegion |
| 1912 | TTATTAGAAT.TATTTGCAAA | T | x | 35011_12_17194_XS2_1 | UnannotatedRegion |
| 1912 | TTATTAGAAT.TATTTGCAAA | T | x | 35012_12_17009_XS2_1 | UnannotatedRegion |
| 1912 | TTATTAGAAT.TATTTGCAAA | T | x | 35013_12_14973_XS2_1 | UnannotatedRegion |
